# Supplementary figures and images for: RAPSYN-mediated neddylation of BCR-ABL alternatively determines the fate of Philadelphia chromosome-positive leukemia (part 1 of 5)
Source: eLife. 2024 Jun 12;12:RP88375. doi: 10.7554/eLife.88375 (PMC11168747; doi:10.7554/eLife.88375)

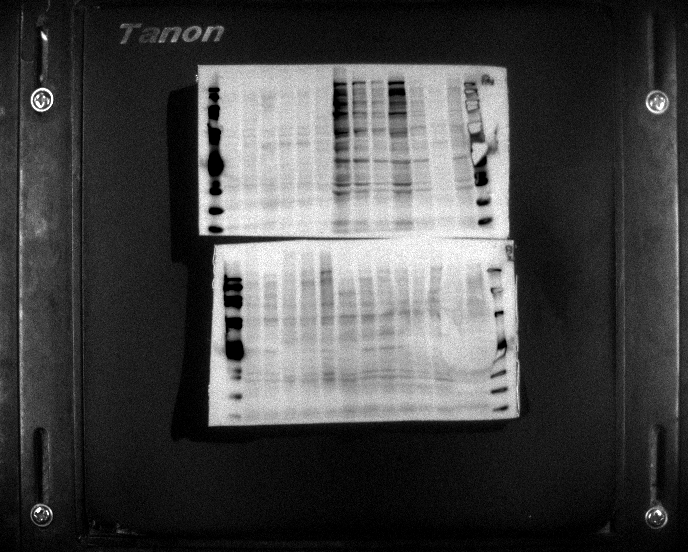

Supplement: Figure 1—source data 1. [file elife-88375-fig1-data1.zip › Figure 1-source data 1/anti-BCR-ABL.tif]

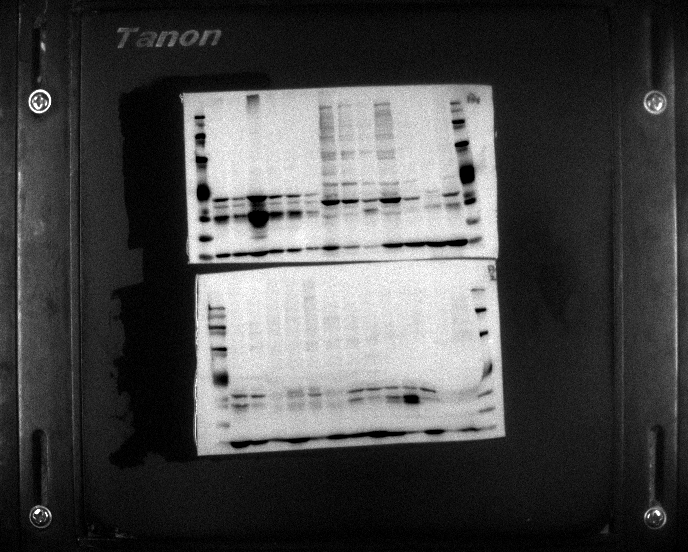

Supplement: Figure 1—source data 1. [file elife-88375-fig1-data1.zip › Figure 1-source data 1/anti-GAPDH.tif]

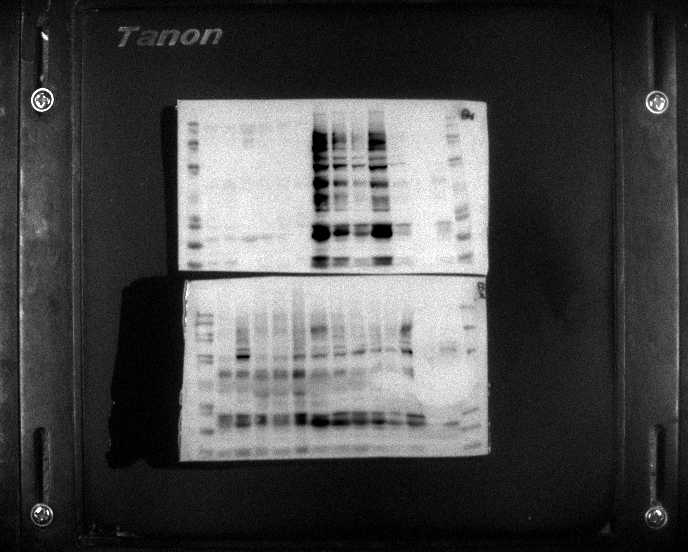

Supplement: Figure 1—source data 1. [file elife-88375-fig1-data1.zip › Figure 1-source data 1/anti-RAPSYN.tif]

A

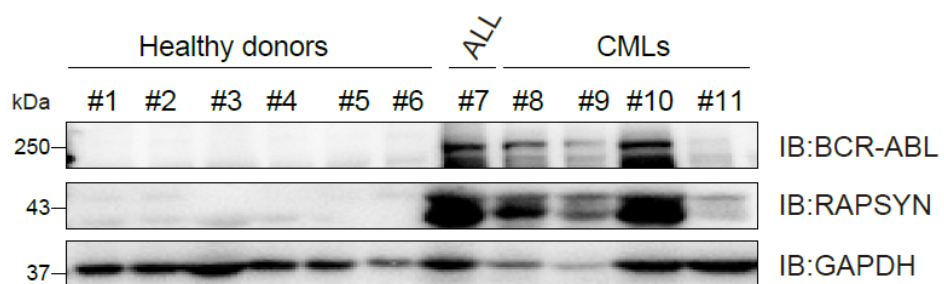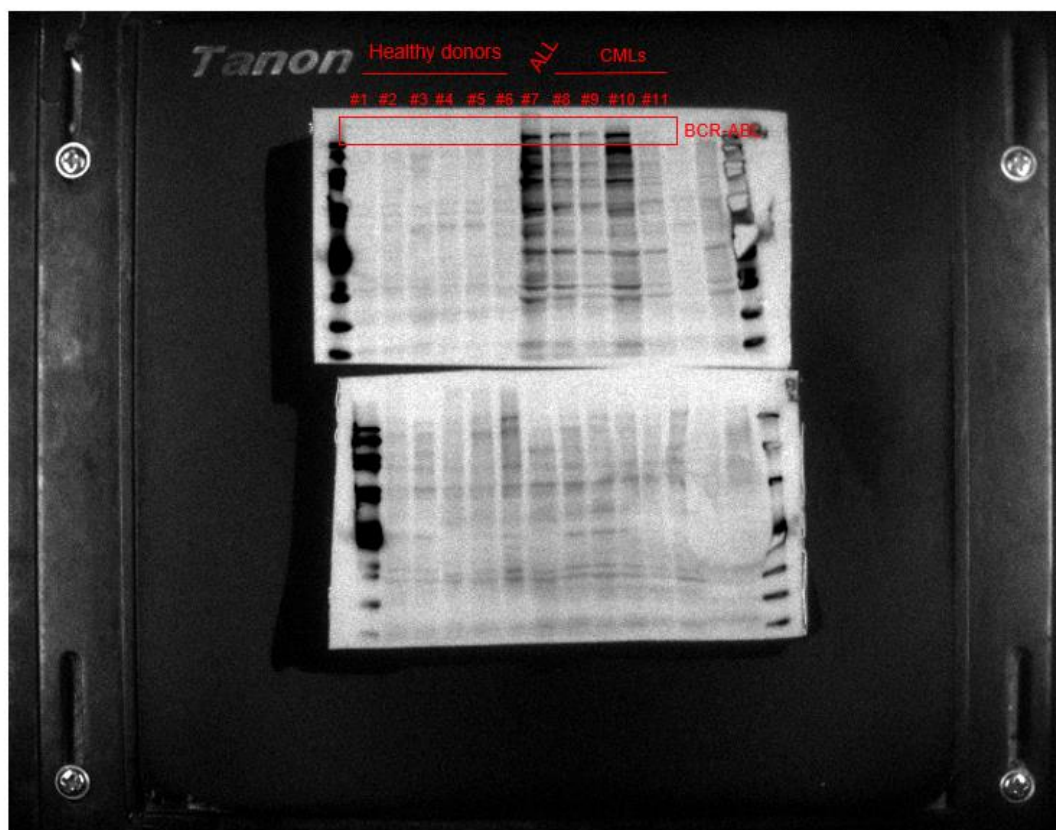

Tanon

Healthy donors

ALL

CMLs

#1 #2 #3 #4 #5 #6 #7 #8 #9 #10 #11

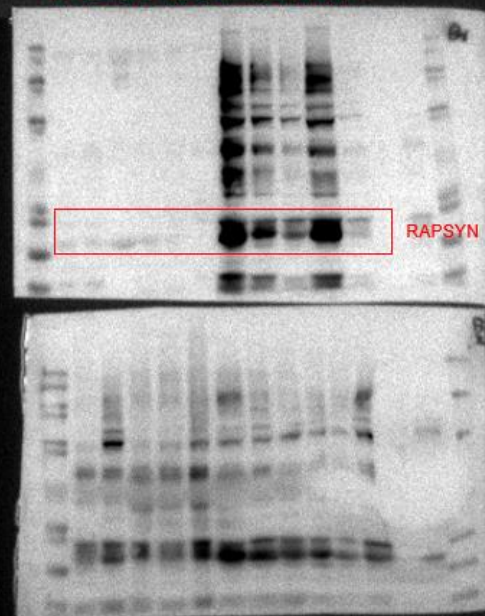

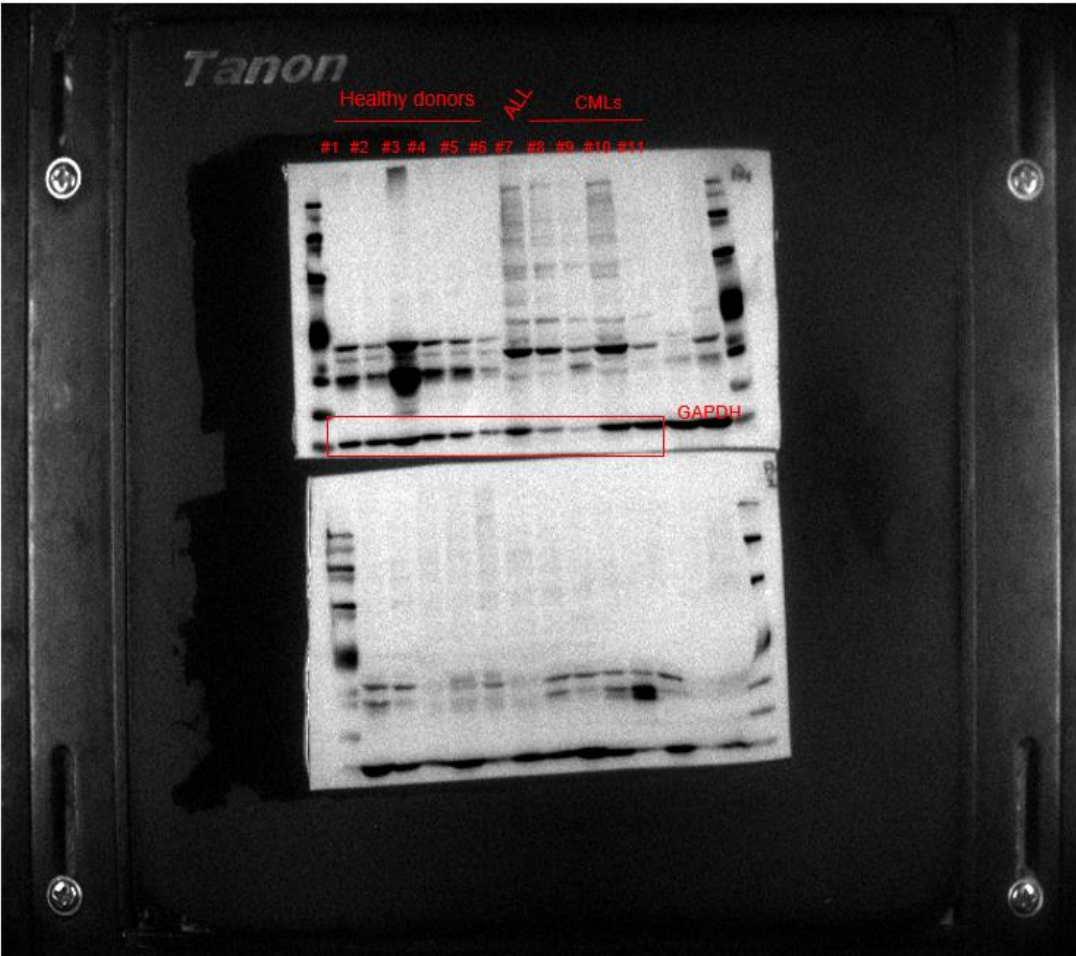

Supplement: Figure 1—source data 2. [file elife-88375-fig1-data2.zip › Figure 1-source data 2/Figure 1-source data 2.pdf]

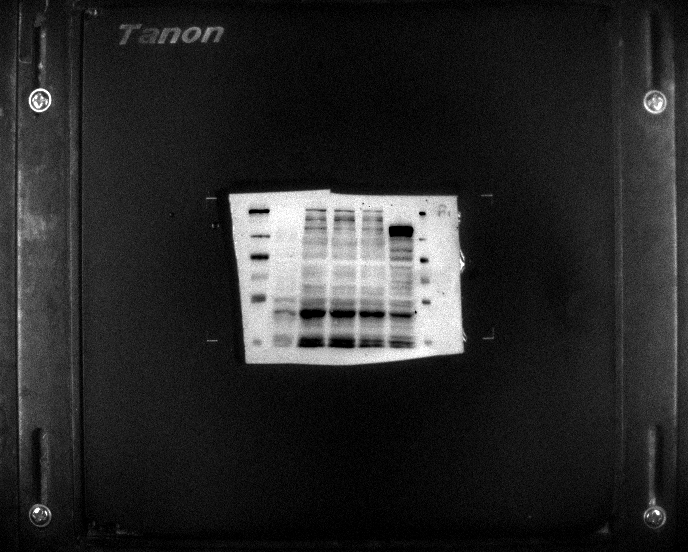

Supplement: Figure 1—source data 3. [file elife-88375-fig1-data3.zip › Figure 1-source data 3/BCR-ABL.tif]

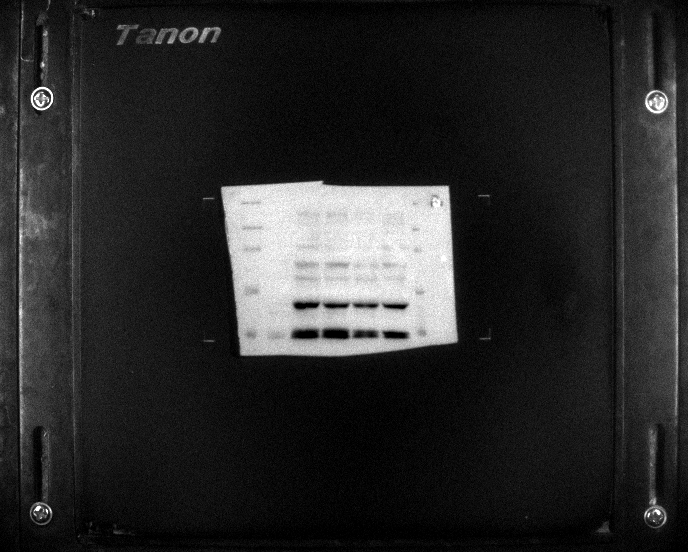

Supplement: Figure 1—source data 3. [file elife-88375-fig1-data3.zip › Figure 1-source data 3/RAPSYN.tif]

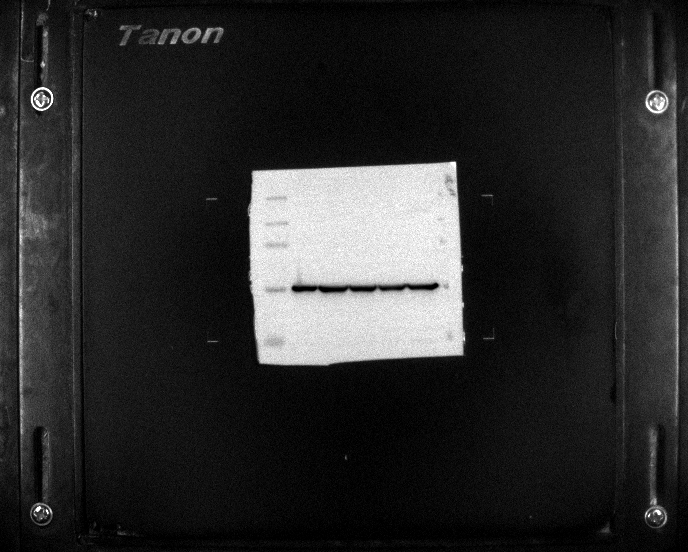

Supplement: Figure 1—source data 3. [file elife-88375-fig1-data3.zip › Figure 1-source data 3/a┬-Tubulin.tif]

B

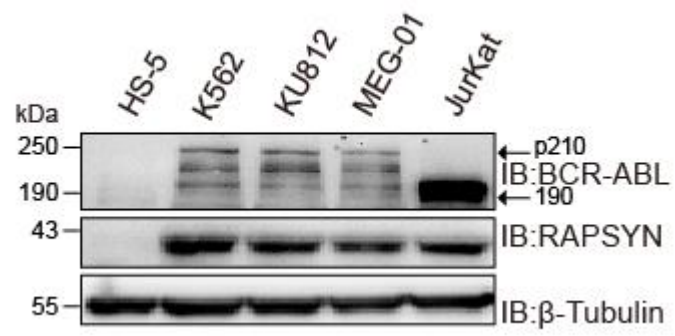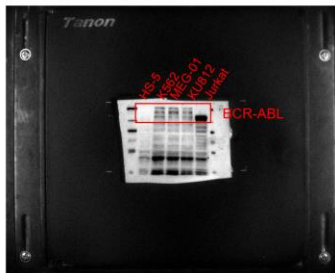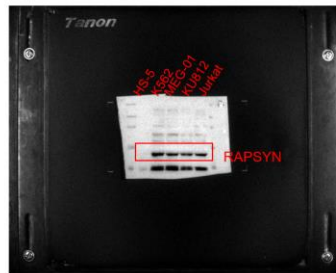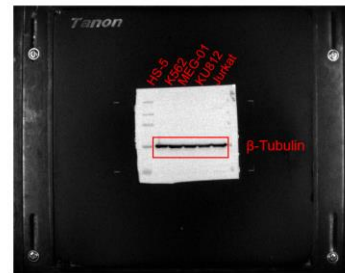

Supplement: Figure 1—source data 4. [file elife-88375-fig1-data4.zip › Figure 1-source data 4/Figure 1-source data 4.pdf]

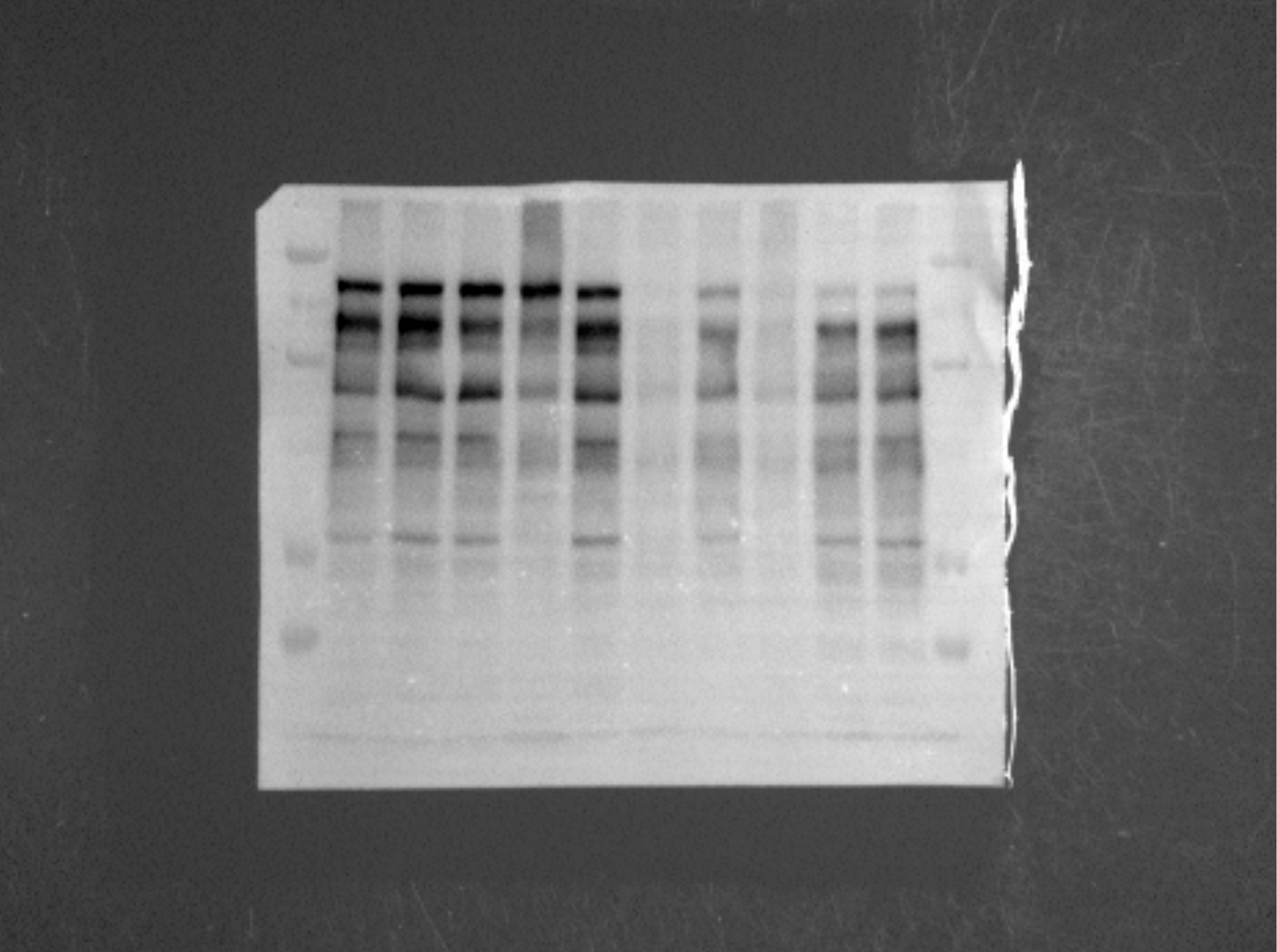

Supplement: Figure 1—source data 5. [file elife-88375-fig1-data5.zip › Figure 1-source data 5/BCR-ABL.jpg]

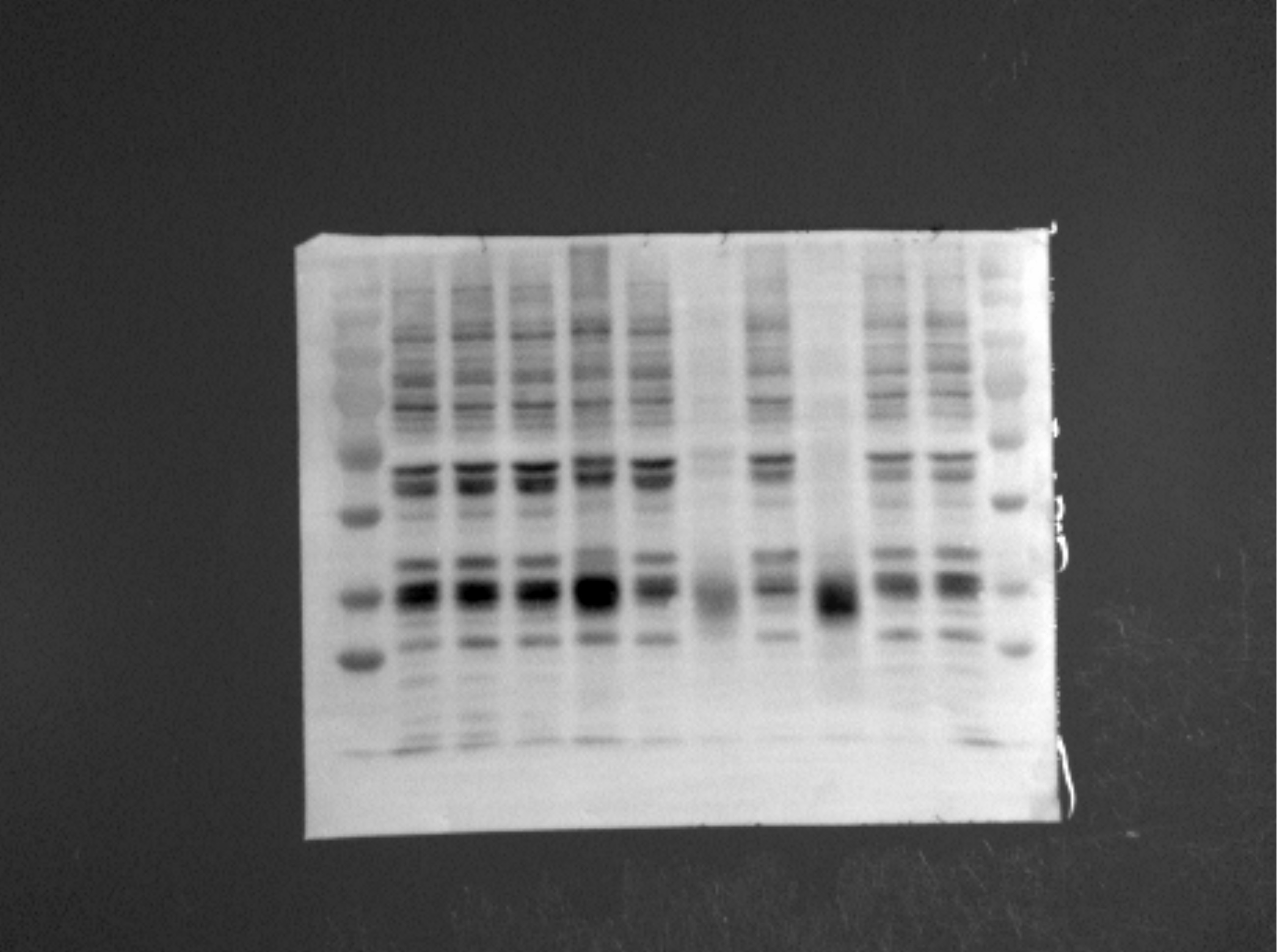

Supplement: Figure 1—source data 5. [file elife-88375-fig1-data5.zip › Figure 1-source data 5/RAPSYN.jpg]

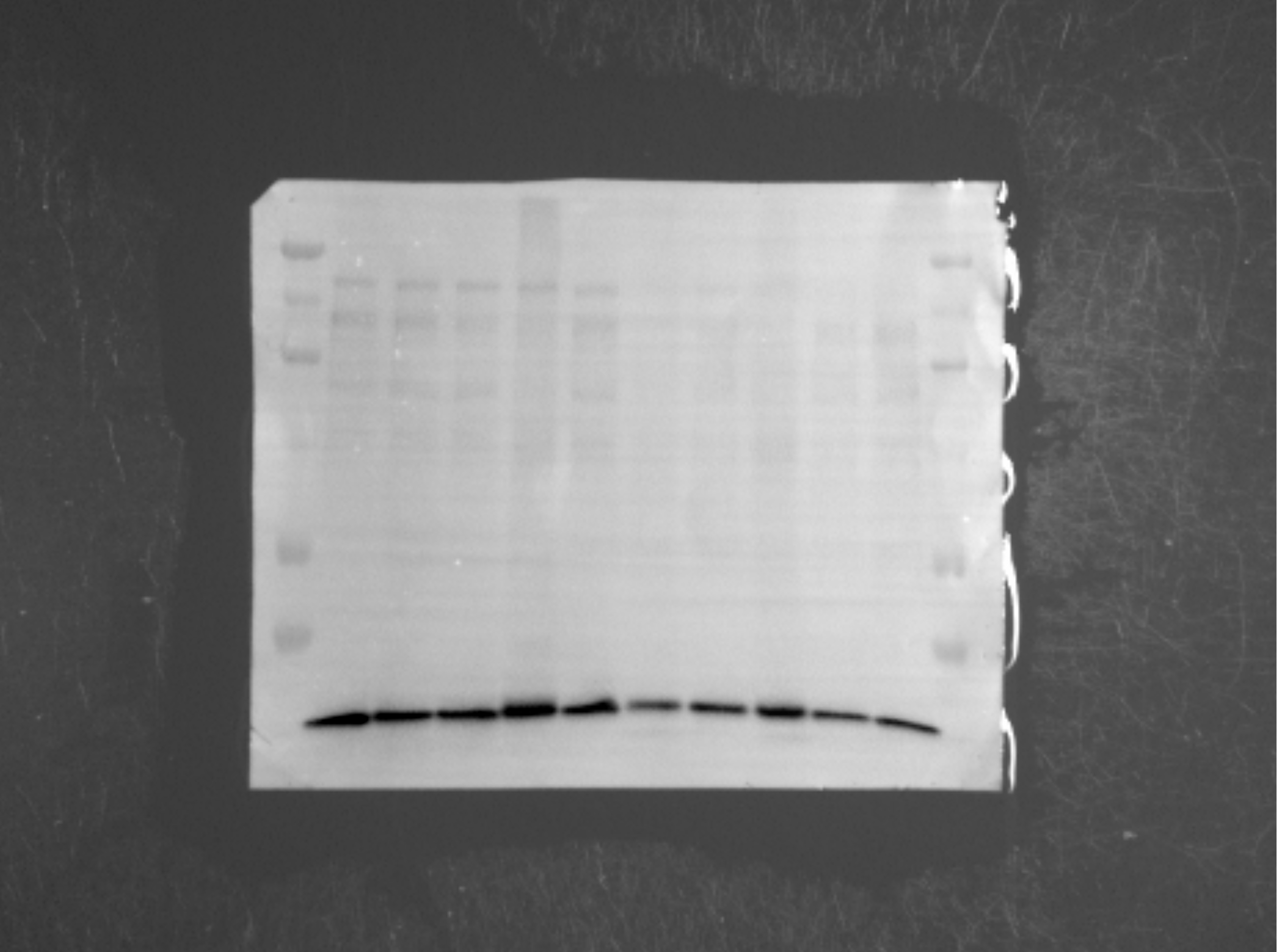

Supplement: Figure 1—source data 5. [file elife-88375-fig1-data5.zip › Figure 1-source data 5/a┬-Tubulin.jpg]

I

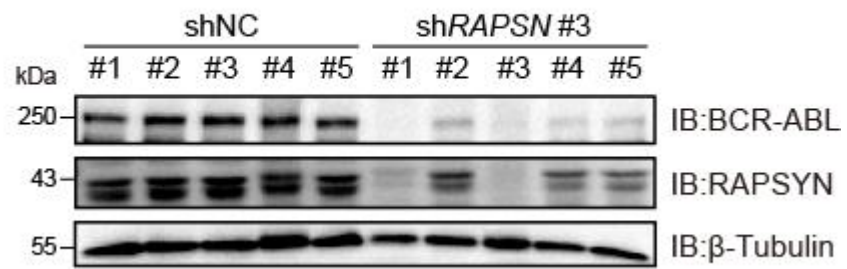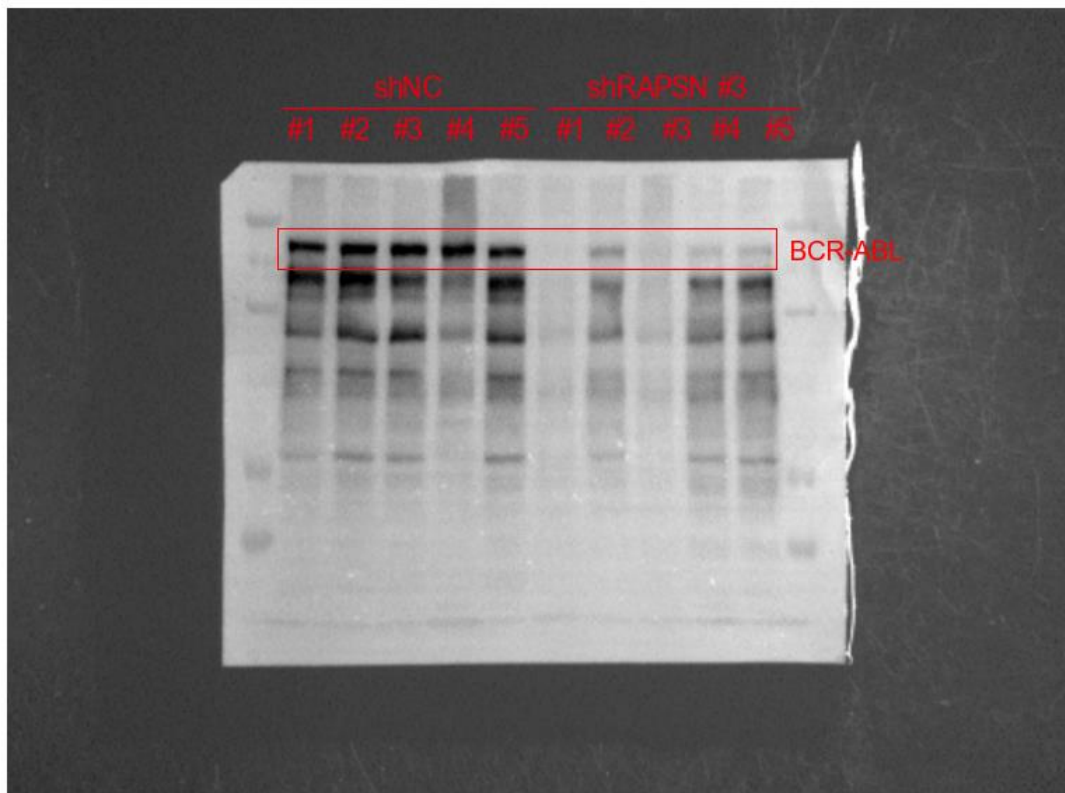

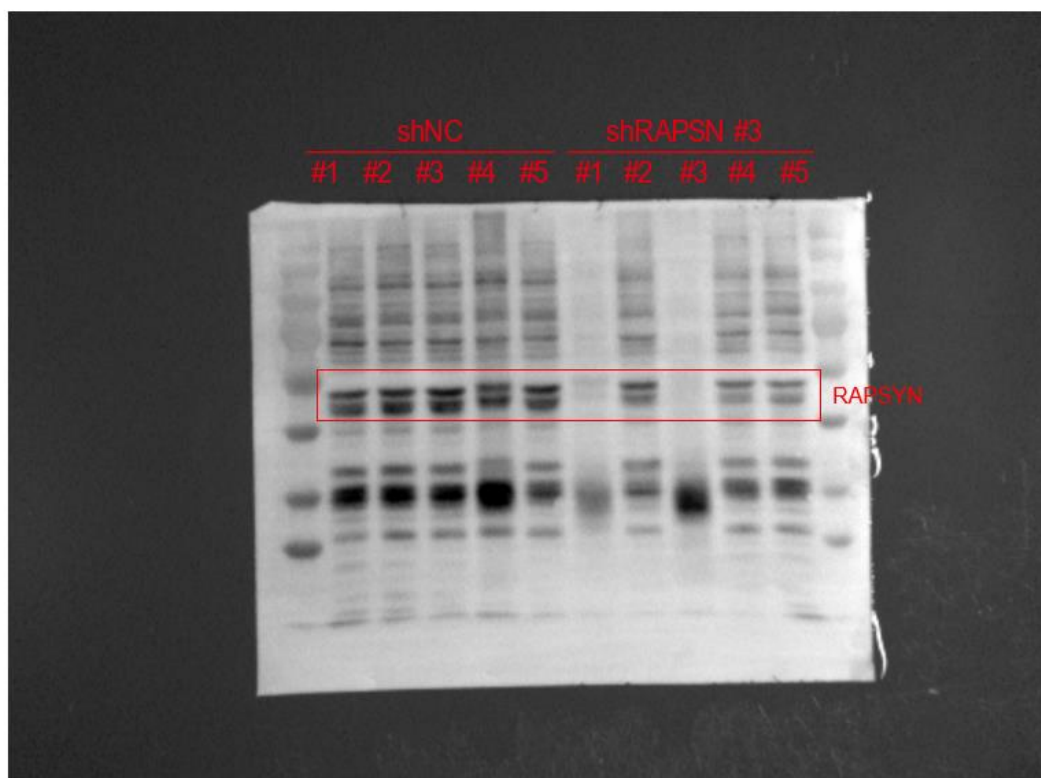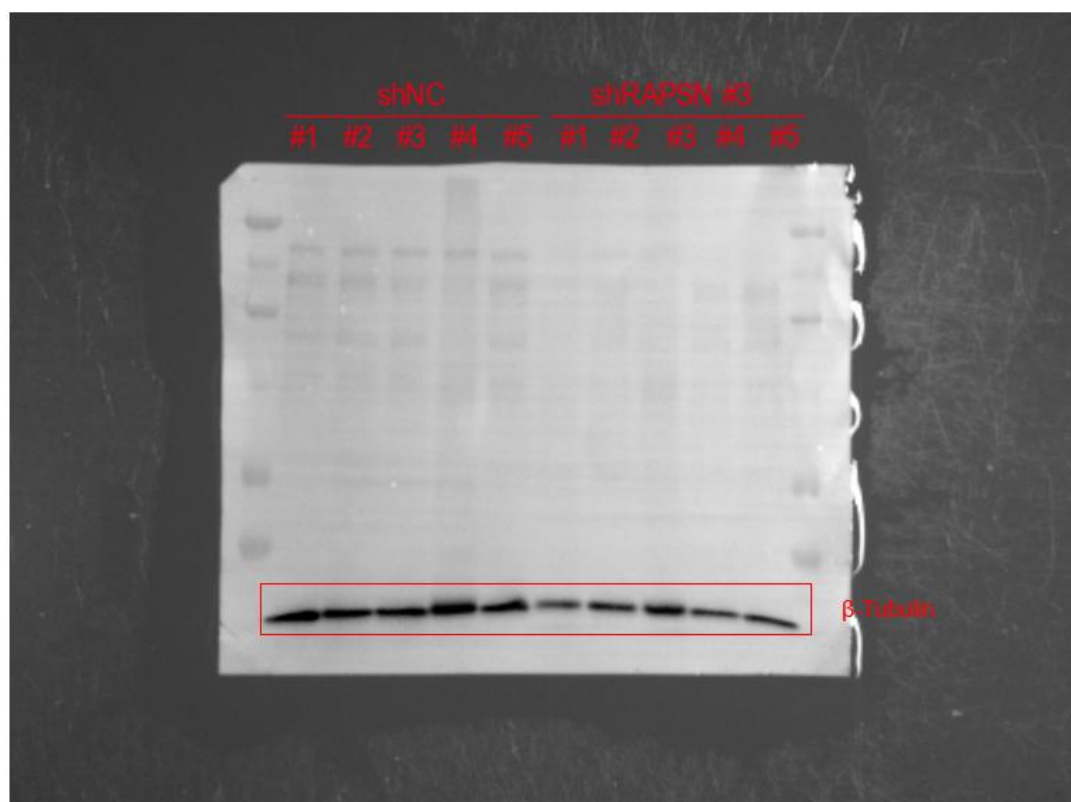

Supplement: Figure 1—source data 6. [file elife-88375-fig1-data6.zip › Figure 1-source data 6/Figure 1-source data 6.pdf]

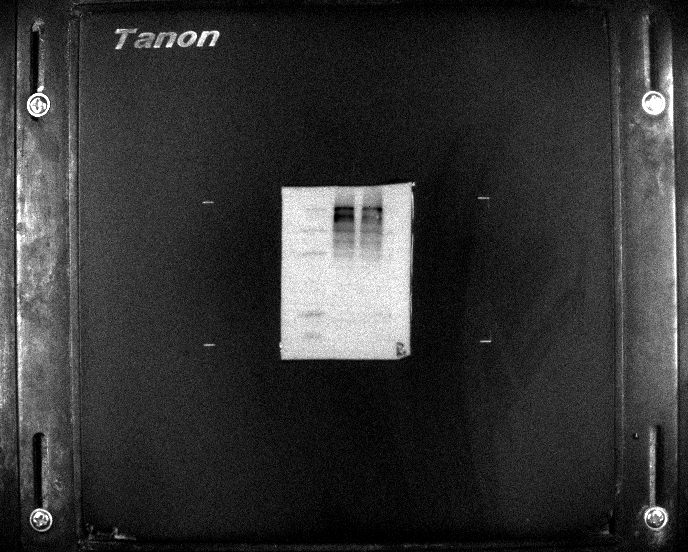

Supplement: Figure 1—source data 7. [file elife-88375-fig1-data7.zip › Figure 1-source data 7/anti-BCR-ABL.tif]

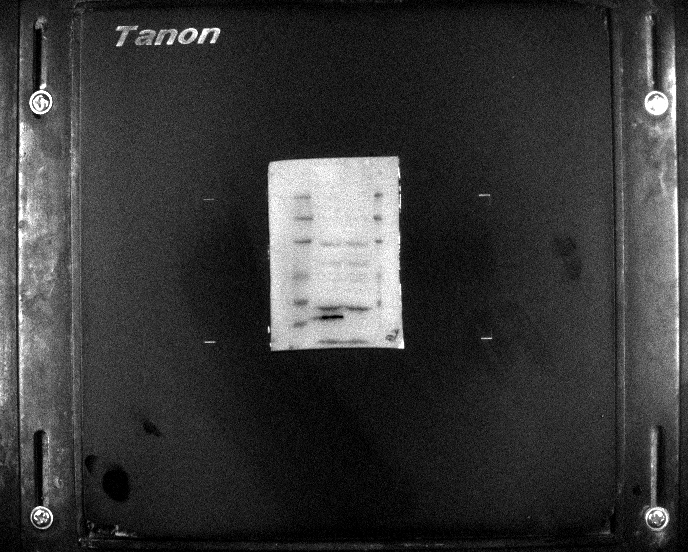

Supplement: Figure 1—source data 7. [file elife-88375-fig1-data7.zip › Figure 1-source data 7/anti-RAPSYN.tif]

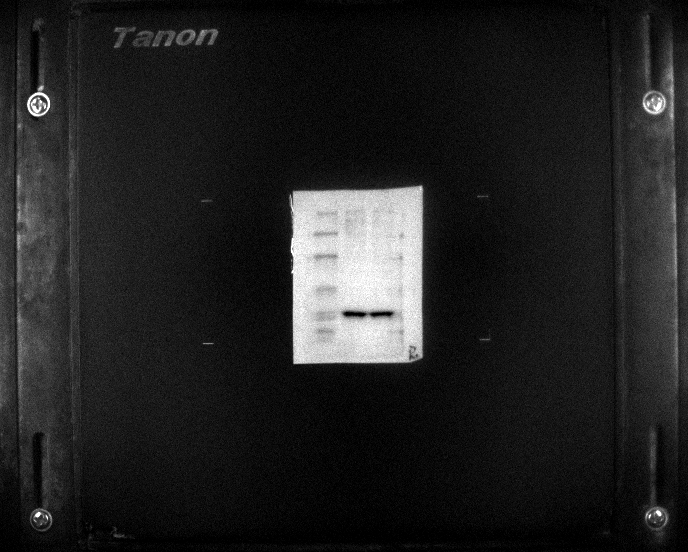

Supplement: Figure 1—source data 7. [file elife-88375-fig1-data7.zip › Figure 1-source data 7/anti-a┬-Tubulin.tif]

J

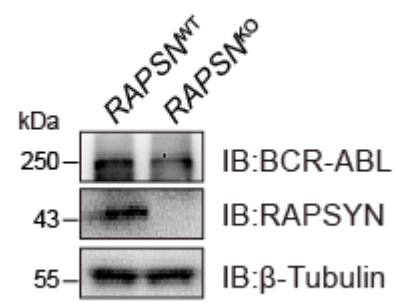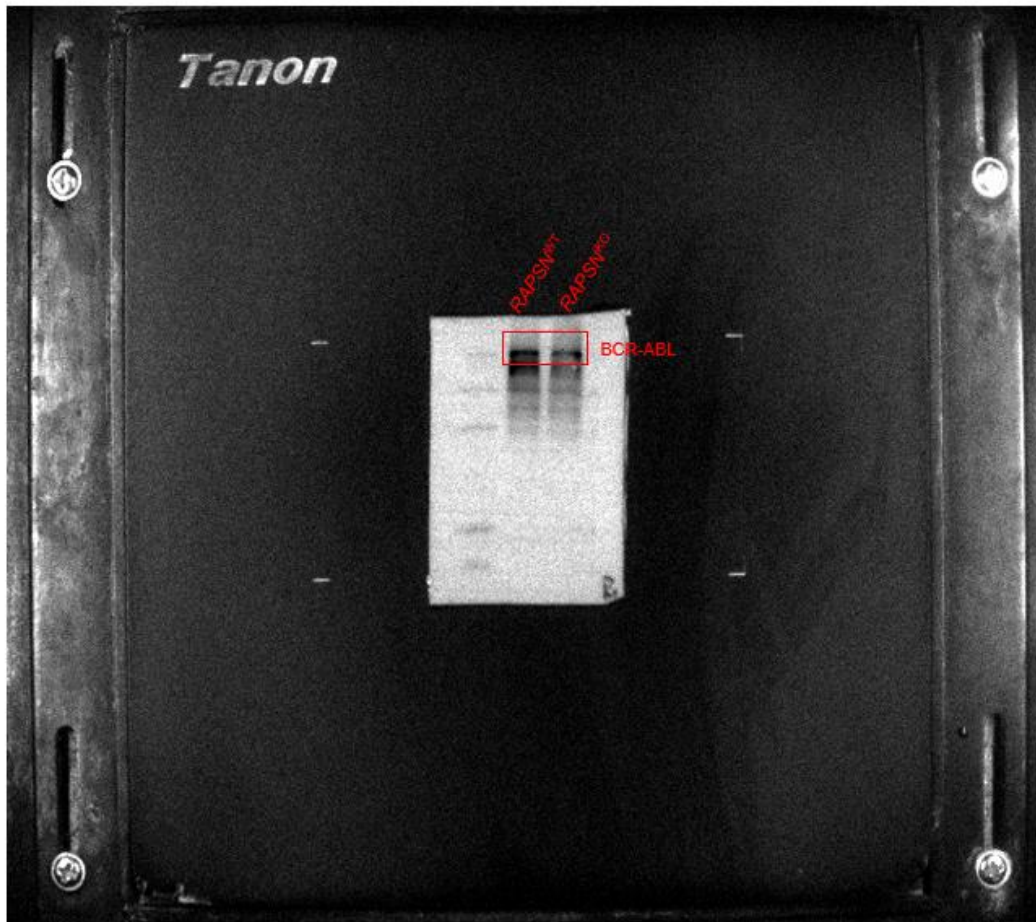

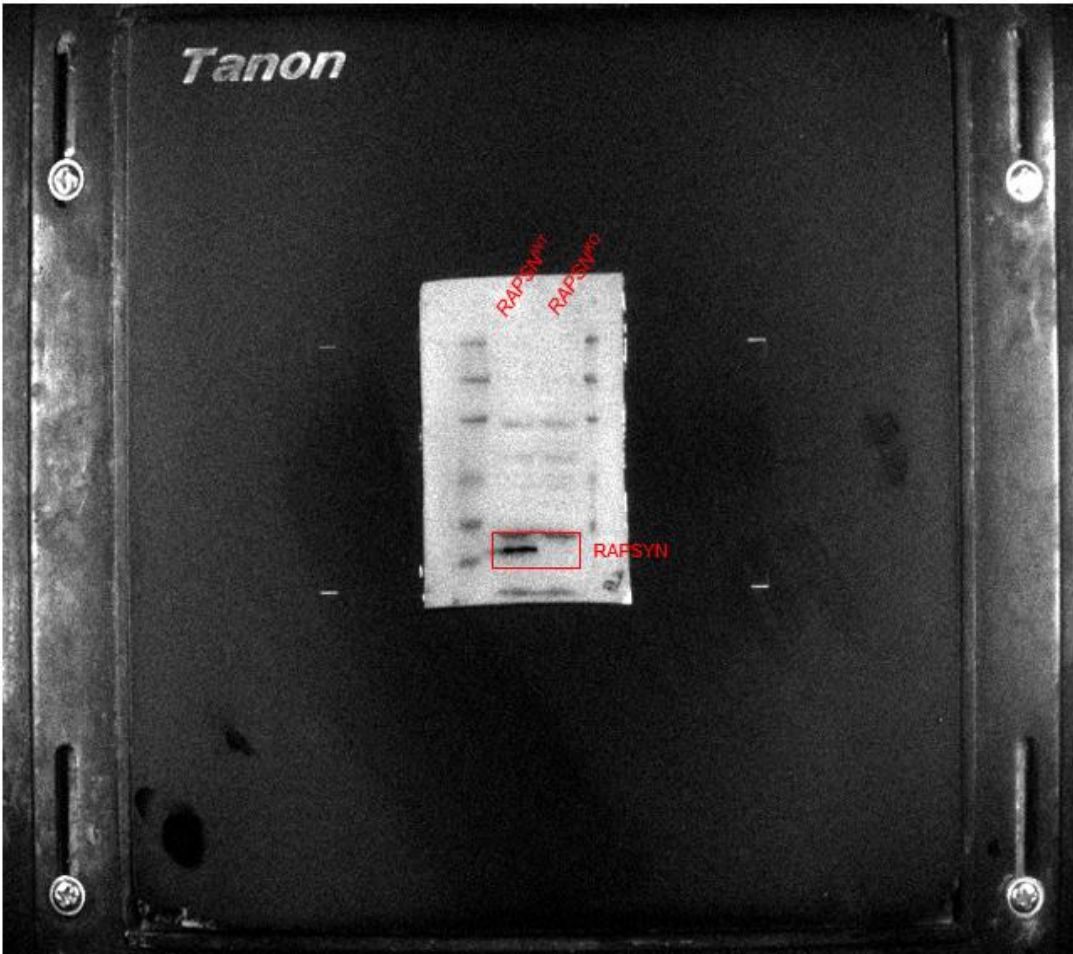

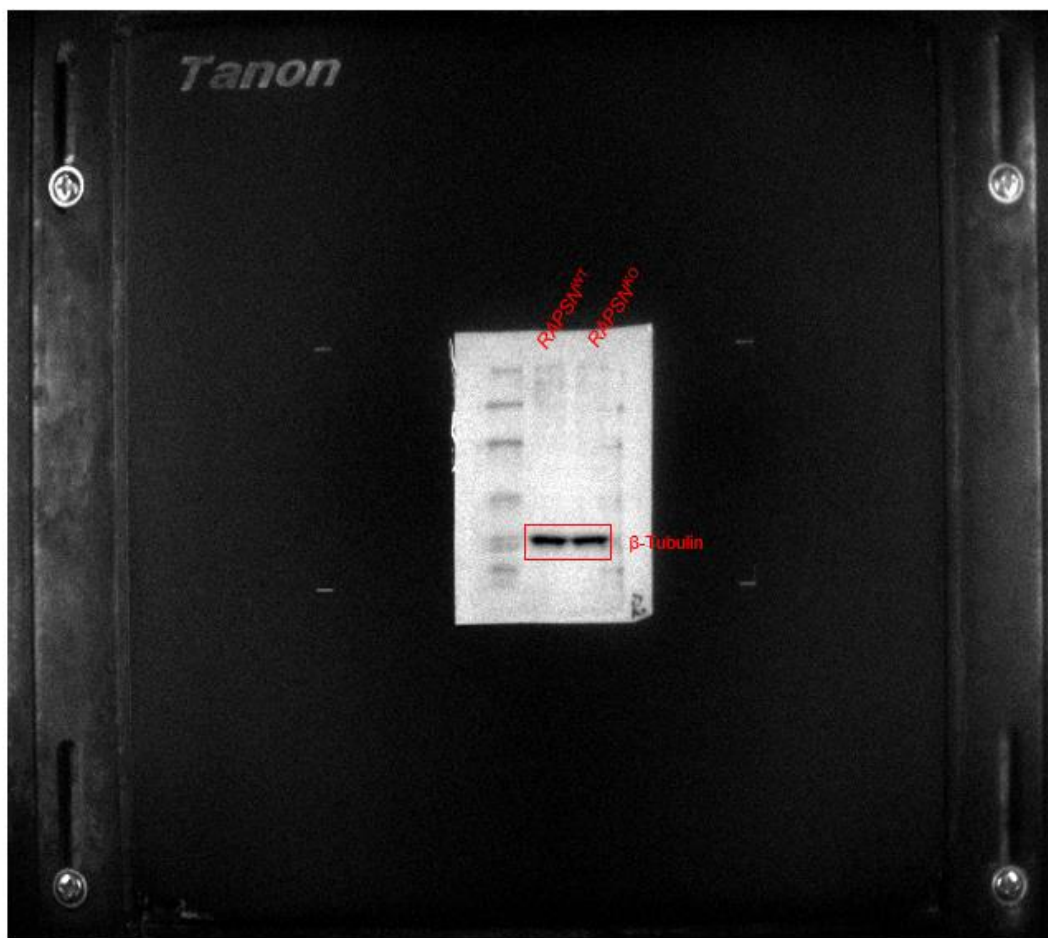

Supplement: Figure 1—source data 8. [file elife-88375-fig1-data8.zip › Figure 1-source data 8/Figure 1-source data 8.pdf]

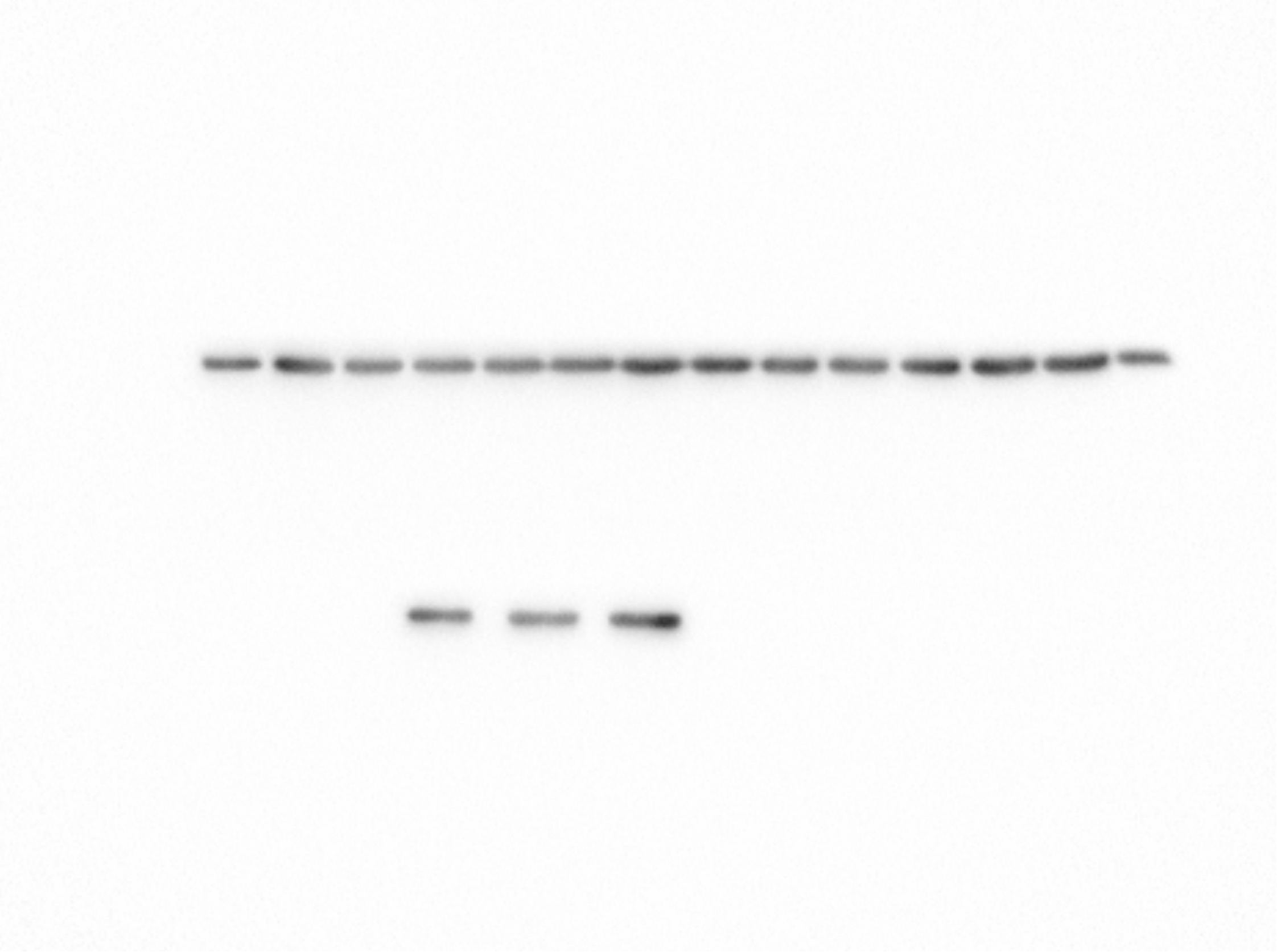

Supplement: Figure 1—figure supplement 1—source data 1. [file elife-88375-fig1-figsupp1-data1.zip › Figure supplement 1-source data 1/GAPDH.jpg]

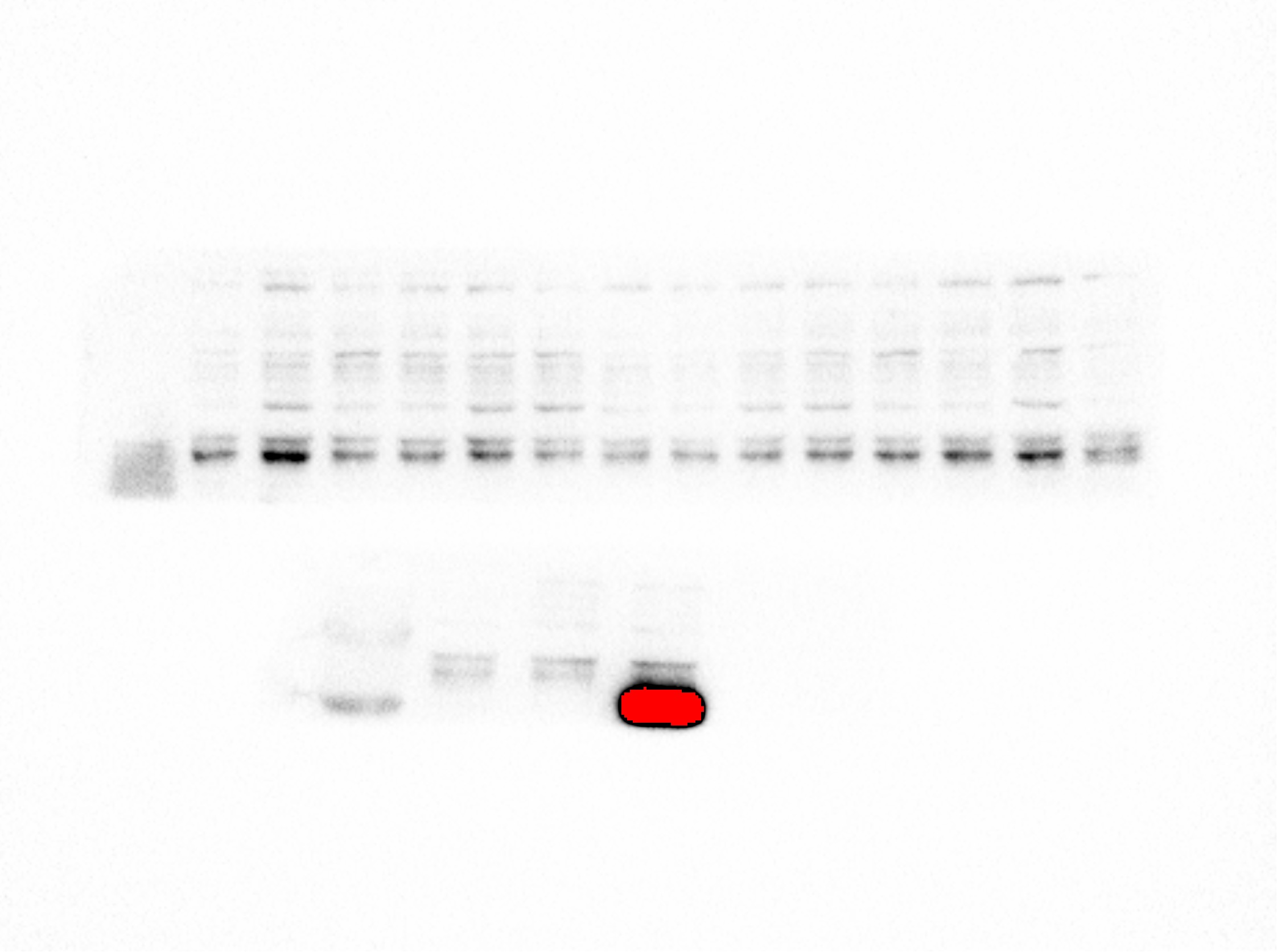

Supplement: Figure 1—figure supplement 1—source data 1. [file elife-88375-fig1-figsupp1-data1.zip › Figure supplement 1-source data 1/RAPSYN.jpg]

E

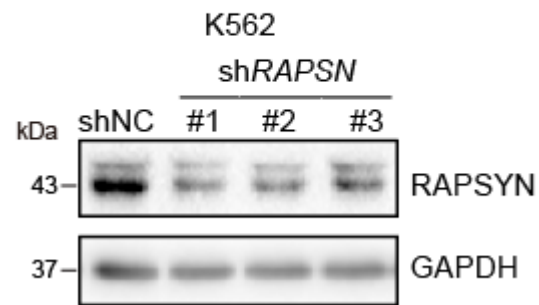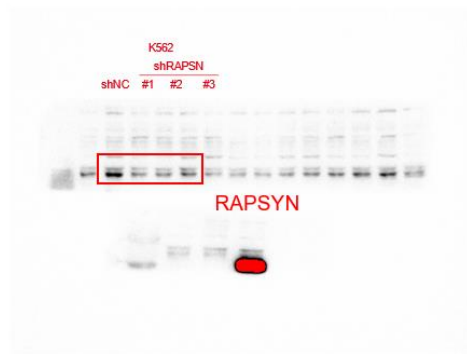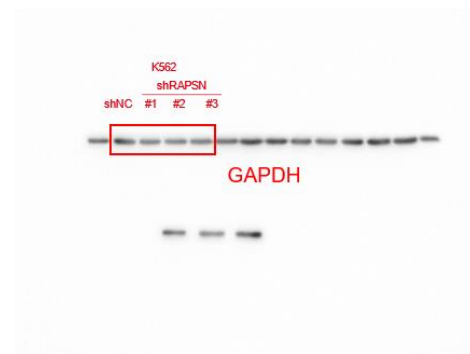

Supplement: Figure 1—figure supplement 1—source data 2. [file elife-88375-fig1-figsupp1-data2.zip › Figure supplement 1-source data 2/Figure supplement 1-source data 2.pdf]

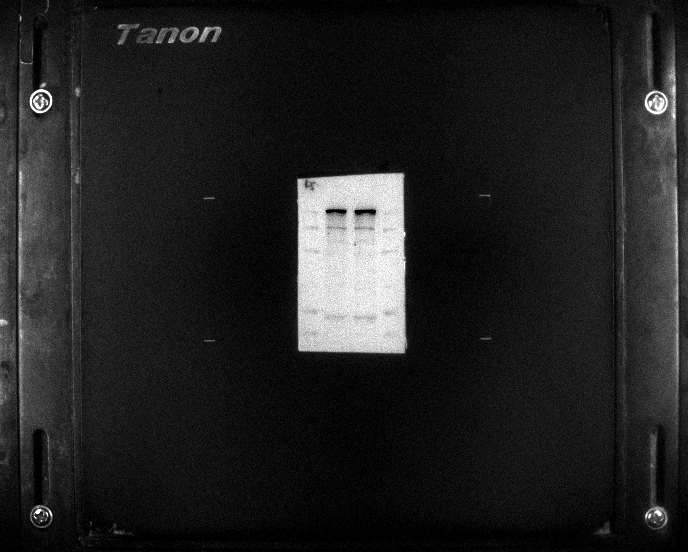

Supplement: Figure 2—source data 1. [file elife-88375-fig2-data1.zip › Figure 2-source data 1/K562 Input BCR-ABL.tif]

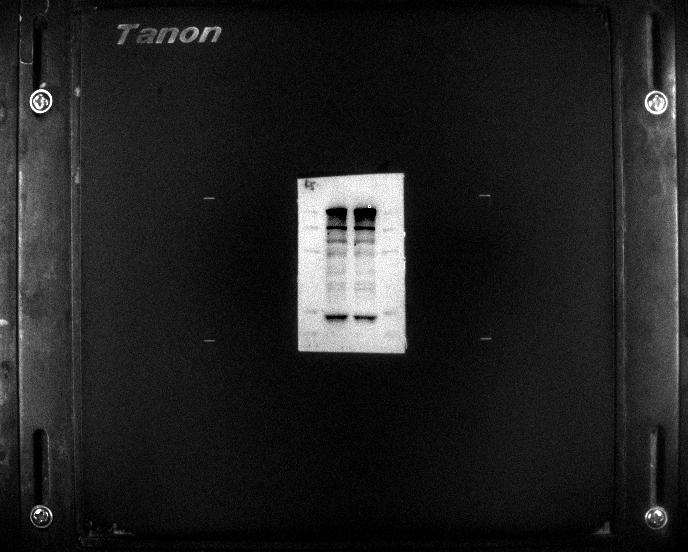

Supplement: Figure 2—source data 1. [file elife-88375-fig2-data1.zip › Figure 2-source data 1/K562 Input RAPSYN.tif]

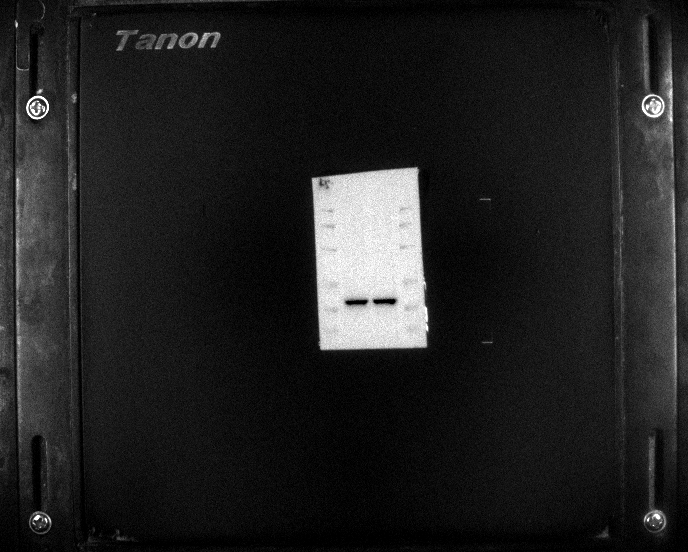

Supplement: Figure 2—source data 1. [file elife-88375-fig2-data1.zip › Figure 2-source data 1/K562 Input a┬-Tubulin.tif]

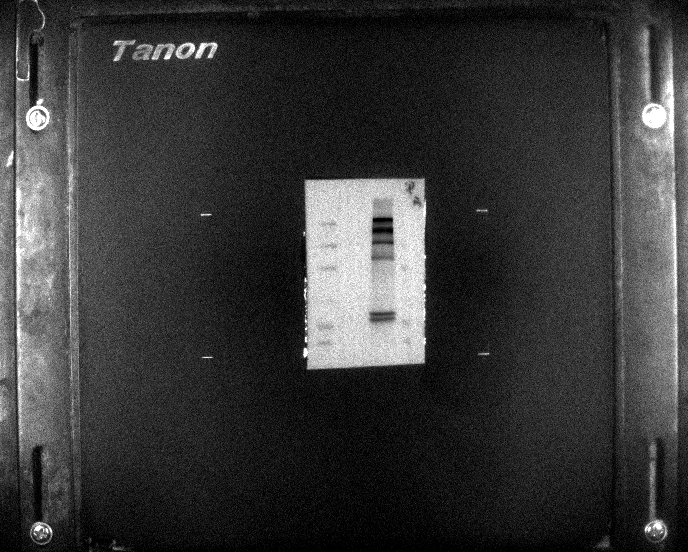

Supplement: Figure 2—source data 1. [file elife-88375-fig2-data1.zip › Figure 2-source data 1/K562 IP BCR-ABL-IB BCR-ABL.tif]

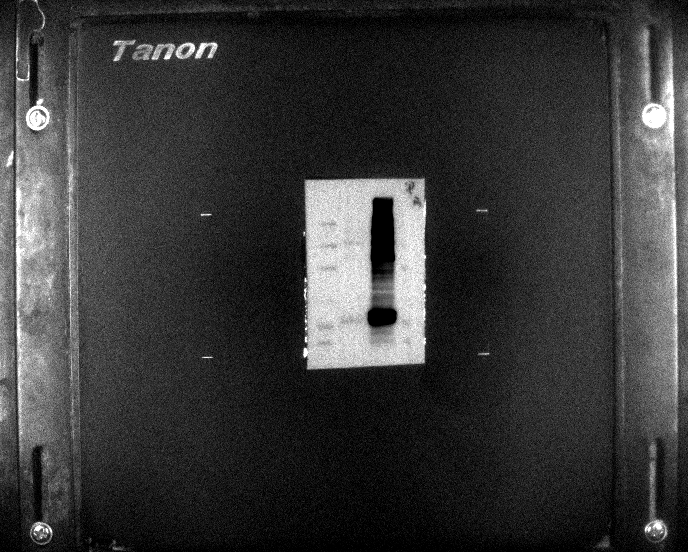

Supplement: Figure 2—source data 1. [file elife-88375-fig2-data1.zip › Figure 2-source data 1/K562 IP BCR-ABL-IB RAPSYN.tif]

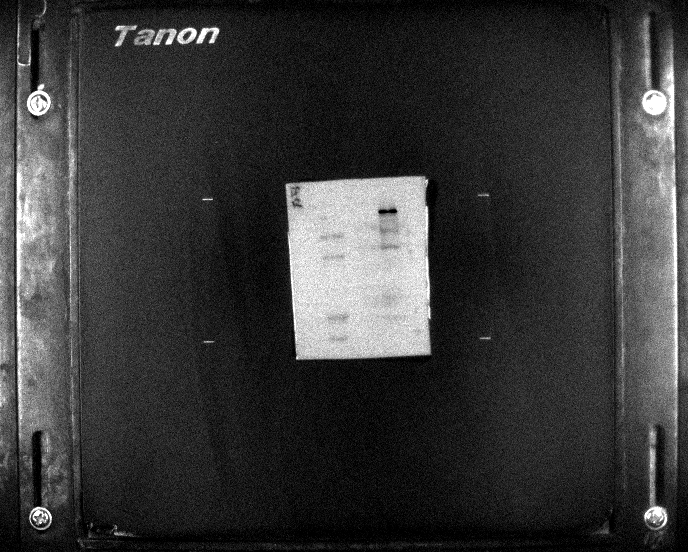

Supplement: Figure 2—source data 1. [file elife-88375-fig2-data1.zip › Figure 2-source data 1/K562 IP RAPSYN-IB BCR-ABL.tif]

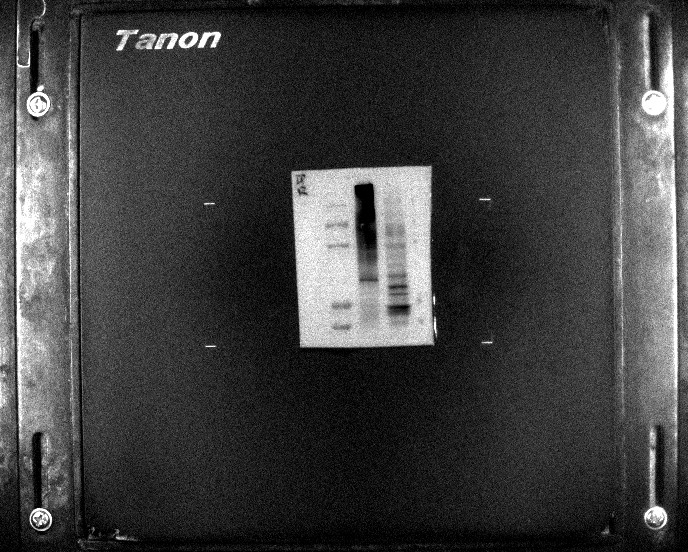

Supplement: Figure 2—source data 1. [file elife-88375-fig2-data1.zip › Figure 2-source data 1/K562 IP Rapsyn-IB RAPSYN.tif]

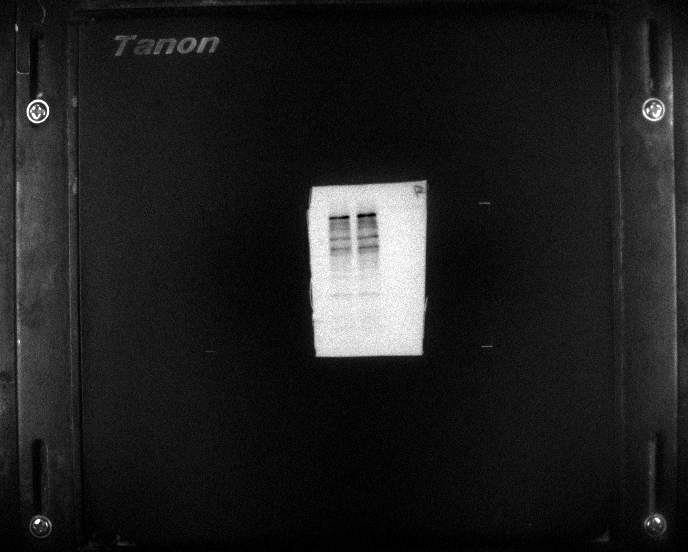

Supplement: Figure 2—source data 1. [file elife-88375-fig2-data1.zip › Figure 2-source data 1/MEG-01 Inut BCR-ABL.tif]

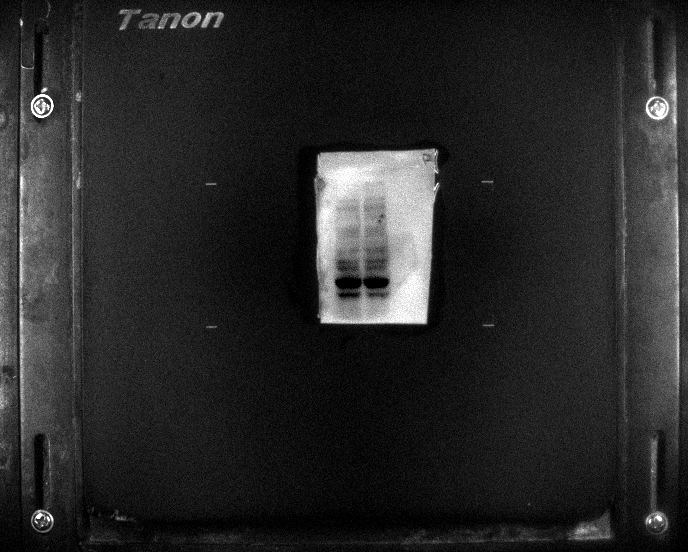

Supplement: Figure 2—source data 1. [file elife-88375-fig2-data1.zip › Figure 2-source data 1/MEG-01 Inut RAPSYN.tif]

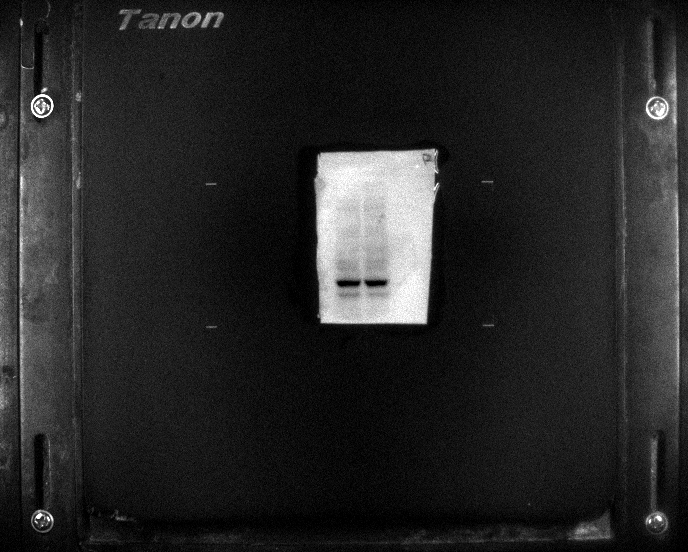

Supplement: Figure 2—source data 1. [file elife-88375-fig2-data1.zip › Figure 2-source data 1/MEG-01 Inut a┬-Tubulin.tif]

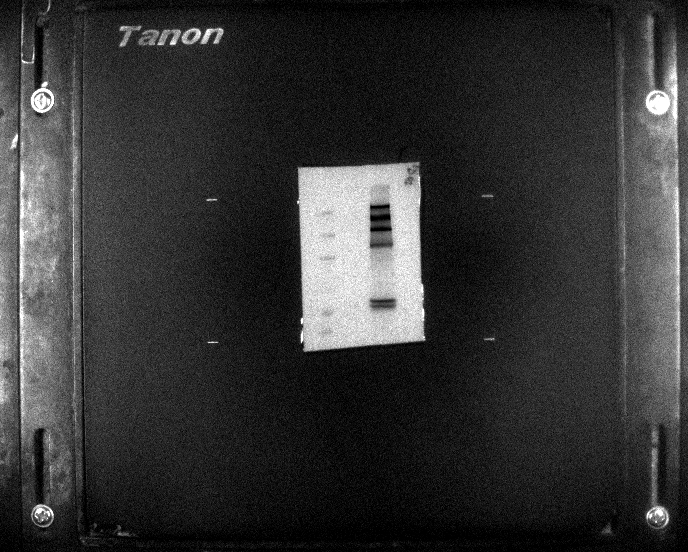

Supplement: Figure 2—source data 1. [file elife-88375-fig2-data1.zip › Figure 2-source data 1/MEG-01 IP BCR-ABL-IB BCR-ABL.tif]

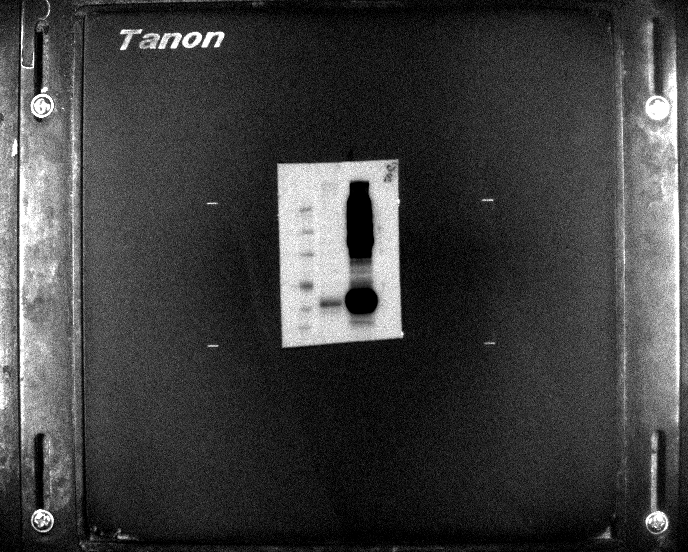

Supplement: Figure 2—source data 1. [file elife-88375-fig2-data1.zip › Figure 2-source data 1/MEG-01 IP BCR-ABL-IB RAPSYN.tif]

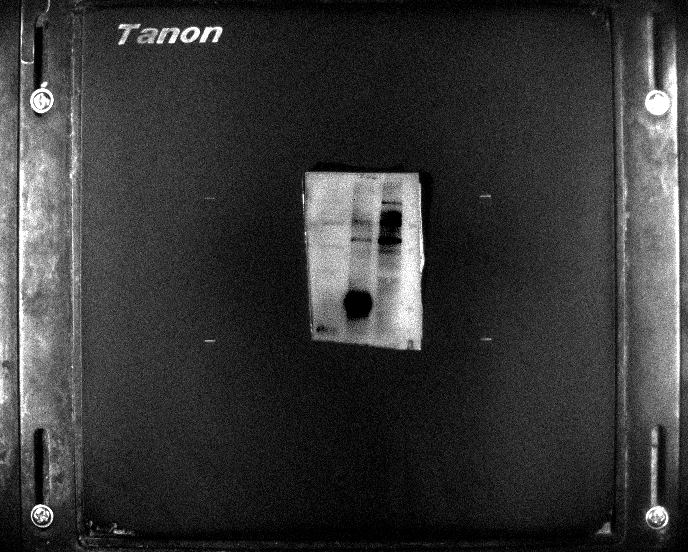

Supplement: Figure 2—source data 1. [file elife-88375-fig2-data1.zip › Figure 2-source data 1/MEG-01 IP RAPSYN-IB BCR-ABL.tif]

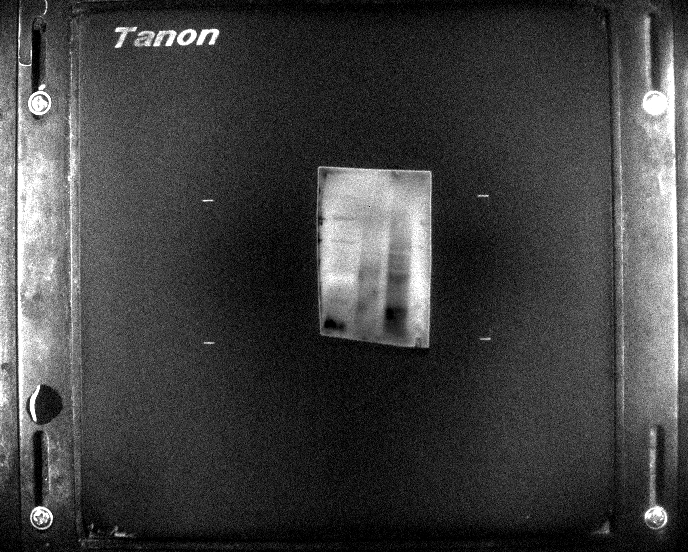

Supplement: Figure 2—source data 1. [file elife-88375-fig2-data1.zip › Figure 2-source data 1/MEG-01 IP RAPSYN-IB RAPSYN.tif]

A

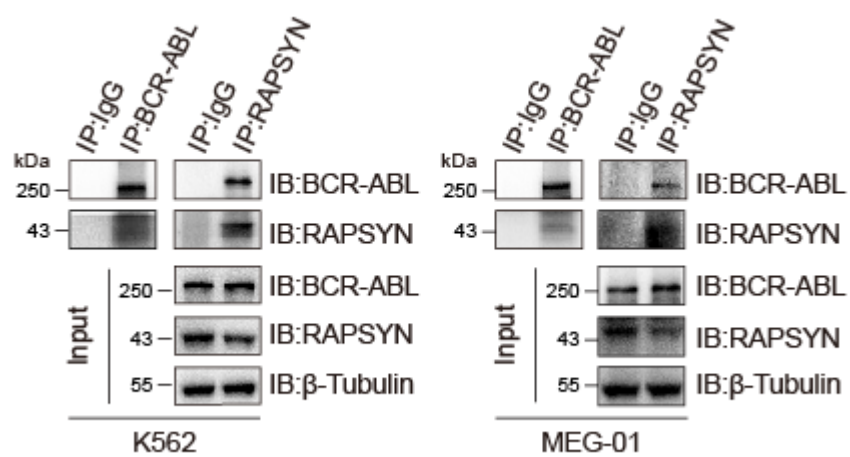

K562

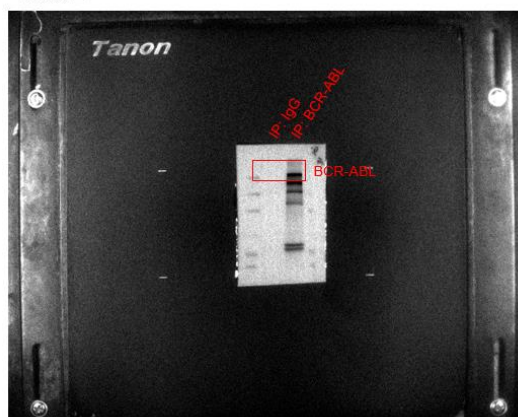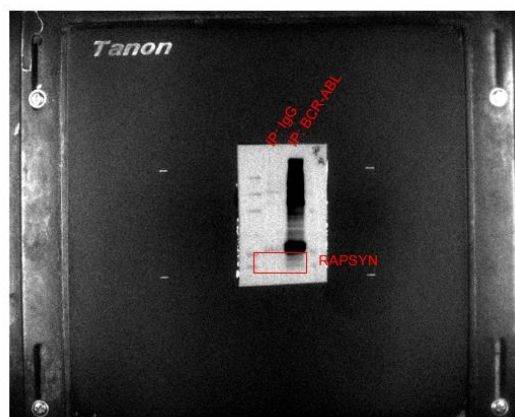

K562

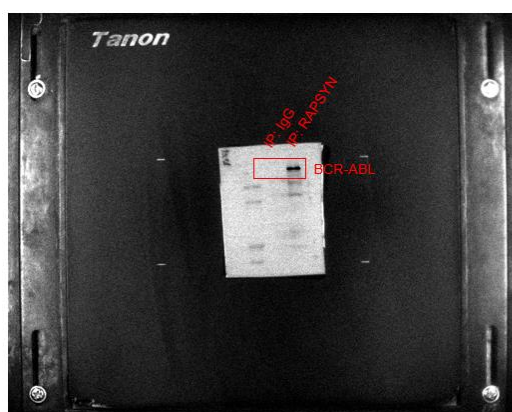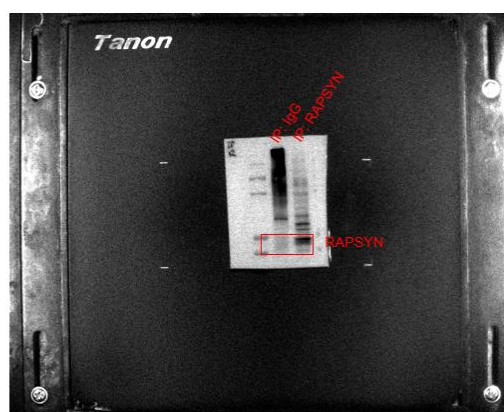

## K562 Input

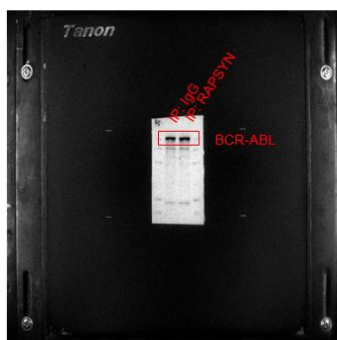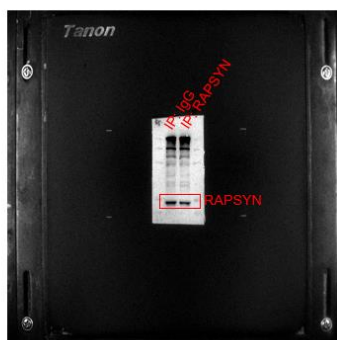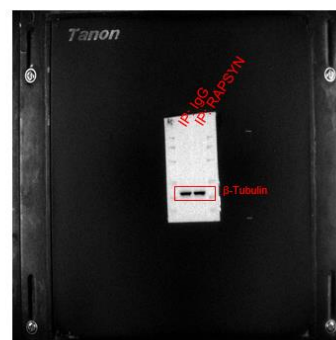

## MEG-01

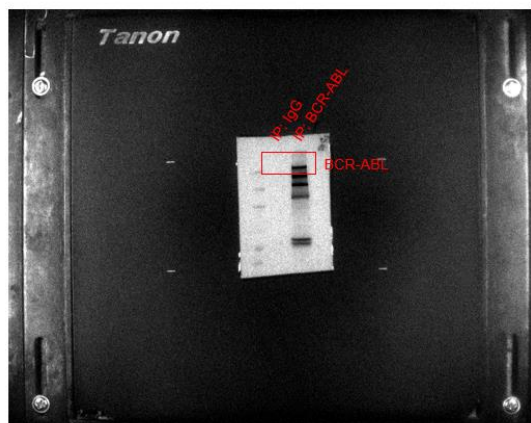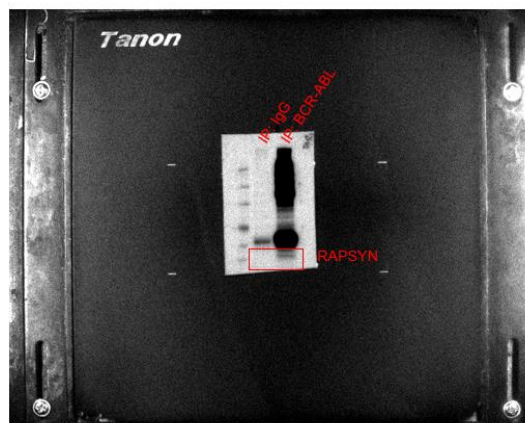

## MEG-01

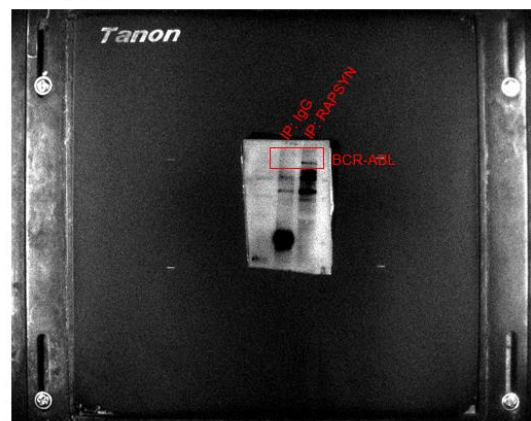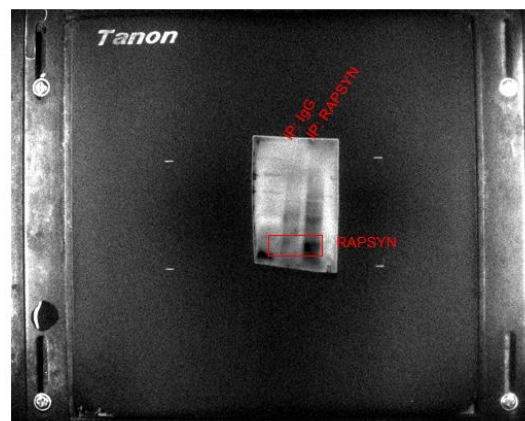

## MEG-01 Input

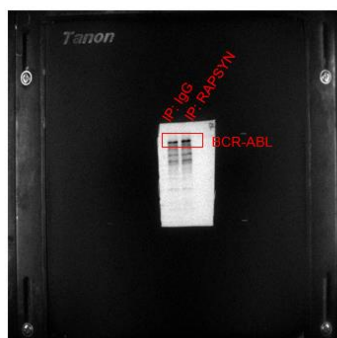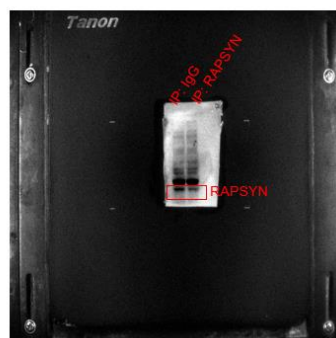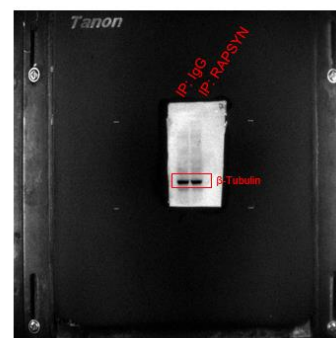

Supplement: Figure 2—source data 2. [file elife-88375-fig2-data2.zip › Figure 2-source data 2/Figure 2-source data 2.pdf]

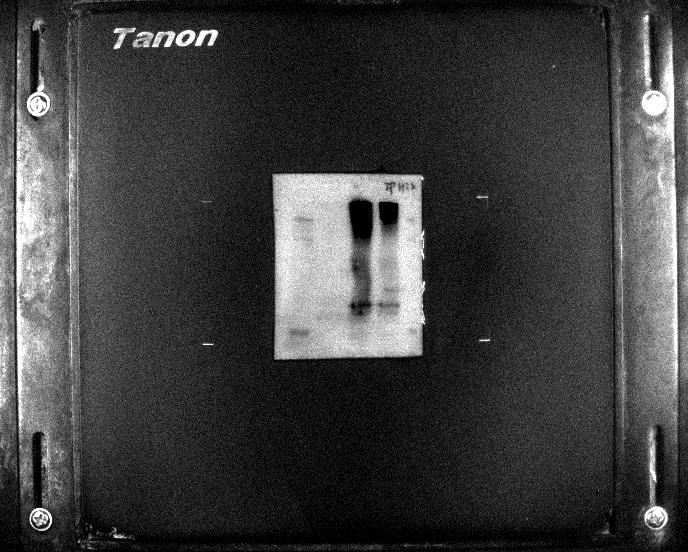

Supplement: Figure 2—source data 3. [file elife-88375-fig2-data3.zip › Figure 2-source data 3/IP GST-IB GST.tif]

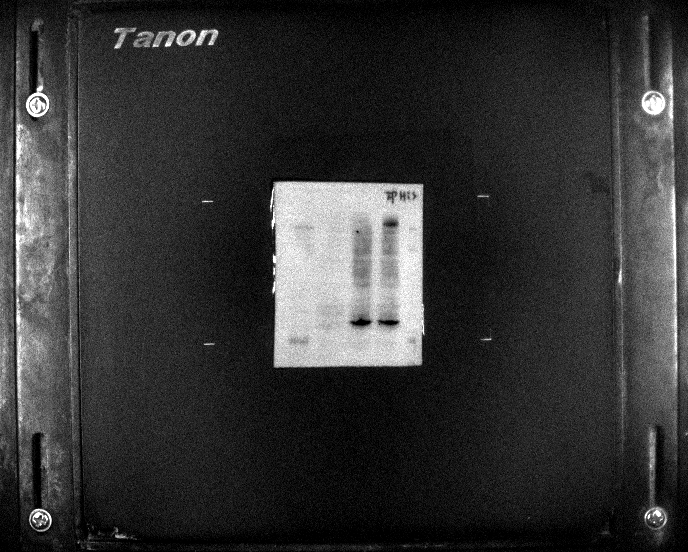

Supplement: Figure 2—source data 3. [file elife-88375-fig2-data3.zip › Figure 2-source data 3/IP GST-IB His.tif]

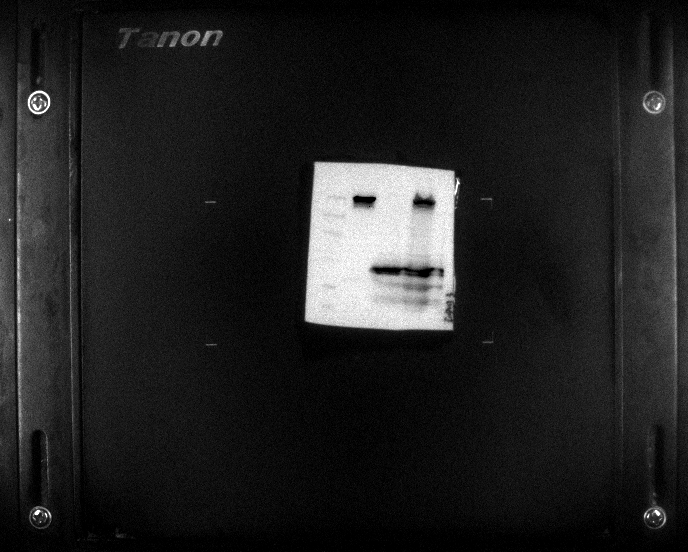

Supplement: Figure 2—source data 3. [file elife-88375-fig2-data3.zip › Figure 2-source data 3/IP GST-Input GST.tif]

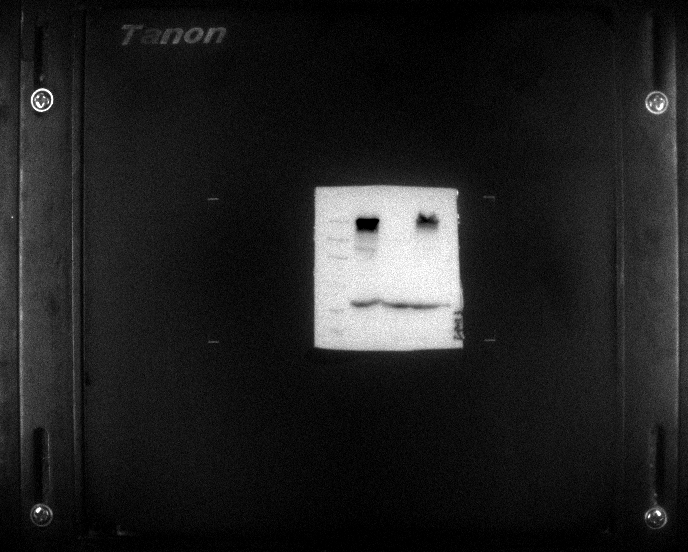

Supplement: Figure 2—source data 3. [file elife-88375-fig2-data3.zip › Figure 2-source data 3/IP GST-Input His.tif]

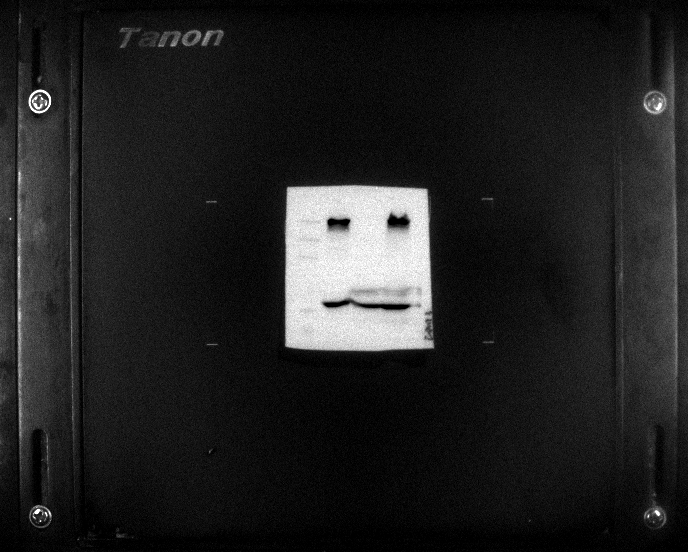

Supplement: Figure 2—source data 3. [file elife-88375-fig2-data3.zip › Figure 2-source data 3/IP GST-Input a┬-Tubulin.tif]

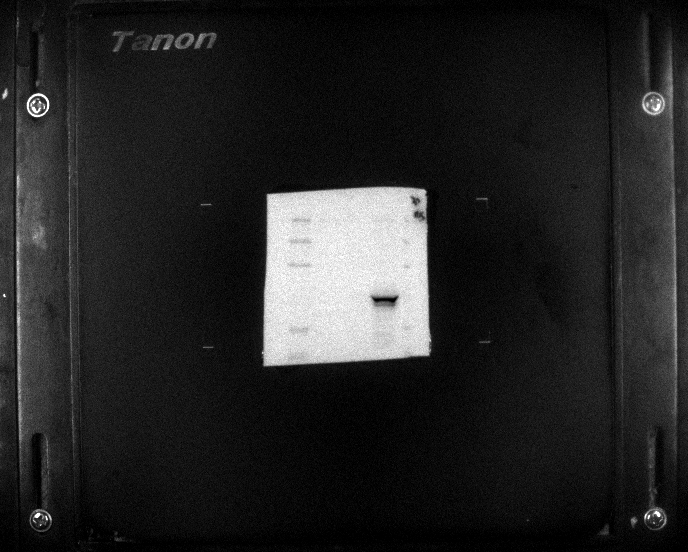

Supplement: Figure 2—source data 3. [file elife-88375-fig2-data3.zip › Figure 2-source data 3/IP His-IB GST.tif]

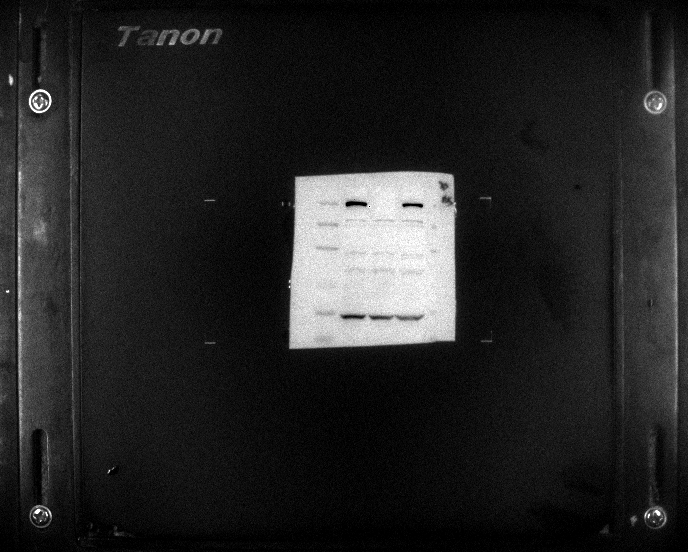

Supplement: Figure 2—source data 3. [file elife-88375-fig2-data3.zip › Figure 2-source data 3/IP His-IB His.tif]

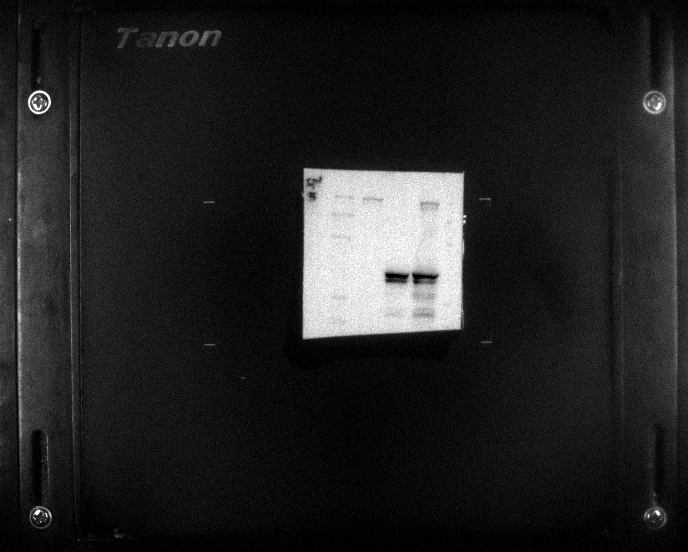

Supplement: Figure 2—source data 3. [file elife-88375-fig2-data3.zip › Figure 2-source data 3/IP His-Inpurt GST.tif]

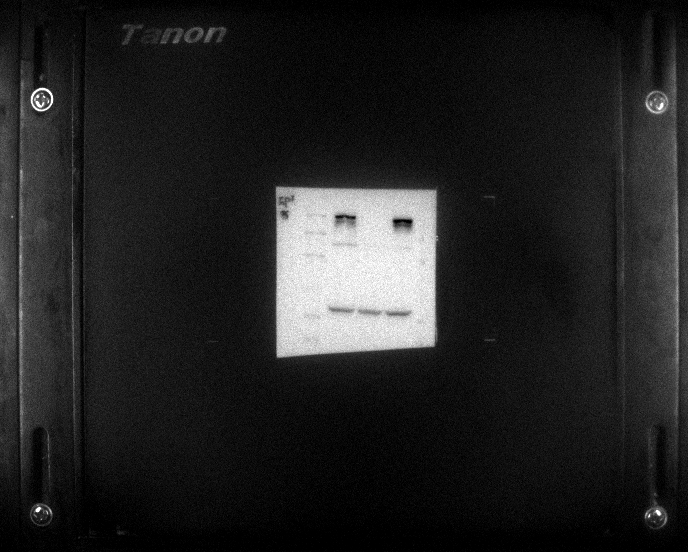

Supplement: Figure 2—source data 3. [file elife-88375-fig2-data3.zip › Figure 2-source data 3/IP His-Input His.tif]

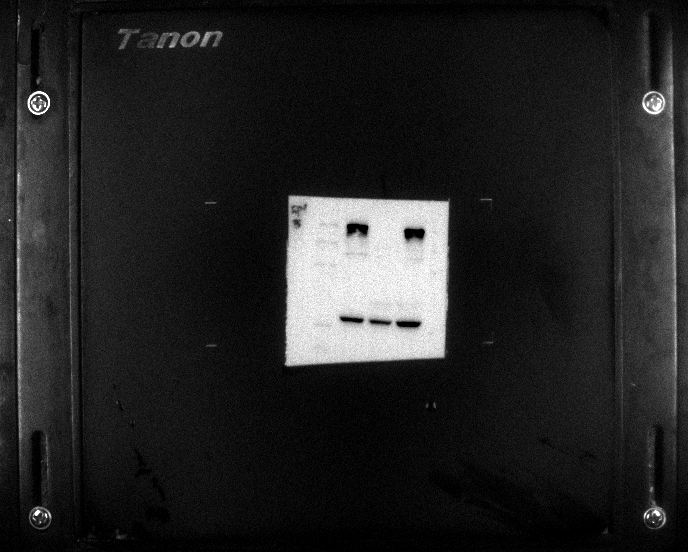

Supplement: Figure 2—source data 3. [file elife-88375-fig2-data3.zip › Figure 2-source data 3/IP His-Input a┬-Tubulin.tif]

B

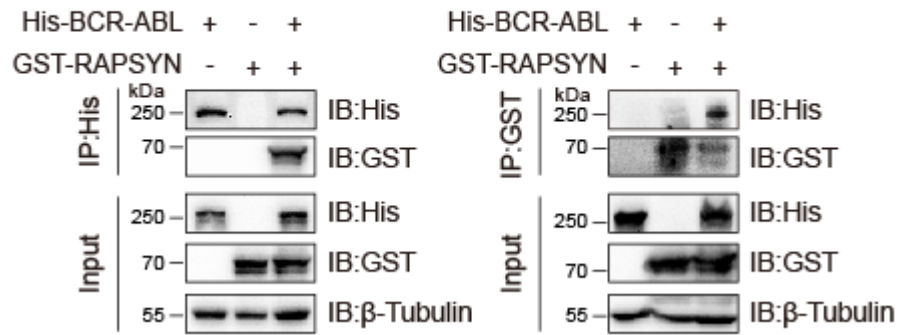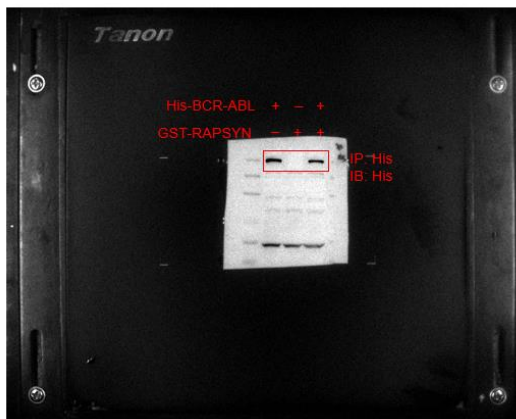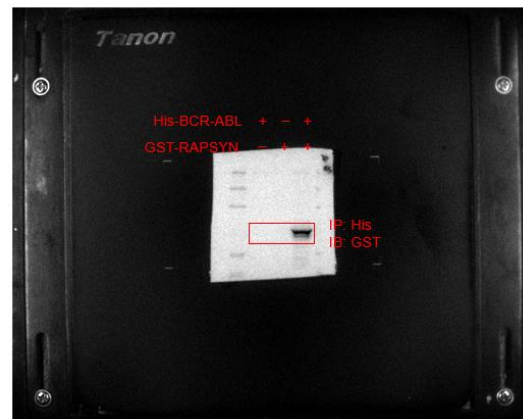

IP: His - Input

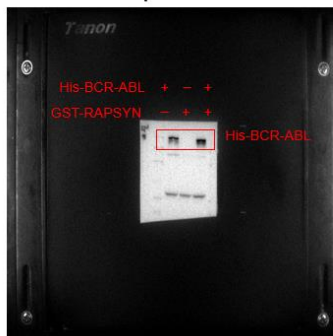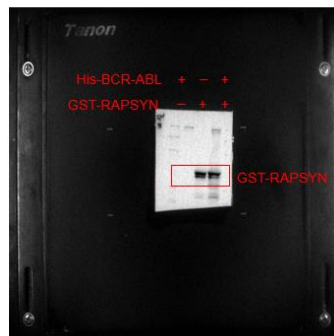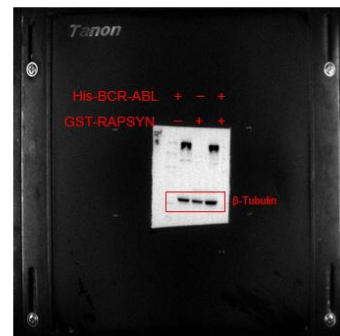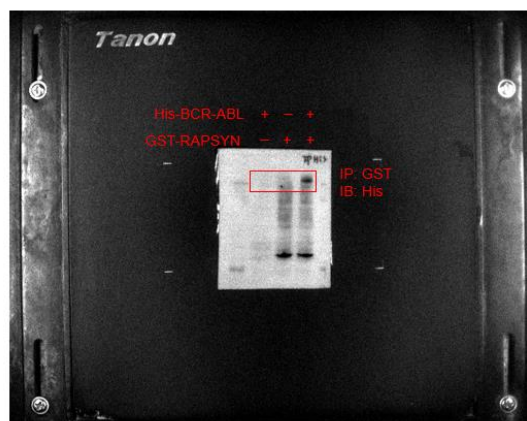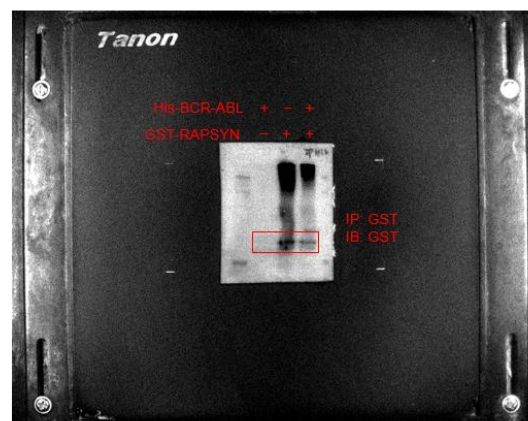

# IP: GST - Input

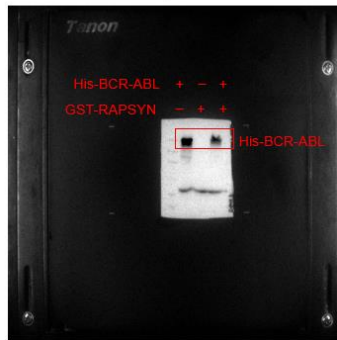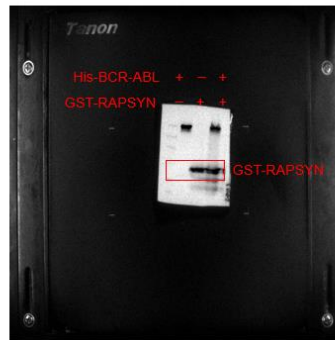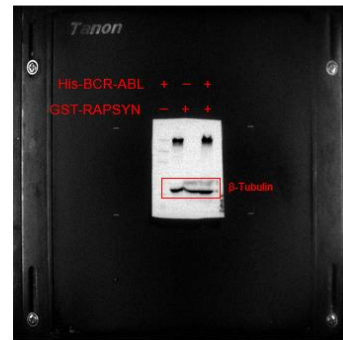

Supplement: Figure 2—source data 4. [file elife-88375-fig2-data4.zip › Figure 2-source data 4/Figure 2-source data 4.pdf]

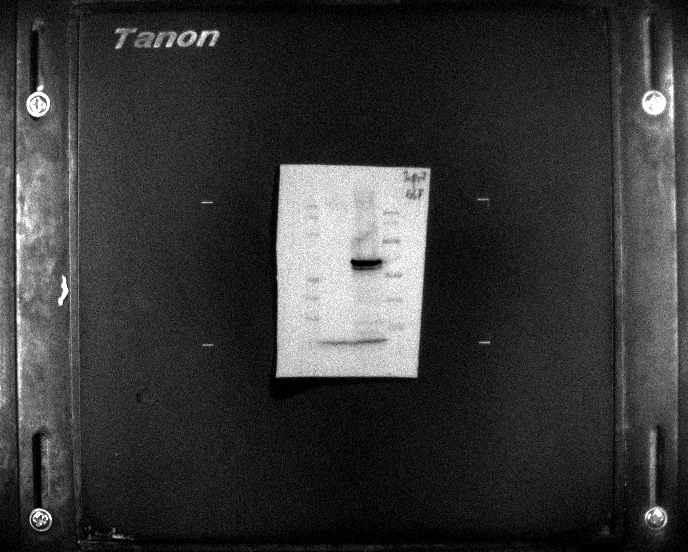

Supplement: Figure 2—source data 5. [file elife-88375-fig2-data5.zip › Figure 2-source data 5/Input GST.tif]

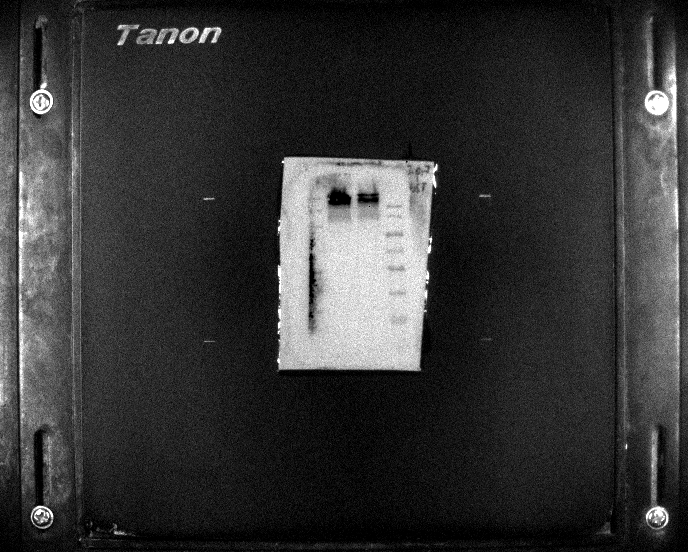

Supplement: Figure 2—source data 5. [file elife-88375-fig2-data5.zip › Figure 2-source data 5/Input His.tif]

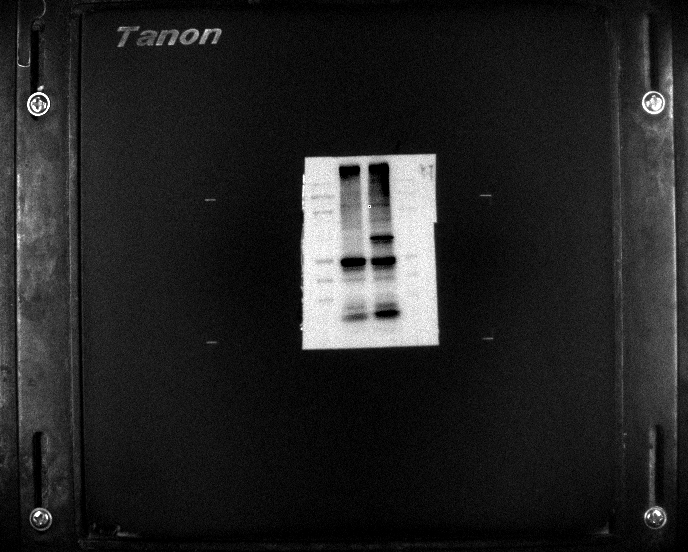

Supplement: Figure 2—source data 5. [file elife-88375-fig2-data5.zip › Figure 2-source data 5/IP GST.tif]

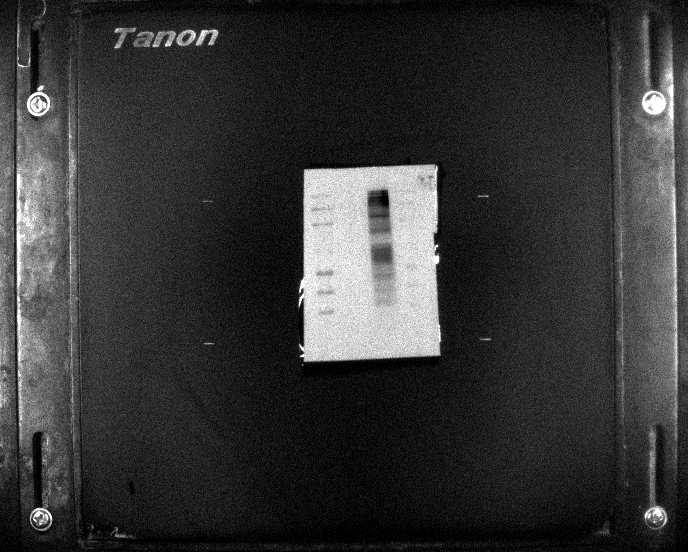

Supplement: Figure 2—source data 5. [file elife-88375-fig2-data5.zip › Figure 2-source data 5/IP His.tif]

C

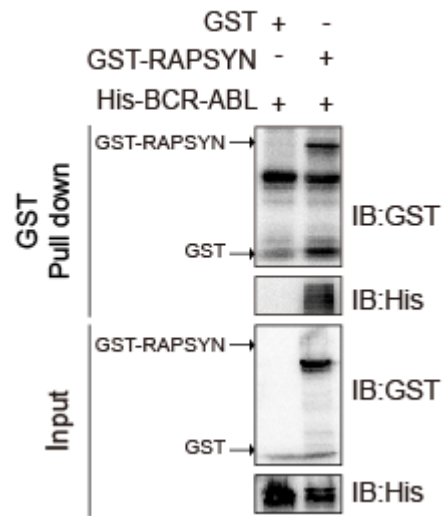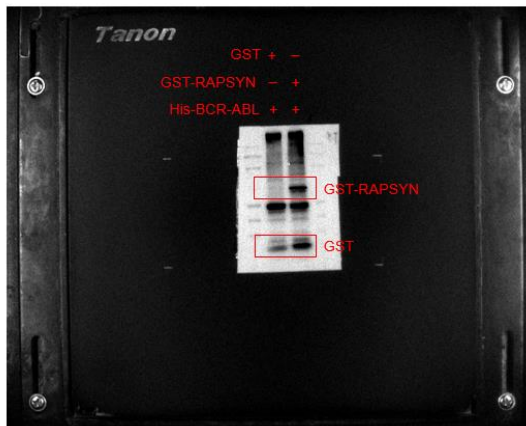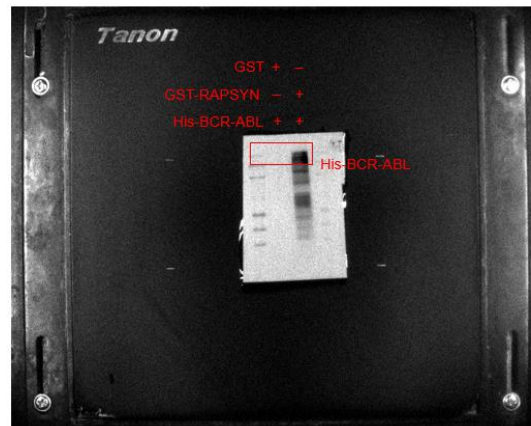

Input

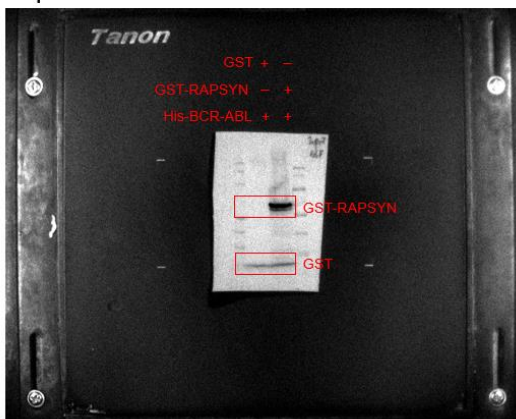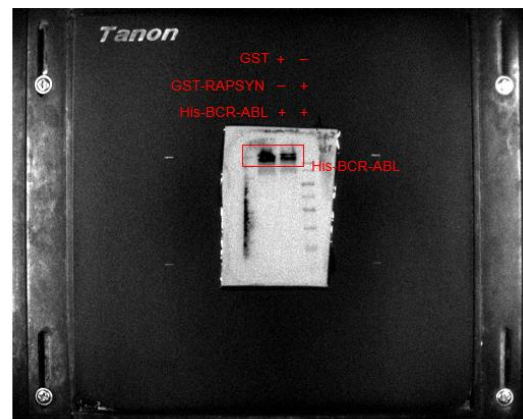

Supplement: Figure 2—source data 6. [file elife-88375-fig2-data6.zip › Figure 2-source data 6/Figure 2-source data 6.pdf]

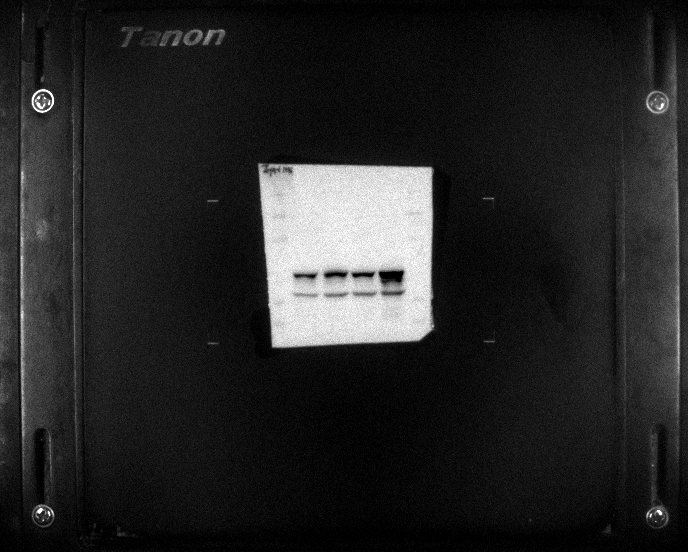

Supplement: Figure 2—source data 7. [file elife-88375-fig2-data7.zip › Figure 2-source data 7/Input GST.tif]

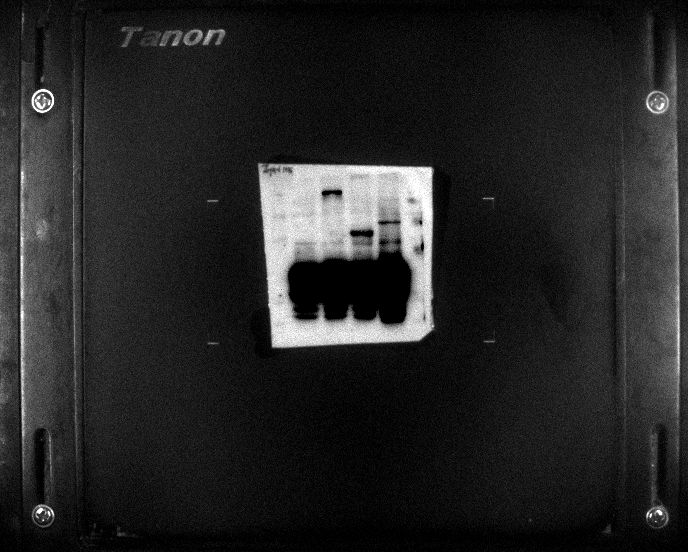

Supplement: Figure 2—source data 7. [file elife-88375-fig2-data7.zip › Figure 2-source data 7/Input His.tif]

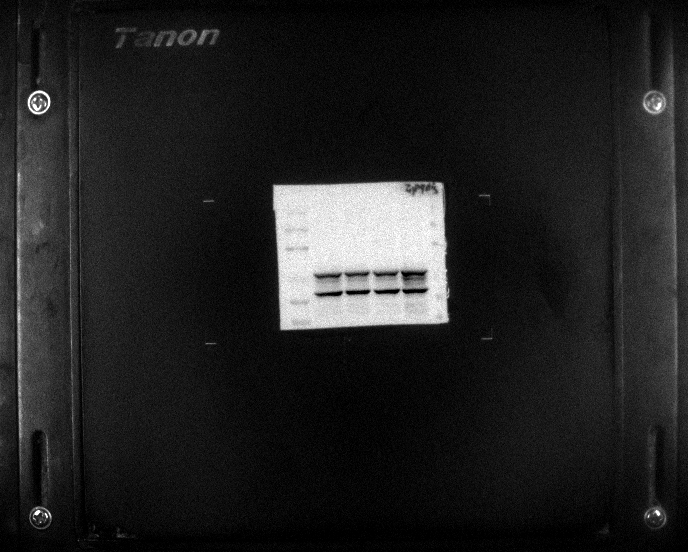

Supplement: Figure 2—source data 7. [file elife-88375-fig2-data7.zip › Figure 2-source data 7/IP GST.tif]

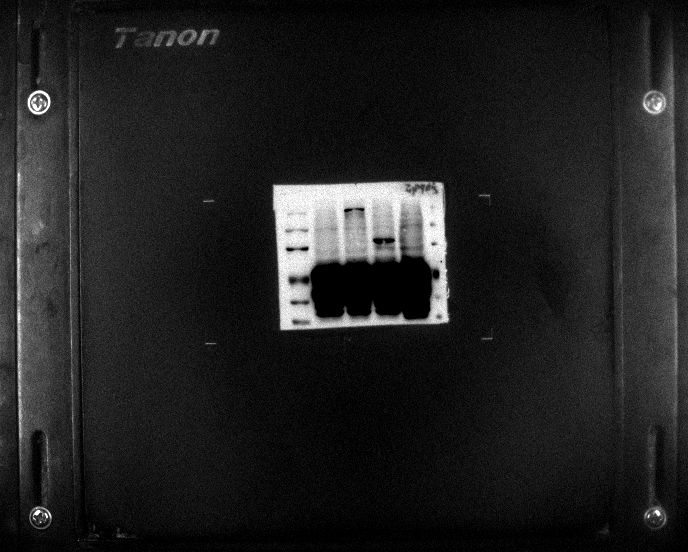

Supplement: Figure 2—source data 7. [file elife-88375-fig2-data7.zip › Figure 2-source data 7/IP HIS.tif]

D

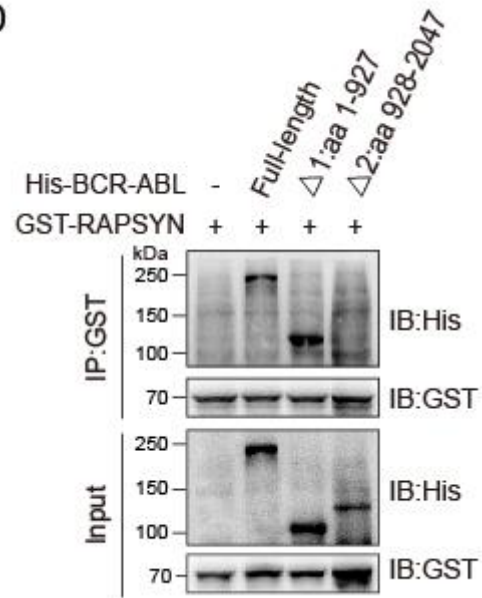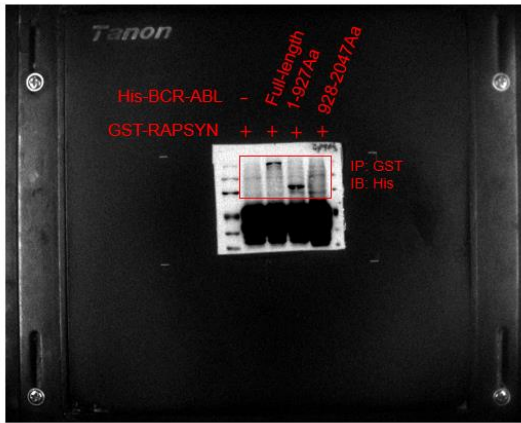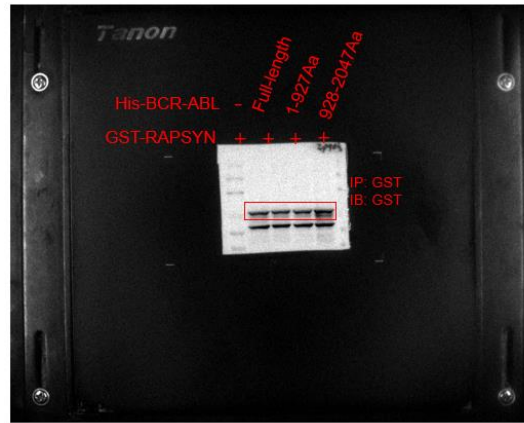

Input

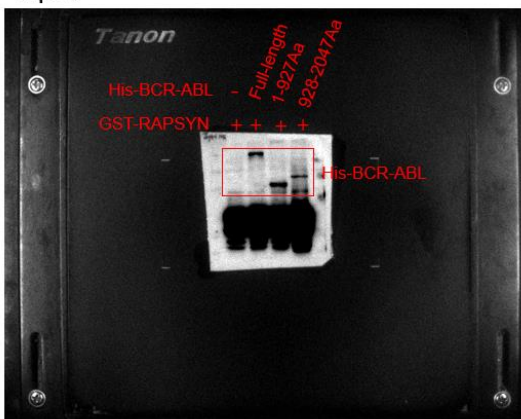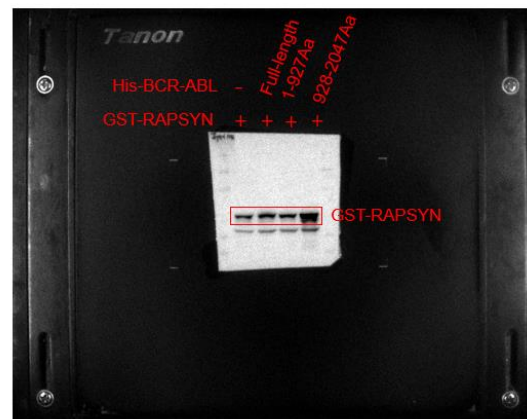

Supplement: Figure 2—source data 8. [file elife-88375-fig2-data8.zip › Figure 2-source data 8/Figure 2-source data 8.pdf]

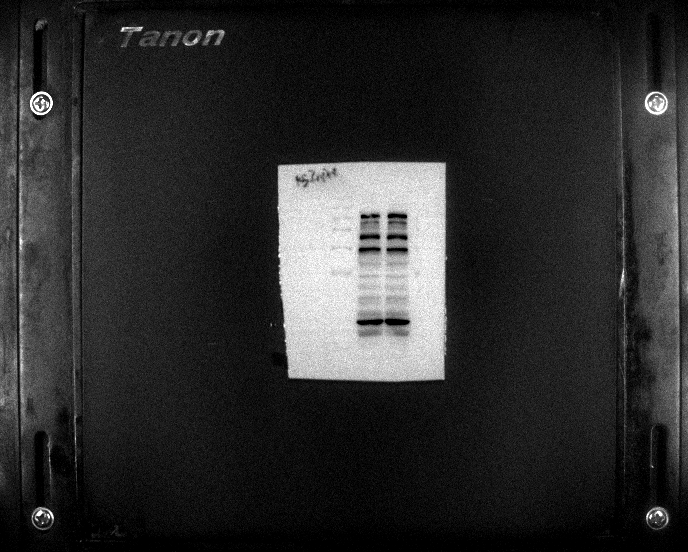

Supplement: Figure 2—source data 9. [file elife-88375-fig2-data9.zip › Figure 2-source data 9/K562 Input BCR-ABL.tif]

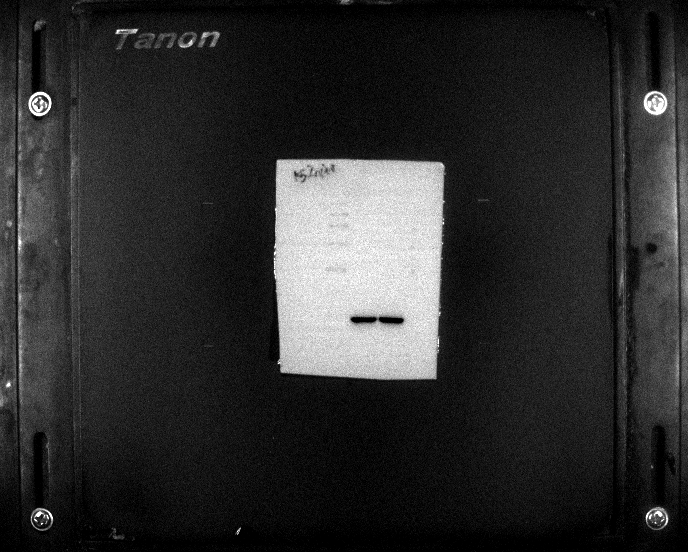

Supplement: Figure 2—source data 9. [file elife-88375-fig2-data9.zip › Figure 2-source data 9/K562 Input a┬-Tubulin.tif]

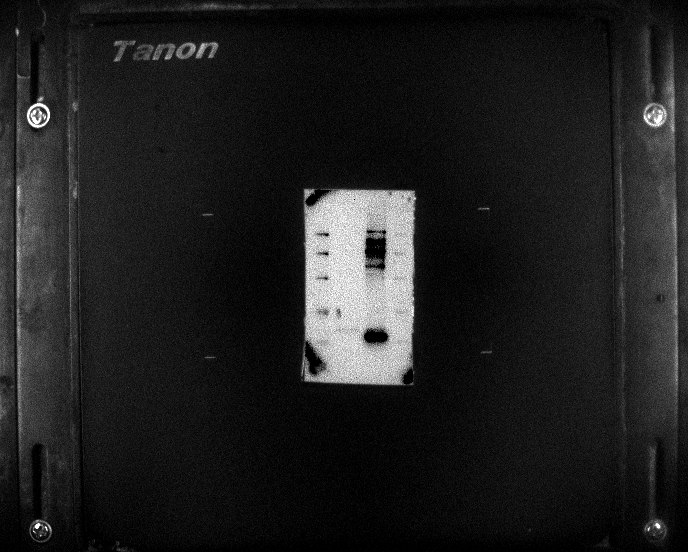

Supplement: Figure 2—source data 9. [file elife-88375-fig2-data9.zip › Figure 2-source data 9/K562 IP BCR-ABL-IB BCR-ABL.tif]

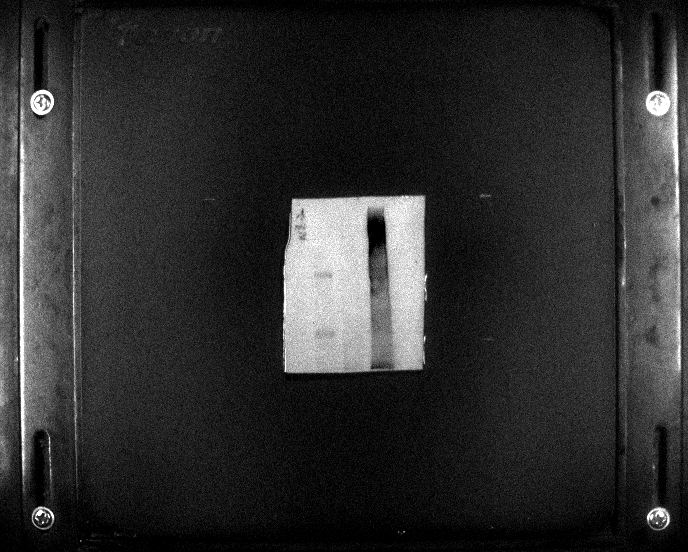

Supplement: Figure 2—source data 9. [file elife-88375-fig2-data9.zip › Figure 2-source data 9/K562 IP BCR-ABL-IB NEDD8.tif]

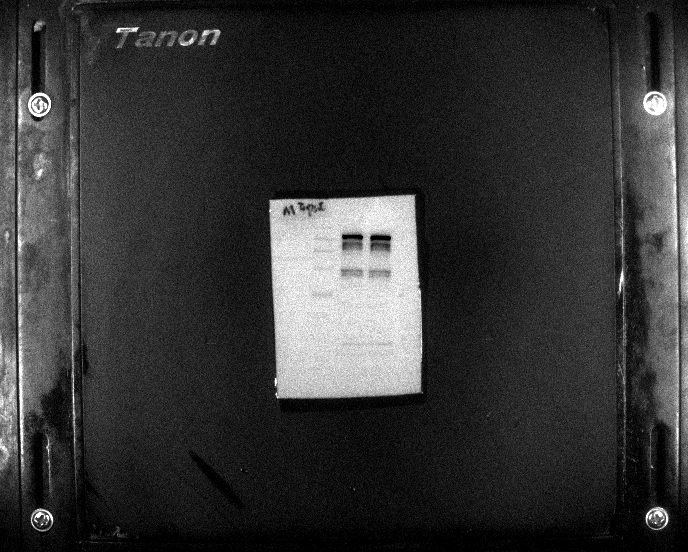

Supplement: Figure 2—source data 9. [file elife-88375-fig2-data9.zip › Figure 2-source data 9/MEG-01 Input BCR-ABL.tif]

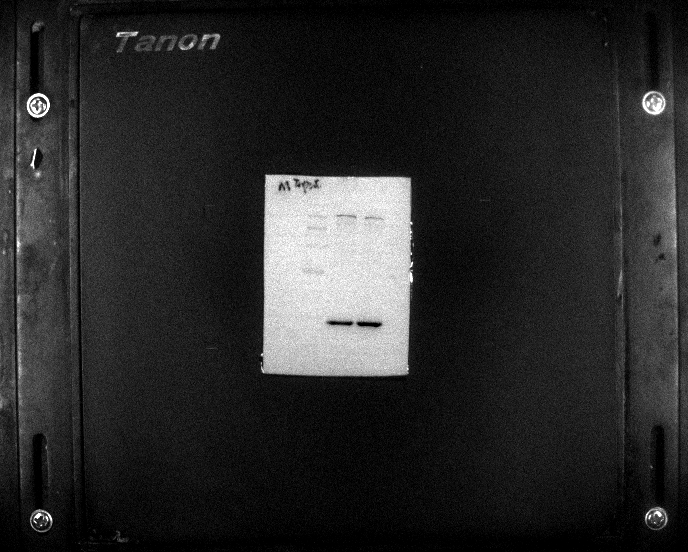

Supplement: Figure 2—source data 9. [file elife-88375-fig2-data9.zip › Figure 2-source data 9/MEG-01 Input a┬-Tubulin.tif]

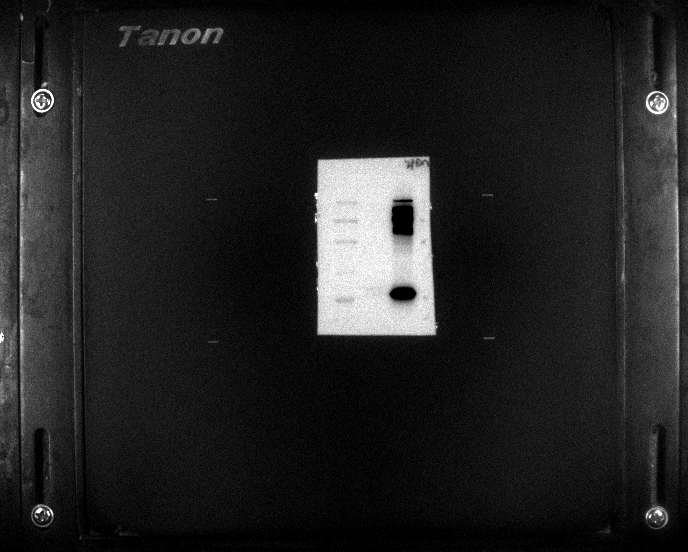

Supplement: Figure 2—source data 9. [file elife-88375-fig2-data9.zip › Figure 2-source data 9/MEG-01 IP BCR-ABL-IB BCR-ABL.tif]

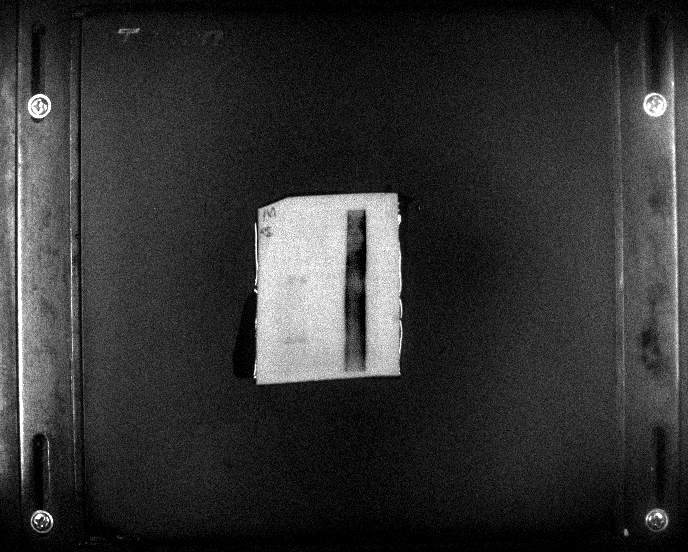

Supplement: Figure 2—source data 9. [file elife-88375-fig2-data9.zip › Figure 2-source data 9/MEG-01 IP BCR-ABL-IB NEDD8.tif]

**E**

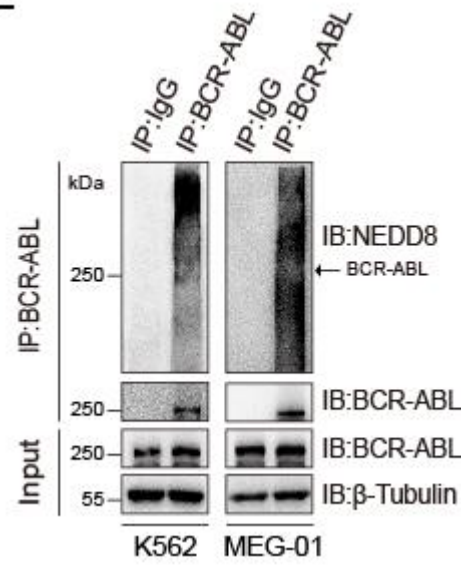

K562

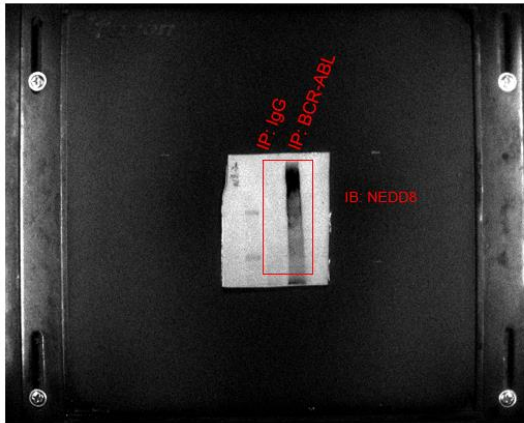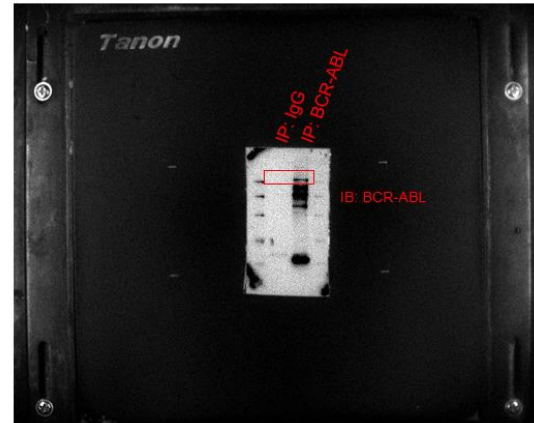

Input

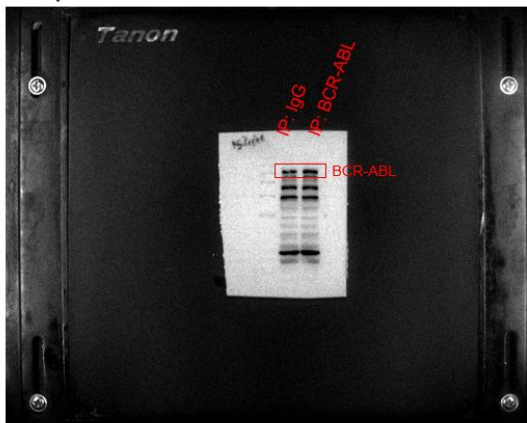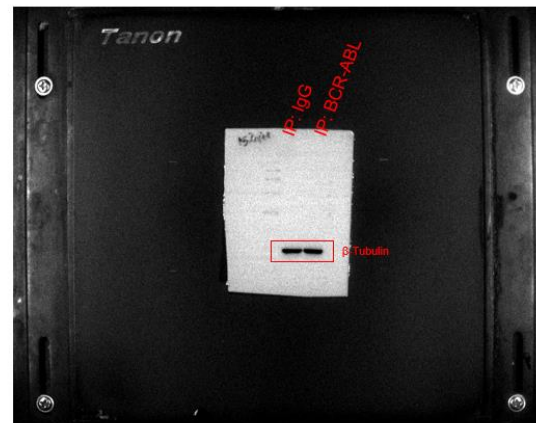

MEG-01

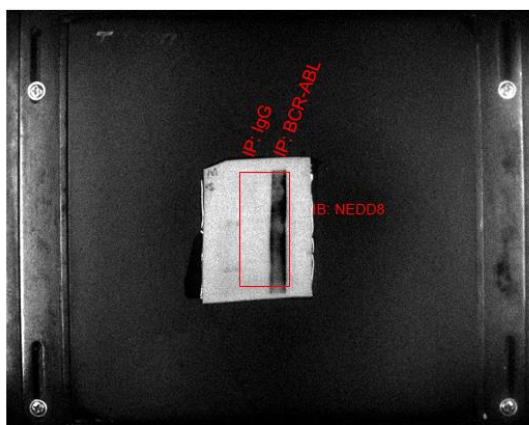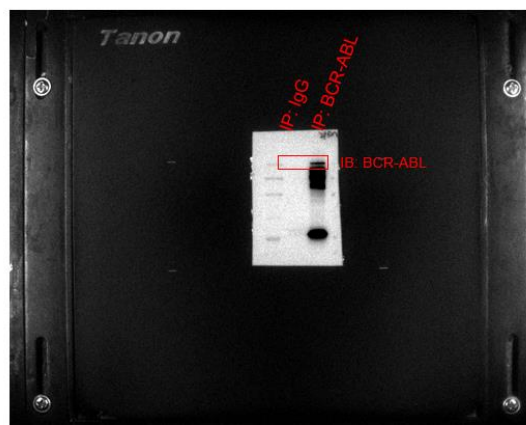

Input

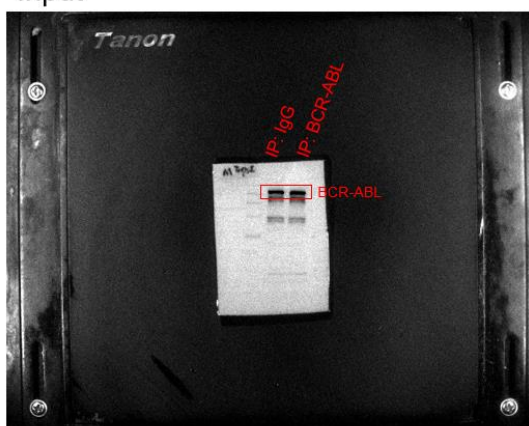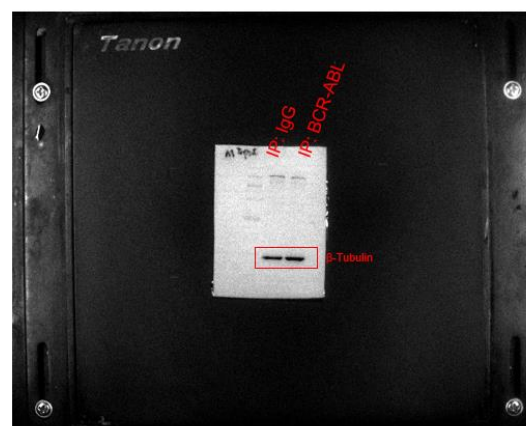

Supplement: Figure 2—source data 10. [file elife-88375-fig2-data10.zip › Figure 2-source data 10/Figure 2-source data 10.pdf]

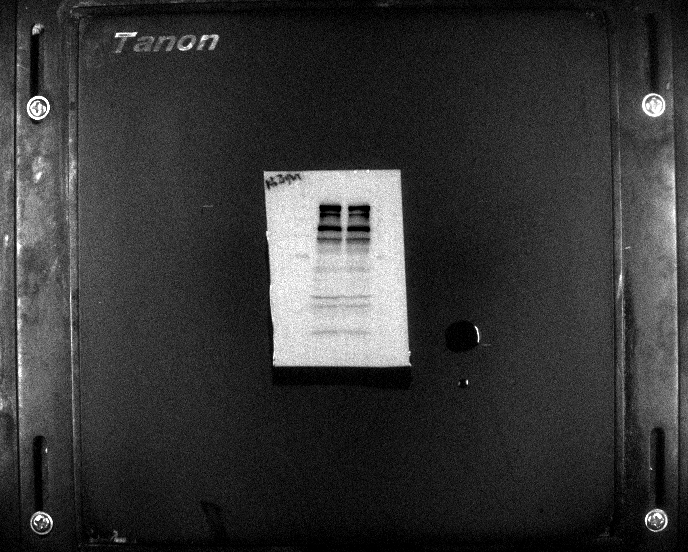

Supplement: Figure 2—source data 11. [file elife-88375-fig2-data11.zip › Figure 2-source data 11/Patient#1 Input BCR-ABL.tif]

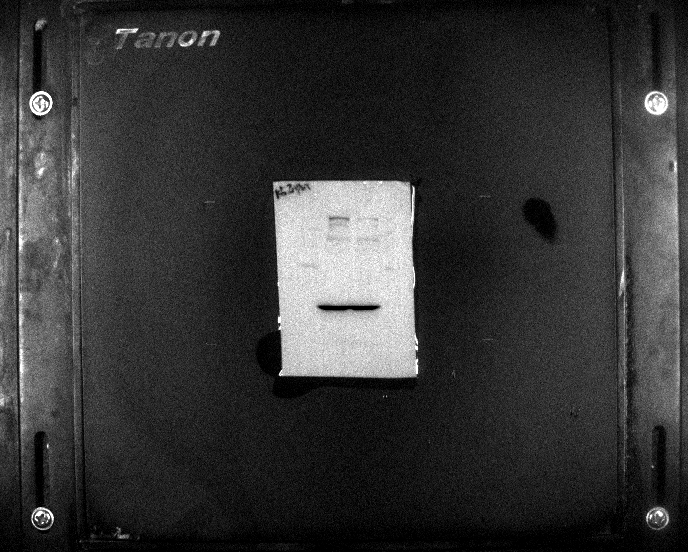

Supplement: Figure 2—source data 11. [file elife-88375-fig2-data11.zip › Figure 2-source data 11/Patient#1 Input a┬-Tubulin.tif]

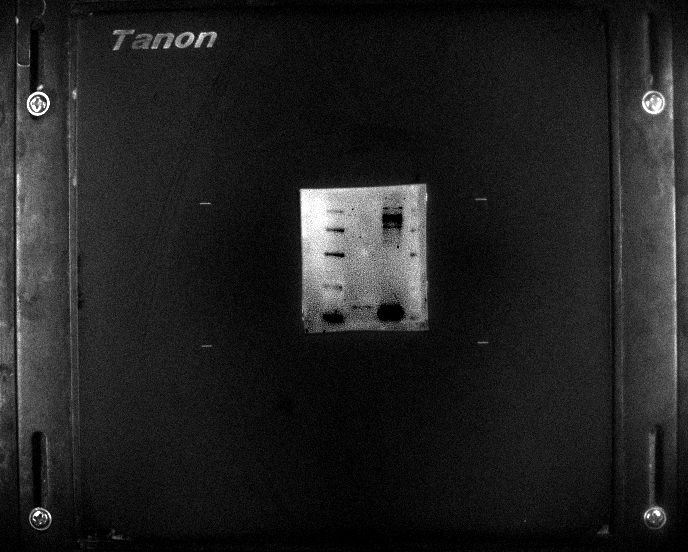

Supplement: Figure 2—source data 11. [file elife-88375-fig2-data11.zip › Figure 2-source data 11/Patient#1 IP BCR-ABL-IB BCR-ABL.tif]

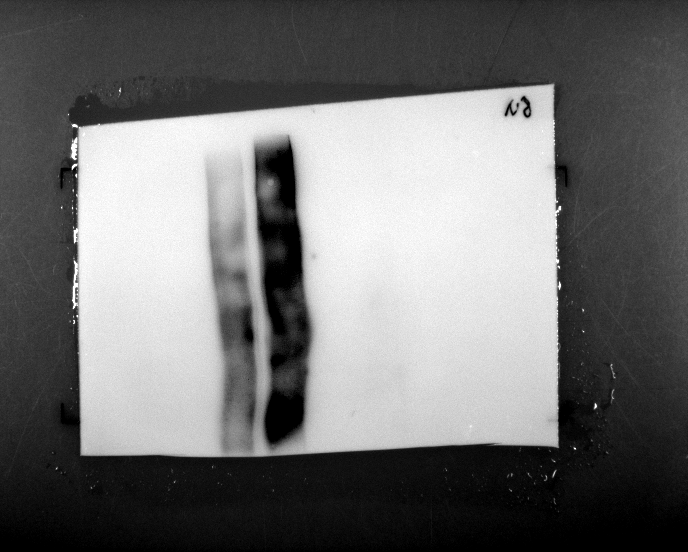

Supplement: Figure 2—source data 11. [file elife-88375-fig2-data11.zip › Figure 2-source data 11/Patient#1 IP BCR-ABL-IB NEDD8.tif]

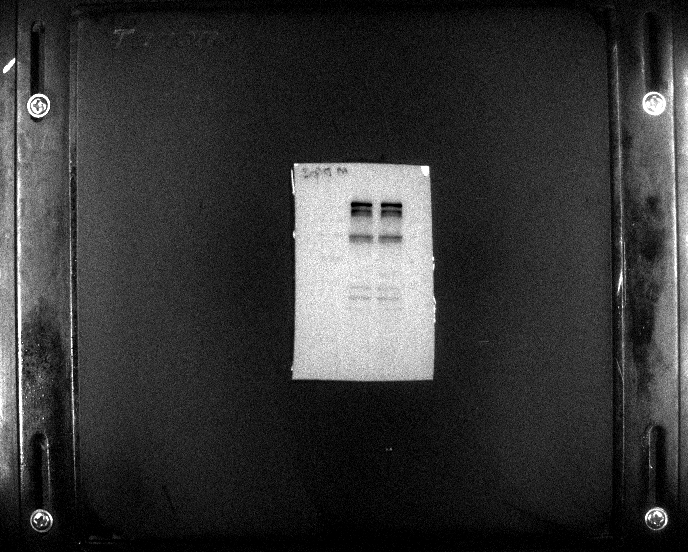

Supplement: Figure 2—source data 11. [file elife-88375-fig2-data11.zip › Figure 2-source data 11/Patient#2 Input BCR-ABL.tif]

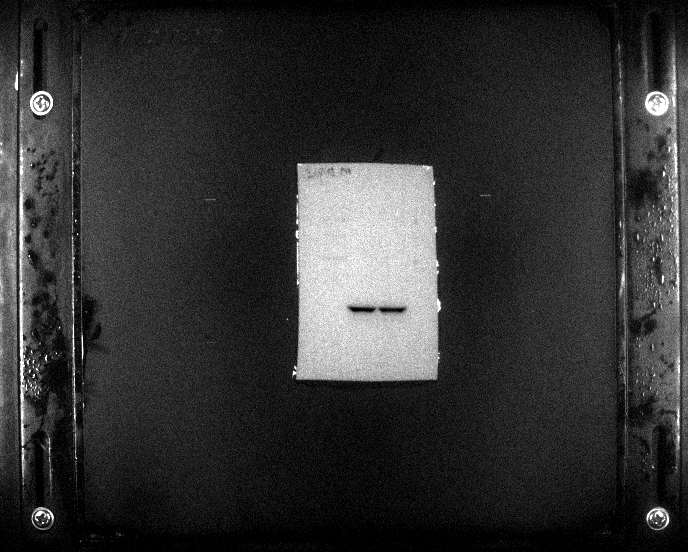

Supplement: Figure 2—source data 11. [file elife-88375-fig2-data11.zip › Figure 2-source data 11/Patient#2 Input a┬-Tubulin.tif]

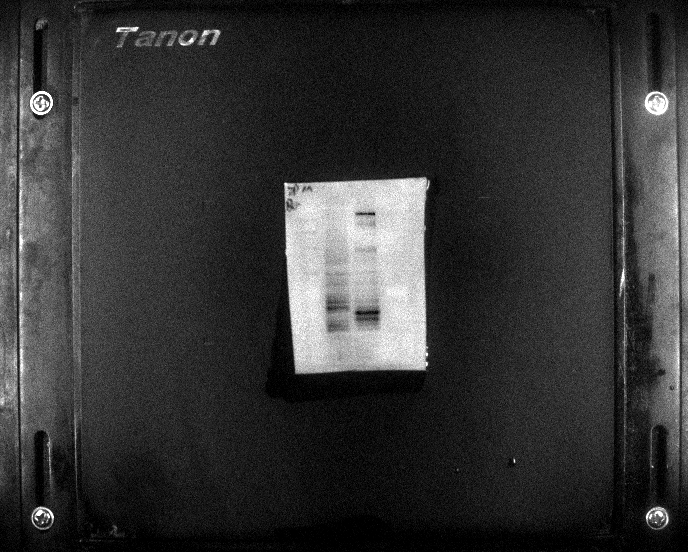

Supplement: Figure 2—source data 11. [file elife-88375-fig2-data11.zip › Figure 2-source data 11/Patient#2 IP BCR-ABL-IB BCR-ABL.tif]

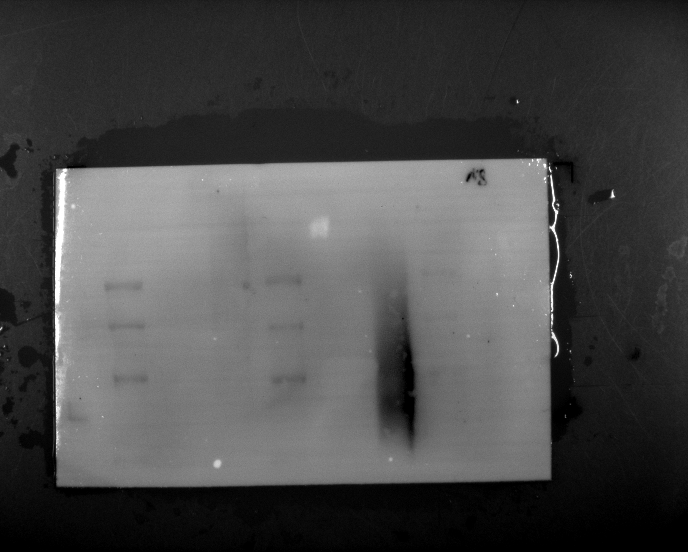

Supplement: Figure 2—source data 11. [file elife-88375-fig2-data11.zip › Figure 2-source data 11/Patient#2 IP BCR-ABL-IB NEDD8.tif]

F

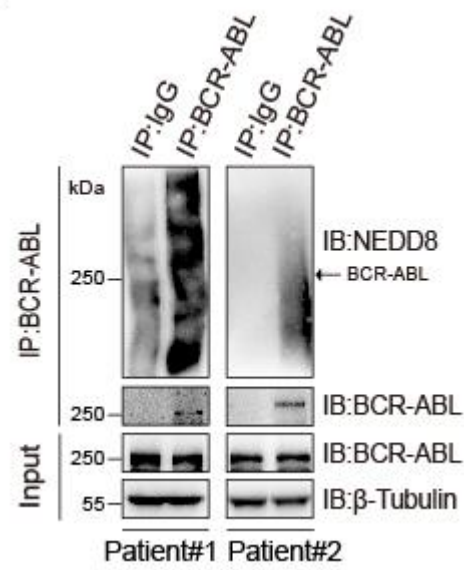

Patient #1

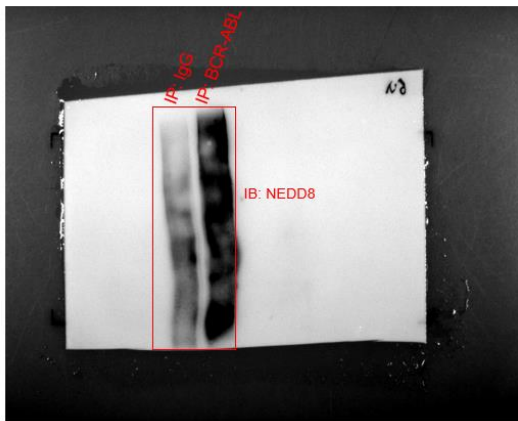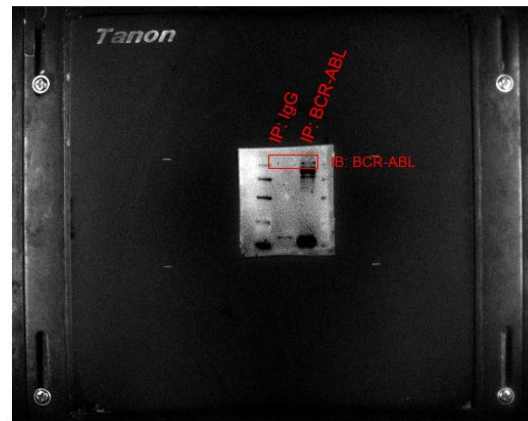

Input

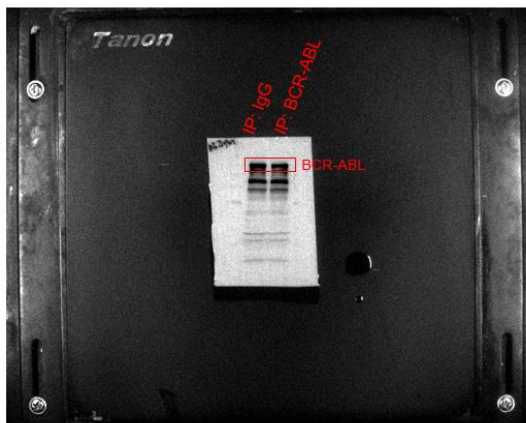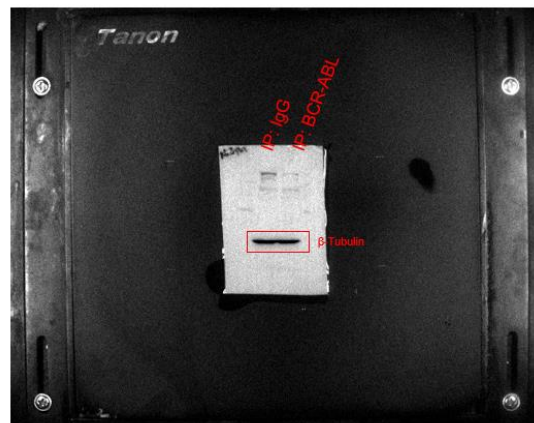

Patient #2

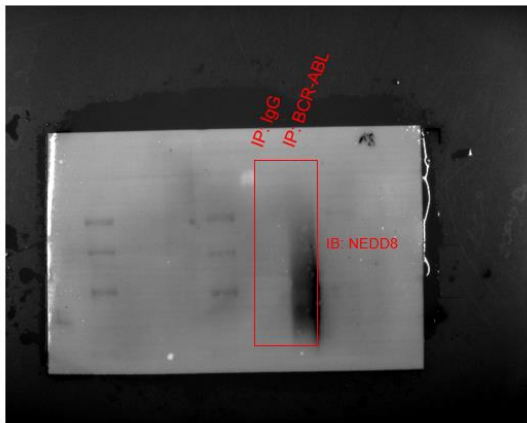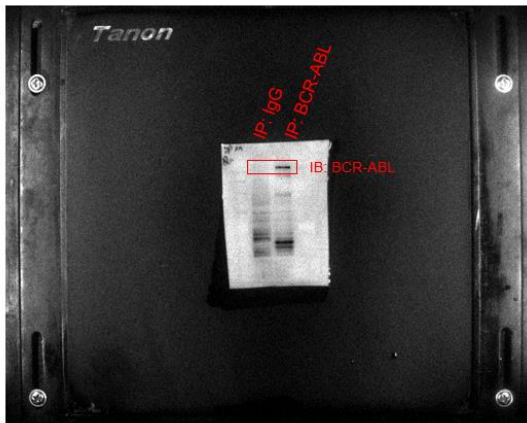

Input

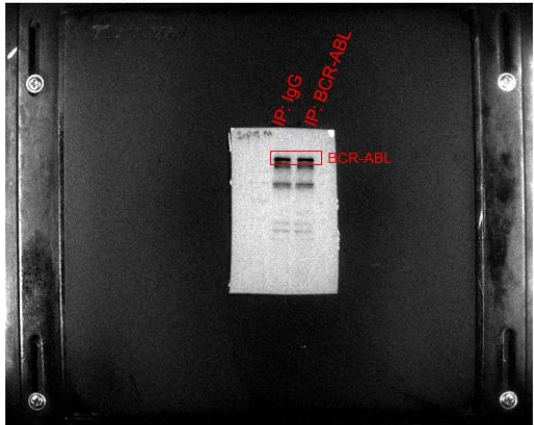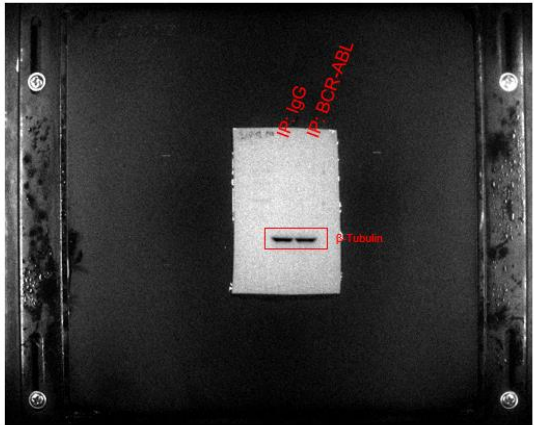

Supplement: Figure 2—source data 12. [file elife-88375-fig2-data12.zip › Figure 2-source data 12/Figure 2-source data 12.pdf]

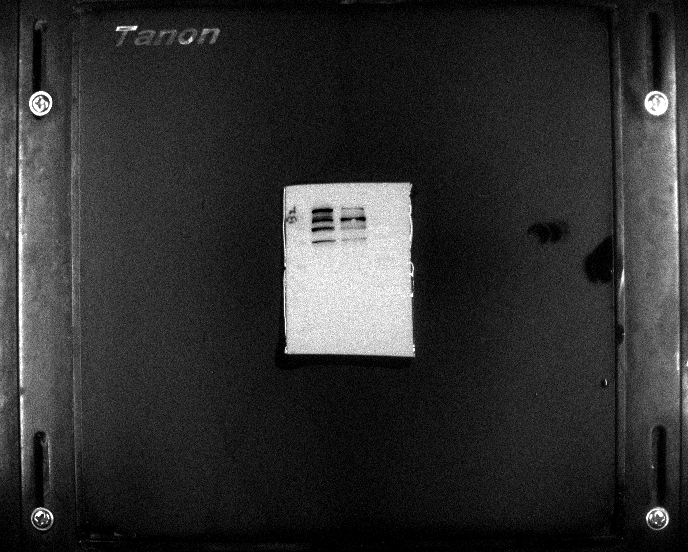

Supplement: Figure 2—source data 13. [file elife-88375-fig2-data13.zip › Figure 2-soure data 13/K562 Input BCR-ABL.tif]

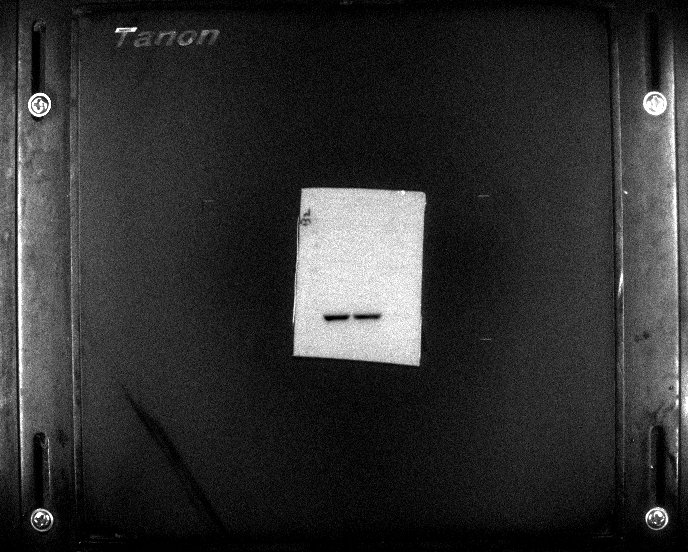

Supplement: Figure 2—source data 13. [file elife-88375-fig2-data13.zip › Figure 2-soure data 13/K562 Input a┬-Tubulin.tif]

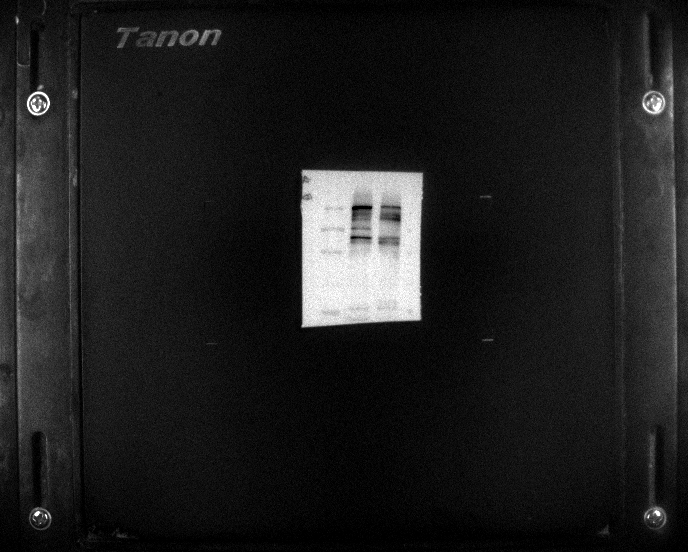

Supplement: Figure 2—source data 13. [file elife-88375-fig2-data13.zip › Figure 2-soure data 13/K562 IP BCR-ABL-IB BCR-ABL.tif]

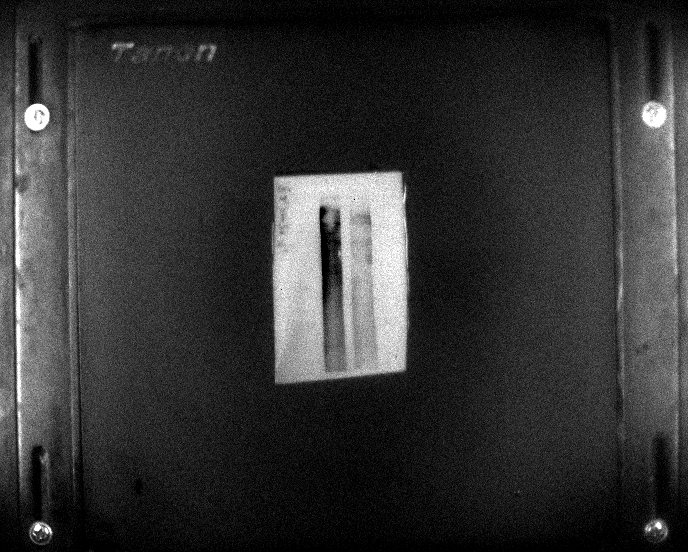

Supplement: Figure 2—source data 13. [file elife-88375-fig2-data13.zip › Figure 2-soure data 13/K562 IP BCR-ABL-IB NEDD8.tif]

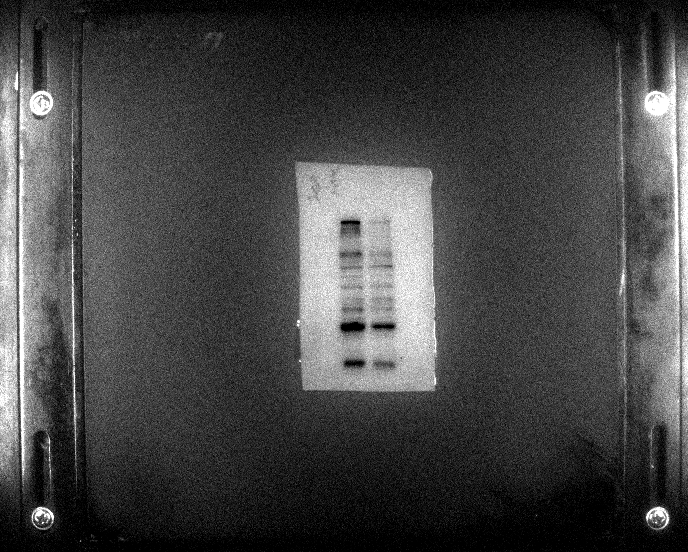

Supplement: Figure 2—source data 13. [file elife-88375-fig2-data13.zip › Figure 2-soure data 13/MEG-01 Input BCR-ABL.tif]

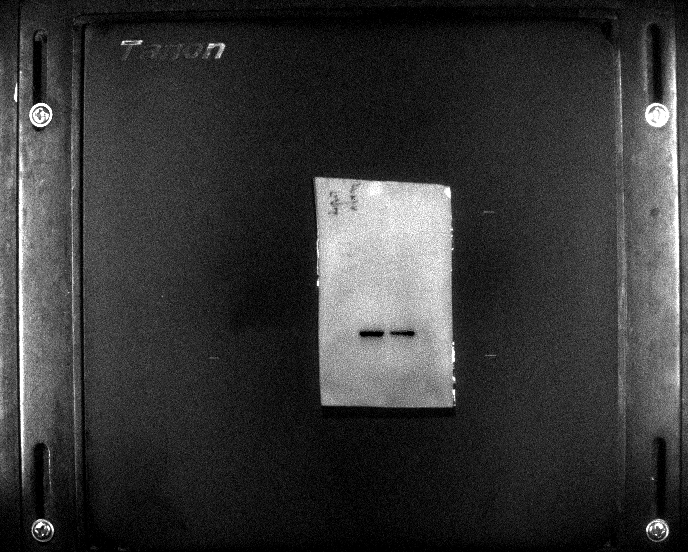

Supplement: Figure 2—source data 13. [file elife-88375-fig2-data13.zip › Figure 2-soure data 13/MEG-01 Input a┬-Tubulin.tif]

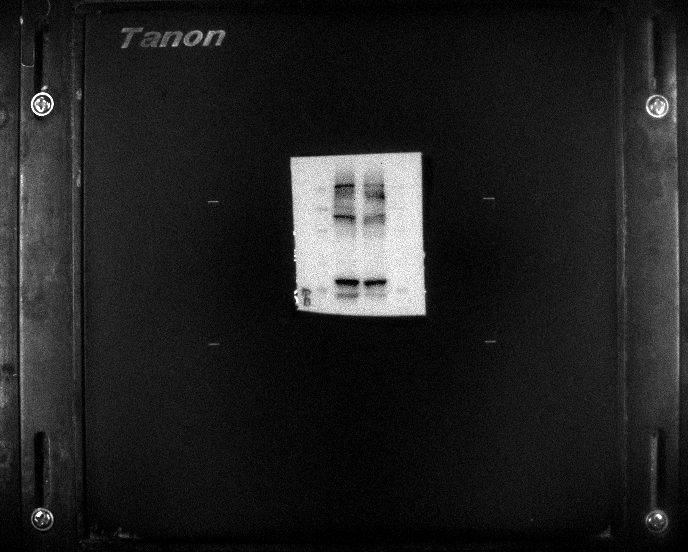

Supplement: Figure 2—source data 13. [file elife-88375-fig2-data13.zip › Figure 2-soure data 13/MEG-01 IP BCR-ABL-IB BCR-ABL.tif]

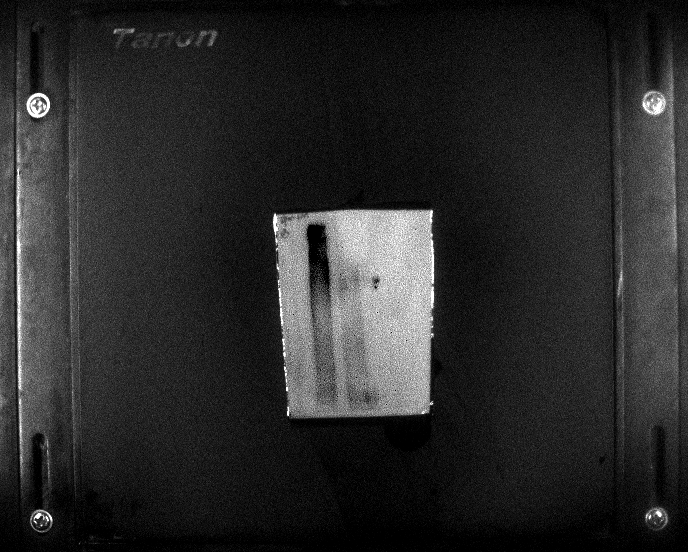

Supplement: Figure 2—source data 13. [file elife-88375-fig2-data13.zip › Figure 2-soure data 13/MEG-01 IP BCR-ABL-IB NEDD8.tif]

G

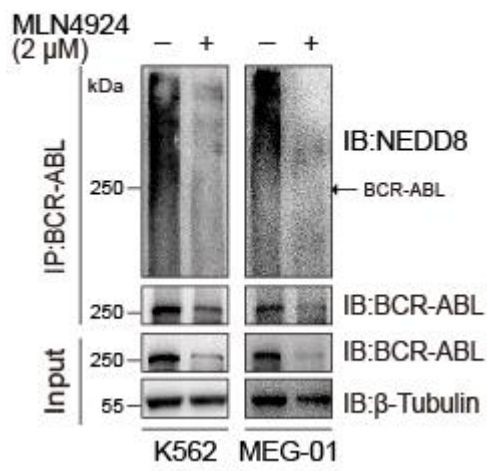

K562

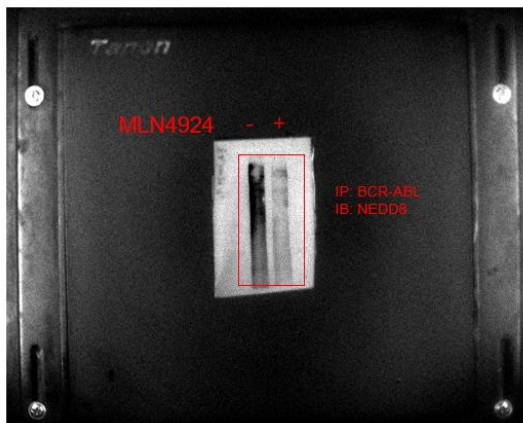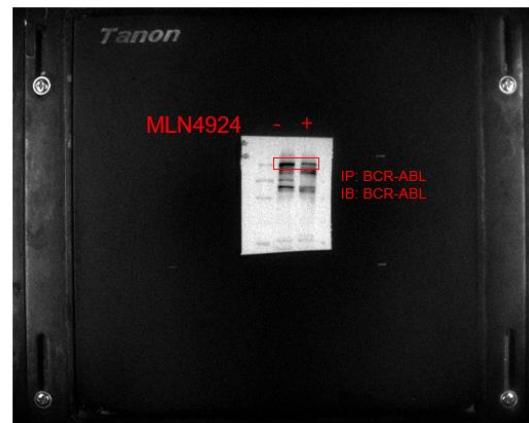

Input

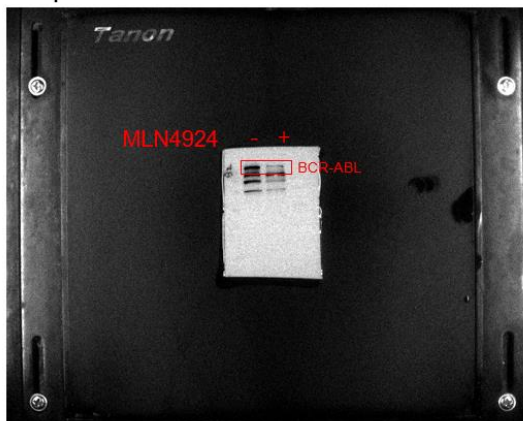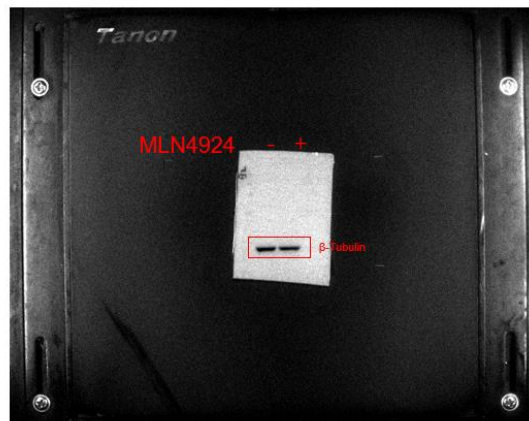

MEG-01

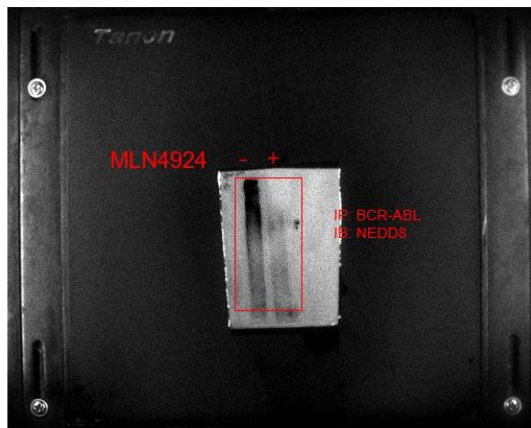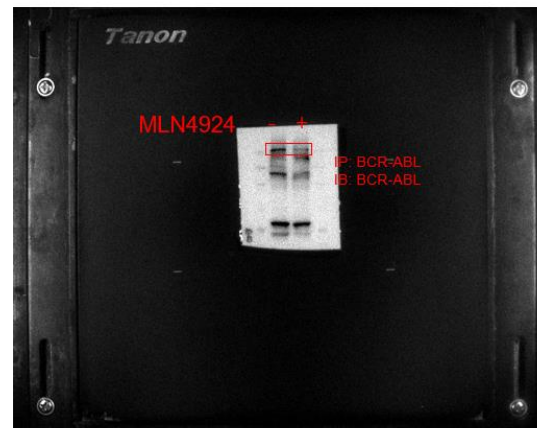

Input

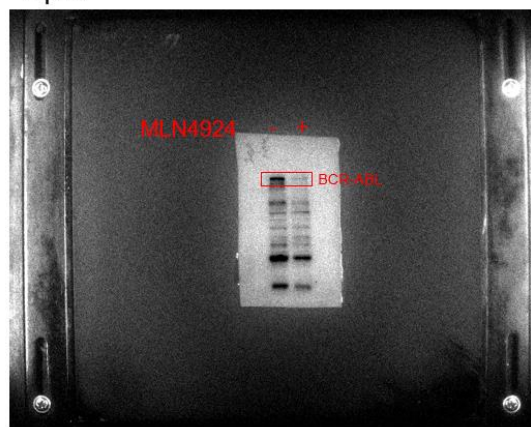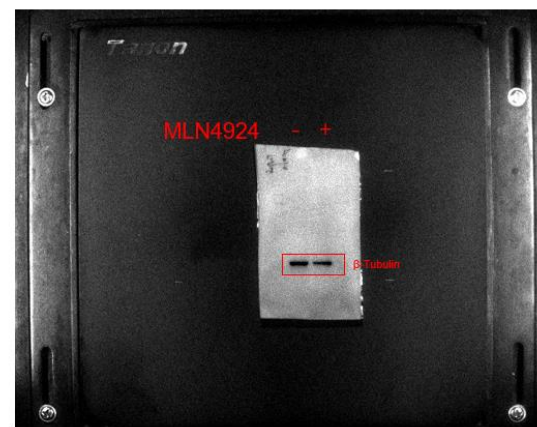

Supplement: Figure 2—source data 14. [file elife-88375-fig2-data14.zip › Figure 2-soure data 14/Figure 2-source data 14.pdf]

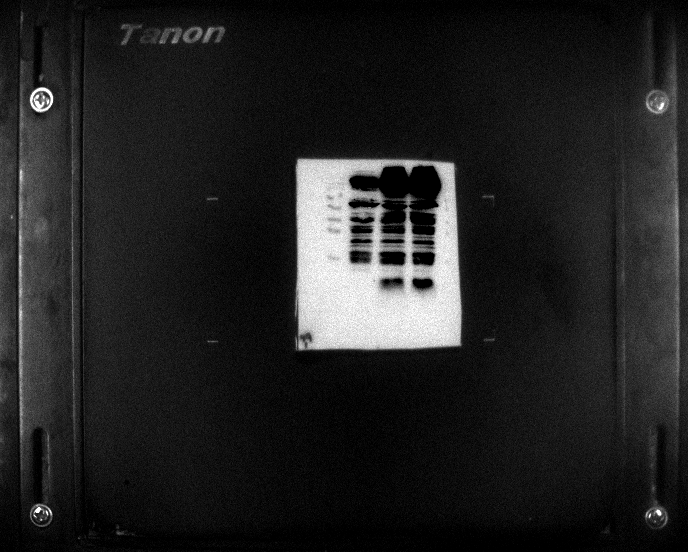

Supplement: Figure 2—source data 15. [file elife-88375-fig2-data15.zip › Figure 2-source data 15/Input HA.tif]

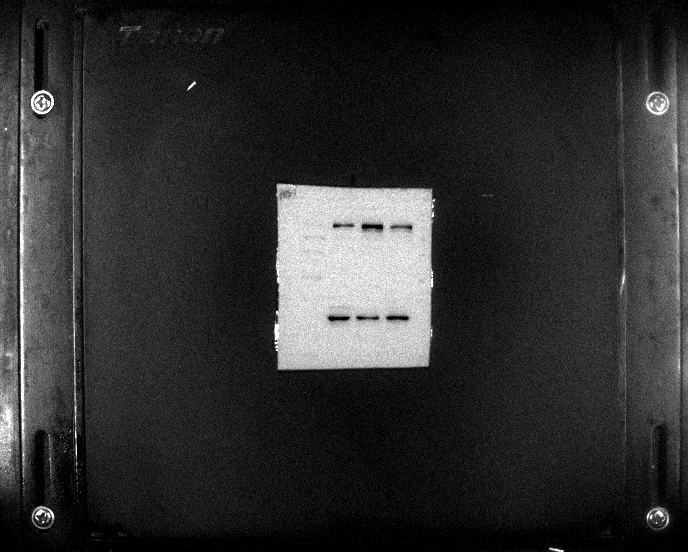

Supplement: Figure 2—source data 15. [file elife-88375-fig2-data15.zip › Figure 2-source data 15/Input His.tif]

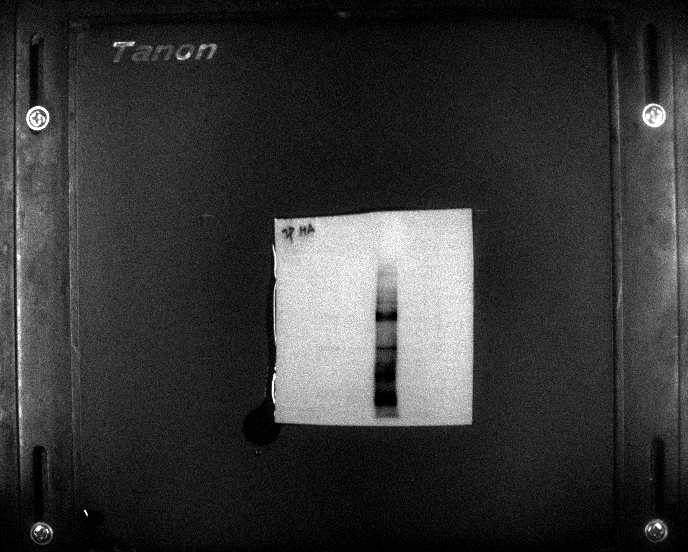

Supplement: Figure 2—source data 15. [file elife-88375-fig2-data15.zip › Figure 2-source data 15/IP His-IB HA.tif]

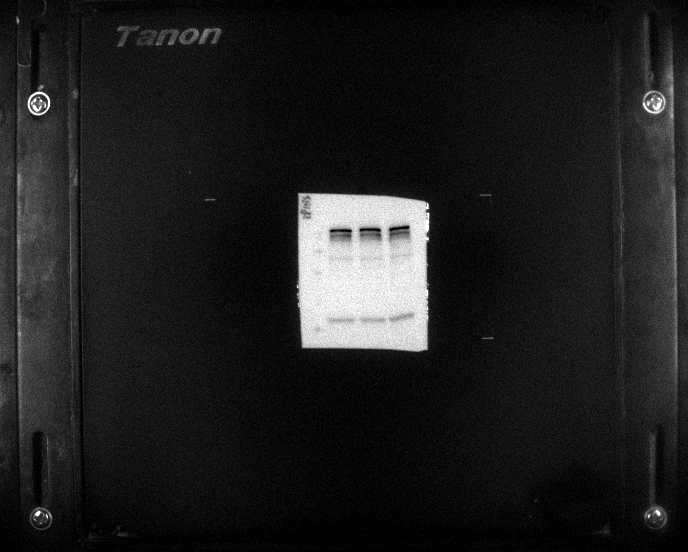

Supplement: Figure 2—source data 15. [file elife-88375-fig2-data15.zip › Figure 2-source data 15/IP His-IB His-2.tif]

H

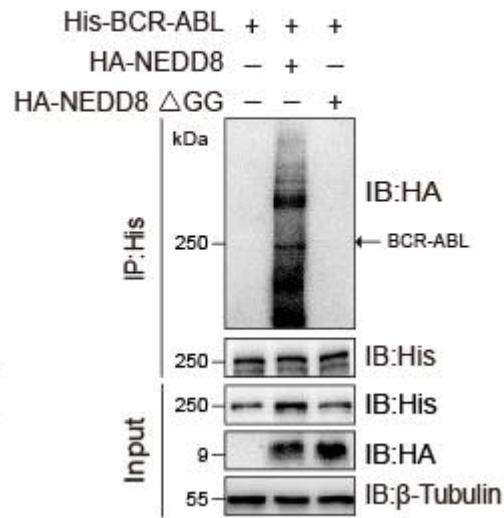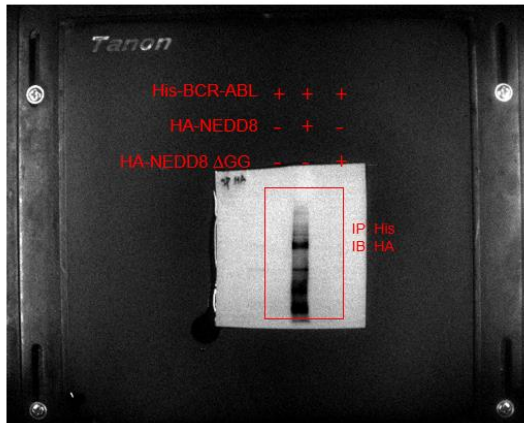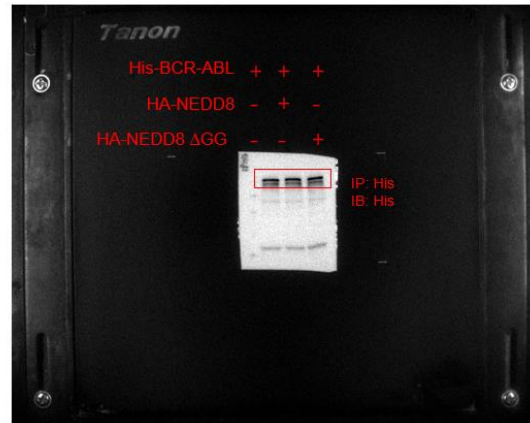

Input

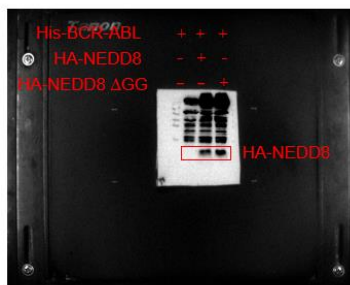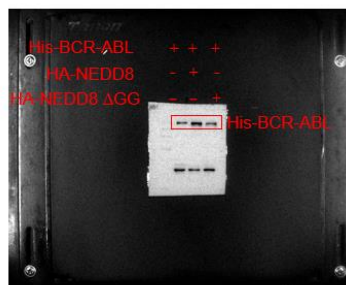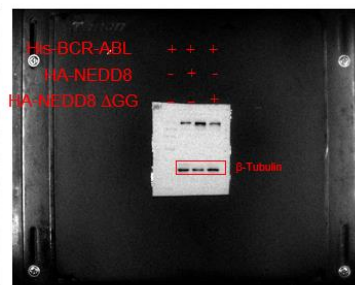

Supplement: Figure 2—source data 16. [file elife-88375-fig2-data16.zip › Figure 2-source data 16/Figure 2-source data 16.pdf]

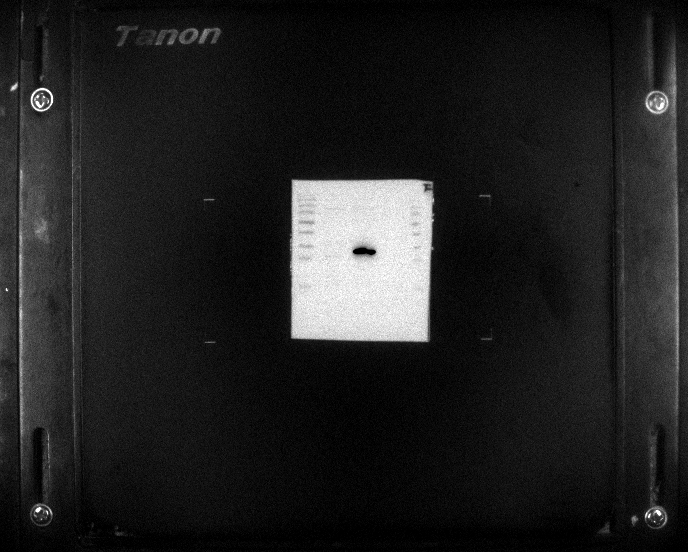

Supplement: Figure 2—source data 17. [file elife-88375-fig2-data17.zip › Figure 2-source data 17/Input Flag.tif]

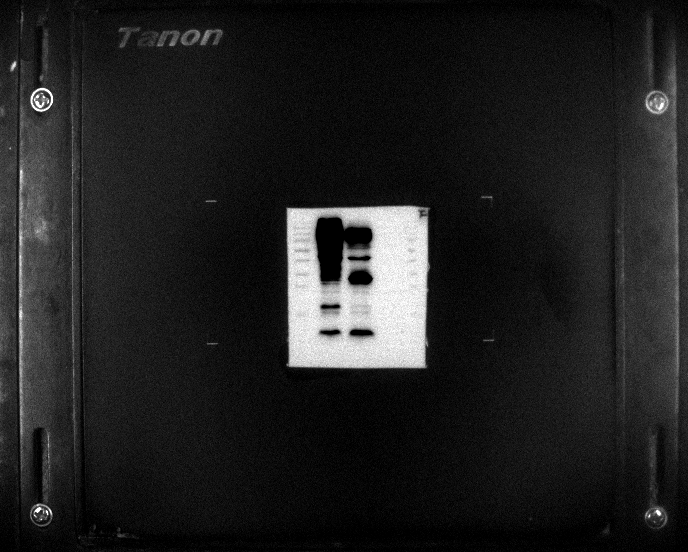

Supplement: Figure 2—source data 17. [file elife-88375-fig2-data17.zip › Figure 2-source data 17/Input HA.tif]

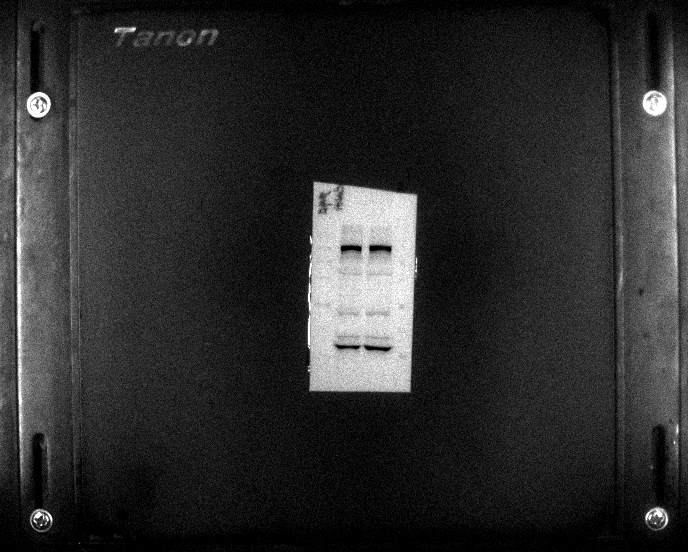

Supplement: Figure 2—source data 17. [file elife-88375-fig2-data17.zip › Figure 2-source data 17/Input His.tif]

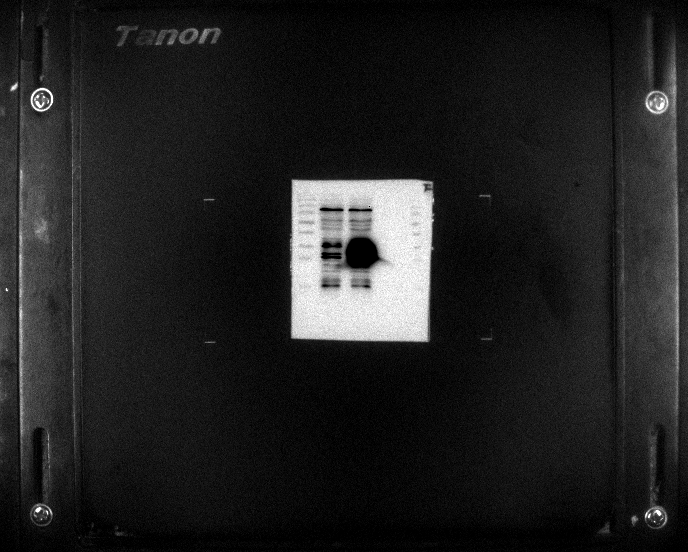

Supplement: Figure 2—source data 17. [file elife-88375-fig2-data17.zip › Figure 2-source data 17/Input a┬-Tubulin-1.tif]

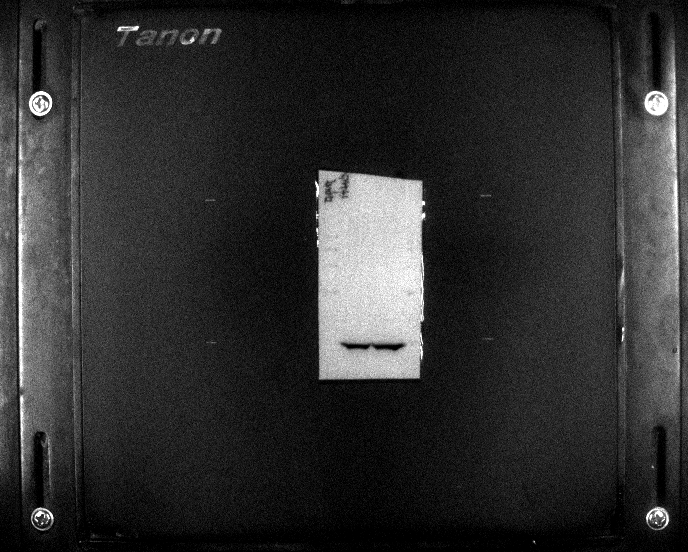

Supplement: Figure 2—source data 17. [file elife-88375-fig2-data17.zip › Figure 2-source data 17/Input a┬-Tubulin-2.tif]

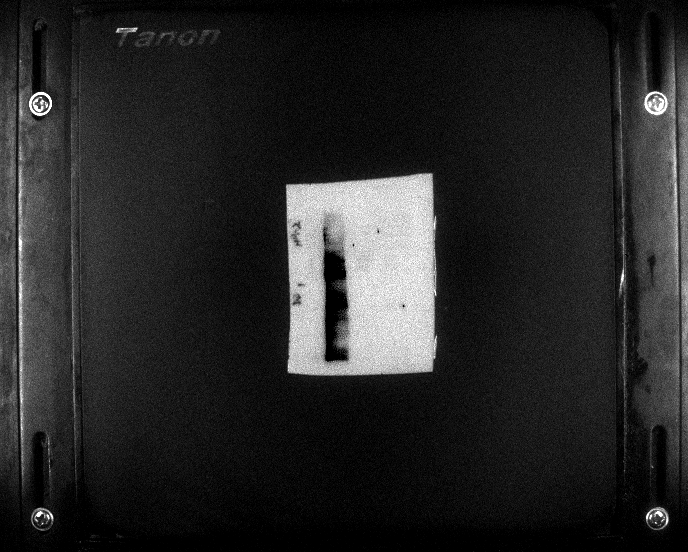

Supplement: Figure 2—source data 17. [file elife-88375-fig2-data17.zip › Figure 2-source data 17/IP His-IB HA.tif]

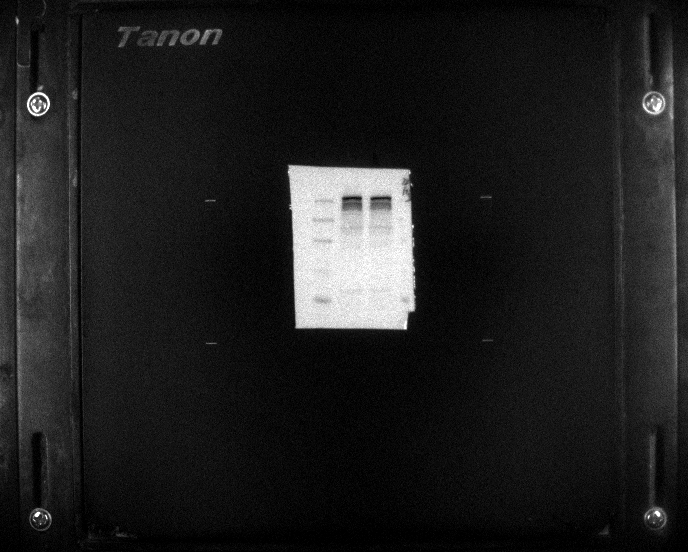

Supplement: Figure 2—source data 17. [file elife-88375-fig2-data17.zip › Figure 2-source data 17/IP His-IB His.tif]

His-BCR-ABL + +  
 Flag-NEDP1 - +  
 HA-NEDD8 + +

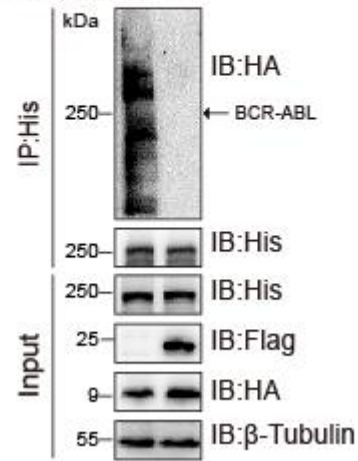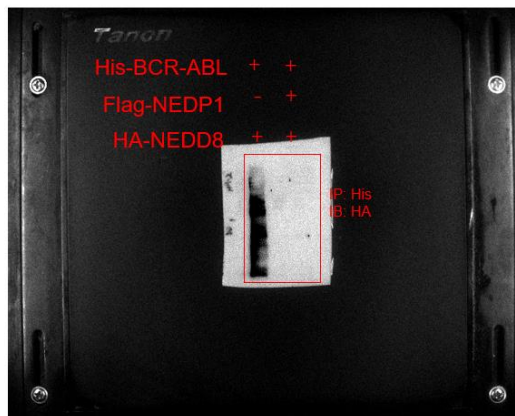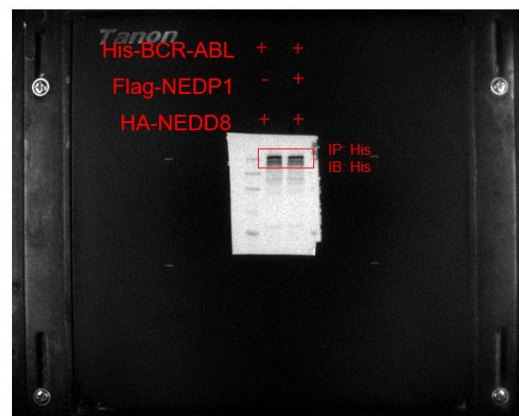

Input

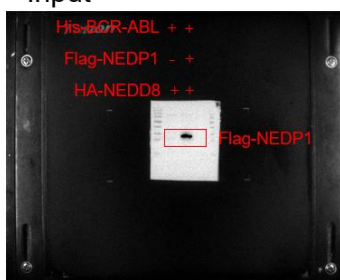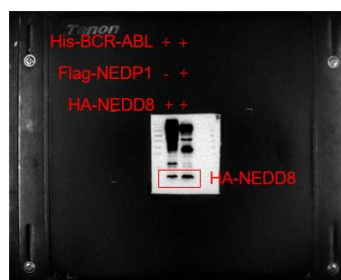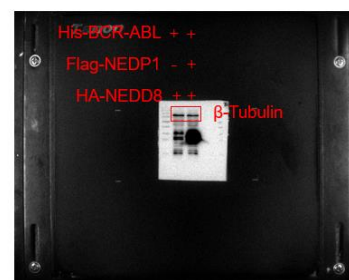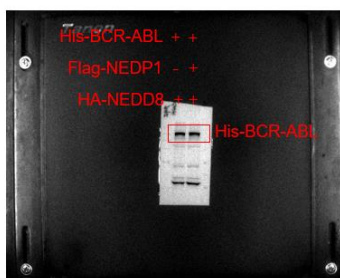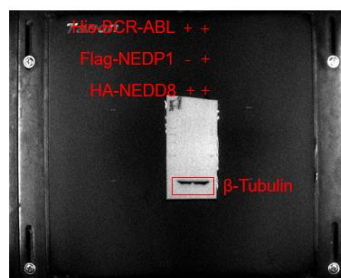

Supplement: Figure 2—source data 18. [file elife-88375-fig2-data18.zip › Figure 2-source data 18/Figure 2-source data 18.pdf]

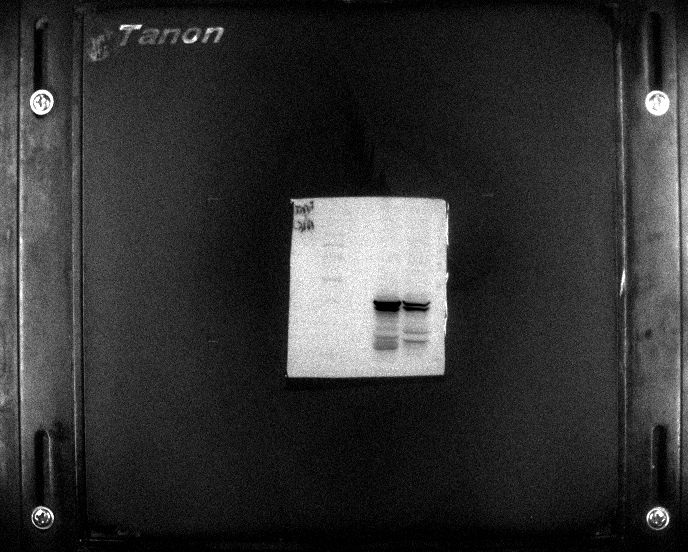

Supplement: Figure 2—source data 19. [file elife-88375-fig2-data19.zip › Figure 2-source data 19/Input GFP.tif]

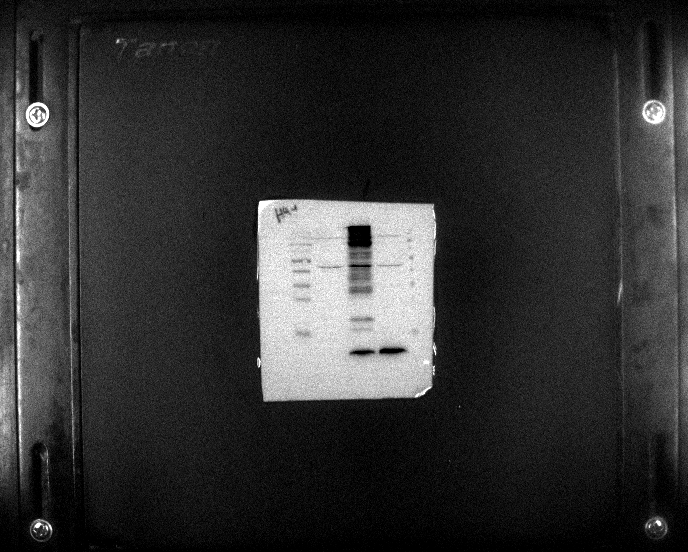

Supplement: Figure 2—source data 19. [file elife-88375-fig2-data19.zip › Figure 2-source data 19/Input HA.tif]

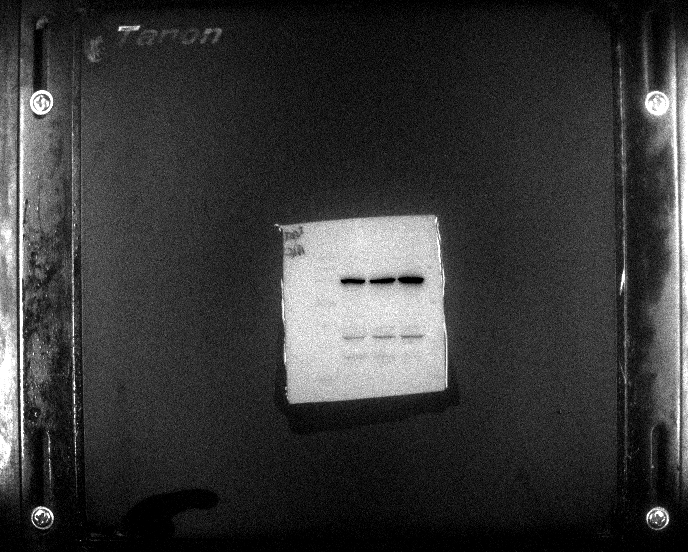

Supplement: Figure 2—source data 19. [file elife-88375-fig2-data19.zip › Figure 2-source data 19/Input His.tif]

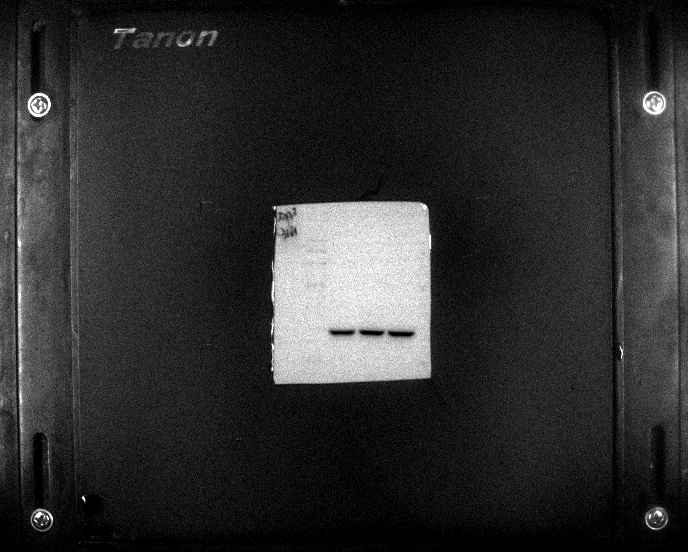

Supplement: Figure 2—source data 19. [file elife-88375-fig2-data19.zip › Figure 2-source data 19/Input a┬-Tubulin.tif]

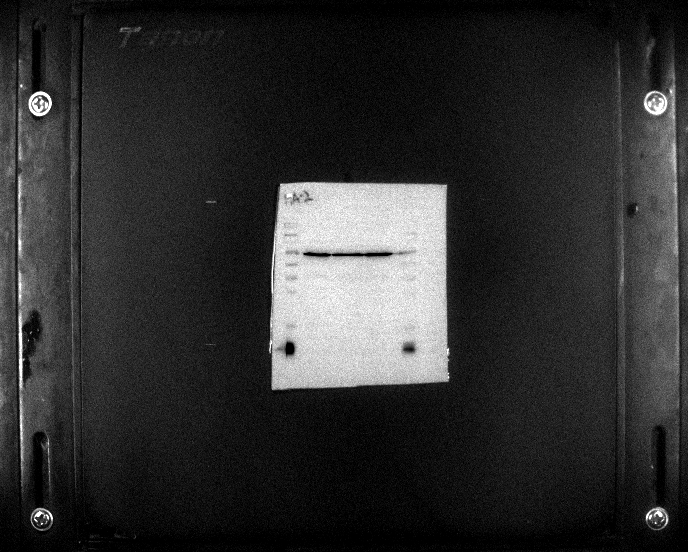

Supplement: Figure 2—source data 19. [file elife-88375-fig2-data19.zip › Figure 2-source data 19/Input a┬-Tubulin-2 (1).tif]
