# Supplementary figures and images for: RAPSYN-mediated neddylation of BCR-ABL alternatively determines the fate of Philadelphia chromosome-positive leukemia (part 2 of 5)
Source: eLife. 2024 Jun 12;12:RP88375. doi: 10.7554/eLife.88375 (PMC11168747; doi:10.7554/eLife.88375)

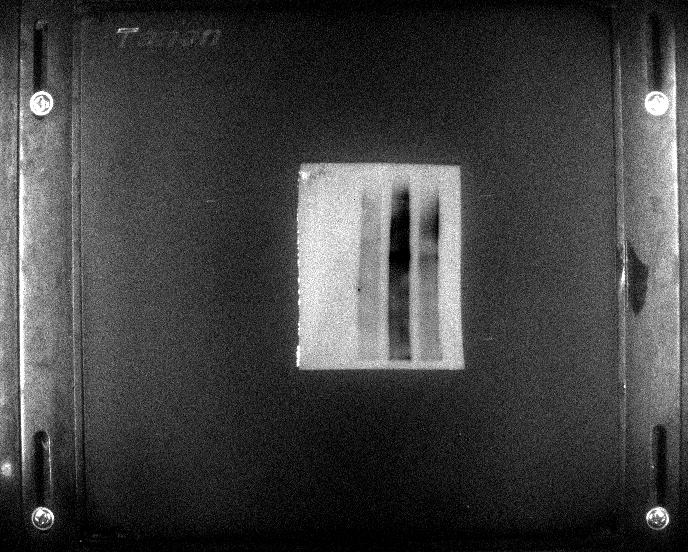

Supplement: Figure 2—source data 19. [file elife-88375-fig2-data19.zip › Figure 2-source data 19/IP His-IB HA.tif]

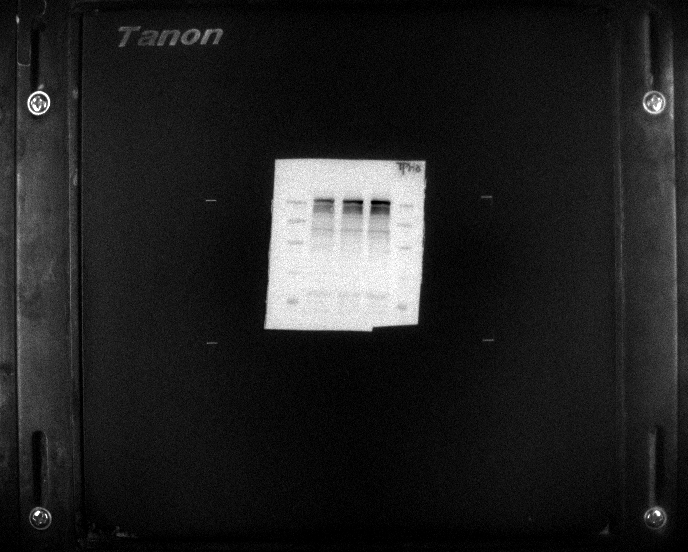

Supplement: Figure 2—source data 19. [file elife-88375-fig2-data19.zip › Figure 2-source data 19/IP His-IB His.tif]

J

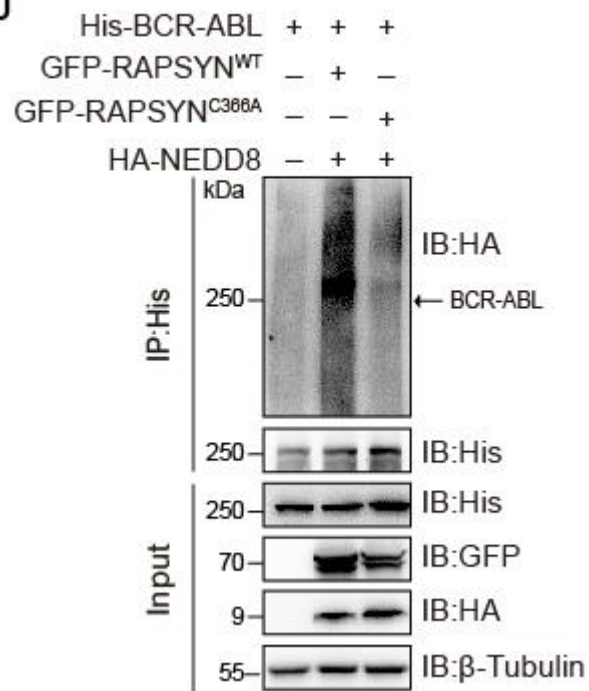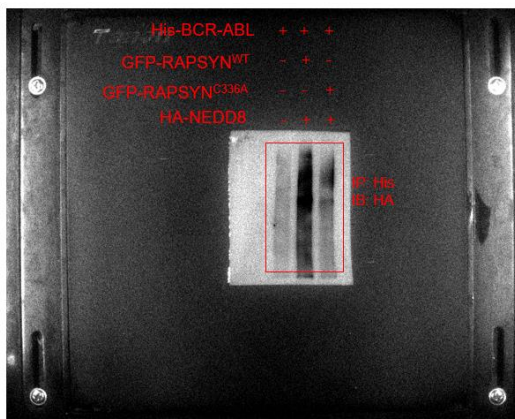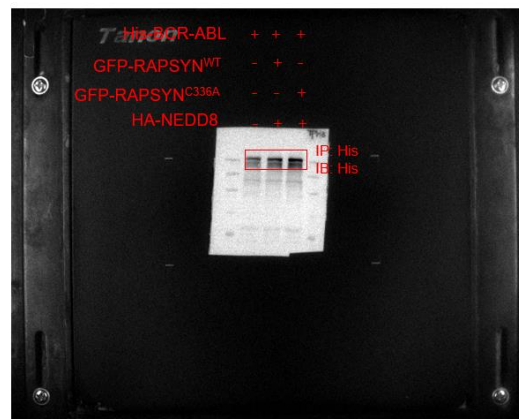

Input

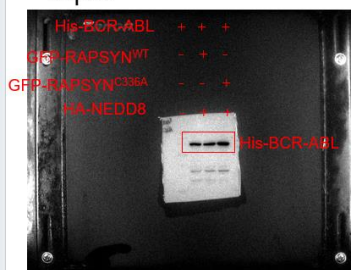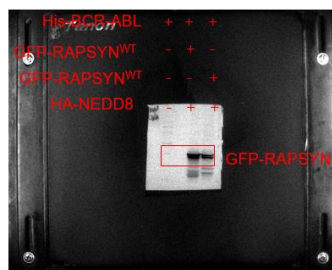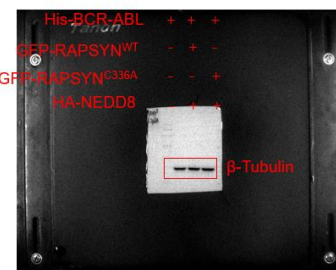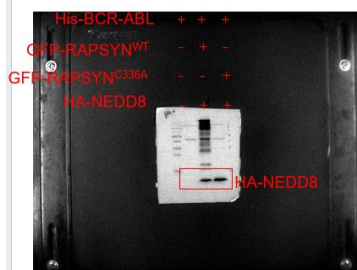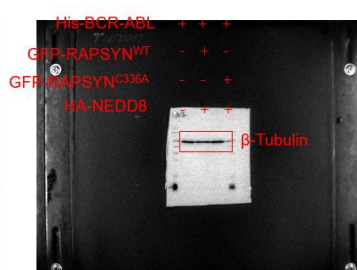

Supplement: Figure 2—source data 20. [file elife-88375-fig2-data20.zip › Figure 2-source data 20/Figure 2-source data 20.pdf]

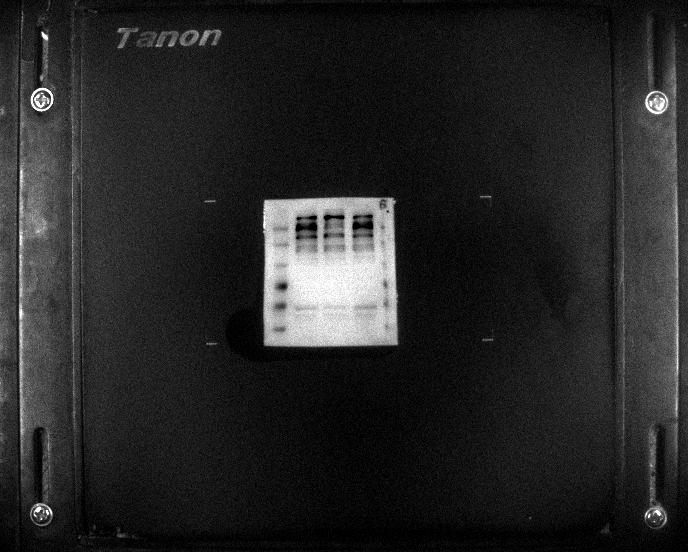

Supplement: Figure 2—source data 21. [file elife-88375-fig2-data21.zip › Figure 2-source data 21/Input BCR-ABL.tif]

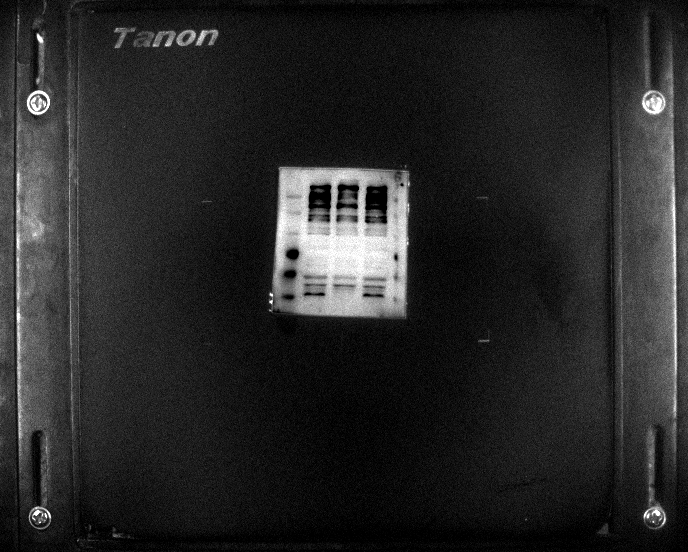

Supplement: Figure 2—source data 21. [file elife-88375-fig2-data21.zip › Figure 2-source data 21/Input RAPSYN.tif]

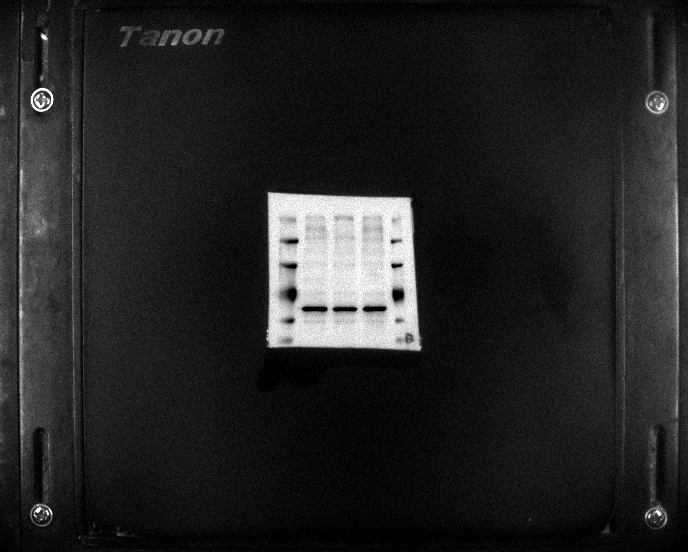

Supplement: Figure 2—source data 21. [file elife-88375-fig2-data21.zip › Figure 2-source data 21/Input a┬-Tubulin.tif]

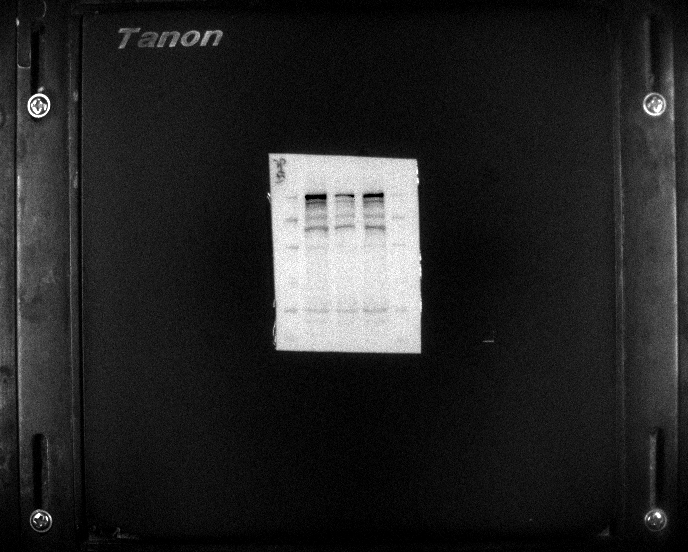

Supplement: Figure 2—source data 21. [file elife-88375-fig2-data21.zip › Figure 2-source data 21/IP BCR-ABL-IB BCR-ABL.tif]

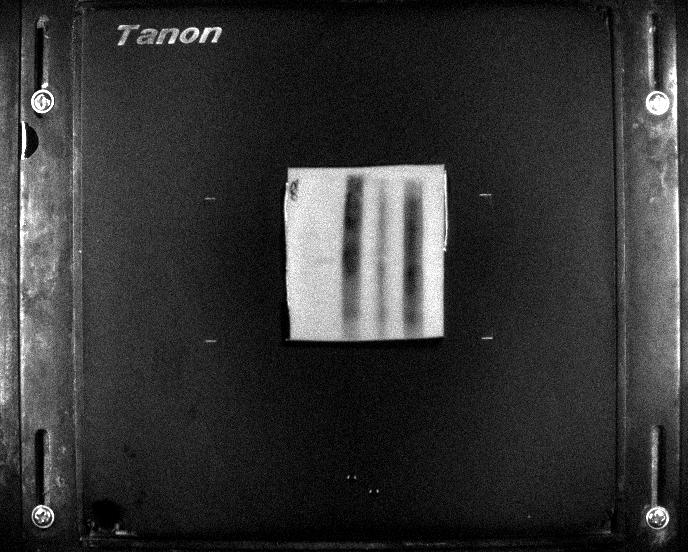

Supplement: Figure 2—source data 21. [file elife-88375-fig2-data21.zip › Figure 2-source data 21/IP BCR-ABL-IB NEDD8.tif]

K

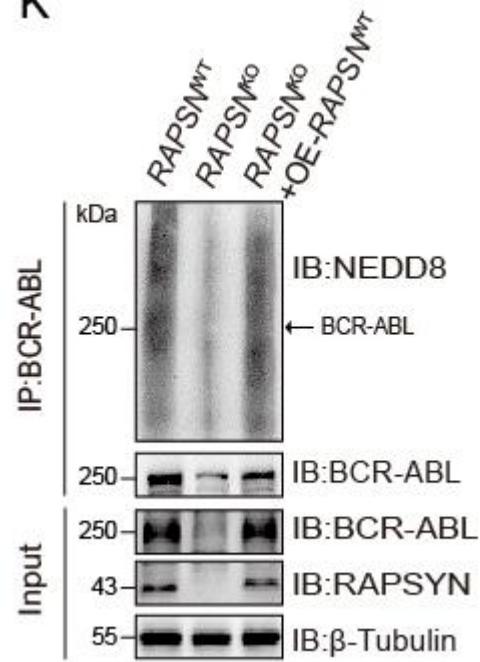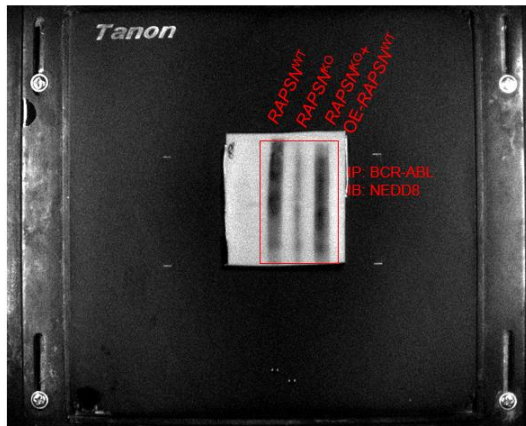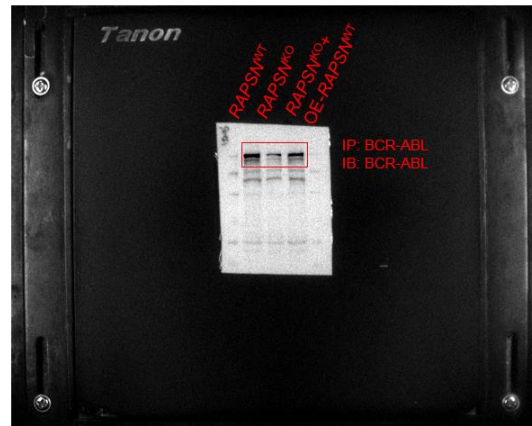

Input

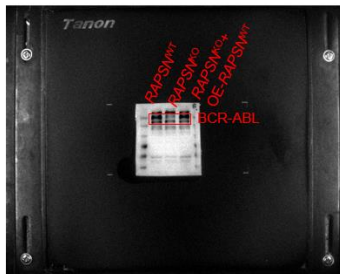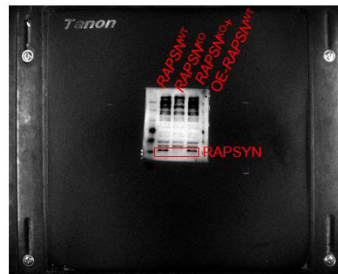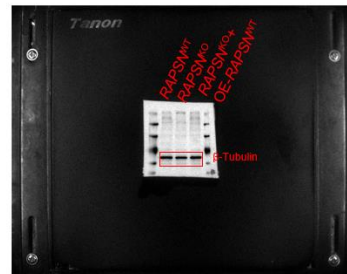

Supplement: Figure 2—source data 22. [file elife-88375-fig2-data22.zip › Figure 2-source data 22/Figure 2-source data 22.pdf]

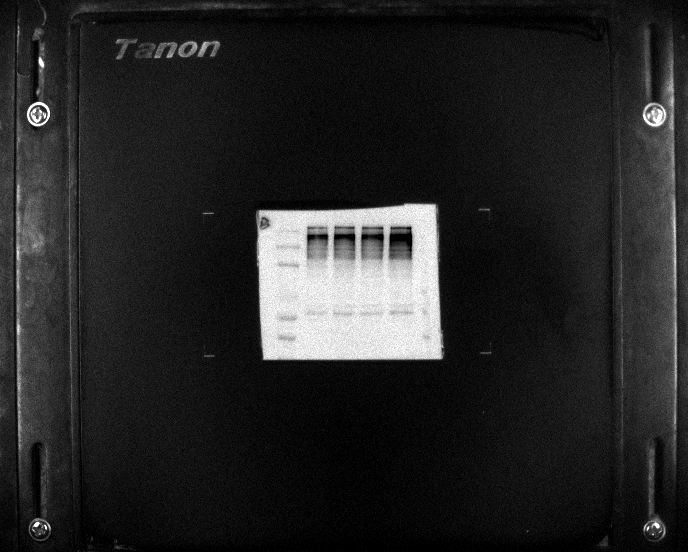

Supplement: Figure 2—source data 23. [file elife-88375-fig2-data23.zip › Figure 2-source data 23/BCR-ABL.tif]

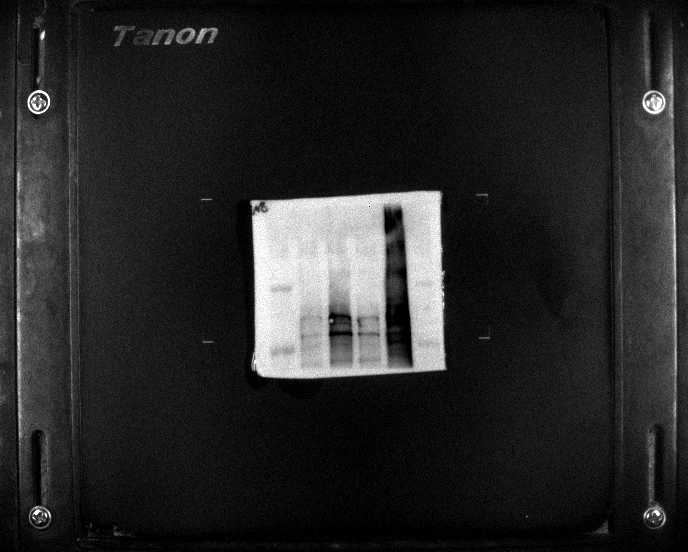

Supplement: Figure 2—source data 23. [file elife-88375-fig2-data23.zip › Figure 2-source data 23/NEDD8.tif]

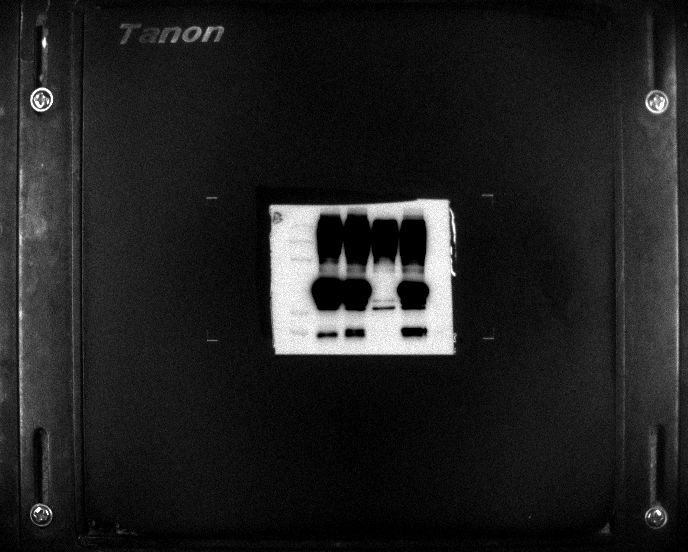

Supplement: Figure 2—source data 23. [file elife-88375-fig2-data23.zip › Figure 2-source data 23/RAPSYN.tif]

L

|                      |   |   |   |   |
|----------------------|---|---|---|---|
| ATP-Mg <sup>2+</sup> | + | + | + | + |
| APPBP1/UBA3          | - | + | + | + |
| UBE2M                | + | - | + | + |
| RAPSYN               | + | + | - | + |
| NEDD8                | + | + | + | + |
| His-BCR-ABL          | + | + | + | + |

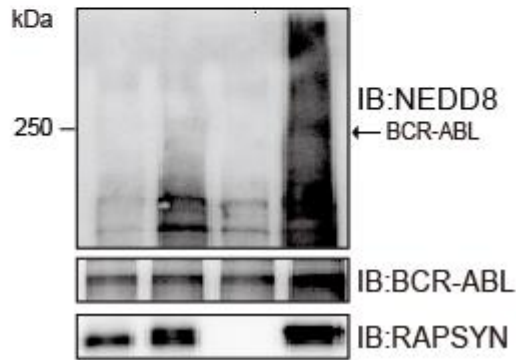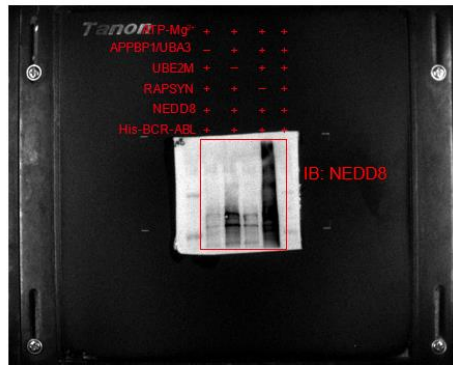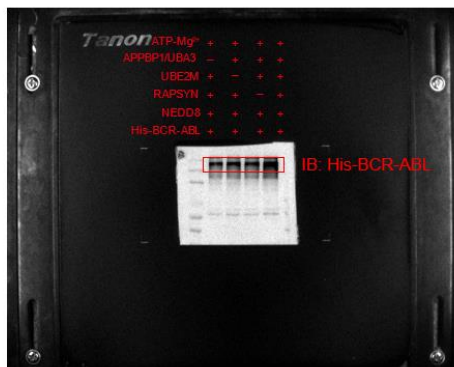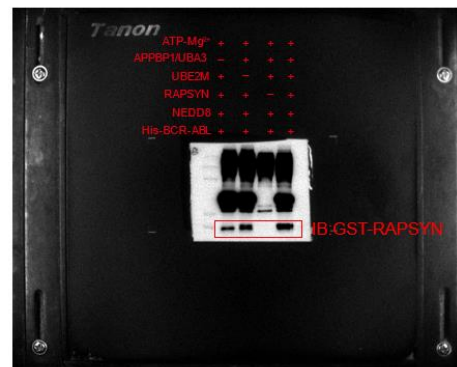

Supplement: Figure 2—source data 24. [file elife-88375-fig2-data24.zip › Figure 2-source data 24/Figure 2-source data 24.pdf]

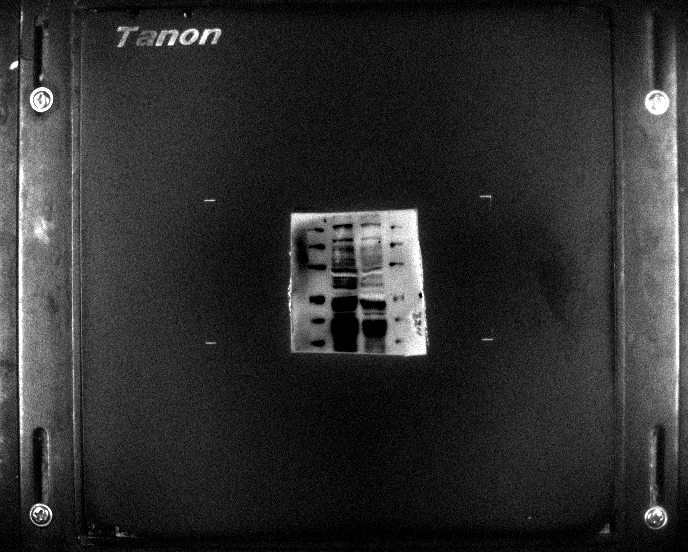

Supplement: Figure 2—source data 25. [file elife-88375-fig2-data25.zip › Figure 2-source data 25/Input BCR-ABL.tif]

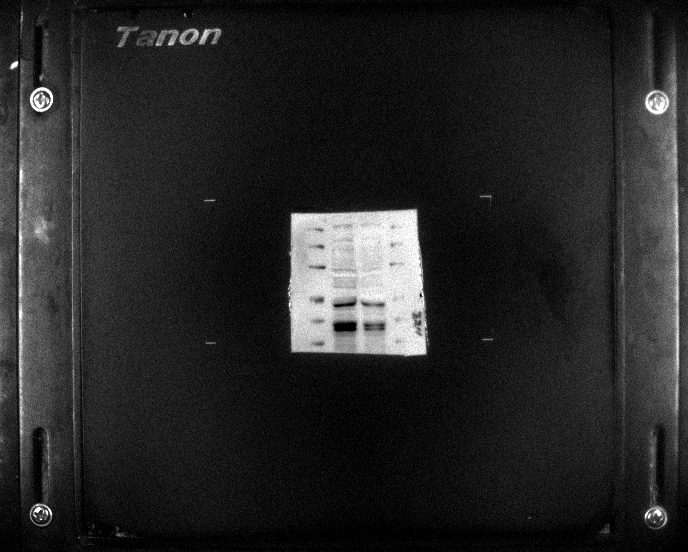

Supplement: Figure 2—source data 25. [file elife-88375-fig2-data25.zip › Figure 2-source data 25/Input RAPSYN.tif]

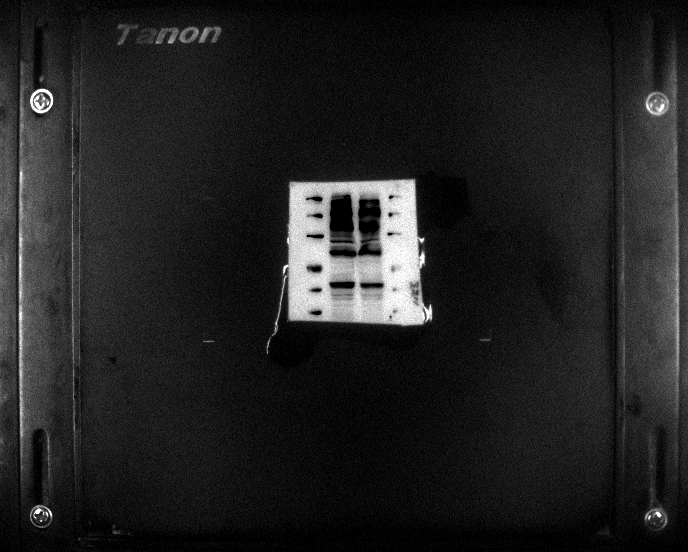

Supplement: Figure 2—source data 25. [file elife-88375-fig2-data25.zip › Figure 2-source data 25/Input a┬-Tubulin.tif]

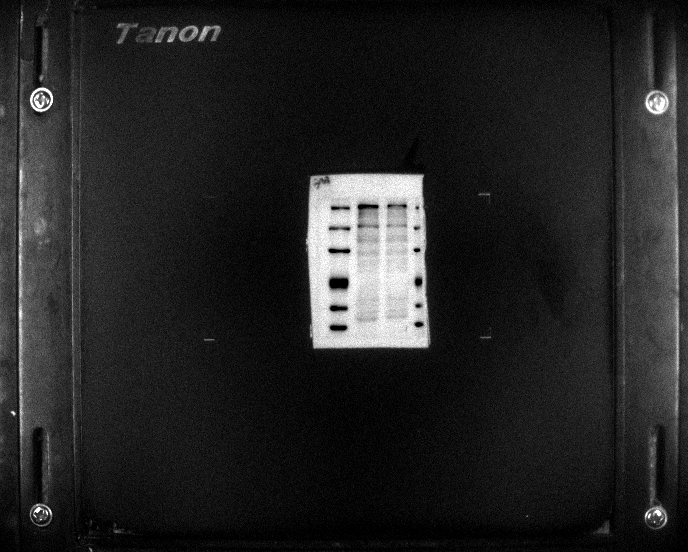

Supplement: Figure 2—source data 25. [file elife-88375-fig2-data25.zip › Figure 2-source data 25/IP BCR-ABL-IB BCR-ABL.tif]

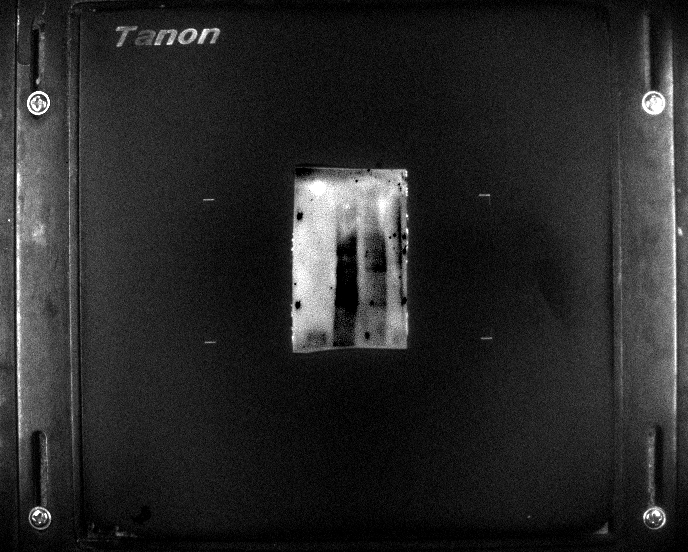

Supplement: Figure 2—source data 25. [file elife-88375-fig2-data25.zip › Figure 2-source data 25/NEDD8-2.tif]

M

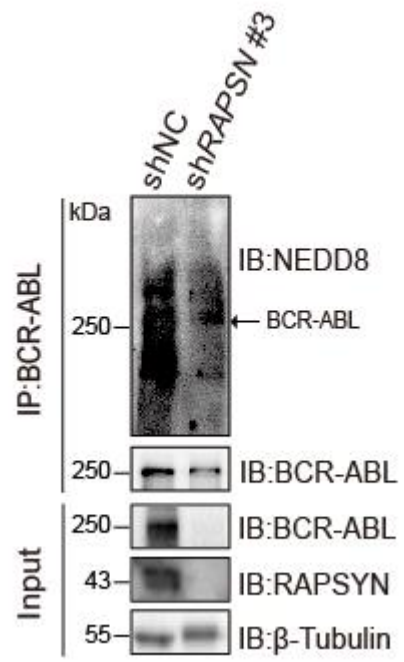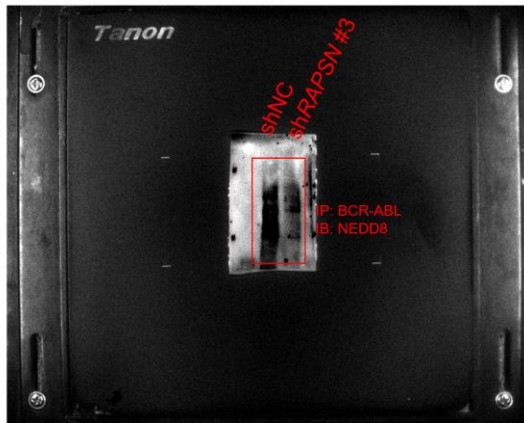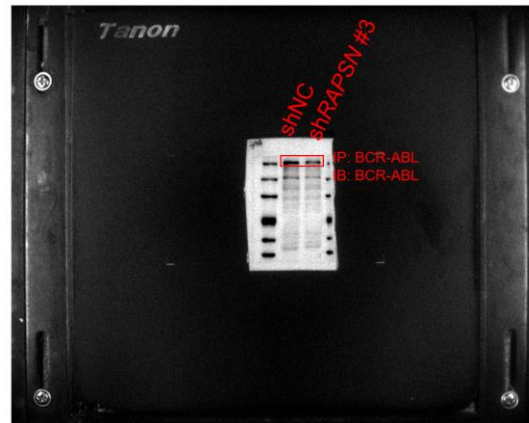

Input

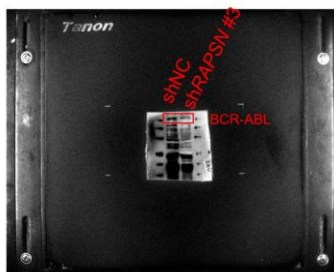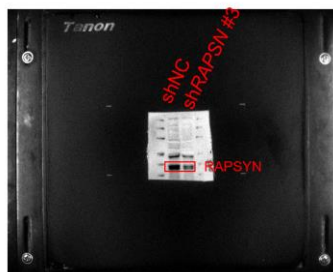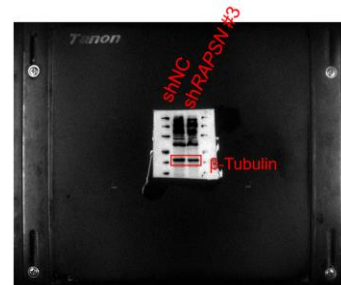

Supplement: Figure 2—source data 26. [file elife-88375-fig2-data26.zip › Figure 2-source data 26/Figure 2-source dara 26.pdf]

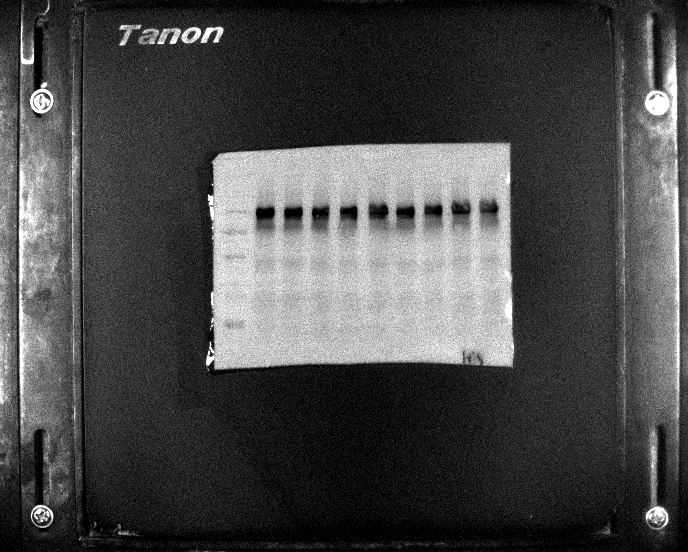

Supplement: Figure 2—source data 27. [file elife-88375-fig2-data27.zip › Figure 2-source data 27/Input His.tif]

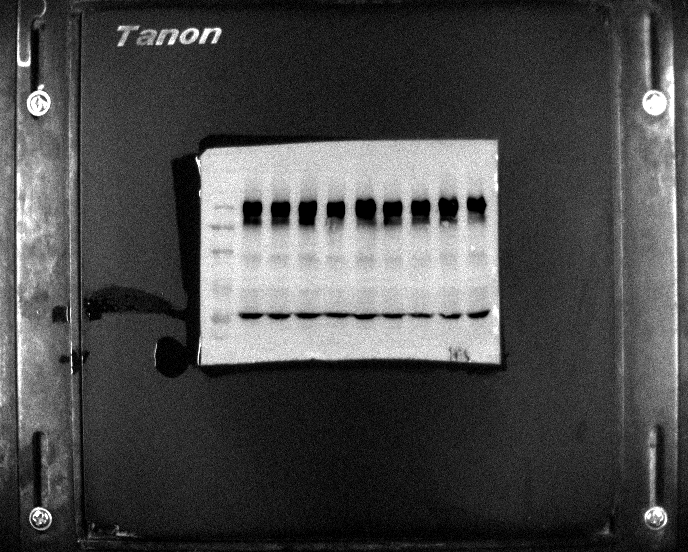

Supplement: Figure 2—source data 27. [file elife-88375-fig2-data27.zip › Figure 2-source data 27/Input a┬-Tubulin.tif]

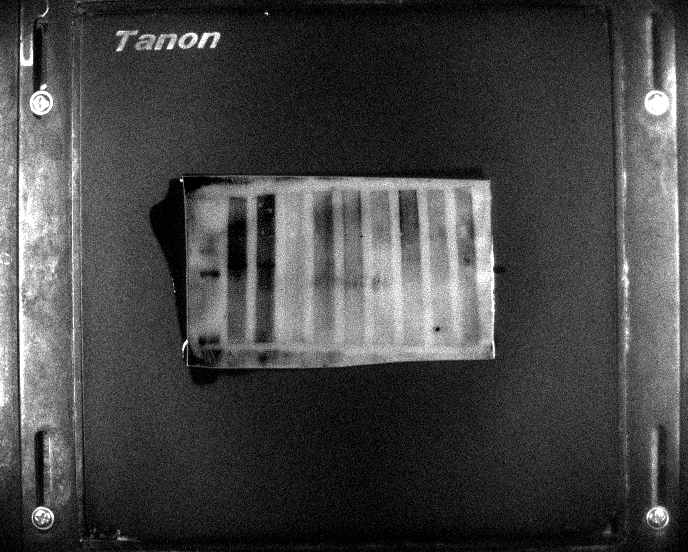

Supplement: Figure 2—source data 27. [file elife-88375-fig2-data27.zip › Figure 2-source data 27/IP His-IB HA.tif]

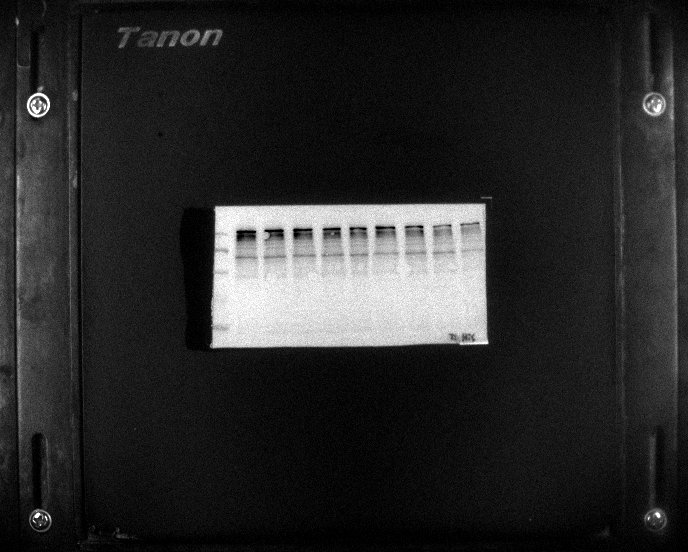

Supplement: Figure 2—source data 27. [file elife-88375-fig2-data27.zip › Figure 2-source data 27/IP His-IB His.tif]

N

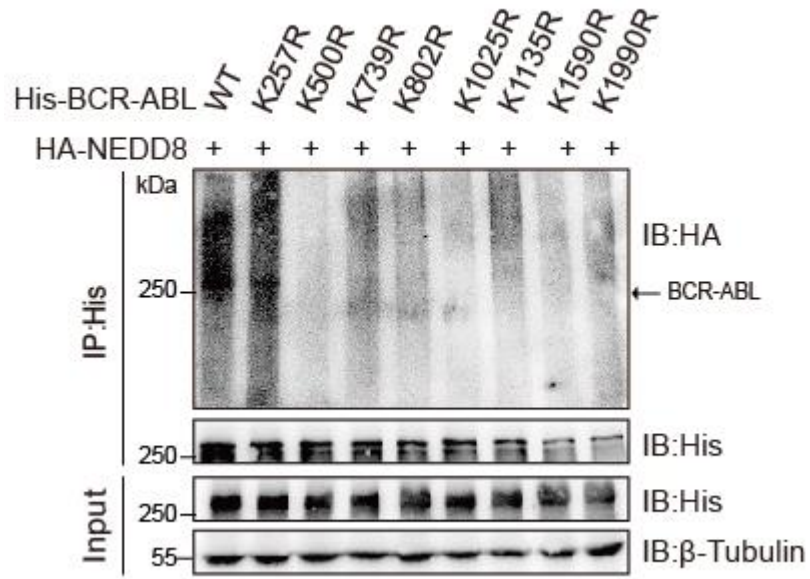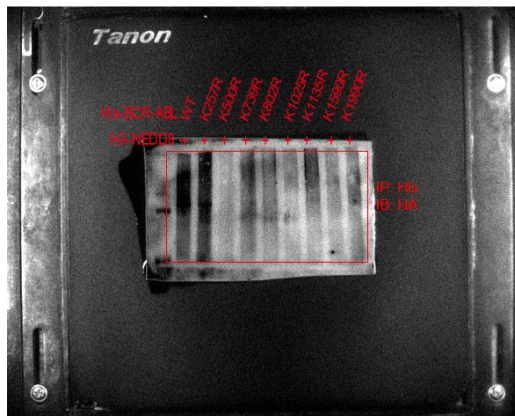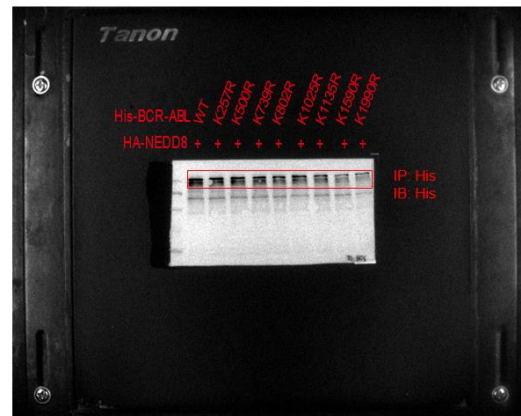

Input

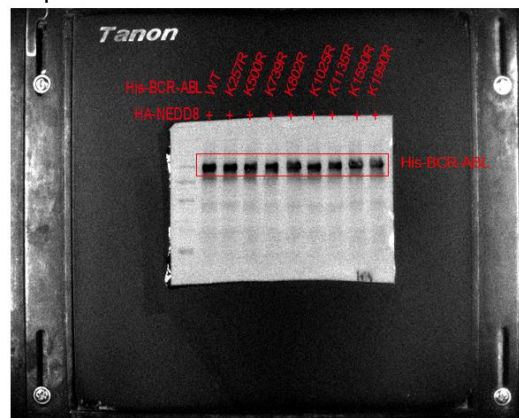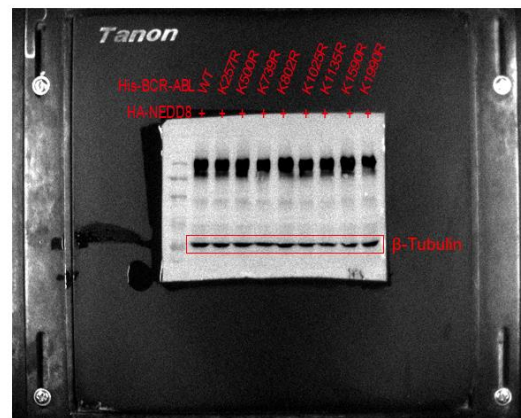

Supplement: Figure 2—source data 28. [file elife-88375-fig2-data28.zip › Figure 2-source data 28/Figure 2-source data 28.pdf]

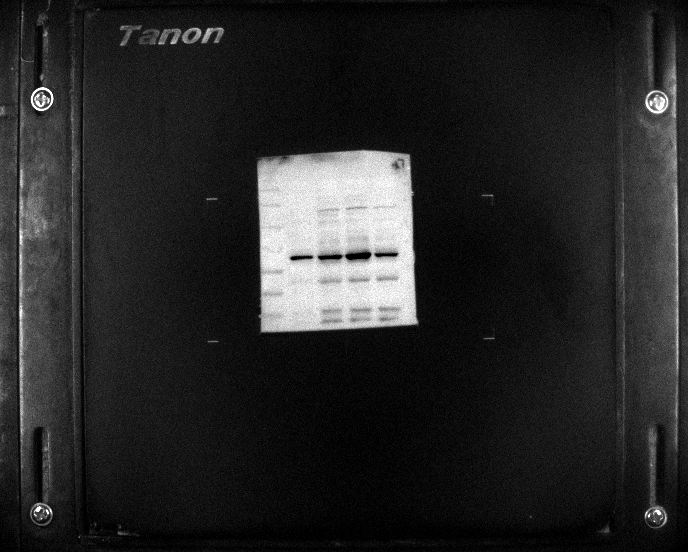

Supplement: Figure 2—figure supplement 1—source data 1. [file elife-88375-fig2-figsupp1-data1.zip › Figure supplement 2-source data 1/AChR a7-2.tif]

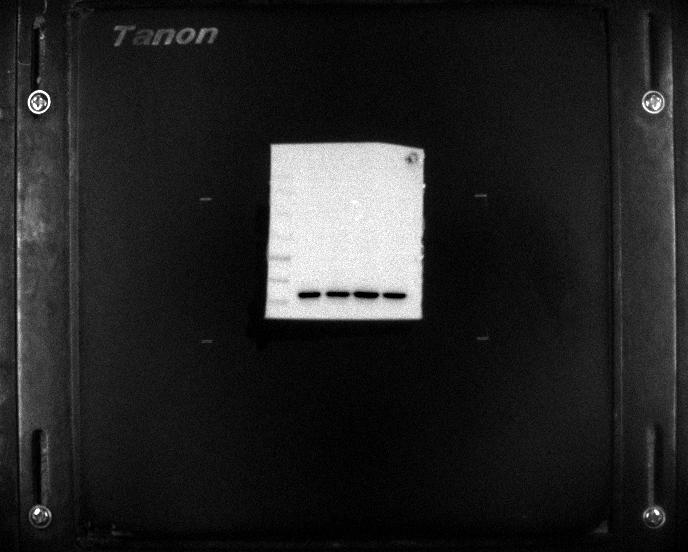

Supplement: Figure 2—figure supplement 1—source data 1. [file elife-88375-fig2-figsupp1-data1.zip › Figure supplement 2-source data 1/AChR a7-GAPDH.tif]

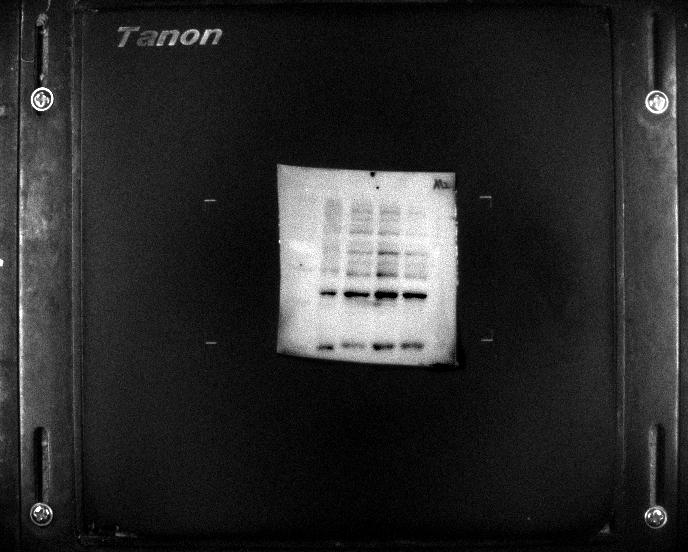

Supplement: Figure 2—figure supplement 1—source data 1. [file elife-88375-fig2-figsupp1-data1.zip › Figure supplement 2-source data 1/AChR M2.tif]

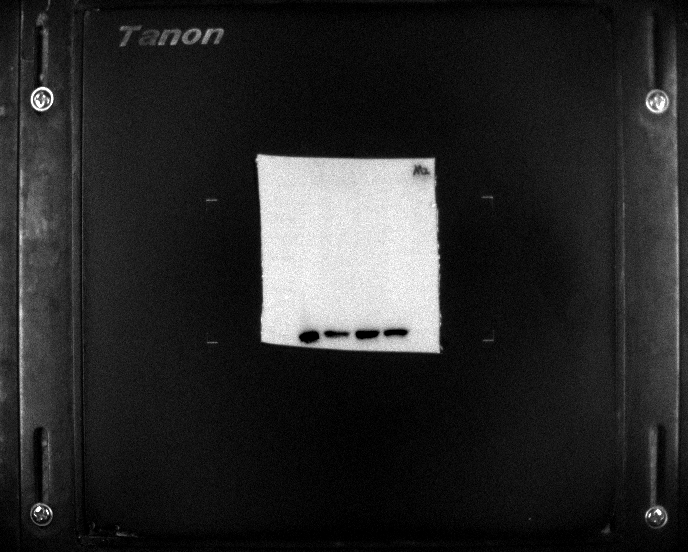

Supplement: Figure 2—figure supplement 1—source data 1. [file elife-88375-fig2-figsupp1-data1.zip › Figure supplement 2-source data 1/AChR M2-GAPDH.tif]

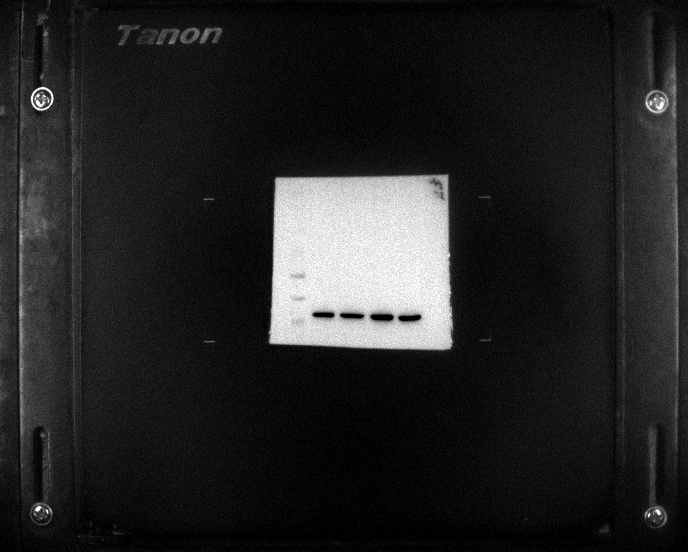

Supplement: Figure 2—figure supplement 1—source data 1. [file elife-88375-fig2-figsupp1-data1.zip › Figure supplement 2-source data 1/AChR M3- GAPDH.tif]

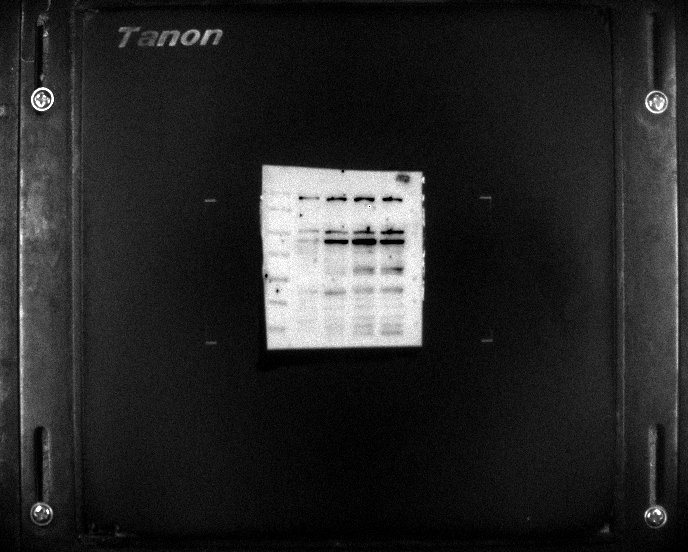

Supplement: Figure 2—figure supplement 1—source data 1. [file elife-88375-fig2-figsupp1-data1.zip › Figure supplement 2-source data 1/AChR M3.tif]

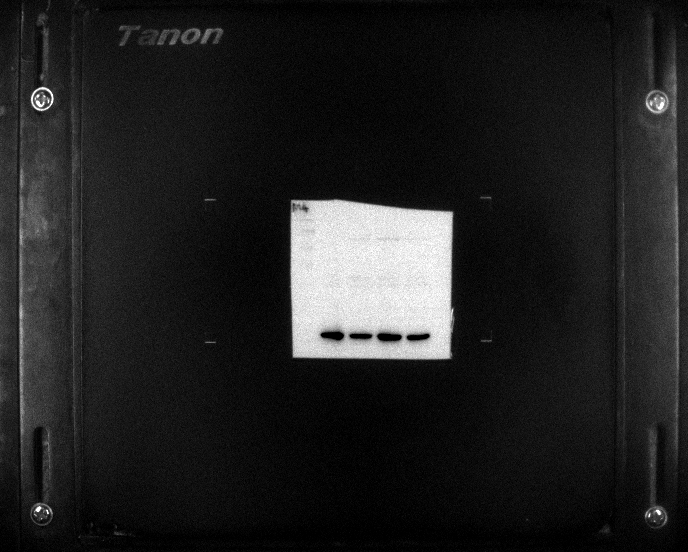

Supplement: Figure 2—figure supplement 1—source data 1. [file elife-88375-fig2-figsupp1-data1.zip › Figure supplement 2-source data 1/AChR M4 GAPDH.tif]

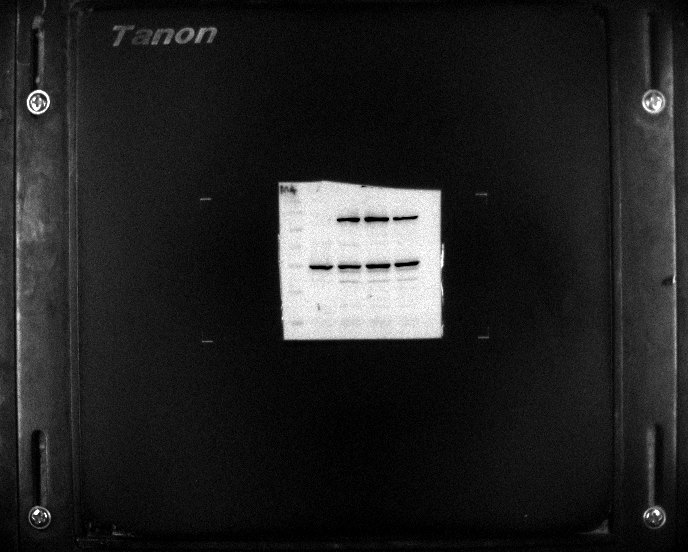

Supplement: Figure 2—figure supplement 1—source data 1. [file elife-88375-fig2-figsupp1-data1.zip › Figure supplement 2-source data 1/AChR M4.tif]

A

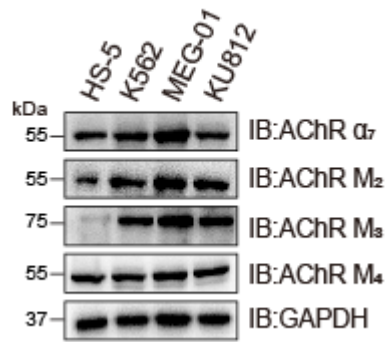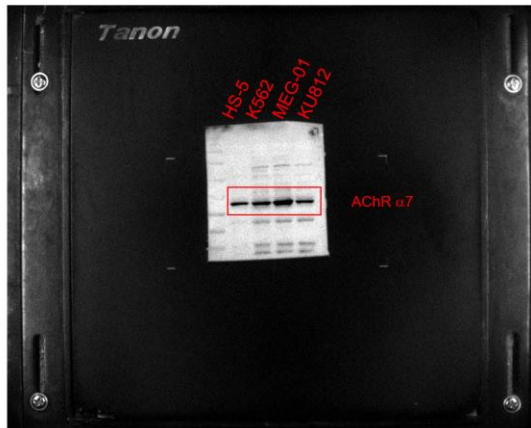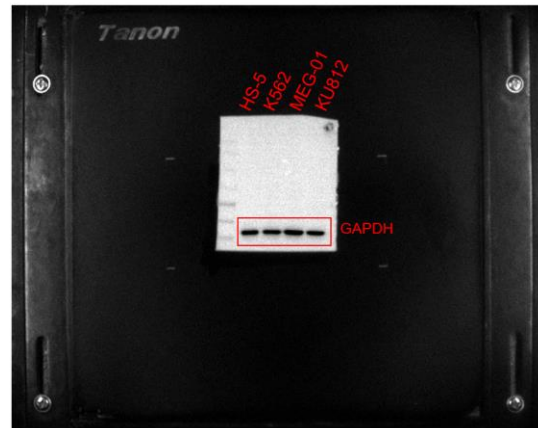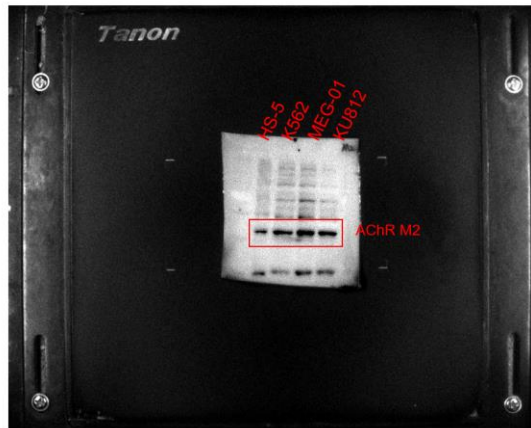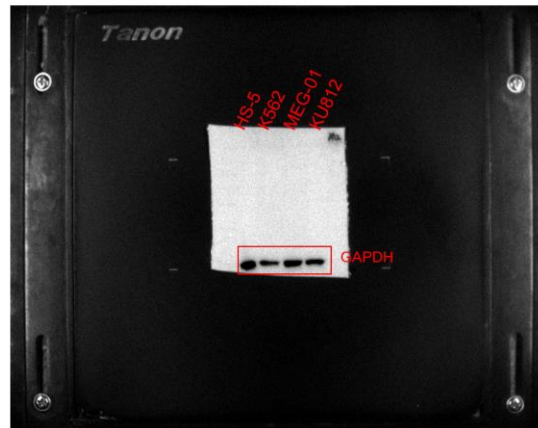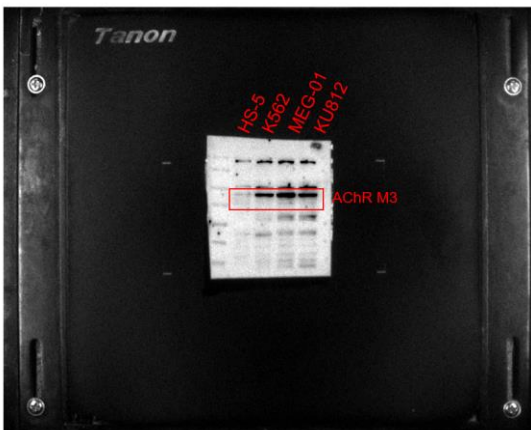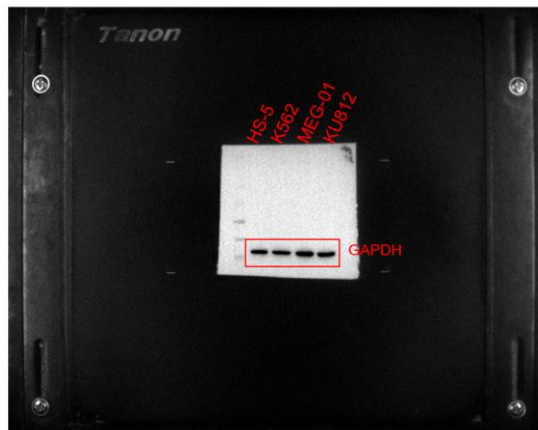

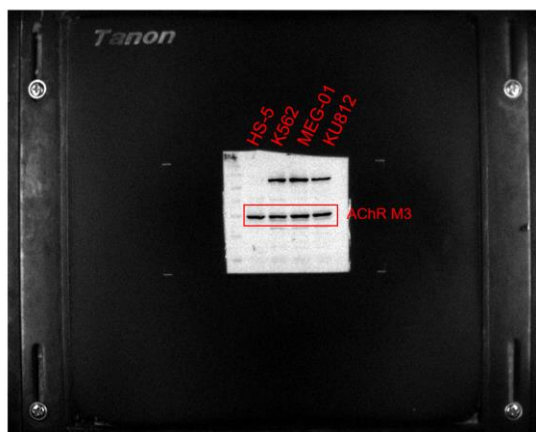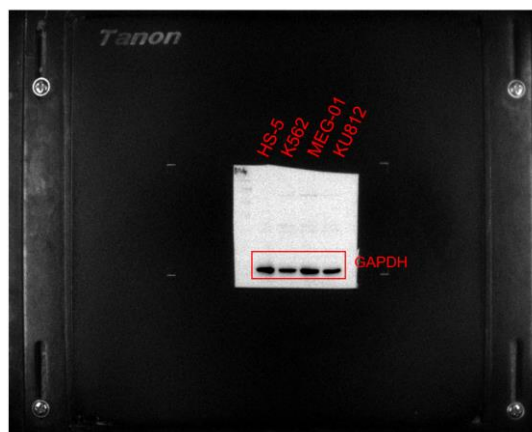

Supplement: Figure 2—figure supplement 1—source data 2. [file elife-88375-fig2-figsupp1-data2.zip › Figure supplement 2-source data 2/Figure supplement 2-source data 2.pdf]

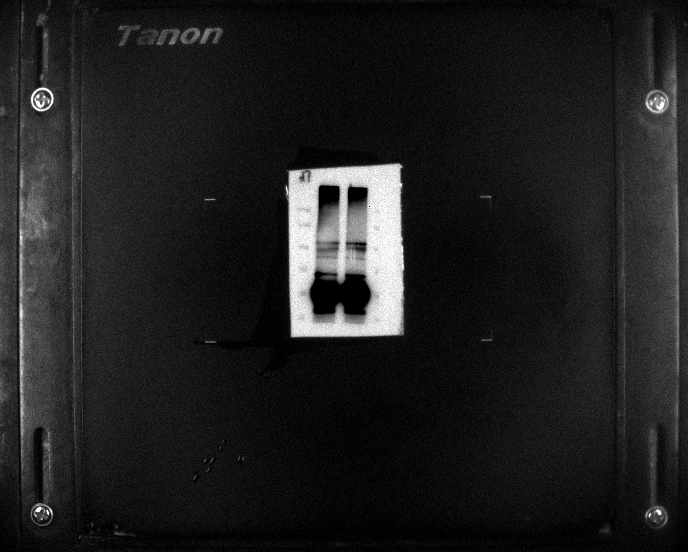

Supplement: Figure 2—figure supplement 1—source data 3. [file elife-88375-fig2-figsupp1-data3.zip › Figure supplement 2-source data 3/AChR a7 IP NEDD8-IB AChR a7.tif]

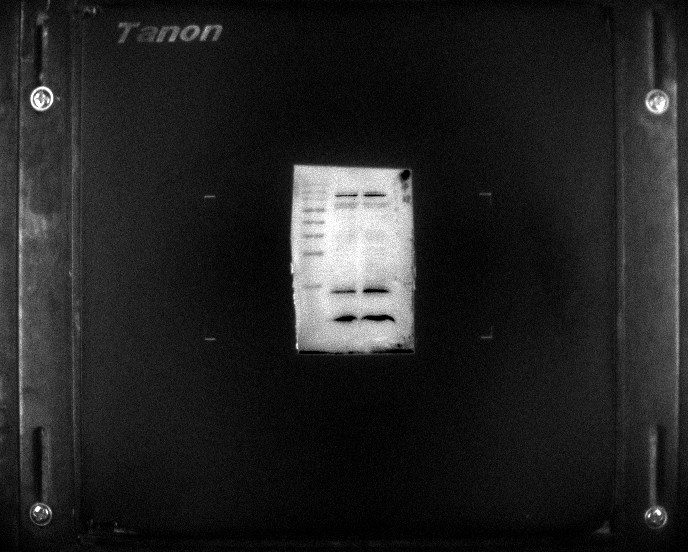

Supplement: Figure 2—figure supplement 1—source data 3. [file elife-88375-fig2-figsupp1-data3.zip › Figure supplement 2-source data 3/AChR a7 IP NEDD8-IB NEDD8.tif]

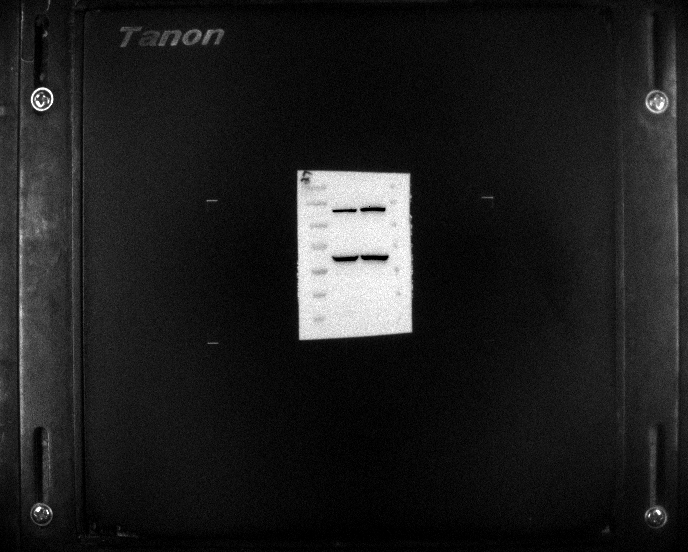

Supplement: Figure 2—figure supplement 1—source data 3. [file elife-88375-fig2-figsupp1-data3.zip › Figure supplement 2-source data 3/AChR a7-Input AChR a7.tif]

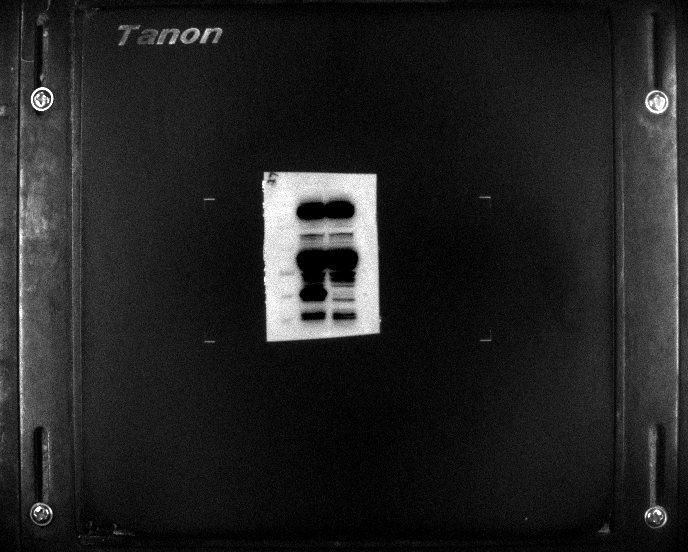

Supplement: Figure 2—figure supplement 1—source data 3. [file elife-88375-fig2-figsupp1-data3.zip › Figure supplement 2-source data 3/AChR a7-Input GAPDH.tif]

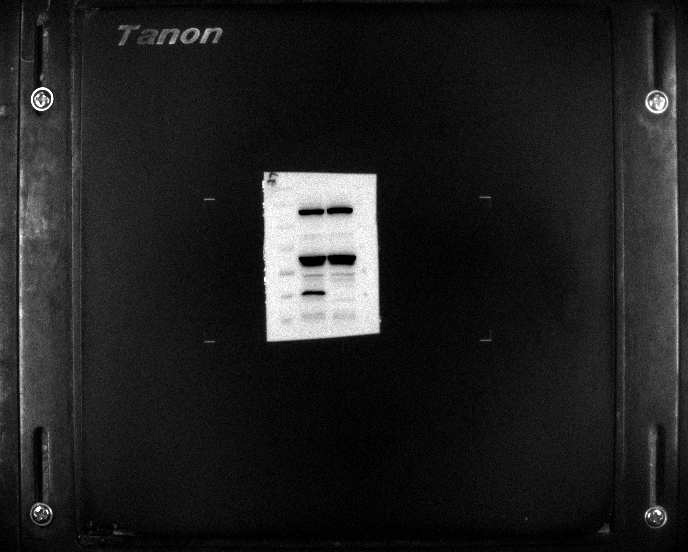

Supplement: Figure 2—figure supplement 1—source data 3. [file elife-88375-fig2-figsupp1-data3.zip › Figure supplement 2-source data 3/AChR a7-Input RAPSYN.tif]

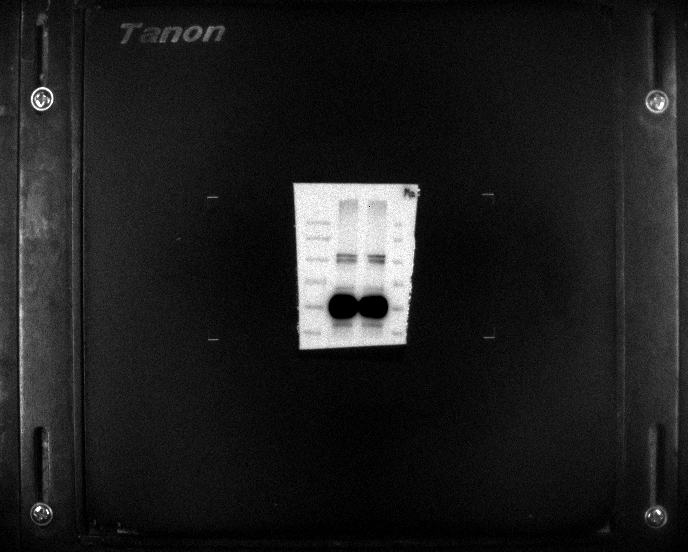

Supplement: Figure 2—figure supplement 1—source data 3. [file elife-88375-fig2-figsupp1-data3.zip › Figure supplement 2-source data 3/AChR M2 IP NEDD8-IB AChR M2.tif]

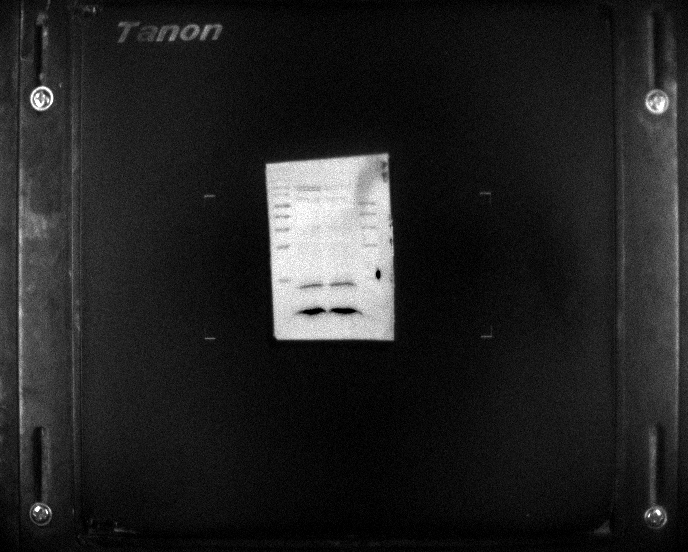

Supplement: Figure 2—figure supplement 1—source data 3. [file elife-88375-fig2-figsupp1-data3.zip › Figure supplement 2-source data 3/AChR M2 IP NEDD8-IB NEDD8.tif]

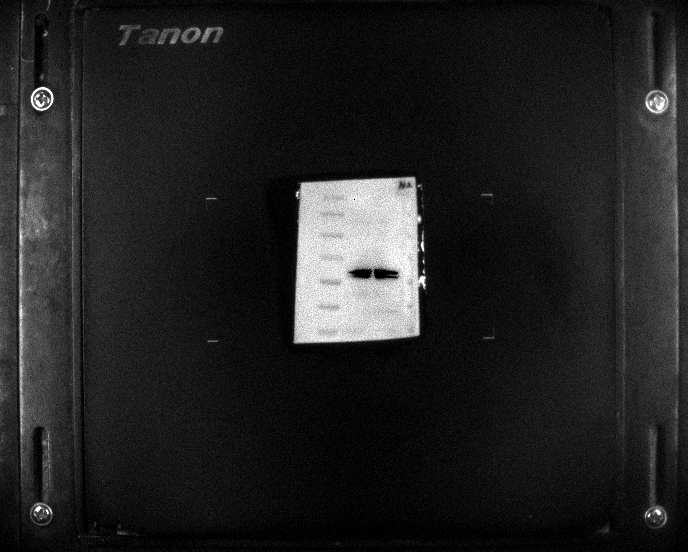

Supplement: Figure 2—figure supplement 1—source data 3. [file elife-88375-fig2-figsupp1-data3.zip › Figure supplement 2-source data 3/AChR M2-Input AChR M2.tif]

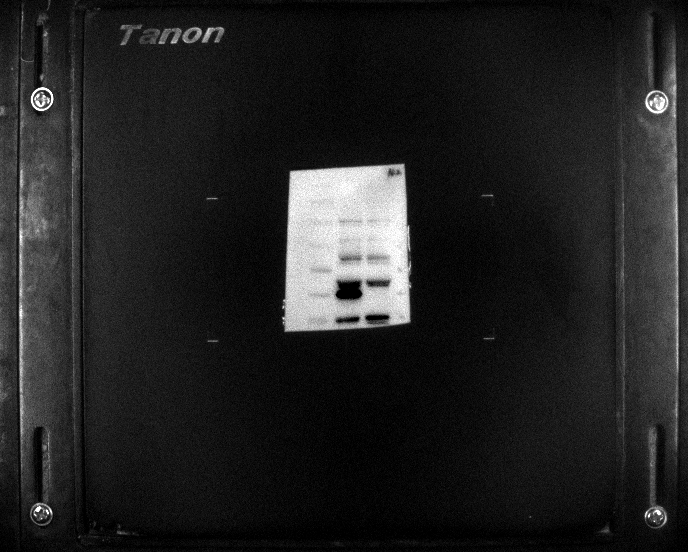

Supplement: Figure 2—figure supplement 1—source data 3. [file elife-88375-fig2-figsupp1-data3.zip › Figure supplement 2-source data 3/AChR M2-Input GAPDH.tif]

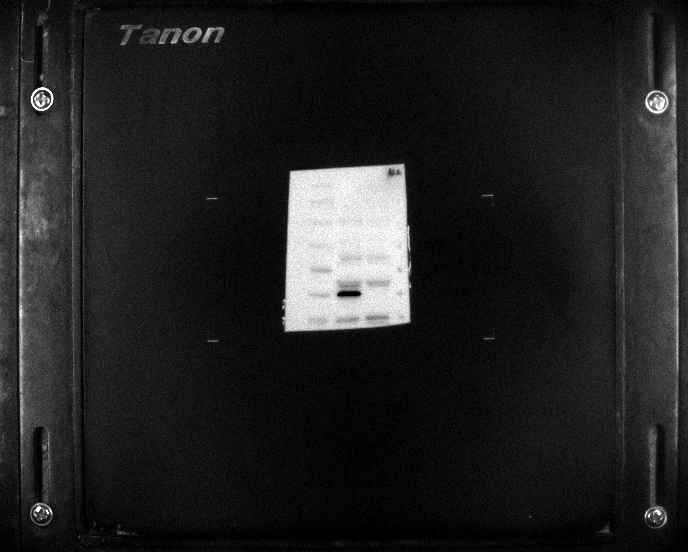

Supplement: Figure 2—figure supplement 1—source data 3. [file elife-88375-fig2-figsupp1-data3.zip › Figure supplement 2-source data 3/AChR M2-Input RAPSYN.tif]

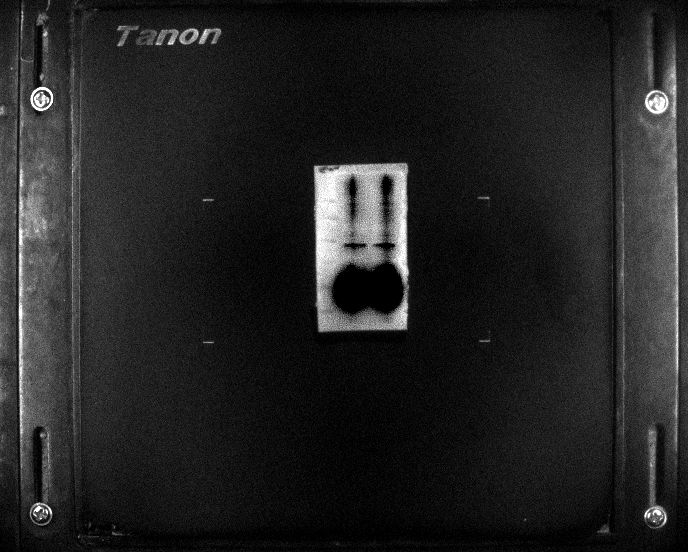

Supplement: Figure 2—figure supplement 1—source data 3. [file elife-88375-fig2-figsupp1-data3.zip › Figure supplement 2-source data 3/AChR M3 IP NEDD8-IB AChR M3.tif]

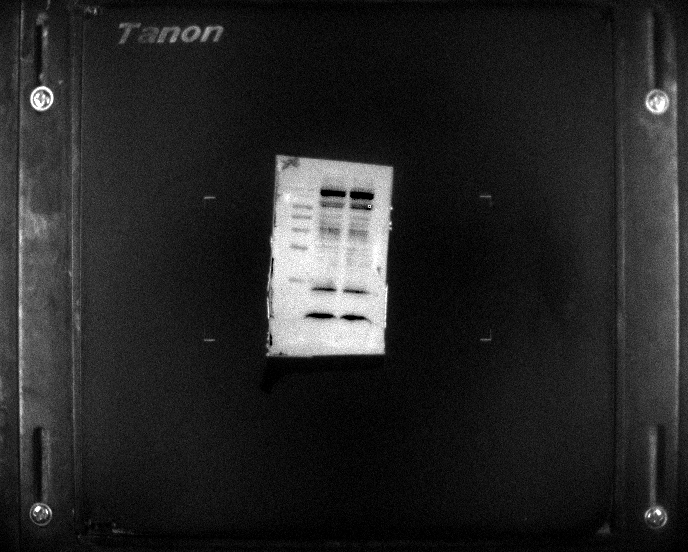

Supplement: Figure 2—figure supplement 1—source data 3. [file elife-88375-fig2-figsupp1-data3.zip › Figure supplement 2-source data 3/AChR M3 IP NEDD8-IB NEDD8.tif]

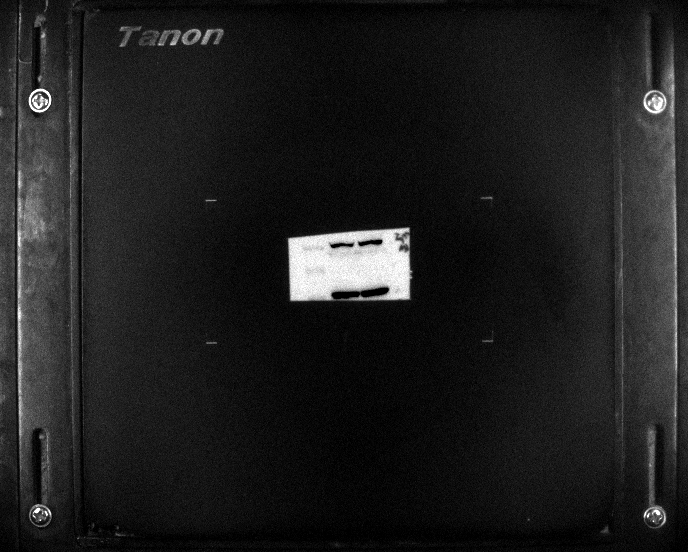

Supplement: Figure 2—figure supplement 1—source data 3. [file elife-88375-fig2-figsupp1-data3.zip › Figure supplement 2-source data 3/AChR M3-Input AChR M3.tif]

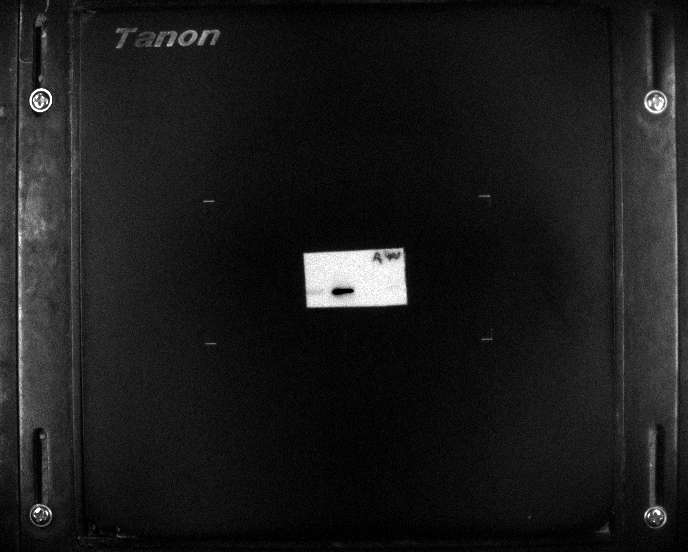

Supplement: Figure 2—figure supplement 1—source data 3. [file elife-88375-fig2-figsupp1-data3.zip › Figure supplement 2-source data 3/AChR M3-Input RAPSYN.tif]

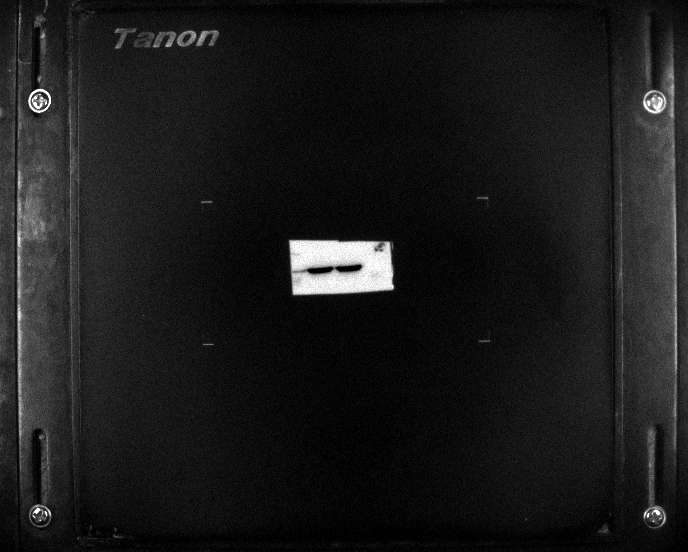

Supplement: Figure 2—figure supplement 1—source data 3. [file elife-88375-fig2-figsupp1-data3.zip › Figure supplement 2-source data 3/AChR M3-Input GAPDH.tif]

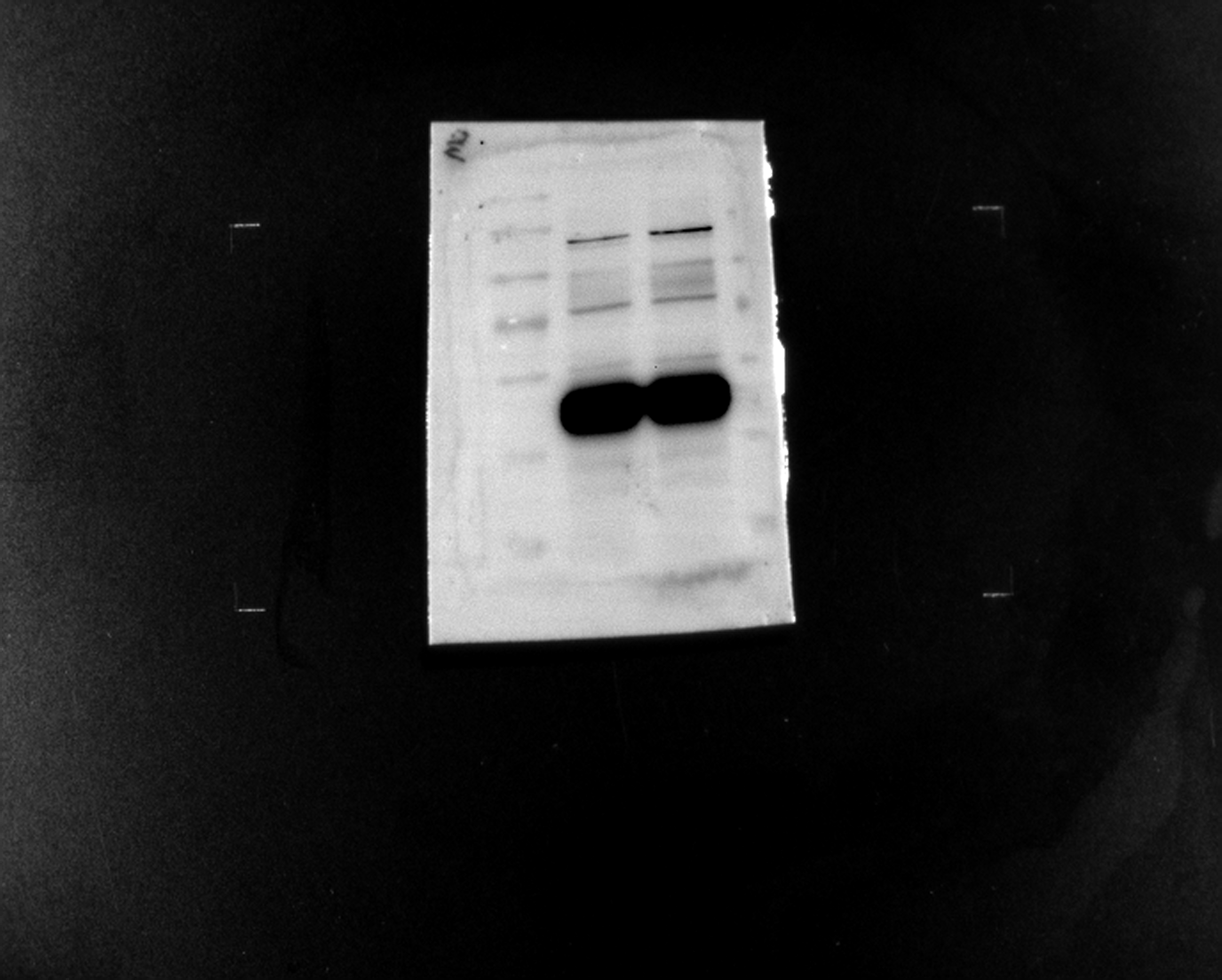

Supplement: Figure 2—figure supplement 1—source data 3. [file elife-88375-fig2-figsupp1-data3.zip › Figure supplement 2-source data 3/AChR M4 IP NEDD8-IB AChR M4.tif]

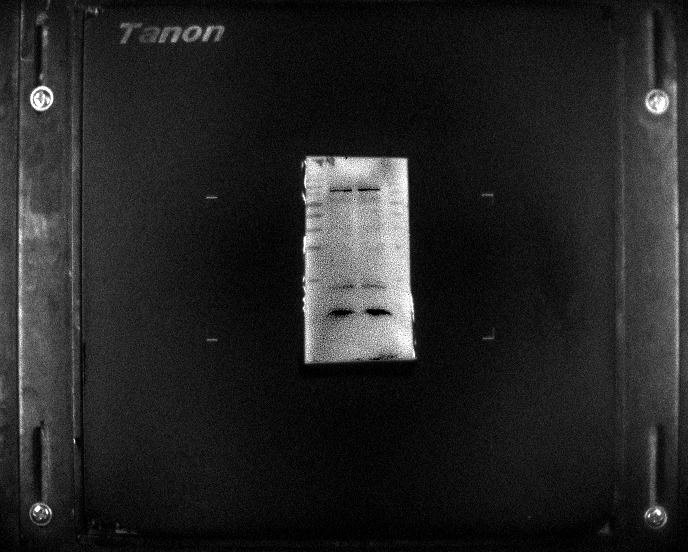

Supplement: Figure 2—figure supplement 1—source data 3. [file elife-88375-fig2-figsupp1-data3.zip › Figure supplement 2-source data 3/AChR M4 IP NEDD8-IB NEDD8.tif]

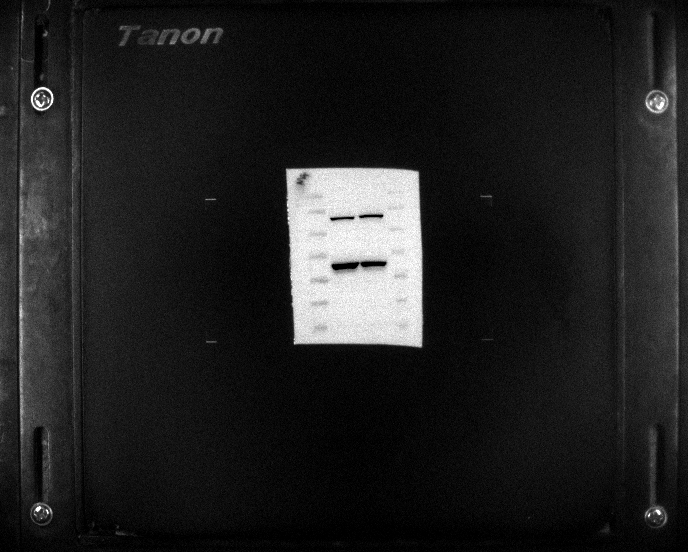

Supplement: Figure 2—figure supplement 1—source data 3. [file elife-88375-fig2-figsupp1-data3.zip › Figure supplement 2-source data 3/AChR M4-Input AChR M4.tif]

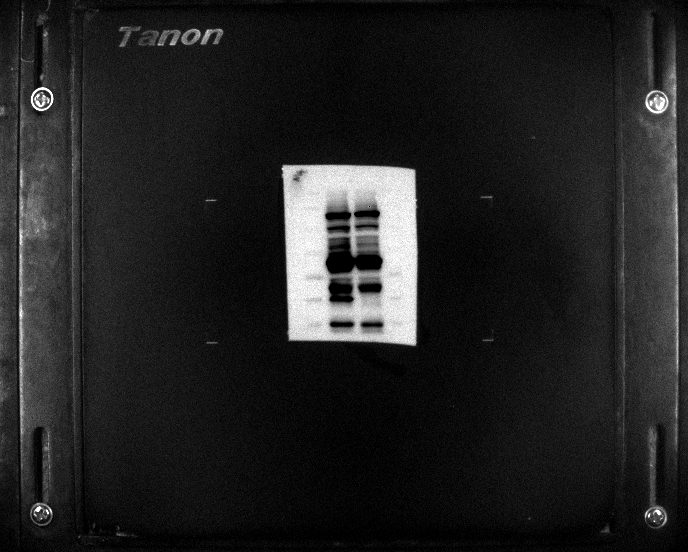

Supplement: Figure 2—figure supplement 1—source data 3. [file elife-88375-fig2-figsupp1-data3.zip › Figure supplement 2-source data 3/AChR M4-Input GAPDH.tif]

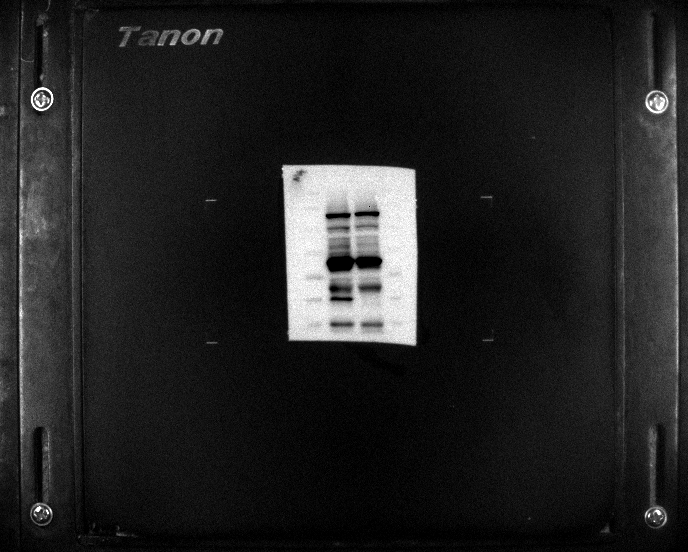

Supplement: Figure 2—figure supplement 1—source data 3. [file elife-88375-fig2-figsupp1-data3.zip › Figure supplement 2-source data 3/AChR M4-Input RAPSYN.tif]

B

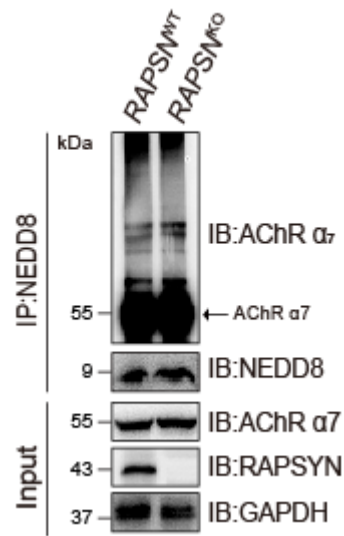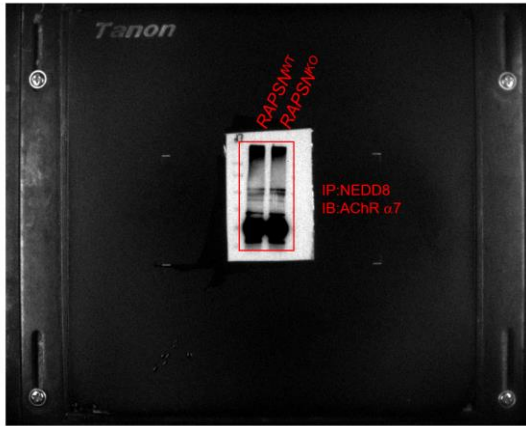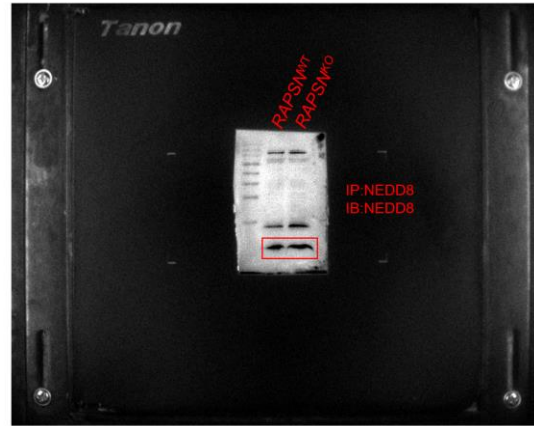

Input

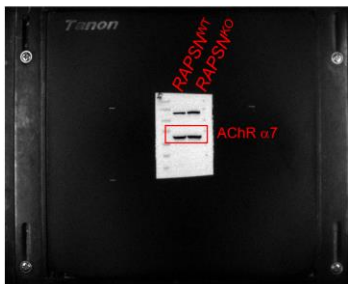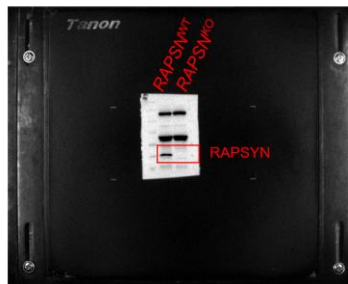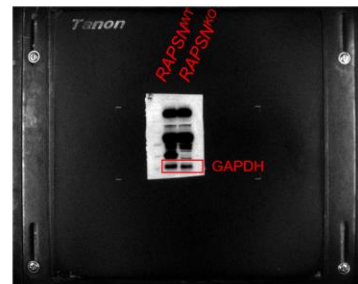

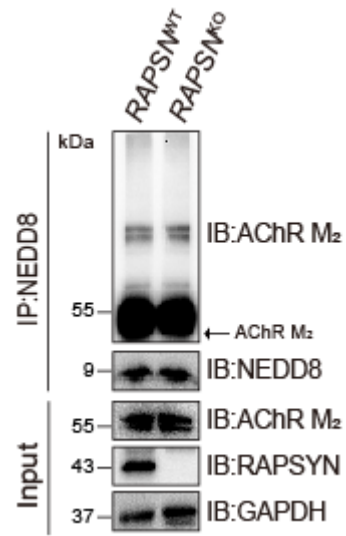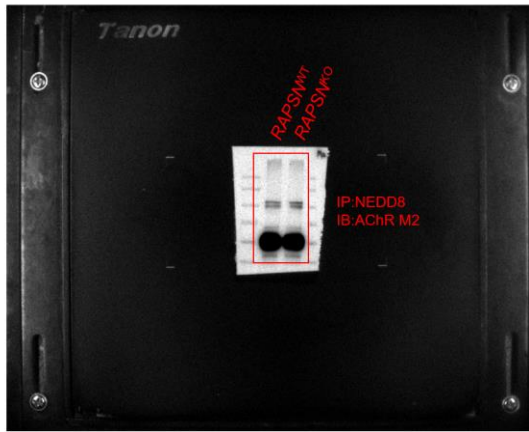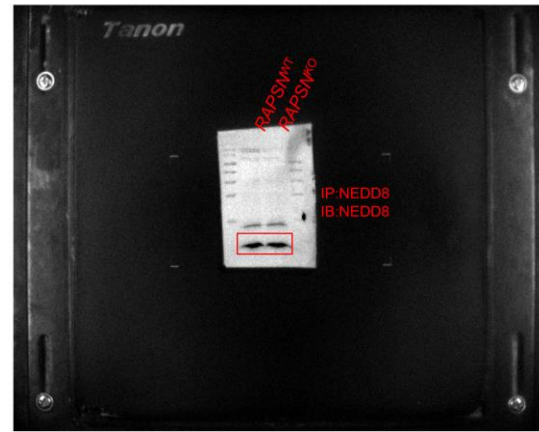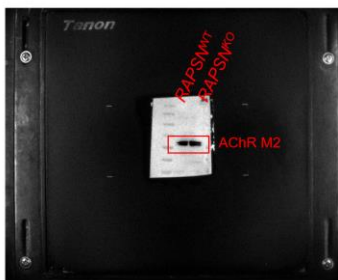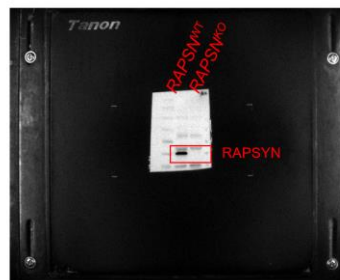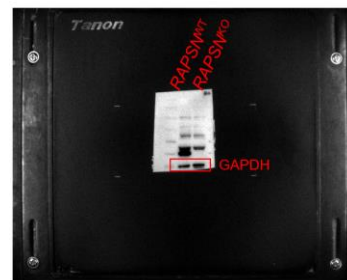

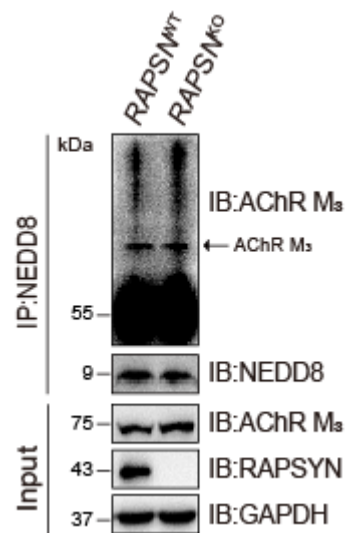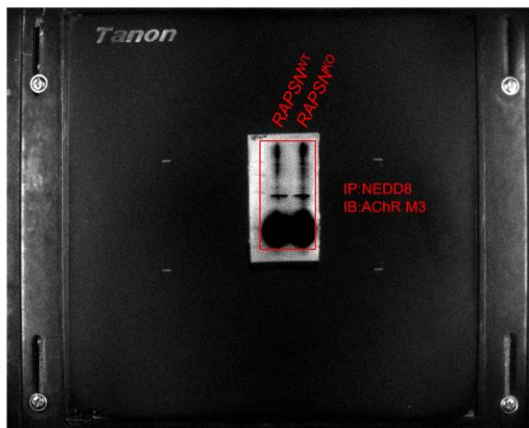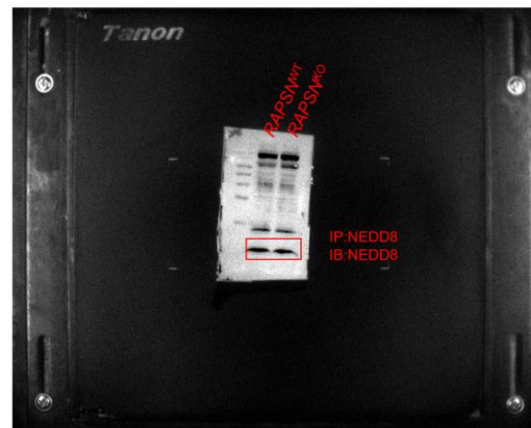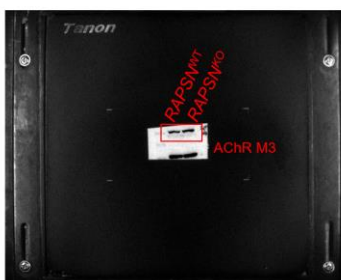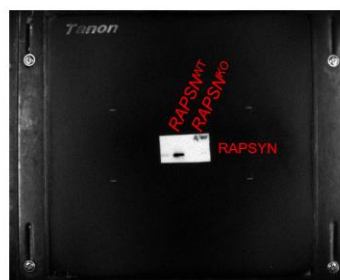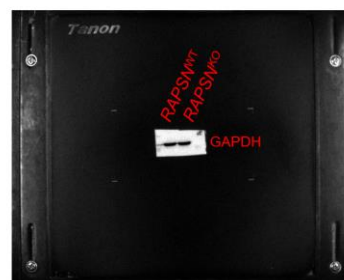

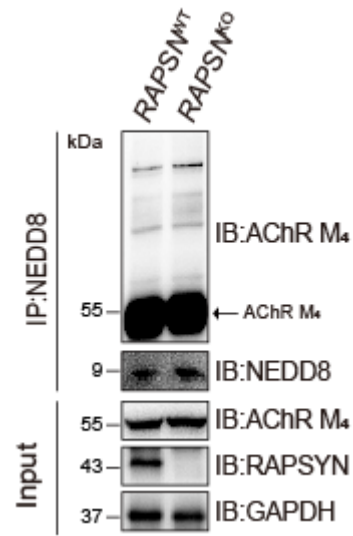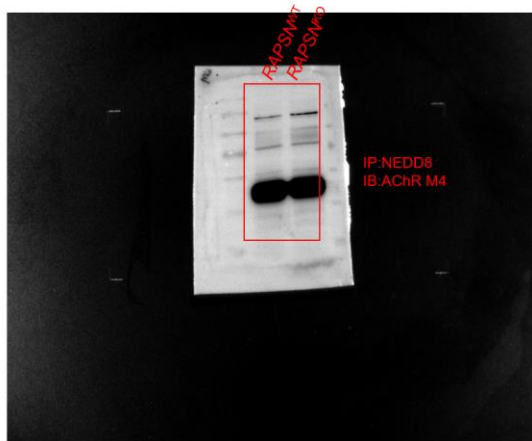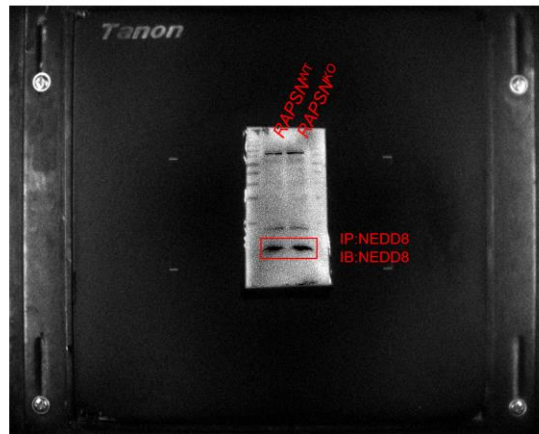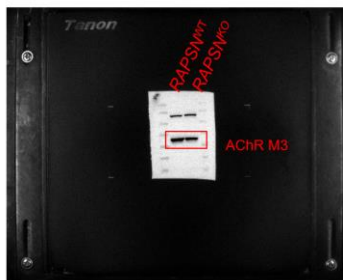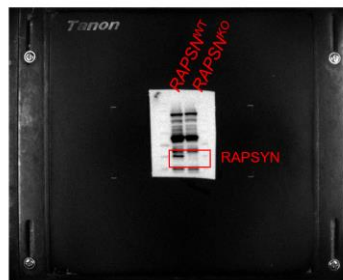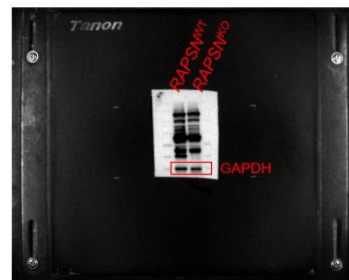

Supplement: Figure 2—figure supplement 1—source data 4. [file elife-88375-fig2-figsupp1-data4.zip › Figure supplement 2-source data 4/Figure supplement 2-source data 4.pdf]

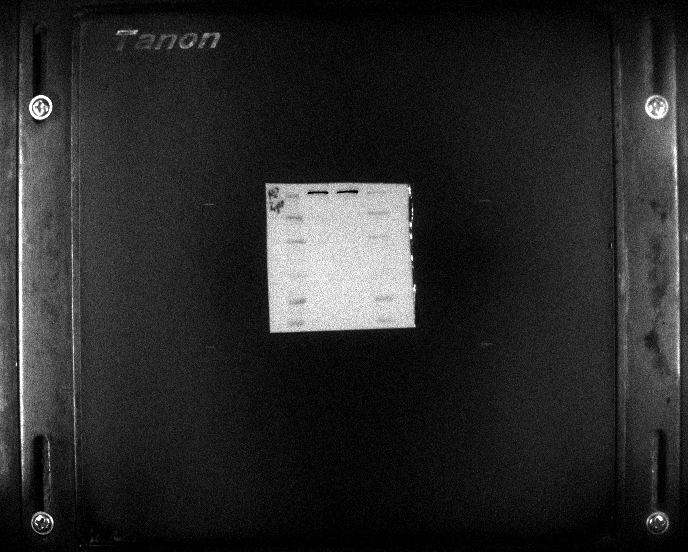

Supplement: Figure 2—figure supplement 1—source data 5. [file elife-88375-fig2-figsupp1-data5.zip › Figure supplement 2-source data 5/Input BCR-ABL.tif]

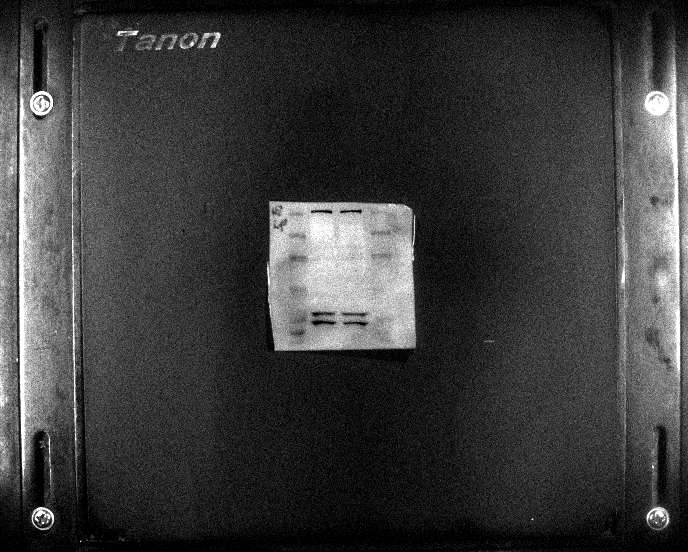

Supplement: Figure 2—figure supplement 1—source data 5. [file elife-88375-fig2-figsupp1-data5.zip › Figure supplement 2-source data 5/Input RAPSYN.tif]

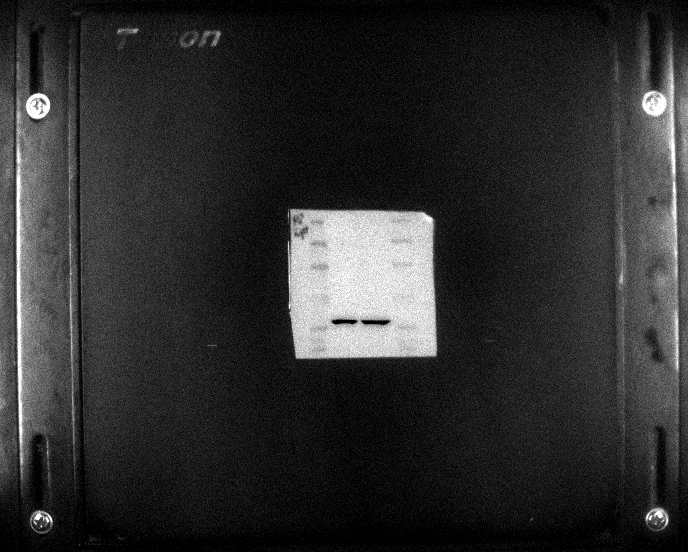

Supplement: Figure 2—figure supplement 1—source data 5. [file elife-88375-fig2-figsupp1-data5.zip › Figure supplement 2-source data 5/Input a┬-Tubulin.tif]

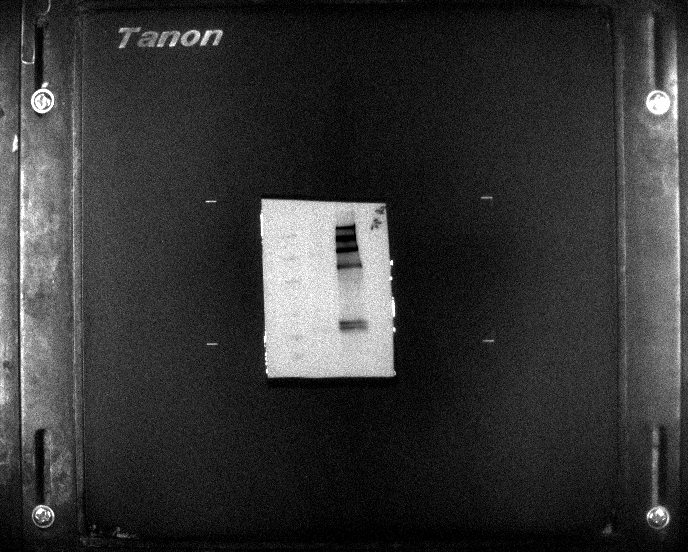

Supplement: Figure 2—figure supplement 1—source data 5. [file elife-88375-fig2-figsupp1-data5.zip › Figure supplement 2-source data 5/IP BCR-ABL-IB BCR-ABL.tif]

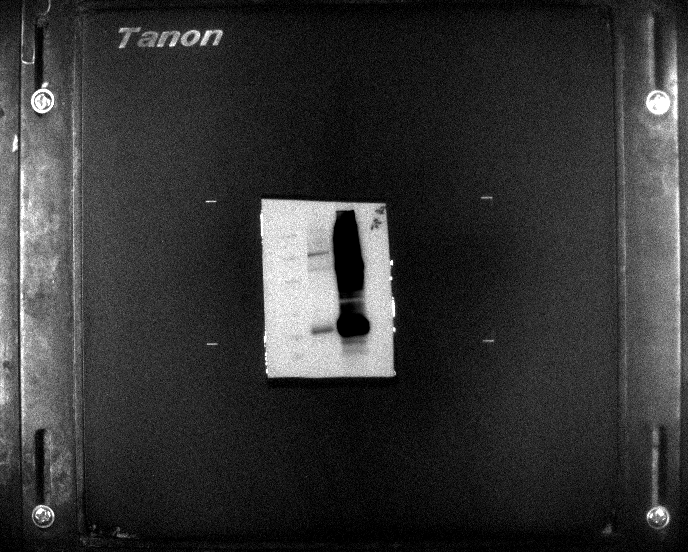

Supplement: Figure 2—figure supplement 1—source data 5. [file elife-88375-fig2-figsupp1-data5.zip › Figure supplement 2-source data 5/IP BCR-ABL-IB RAPSYN.tif]

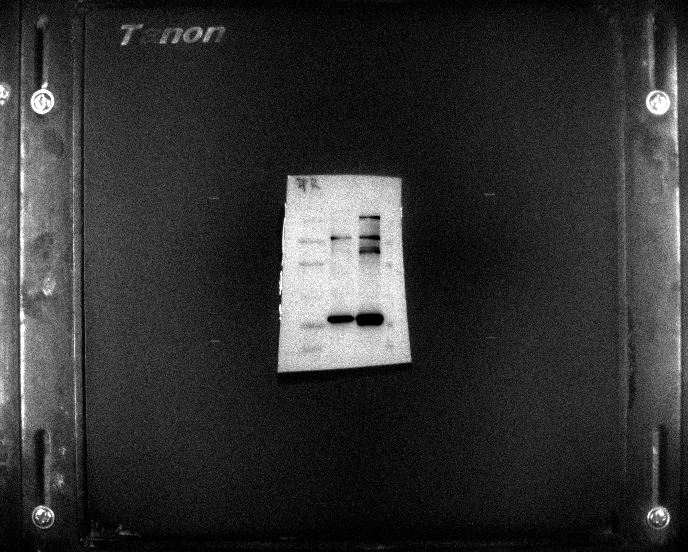

Supplement: Figure 2—figure supplement 1—source data 5. [file elife-88375-fig2-figsupp1-data5.zip › Figure supplement 2-source data 5/IP RAPSYN-IB BCR-ABL.tif]

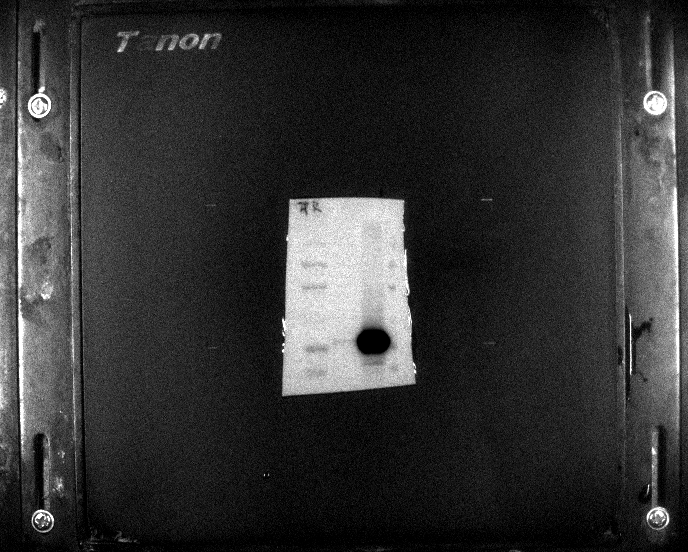

Supplement: Figure 2—figure supplement 1—source data 5. [file elife-88375-fig2-figsupp1-data5.zip › Figure supplement 2-source data 5/IP RAPSYN-IB RAPSY.tif]

D

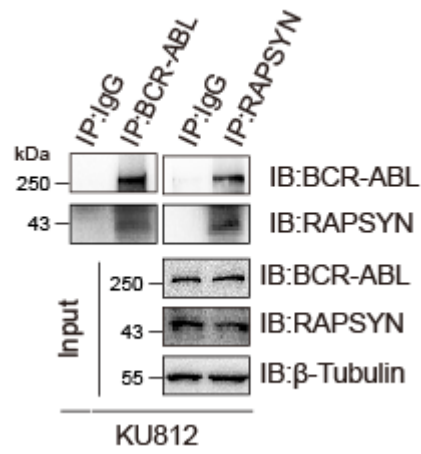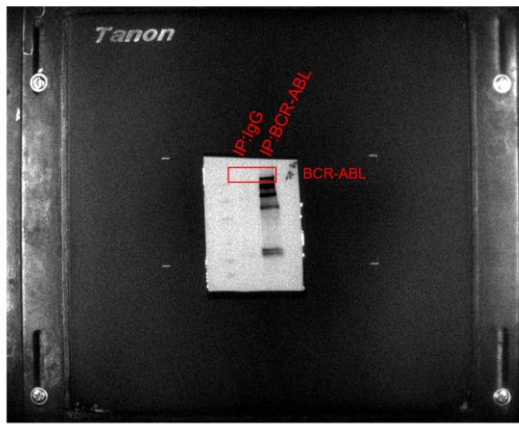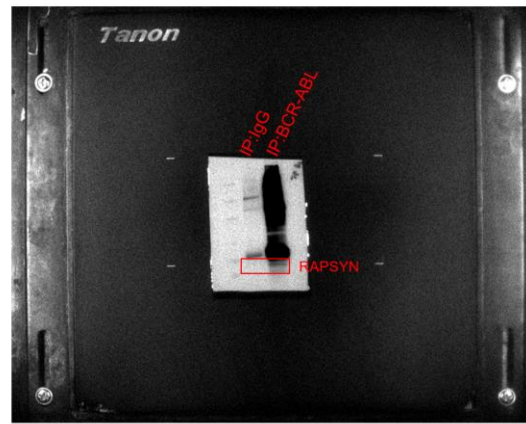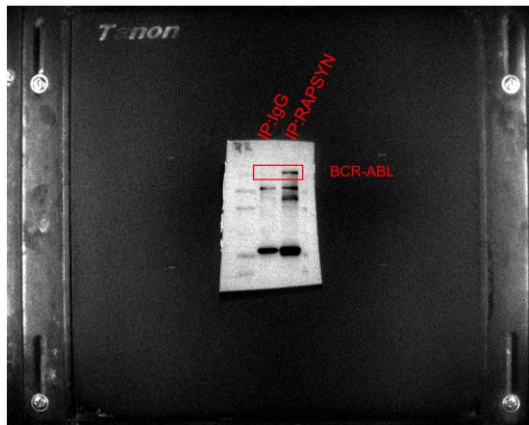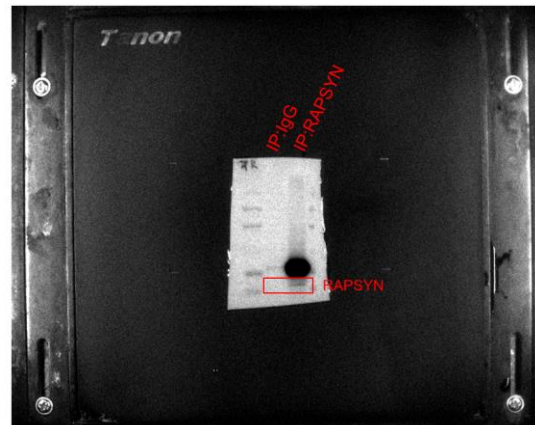

Input

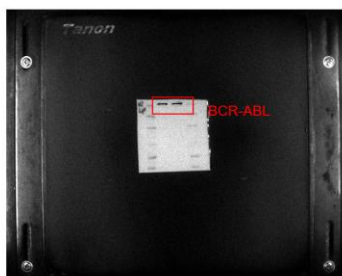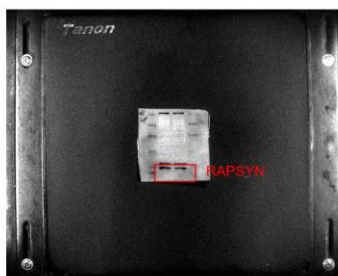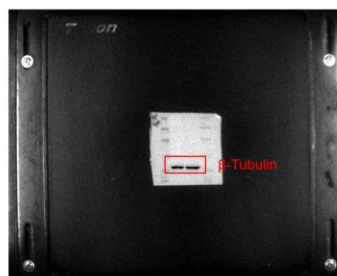

Supplement: Figure 2—figure supplement 1—source data 6. [file elife-88375-fig2-figsupp1-data6.zip › Figure supplement 2-source data 6/Figure supplement 2-source data 6.pdf]

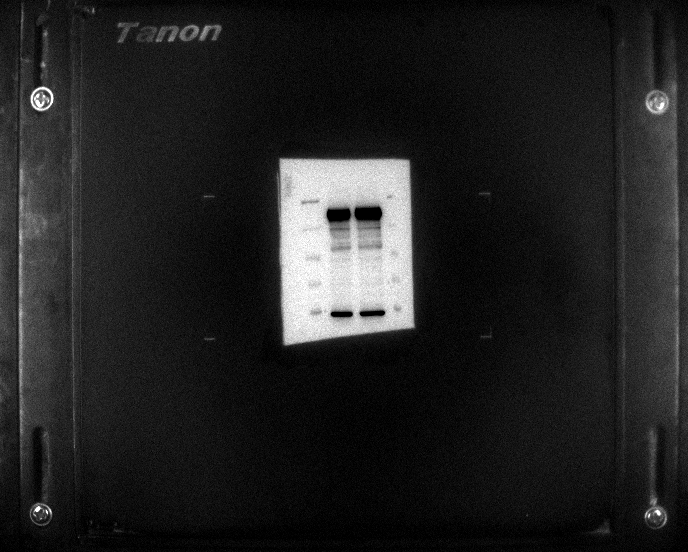

Supplement: Figure 2—figure supplement 1—source data 7. [file elife-88375-fig2-figsupp1-data7.zip › Figure supplement 2-source data 7/Jurkat Input a┬-Tubulin.tif]

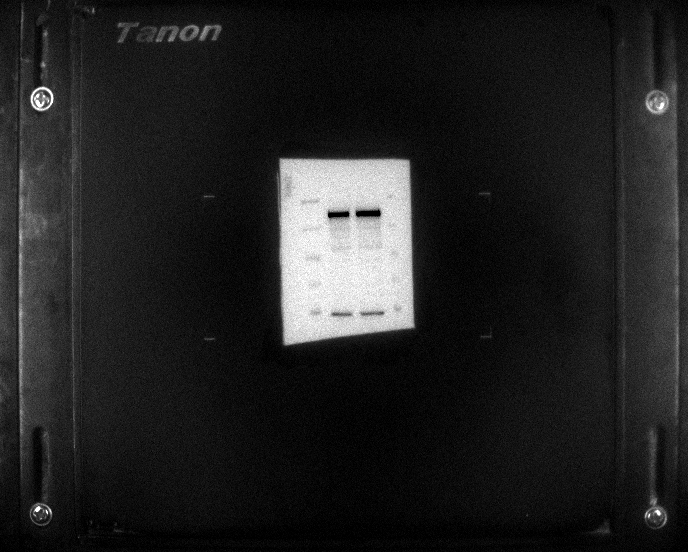

Supplement: Figure 2—figure supplement 1—source data 7. [file elife-88375-fig2-figsupp1-data7.zip › Figure supplement 2-source data 7/Jurkat Input BCR-ABL.tif]

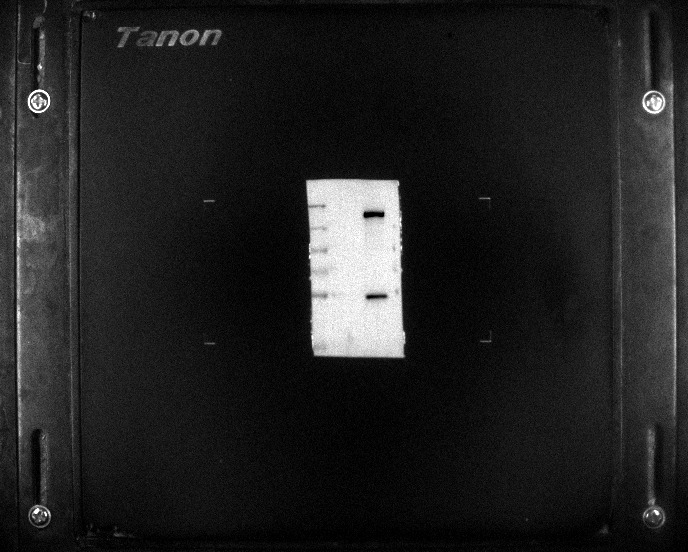

Supplement: Figure 2—figure supplement 1—source data 7. [file elife-88375-fig2-figsupp1-data7.zip › Figure supplement 2-source data 7/Jurkat IP BCR-ABL-IB BCR-ABL.tif]

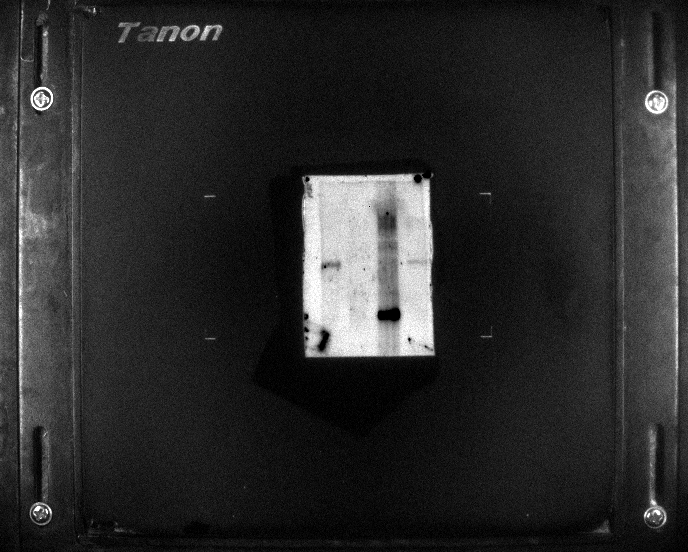

Supplement: Figure 2—figure supplement 1—source data 7. [file elife-88375-fig2-figsupp1-data7.zip › Figure supplement 2-source data 7/Jurkat IP BCR-ABL-IB NEDD8.tif]

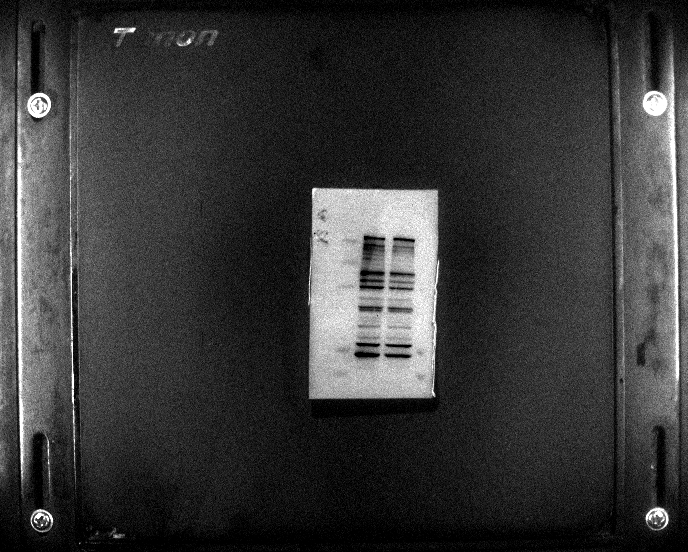

Supplement: Figure 2—figure supplement 1—source data 7. [file elife-88375-fig2-figsupp1-data7.zip › Figure supplement 2-source data 7/KU812 Input BCR-ABL.tif]

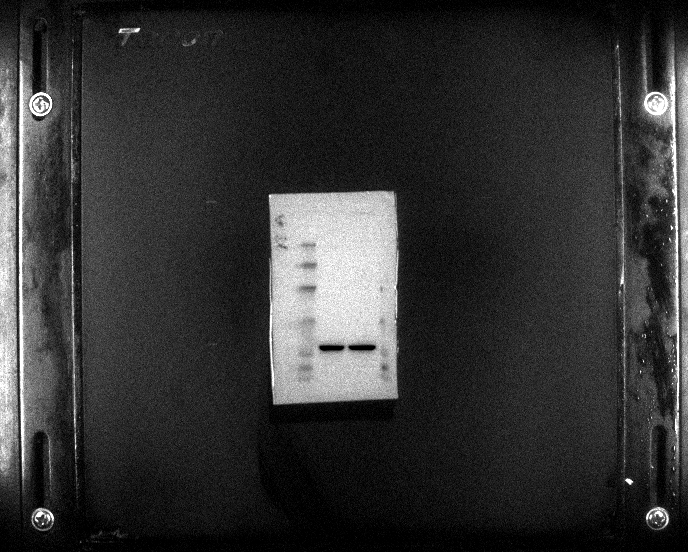

Supplement: Figure 2—figure supplement 1—source data 7. [file elife-88375-fig2-figsupp1-data7.zip › Figure supplement 2-source data 7/KU812 Input a┬-Tubulin.tif]

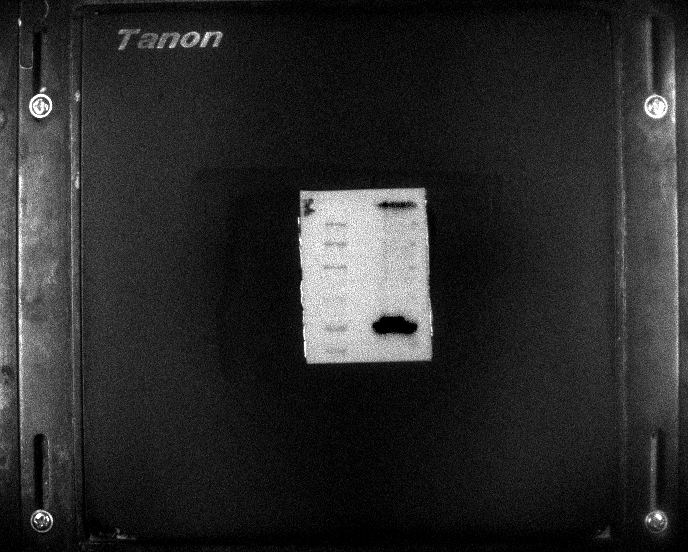

Supplement: Figure 2—figure supplement 1—source data 7. [file elife-88375-fig2-figsupp1-data7.zip › Figure supplement 2-source data 7/KU812 IP BCR-ABL-IB BCR-ABL.tif]

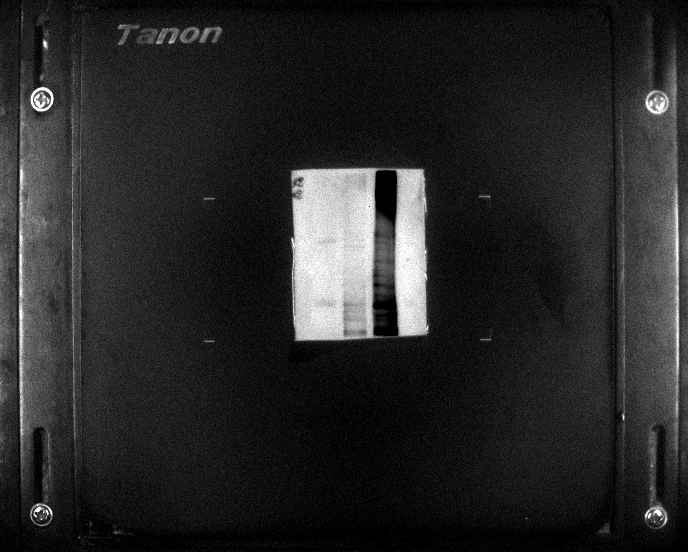

Supplement: Figure 2—figure supplement 1—source data 7. [file elife-88375-fig2-figsupp1-data7.zip › Figure supplement 2-source data 7/KU812 IP BCR-ABL-IB NEDD8.tif]

E

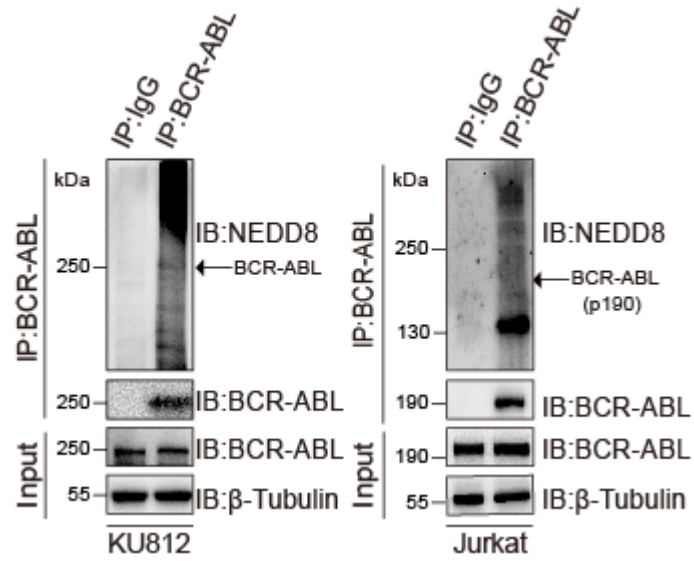

KU812

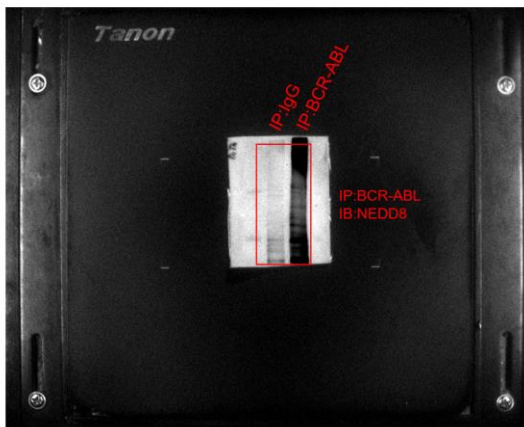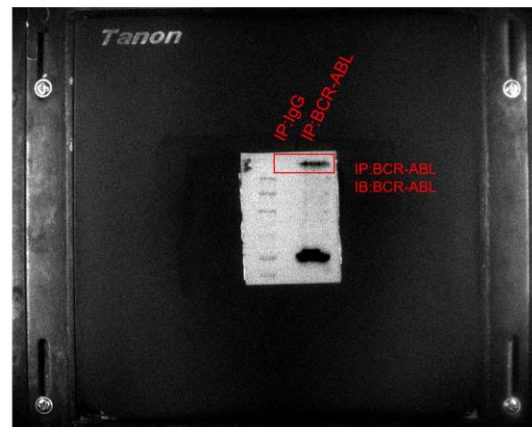

Input

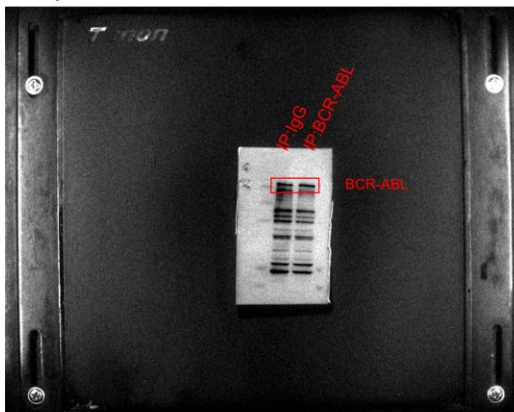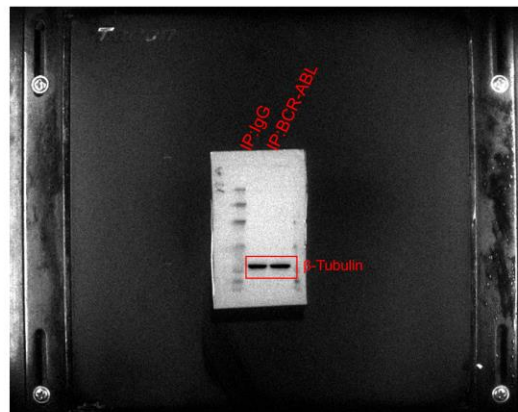

# Jurkat

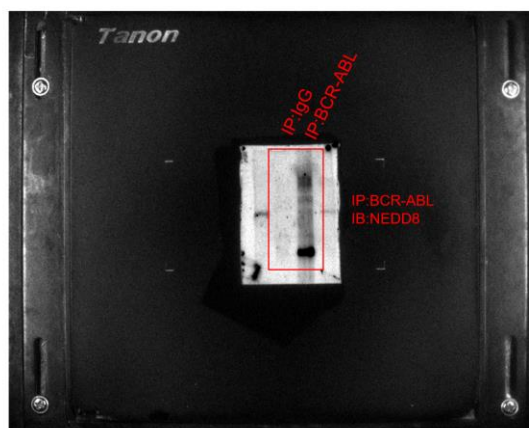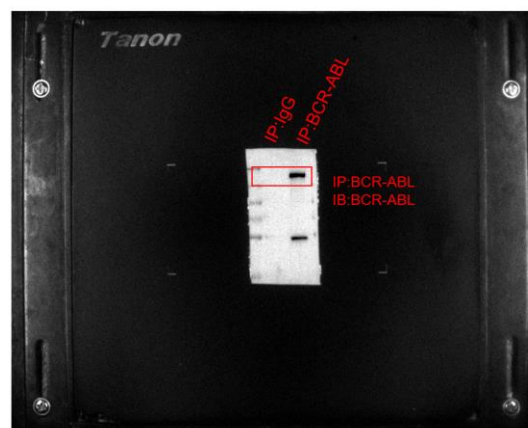

# Input

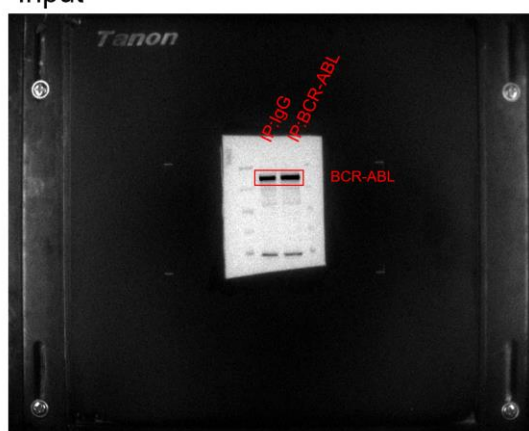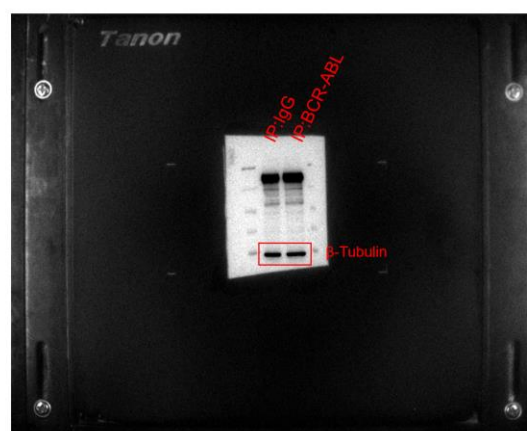

Supplement: Figure 2—figure supplement 1—source data 8. [file elife-88375-fig2-figsupp1-data8.zip › Figure supplement 2-source data 8/Figure supplement 2-source data 8.pdf]

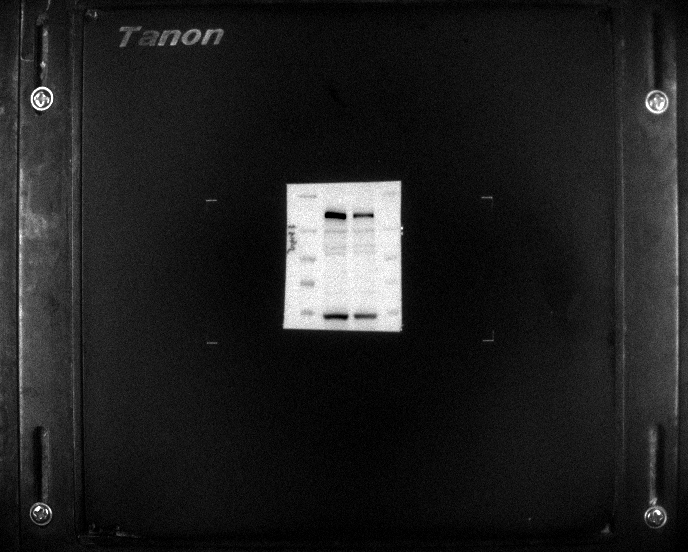

Supplement: Figure 2—figure supplement 1—source data 9. [file elife-88375-fig2-figsupp1-data9.zip › Figure supplement 2-source data 9/Jurkat Input BCR-ABL.tif]

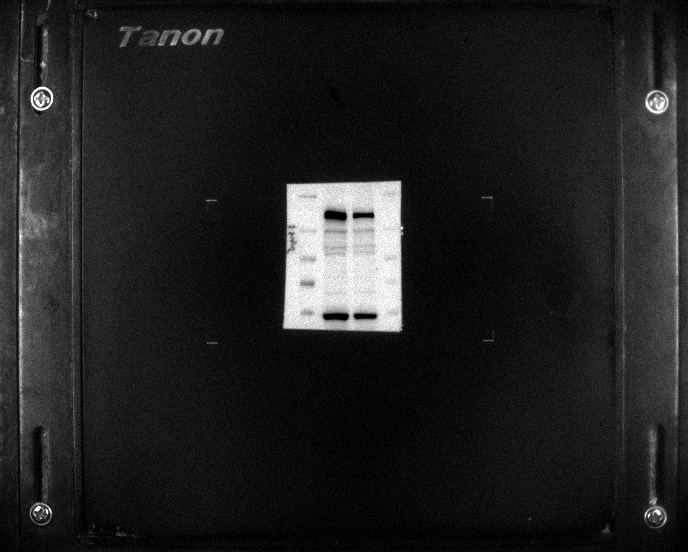

Supplement: Figure 2—figure supplement 1—source data 9. [file elife-88375-fig2-figsupp1-data9.zip › Figure supplement 2-source data 9/Jurkat Input a┬-Tubulin.tif]

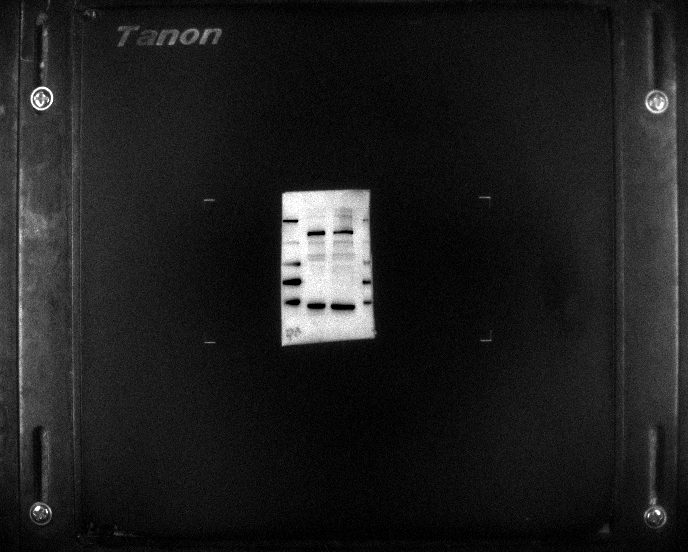

Supplement: Figure 2—figure supplement 1—source data 9. [file elife-88375-fig2-figsupp1-data9.zip › Figure supplement 2-source data 9/Jurkat IP BCR-ABL-IB BCR-ABL.tif]

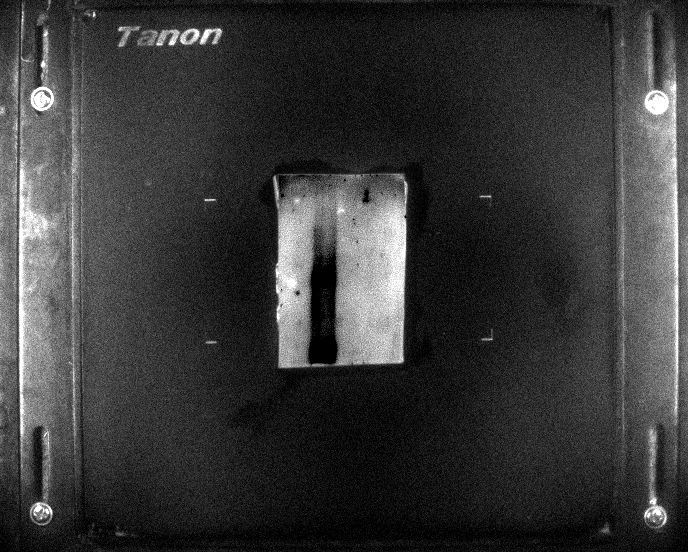

Supplement: Figure 2—figure supplement 1—source data 9. [file elife-88375-fig2-figsupp1-data9.zip › Figure supplement 2-source data 9/Jurkat IP BCR-ABL-IB NEDD8.tif]

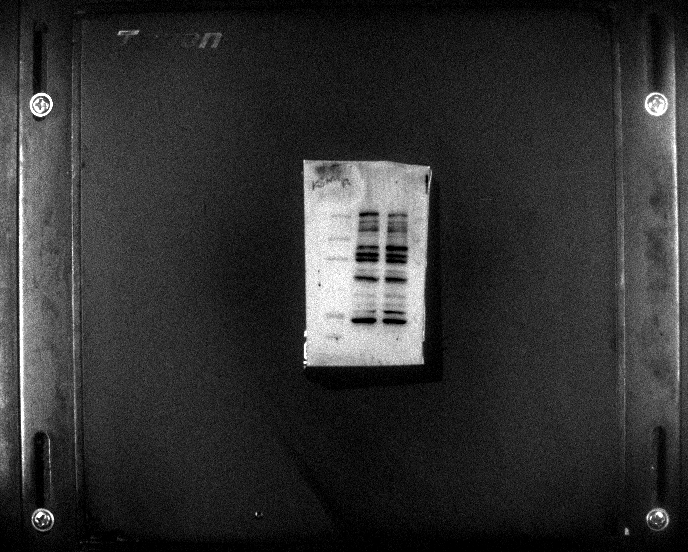

Supplement: Figure 2—figure supplement 1—source data 9. [file elife-88375-fig2-figsupp1-data9.zip › Figure supplement 2-source data 9/KU812 Input BCR-ABL.tif]

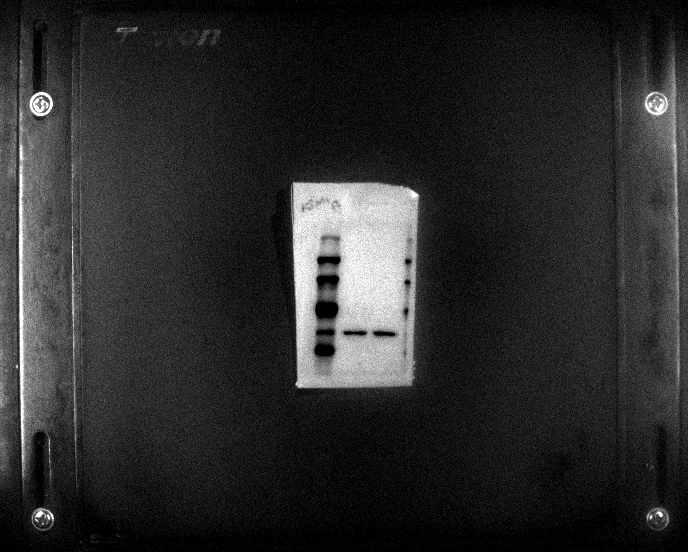

Supplement: Figure 2—figure supplement 1—source data 9. [file elife-88375-fig2-figsupp1-data9.zip › Figure supplement 2-source data 9/KU812 Input a┬-Tubulin.tif]

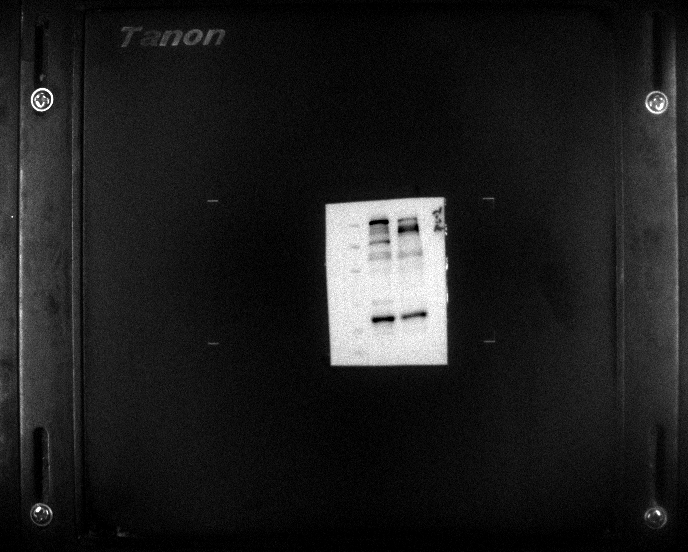

Supplement: Figure 2—figure supplement 1—source data 9. [file elife-88375-fig2-figsupp1-data9.zip › Figure supplement 2-source data 9/KU812 IP BCR-ABL-IB BCR-ABL.tif]

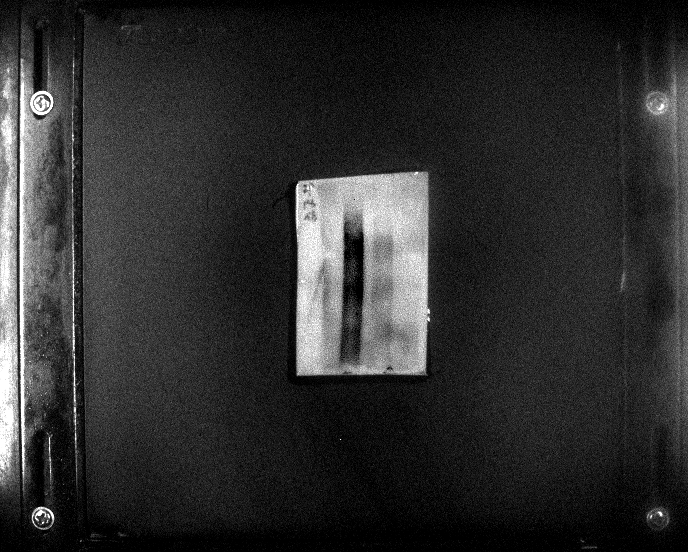

Supplement: Figure 2—figure supplement 1—source data 9. [file elife-88375-fig2-figsupp1-data9.zip › Figure supplement 2-source data 9/KU812 IP BCR-ABL-IB NEDD8.tif]

F

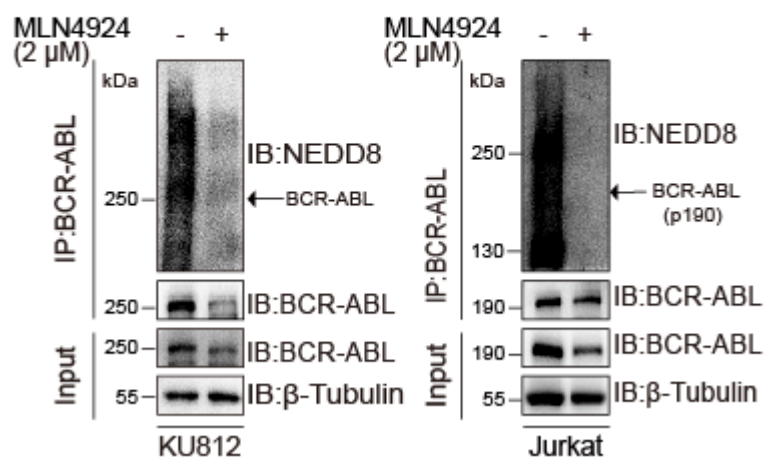

KU812

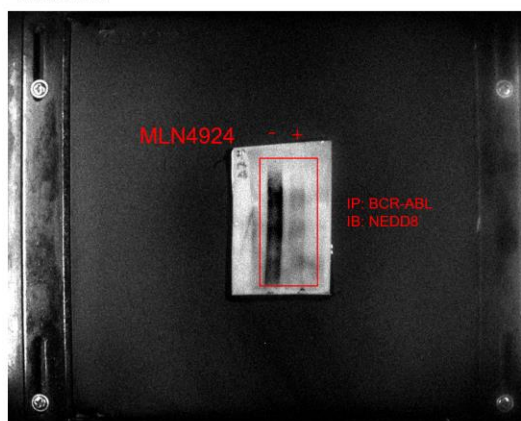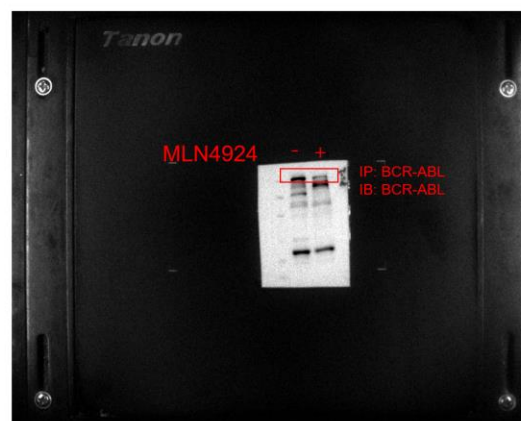

Input

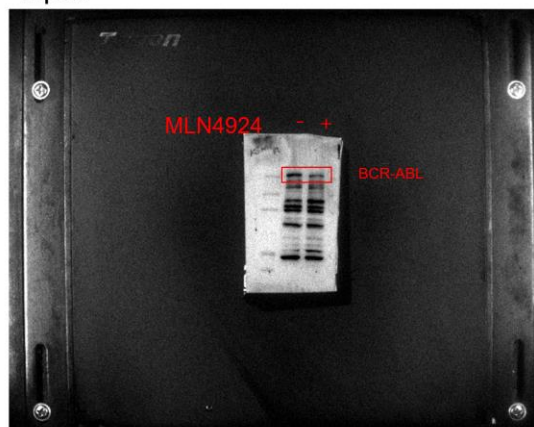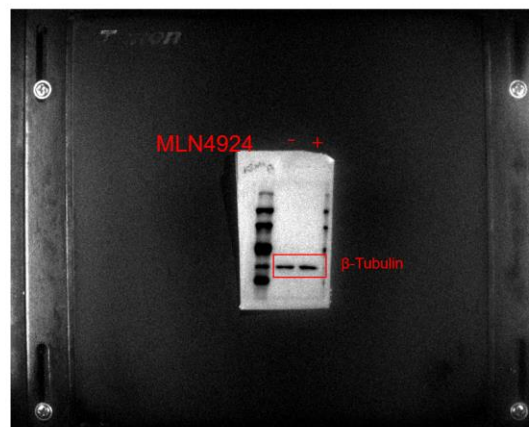

# Jurkat

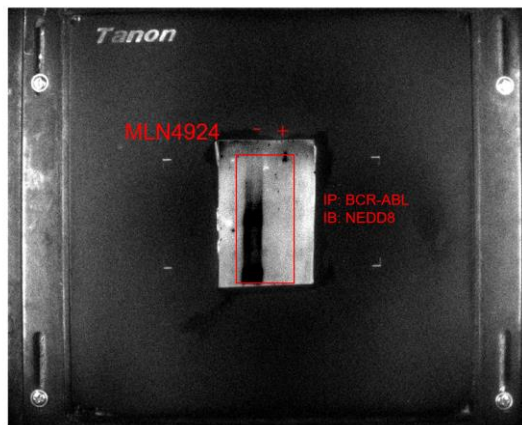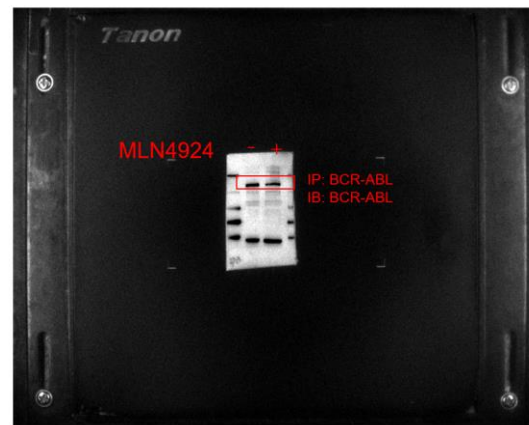

# Input

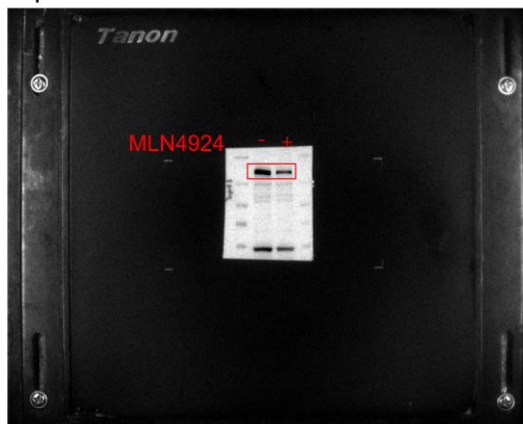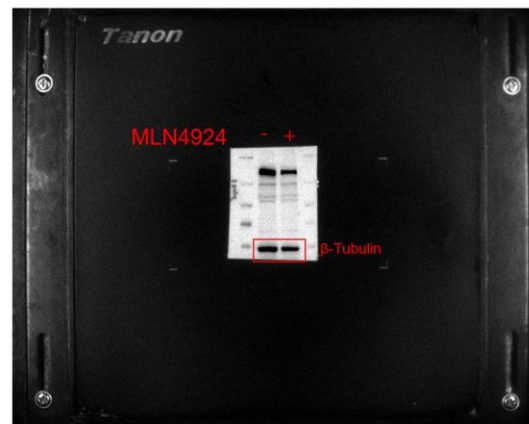

Supplement: Figure 2—figure supplement 1—source data 10. [file elife-88375-fig2-figsupp1-data10.zip › Figure supplement 2-source data 10/Figure supplement 2-source data 10.pdf]

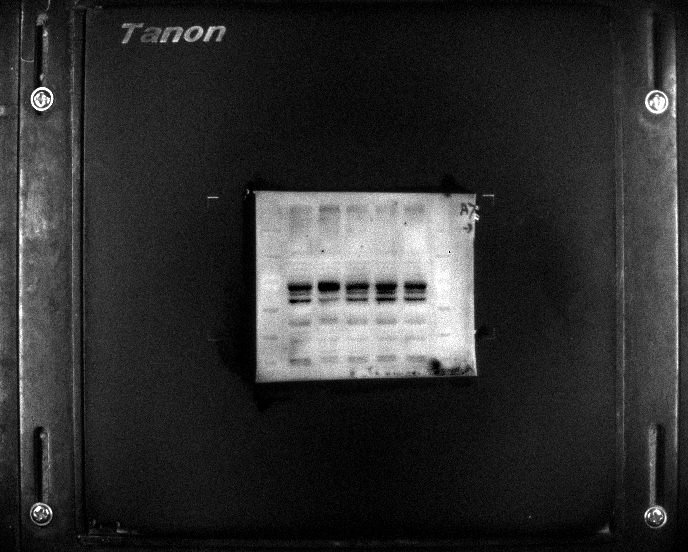

Supplement: Figure 2—figure supplement 1—source data 11. [file elife-88375-fig2-figsupp1-data11.zip › Figure supplement 2-source data 11/Input AChR a7.tif]

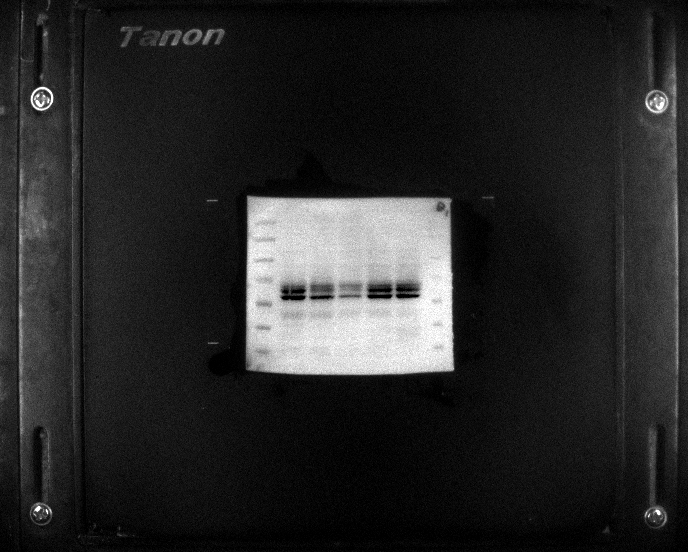

Supplement: Figure 2—figure supplement 1—source data 11. [file elife-88375-fig2-figsupp1-data11.zip › Figure supplement 2-source data 11/Input AChR M2.tif]

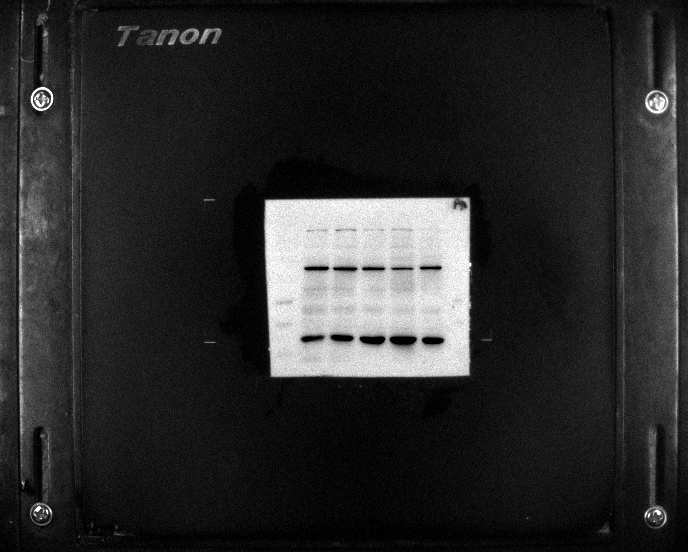

Supplement: Figure 2—figure supplement 1—source data 11. [file elife-88375-fig2-figsupp1-data11.zip › Figure supplement 2-source data 11/Input AChR M3.tif]

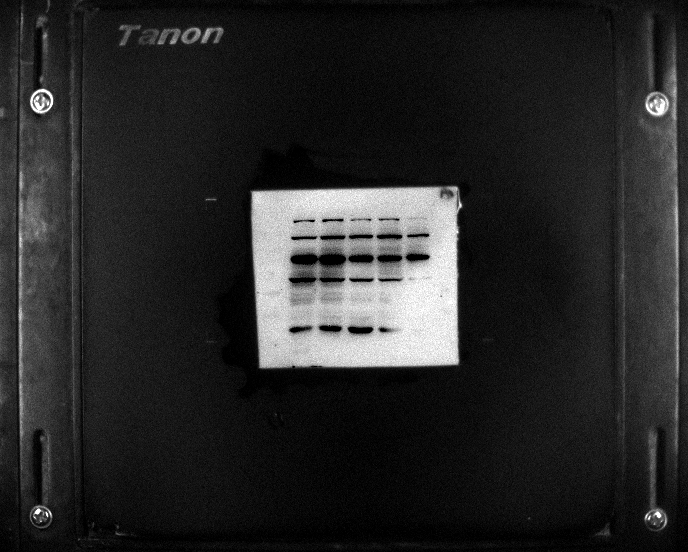

Supplement: Figure 2—figure supplement 1—source data 11. [file elife-88375-fig2-figsupp1-data11.zip › Figure supplement 2-source data 11/Input AChR M4.tif]

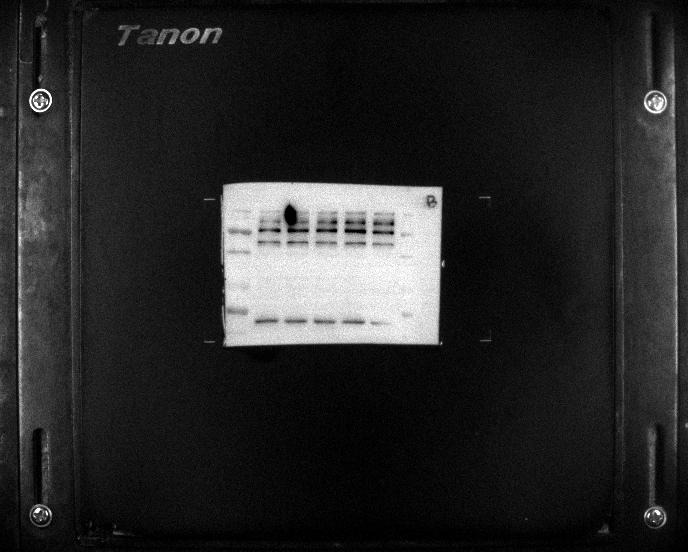

Supplement: Figure 2—figure supplement 1—source data 11. [file elife-88375-fig2-figsupp1-data11.zip › Figure supplement 2-source data 11/Input BCR-ABL.tif]

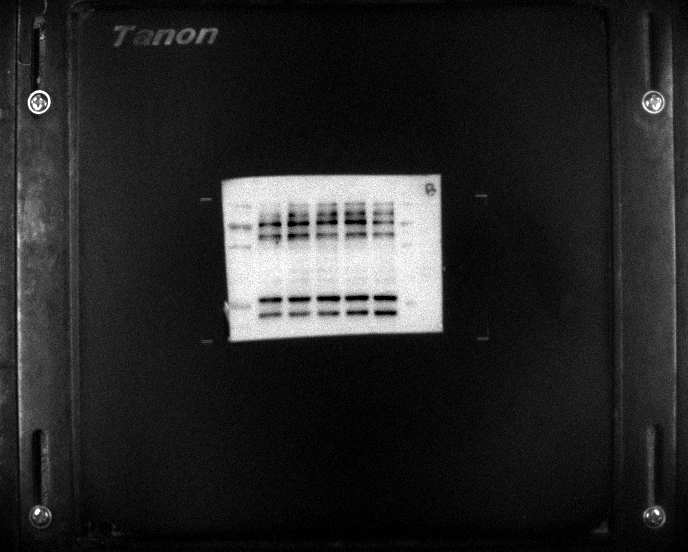

Supplement: Figure 2—figure supplement 1—source data 11. [file elife-88375-fig2-figsupp1-data11.zip › Figure supplement 2-source data 11/Input a┬-Tubulin.tif]

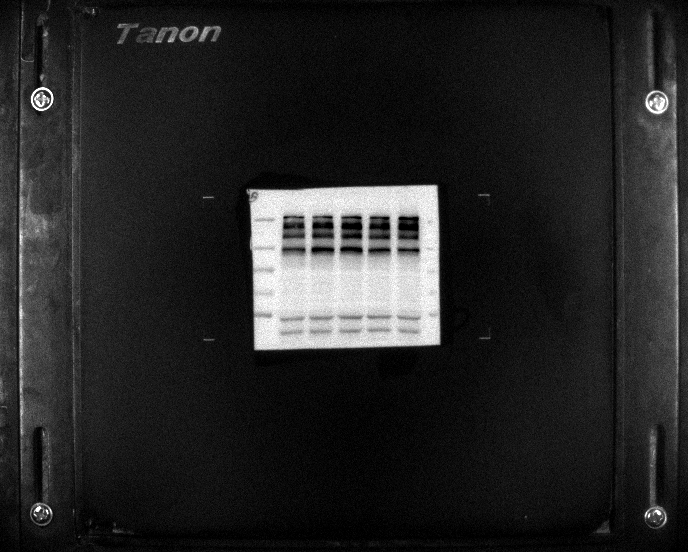

Supplement: Figure 2—figure supplement 1—source data 11. [file elife-88375-fig2-figsupp1-data11.zip › Figure supplement 2-source data 11/IP BCR-ABL-IB BCR-ABL.tif]

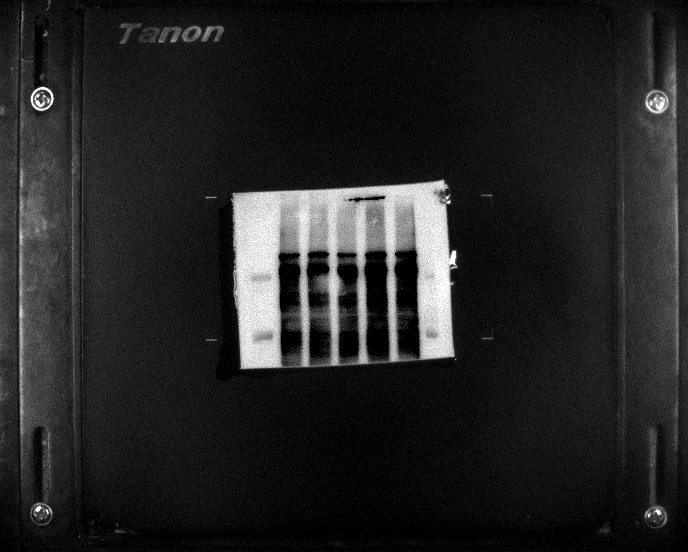

Supplement: Figure 2—figure supplement 1—source data 11. [file elife-88375-fig2-figsupp1-data11.zip › Figure supplement 2-source data 11/IP BCR-ABL-IB NEDD8-4.tif]

G

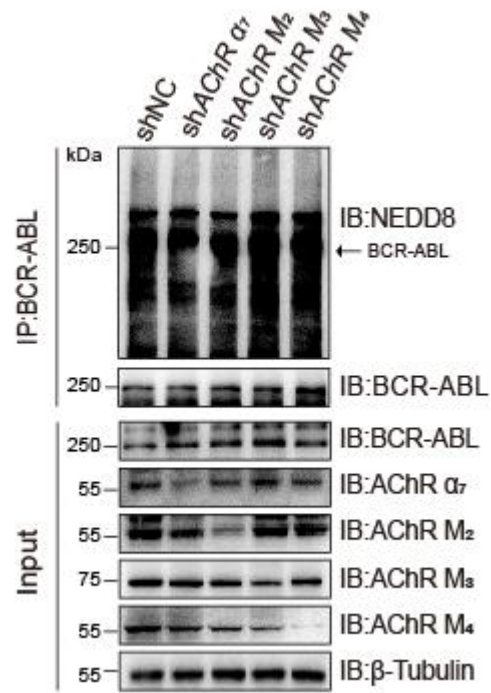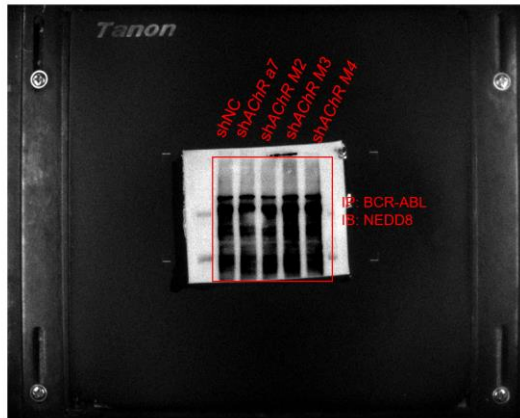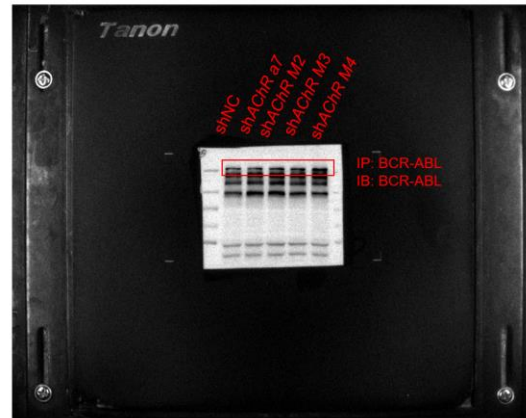

## Input

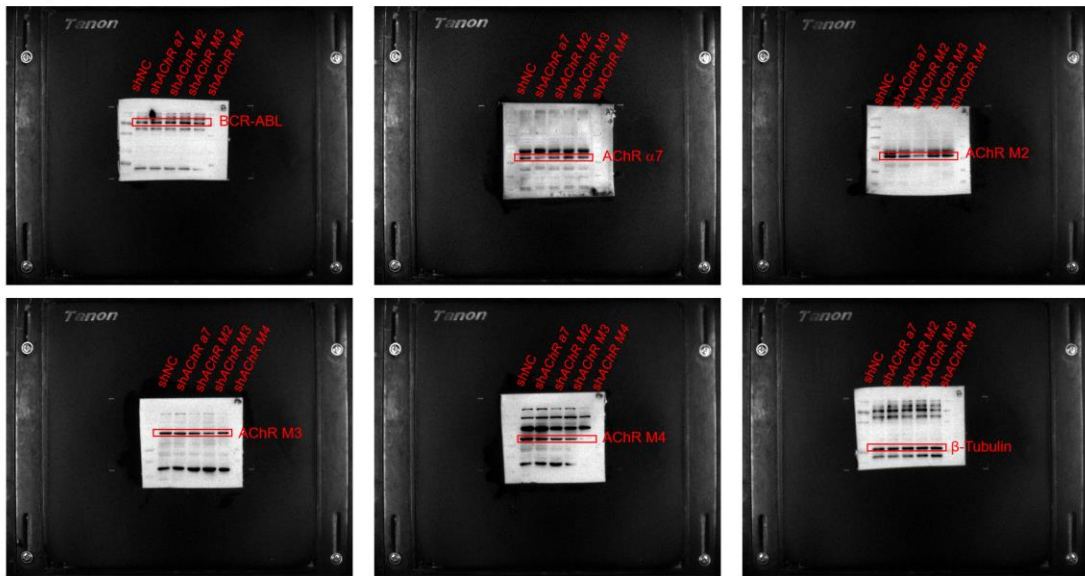

Supplement: Figure 2—figure supplement 1—source data 12. [file elife-88375-fig2-figsupp1-data12.zip › Figure supplement 2-source data 12/Figure supplement 2-source data 12.pdf]

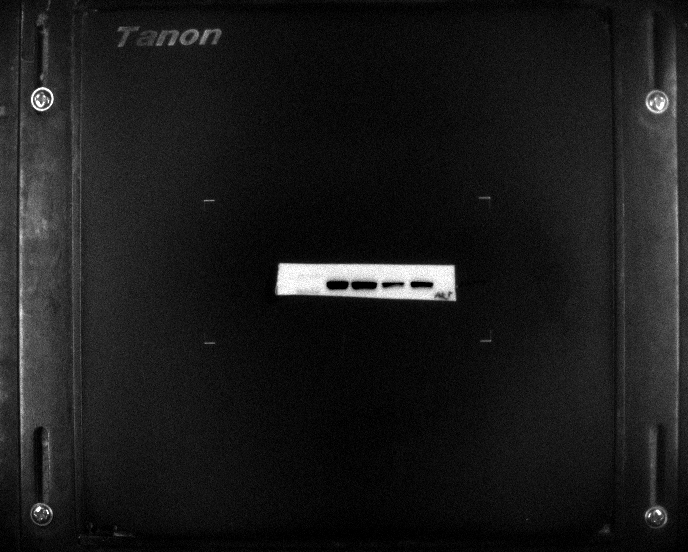

Supplement: Figure 2—figure supplement 1—source data 13. [file elife-88375-fig2-figsupp1-data13.zip › Figure supplement 2-source data 13/AKT.tif]

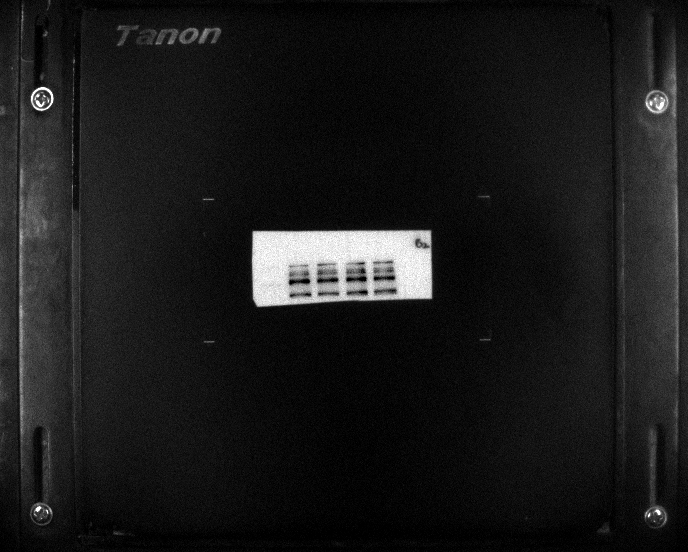

Supplement: Figure 2—figure supplement 1—source data 13. [file elife-88375-fig2-figsupp1-data13.zip › Figure supplement 2-source data 13/BCR-ABL.tif]

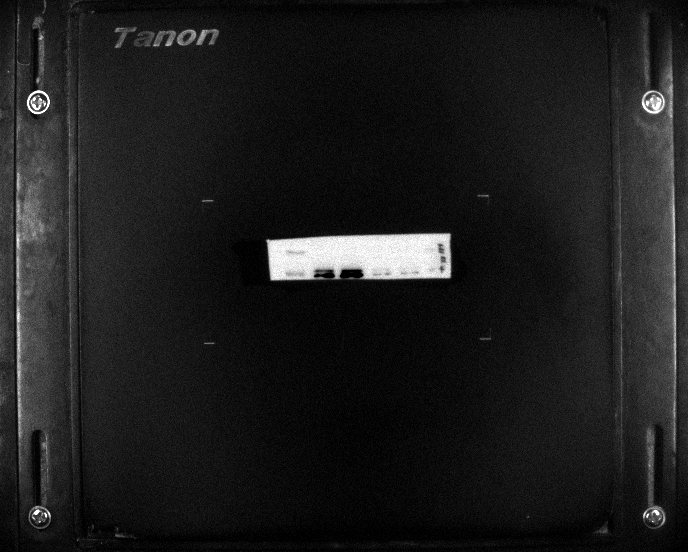

Supplement: Figure 2—figure supplement 1—source data 13. [file elife-88375-fig2-figsupp1-data13.zip › Figure supplement 2-source data 13/ERK.tif]

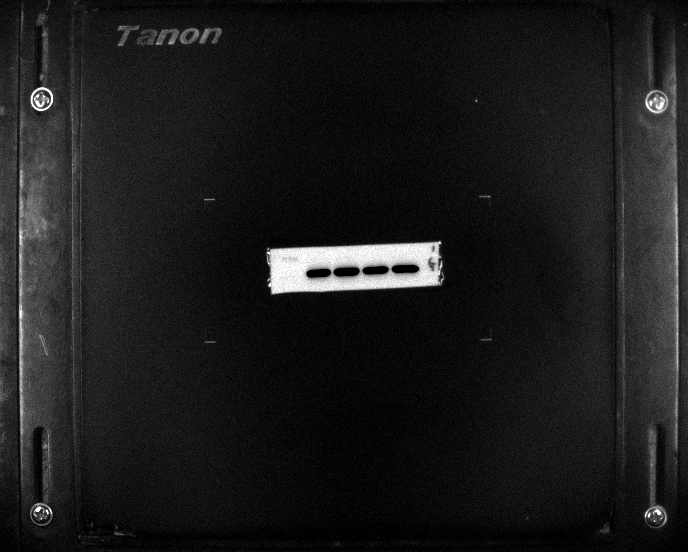

Supplement: Figure 2—figure supplement 1—source data 13. [file elife-88375-fig2-figsupp1-data13.zip › Figure supplement 2-source data 13/GAPDH.tif]

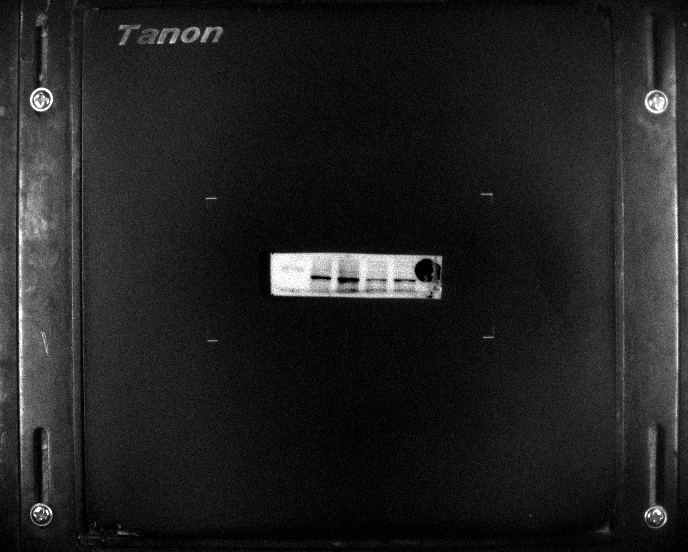

Supplement: Figure 2—figure supplement 1—source data 13. [file elife-88375-fig2-figsupp1-data13.zip › Figure supplement 2-source data 13/JAK2.tif]

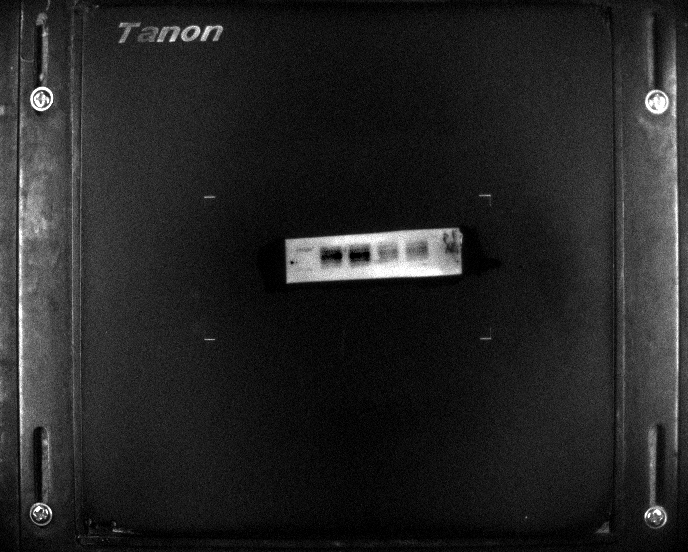

Supplement: Figure 2—figure supplement 1—source data 13. [file elife-88375-fig2-figsupp1-data13.zip › Figure supplement 2-source data 13/P-AKT.tif]

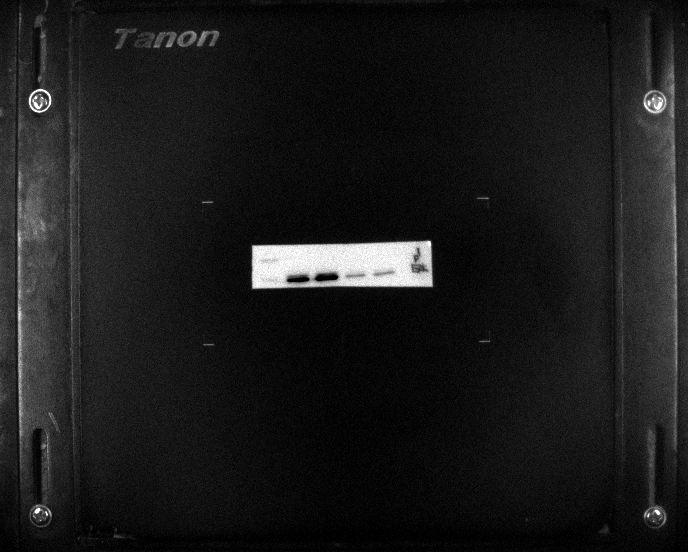

Supplement: Figure 2—figure supplement 1—source data 13. [file elife-88375-fig2-figsupp1-data13.zip › Figure supplement 2-source data 13/p-ERK.tif]

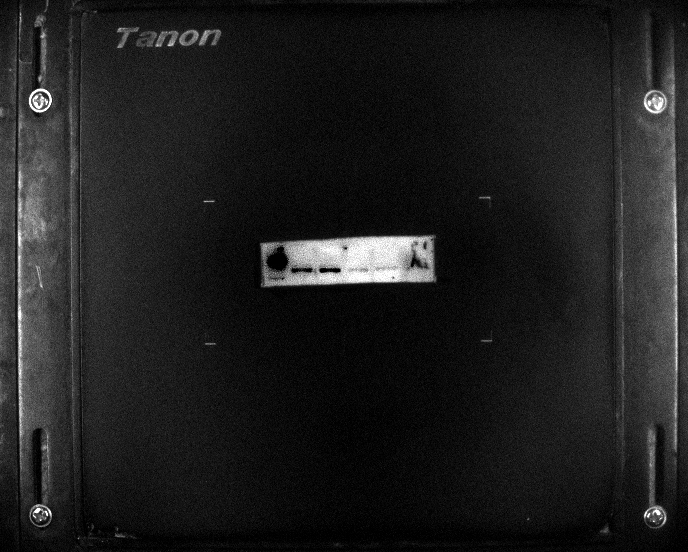

Supplement: Figure 2—figure supplement 1—source data 13. [file elife-88375-fig2-figsupp1-data13.zip › Figure supplement 2-source data 13/P-JAK2.tif]

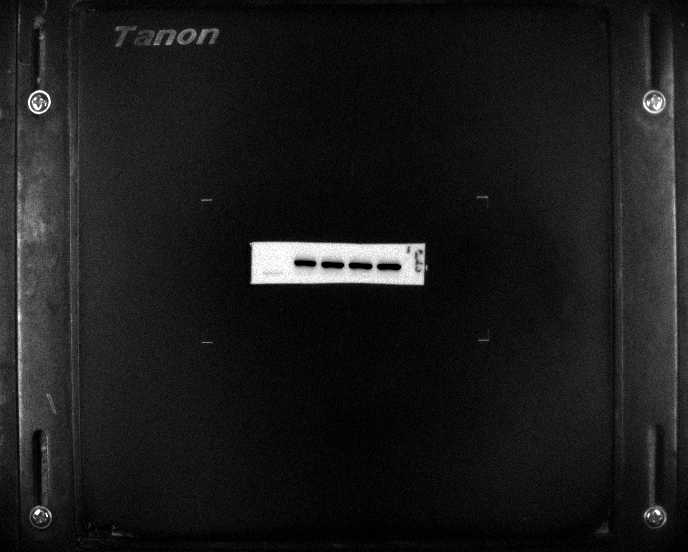

Supplement: Figure 2—figure supplement 1—source data 13. [file elife-88375-fig2-figsupp1-data13.zip › Figure supplement 2-source data 13/PKC.tif]

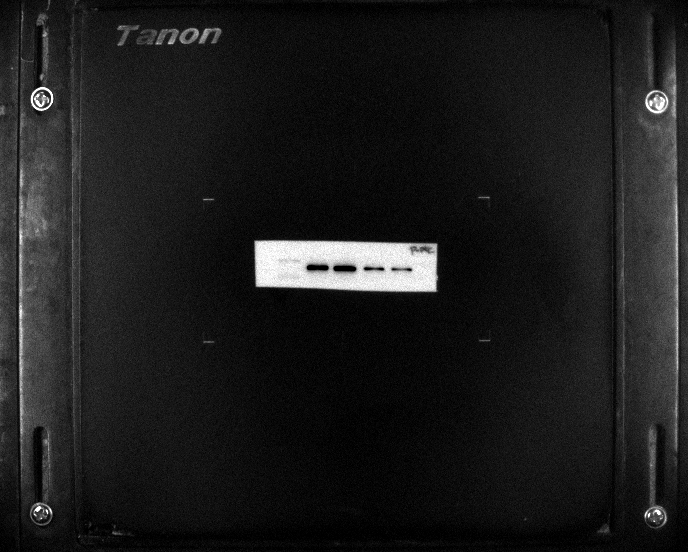

Supplement: Figure 2—figure supplement 1—source data 13. [file elife-88375-fig2-figsupp1-data13.zip › Figure supplement 2-source data 13/p-PKC.tif]

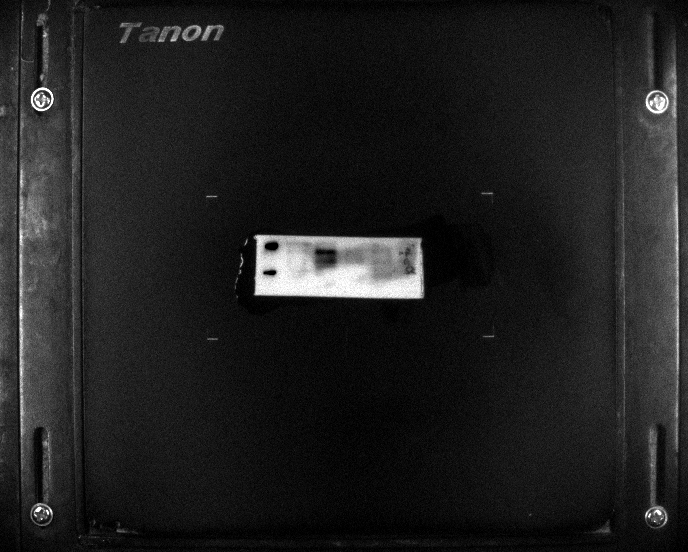

Supplement: Figure 2—figure supplement 1—source data 13. [file elife-88375-fig2-figsupp1-data13.zip › Figure supplement 2-source data 13/RAS.tif]
