# Supplementary figures and images for: RAPSYN-mediated neddylation of BCR-ABL alternatively determines the fate of Philadelphia chromosome-positive leukemia (part 3 of 5)
Source: eLife. 2024 Jun 12;12:RP88375. doi: 10.7554/eLife.88375 (PMC11168747; doi:10.7554/eLife.88375)

H

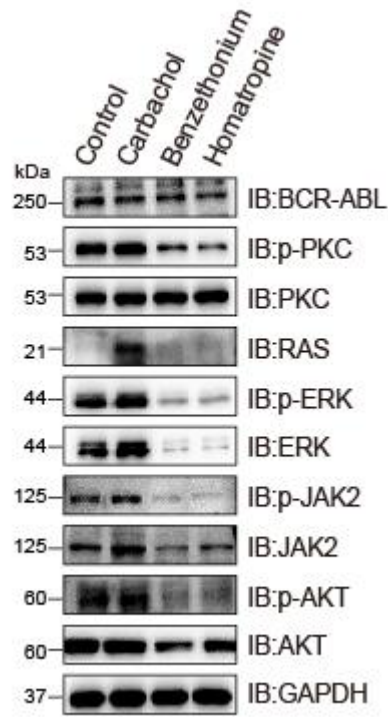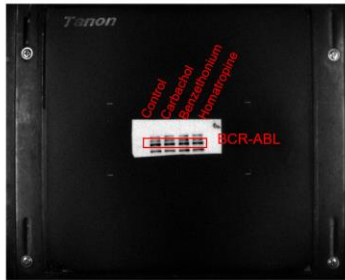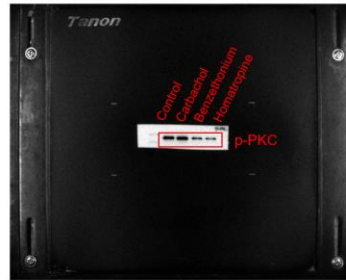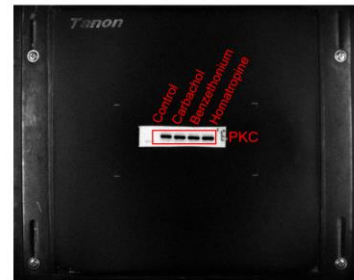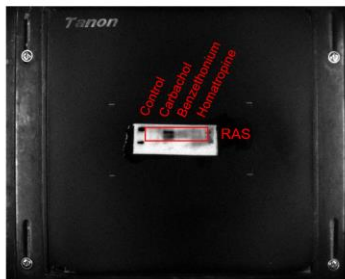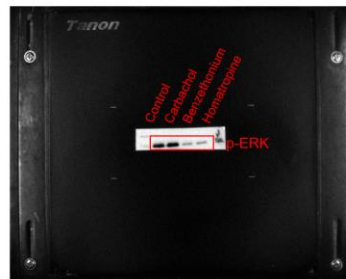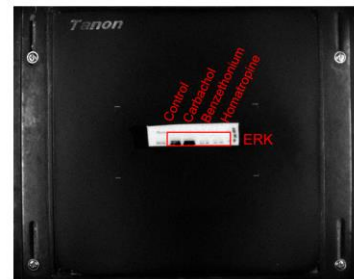

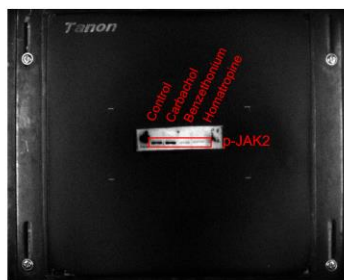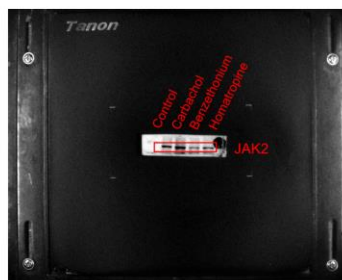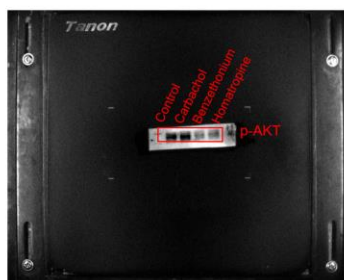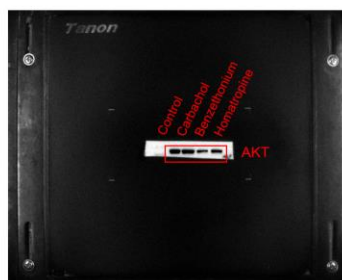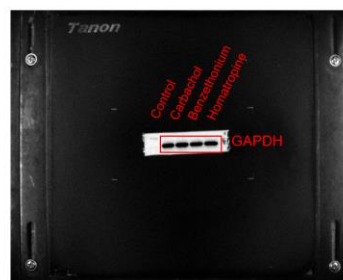

Supplement: Figure 2—figure supplement 1—source data 14. [file elife-88375-fig2-figsupp1-data14.zip › Figure supplement 2-source data 14/Figure supplement 2-source data 14.pdf]

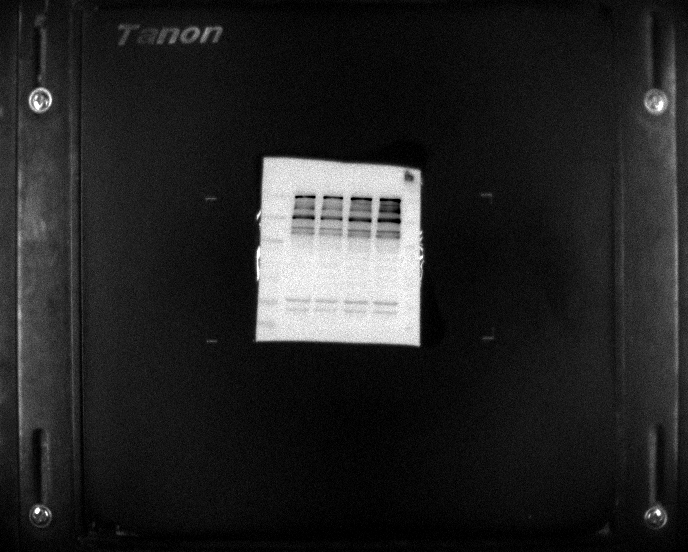

Supplement: Figure 2—figure supplement 1—source data 15. [file elife-88375-fig2-figsupp1-data15.zip › Figure supplement 2-source data 15/Input BCR-ABL.tif]

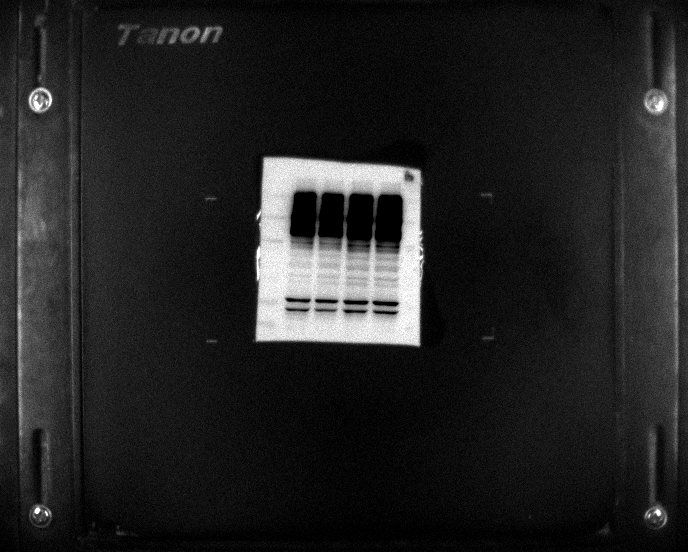

Supplement: Figure 2—figure supplement 1—source data 15. [file elife-88375-fig2-figsupp1-data15.zip › Figure supplement 2-source data 15/Input a┬-Tubulin.tif]

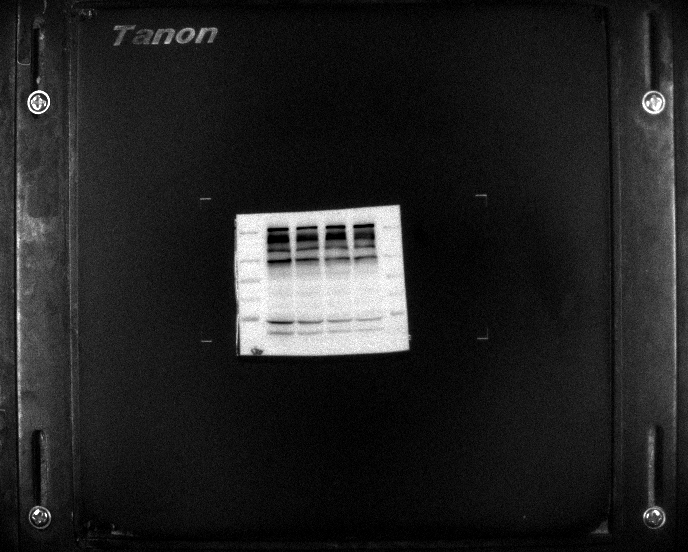

Supplement: Figure 2—figure supplement 1—source data 15. [file elife-88375-fig2-figsupp1-data15.zip › Figure supplement 2-source data 15/IP BCR-ABL-IB BCR-ABL.tif]

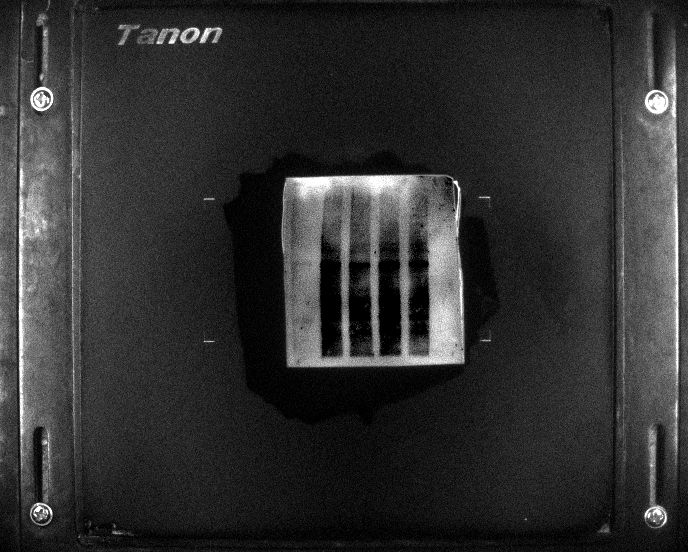

Supplement: Figure 2—figure supplement 1—source data 15. [file elife-88375-fig2-figsupp1-data15.zip › Figure supplement 2-source data 15/IP BCR-ABL-IB NEDD8.tif]

I

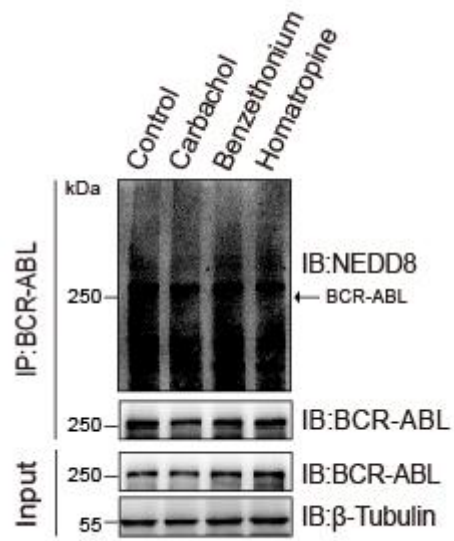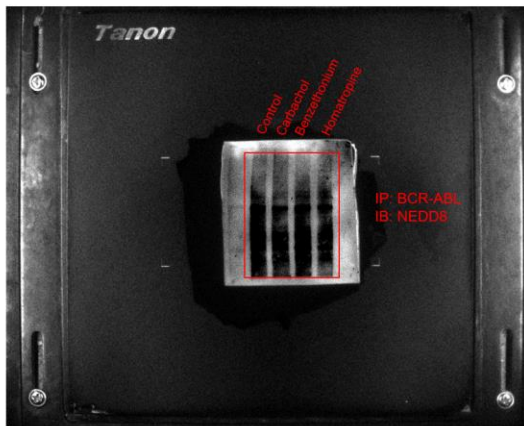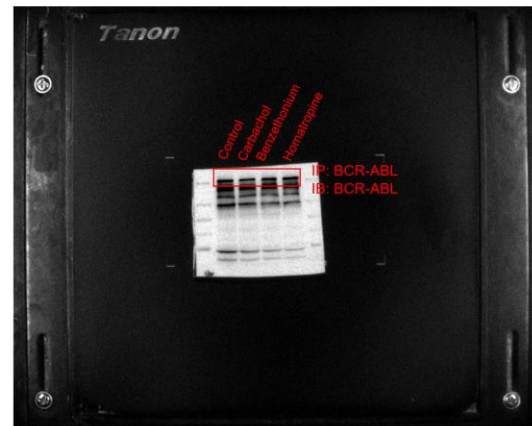

Input

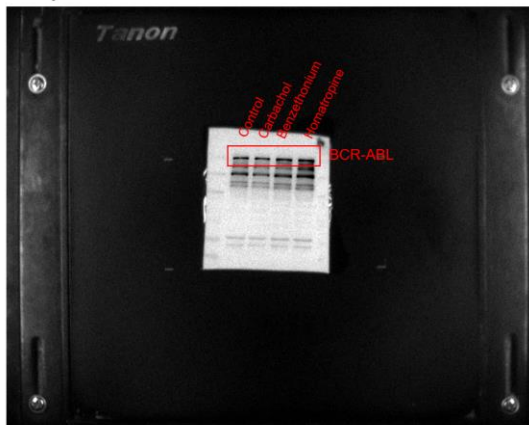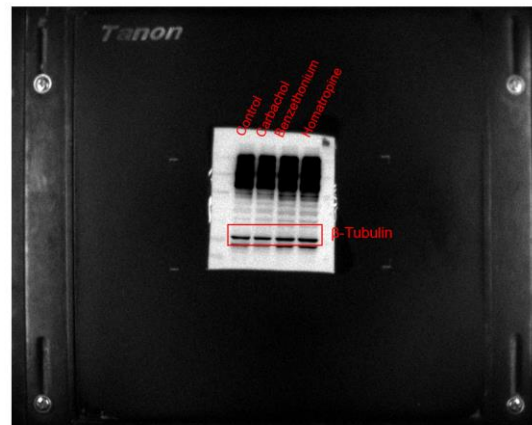

Supplement: Figure 2—figure supplement 1—source data 16. [file elife-88375-fig2-figsupp1-data16.zip › Figure supplement 2-source data 16/Figure supplement 2-source data 16.pdf]

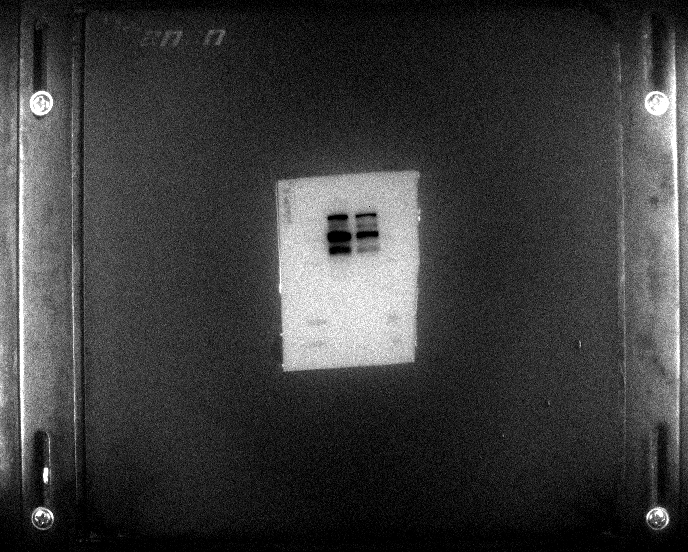

Supplement: Figure 3—source data 1. [file elife-88375-fig3-data1.zip › Figure 3-source data 1/K562 BCR-ABL.tif]

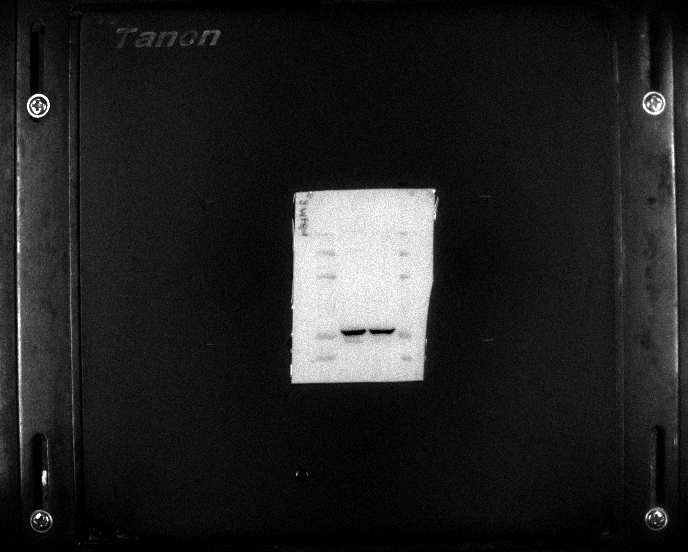

Supplement: Figure 3—source data 1. [file elife-88375-fig3-data1.zip › Figure 3-source data 1/K562 a┬-Tubulin.tif]

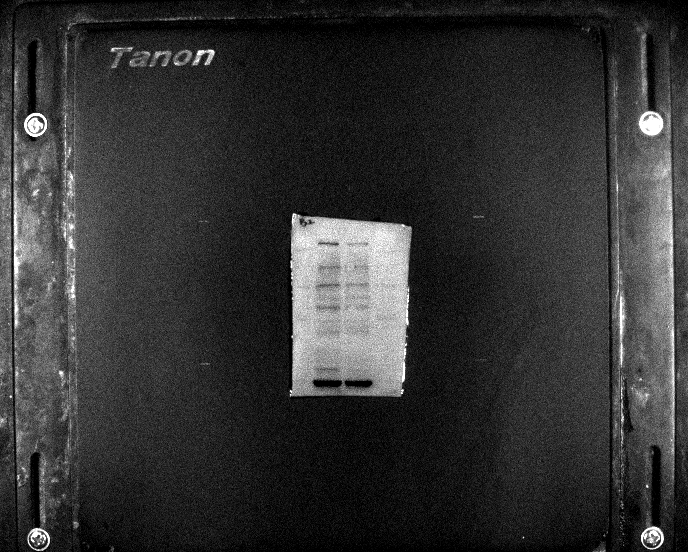

Supplement: Figure 3—source data 1. [file elife-88375-fig3-data1.zip › Figure 3-source data 1/MEG-01 a┬-Tubulin.tif]

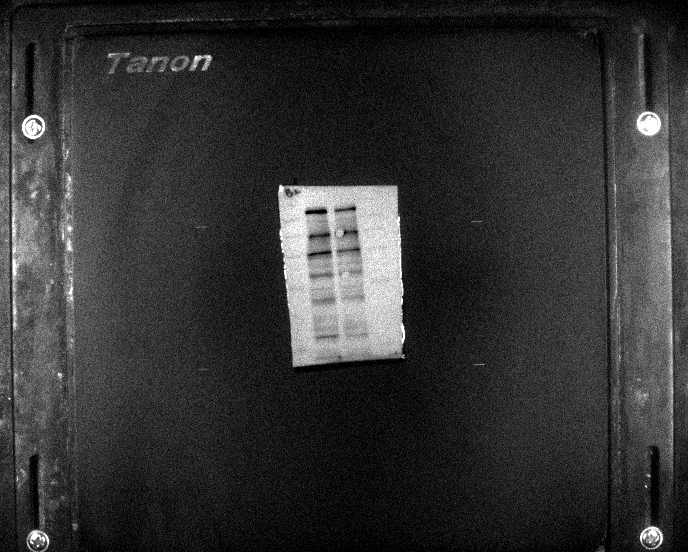

Supplement: Figure 3—source data 1. [file elife-88375-fig3-data1.zip › Figure 3-source data 1/MEG-01 BCR-ABL.tif]

A

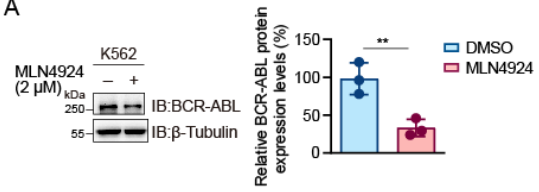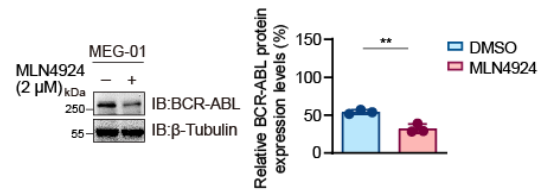

K562

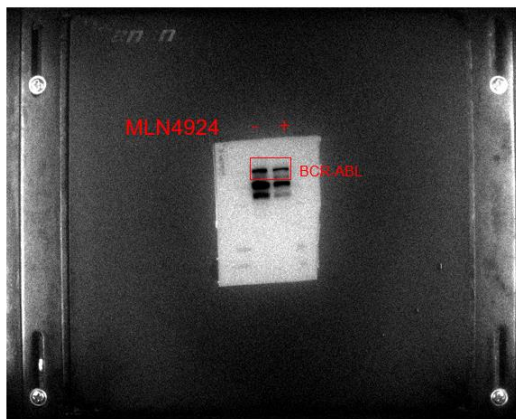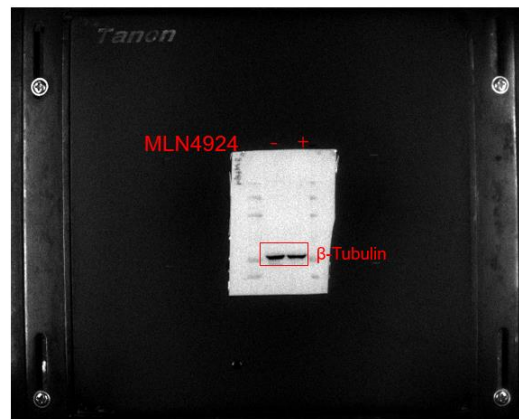

MEG-01

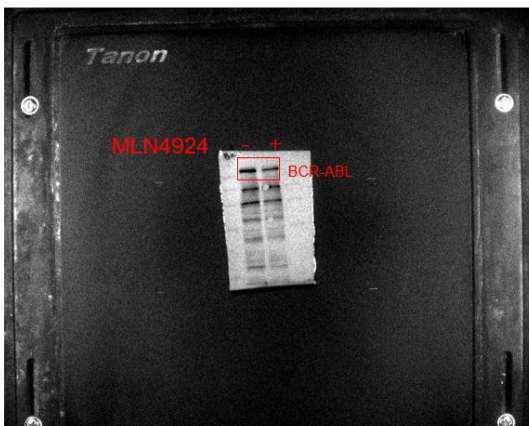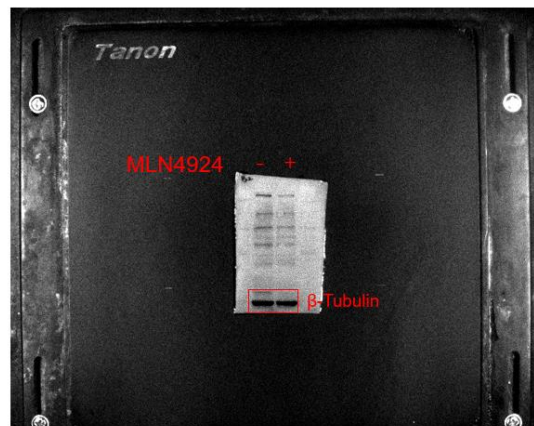

Supplement: Figure 3—source data 2. [file elife-88375-fig3-data2.zip › Figure 3-source data 2/Figure 3-source data 2.pdf]

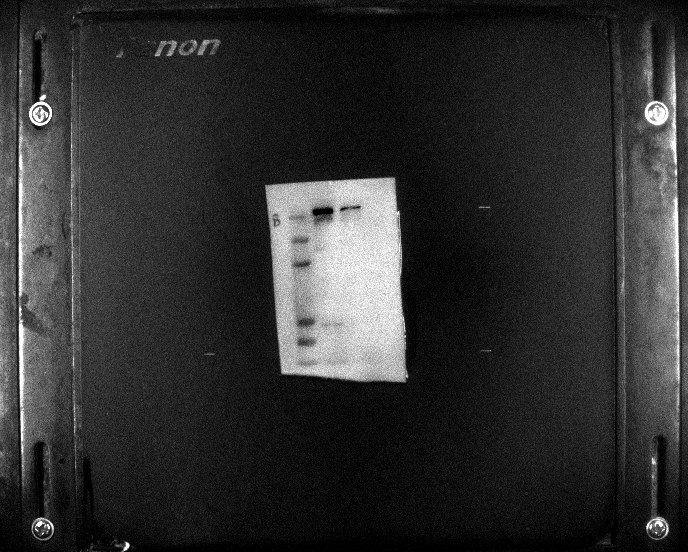

Supplement: Figure 3—source data 3. [file elife-88375-fig3-data3.zip › Figure 3-source data 3/BCR-ABL.tif]

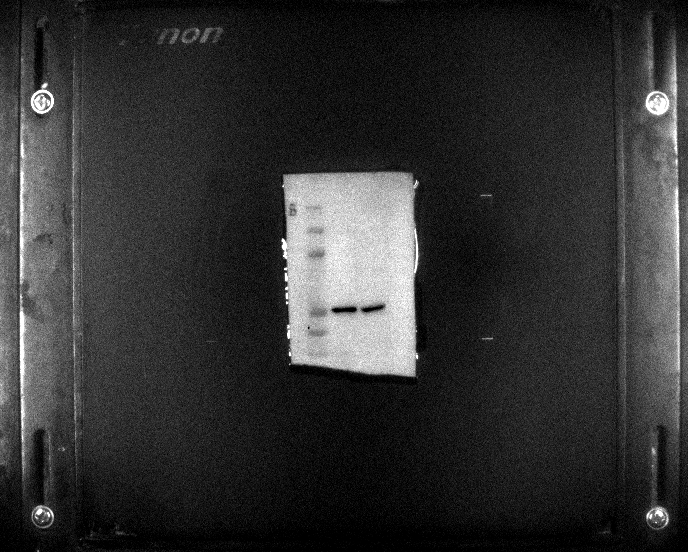

Supplement: Figure 3—source data 3. [file elife-88375-fig3-data3.zip › Figure 3-source data 3/a┬-Tubulin.tif]

B

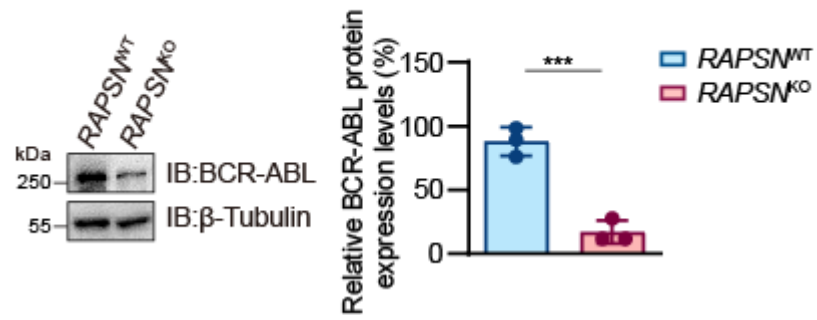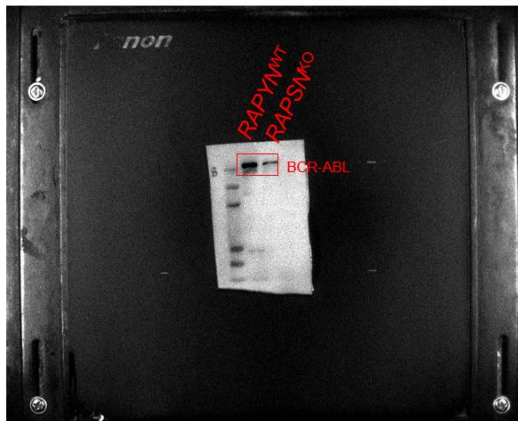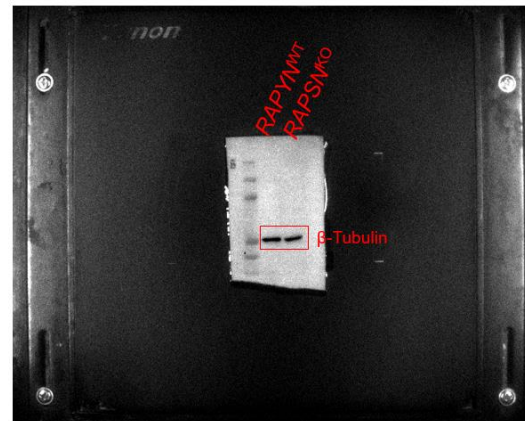

Supplement: Figure 3—source data 4. [file elife-88375-fig3-data4.zip › Figure 3-source data 4/Figure 3-source data 4.pdf]

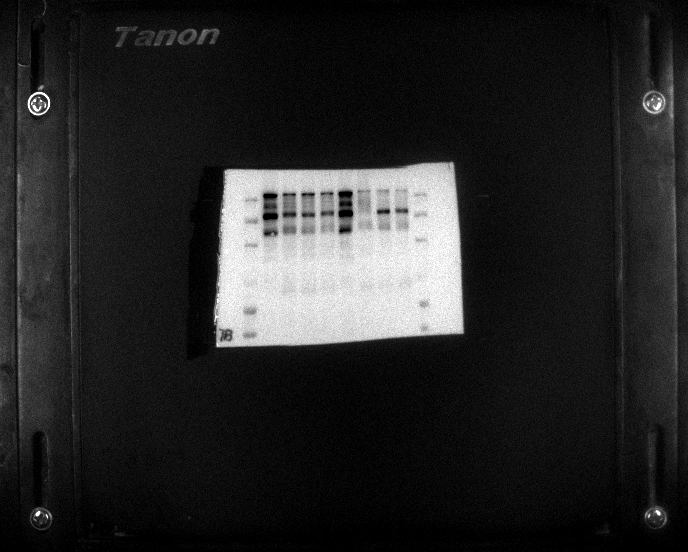

Supplement: Figure 3—source data 5. [file elife-88375-fig3-data5.zip › Figure 3-sourece data 5/BCR-ABL.tif]

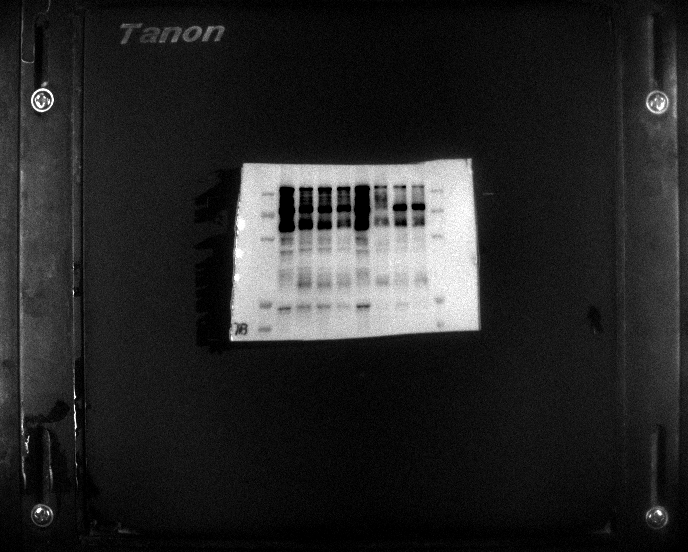

Supplement: Figure 3—source data 5. [file elife-88375-fig3-data5.zip › Figure 3-sourece data 5/RAPSYN.tif]

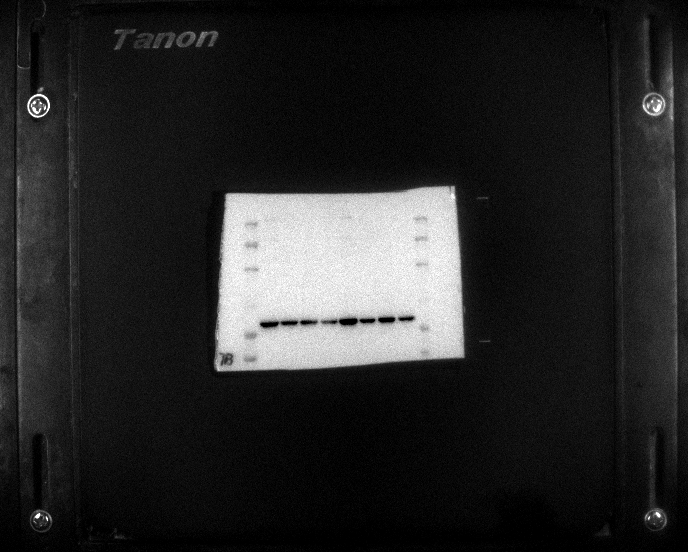

Supplement: Figure 3—source data 5. [file elife-88375-fig3-data5.zip › Figure 3-sourece data 5/a┬-Tubulin.tif]

C

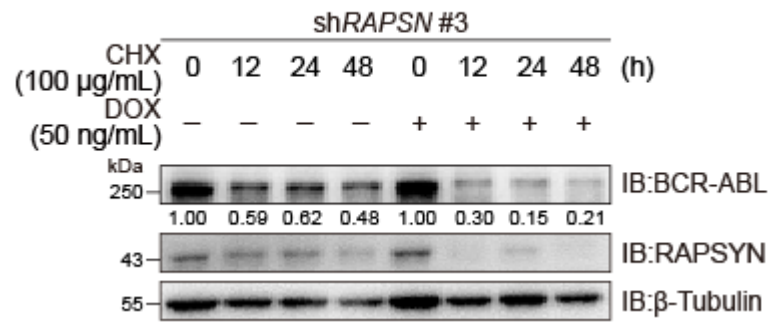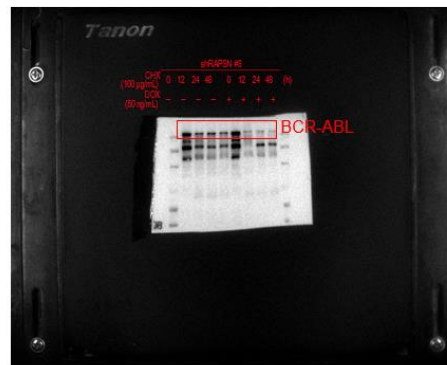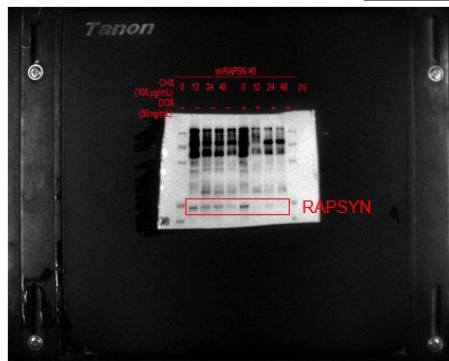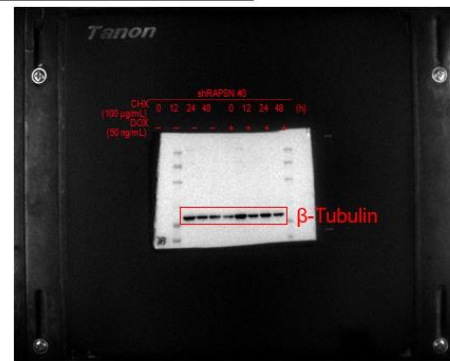

Supplement: Figure 3—source data 6. [file elife-88375-fig3-data6.zip › Figure 3-sourece data 6/Figure 3-source data 6.pdf]

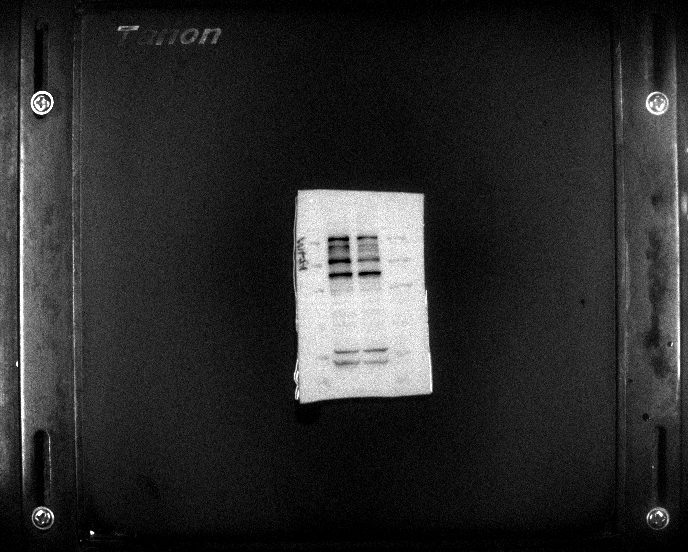

Supplement: Figure 3—source data 7. [file elife-88375-fig3-data7.zip › Figure 3-source data 7/K562 Input BCR-ABL.tif]

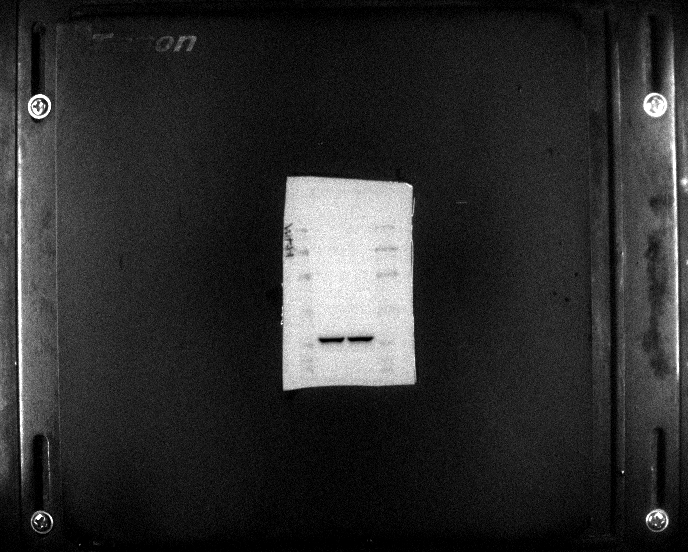

Supplement: Figure 3—source data 7. [file elife-88375-fig3-data7.zip › Figure 3-source data 7/K562 Input a┬-Tubulin.tif]

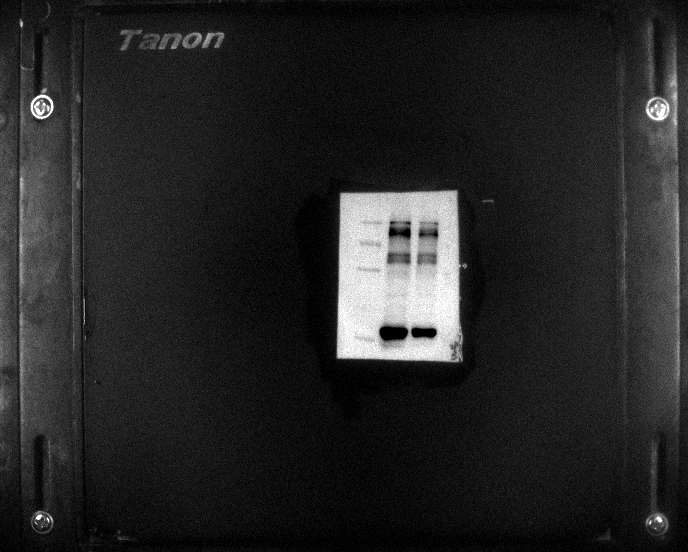

Supplement: Figure 3—source data 7. [file elife-88375-fig3-data7.zip › Figure 3-source data 7/K562 IP BCR-ABL-IB BCR-ABL.tif]

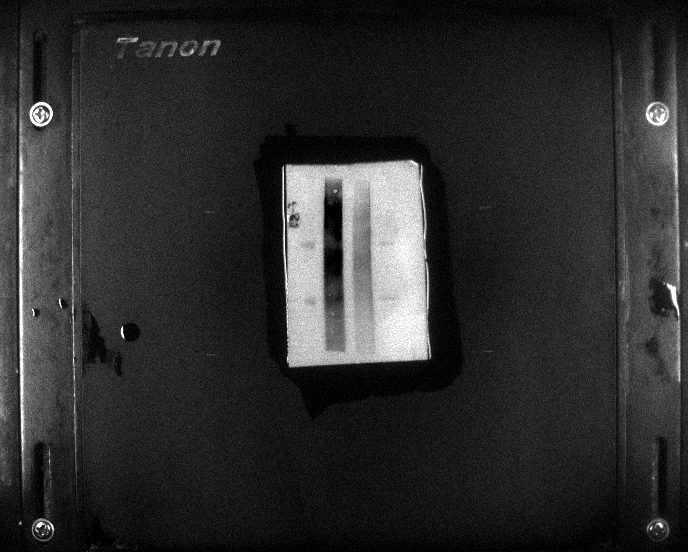

Supplement: Figure 3—source data 7. [file elife-88375-fig3-data7.zip › Figure 3-source data 7/K562 IP BCR-ABL-IB NEDD8.tif]

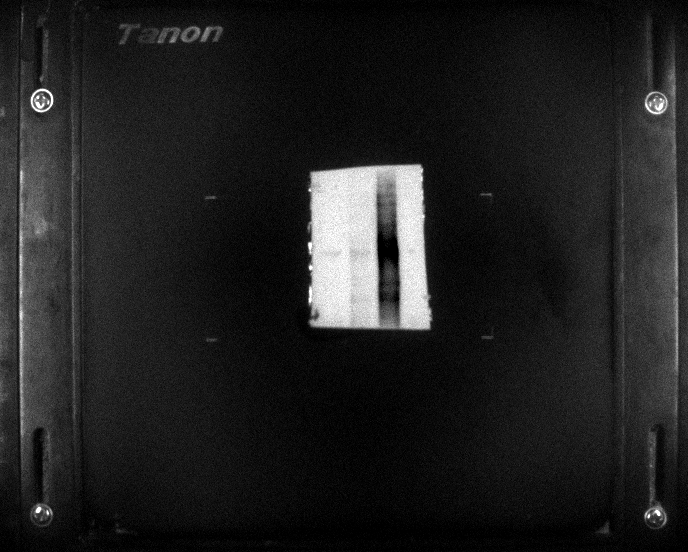

Supplement: Figure 3—source data 7. [file elife-88375-fig3-data7.zip › Figure 3-source data 7/K562 IP BCR-ABL-IB Ub.tif]

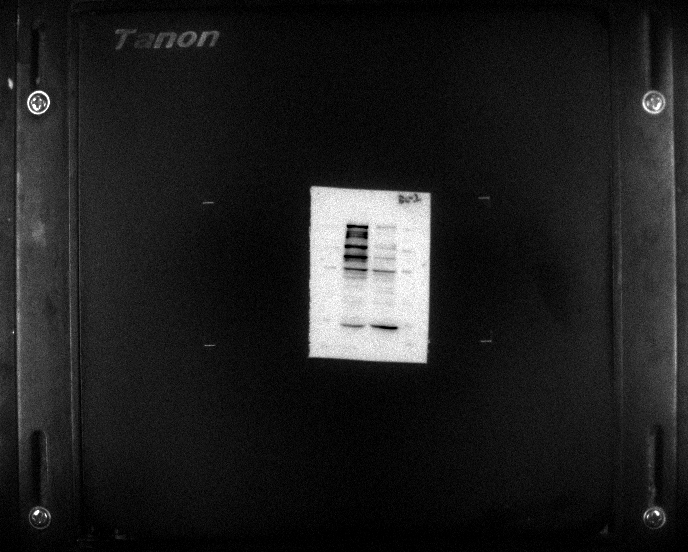

Supplement: Figure 3—source data 7. [file elife-88375-fig3-data7.zip › Figure 3-source data 7/MEG-01 Input BCR-ABL.tif]

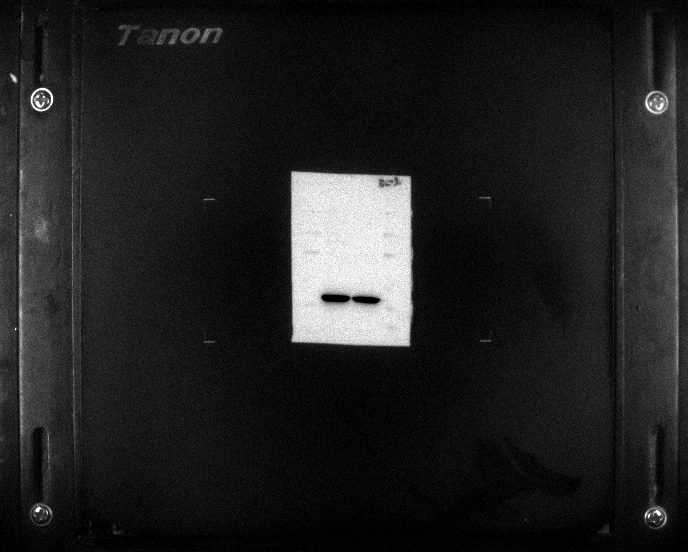

Supplement: Figure 3—source data 7. [file elife-88375-fig3-data7.zip › Figure 3-source data 7/MEG-01 Inut a┬-Tubulin.tif]

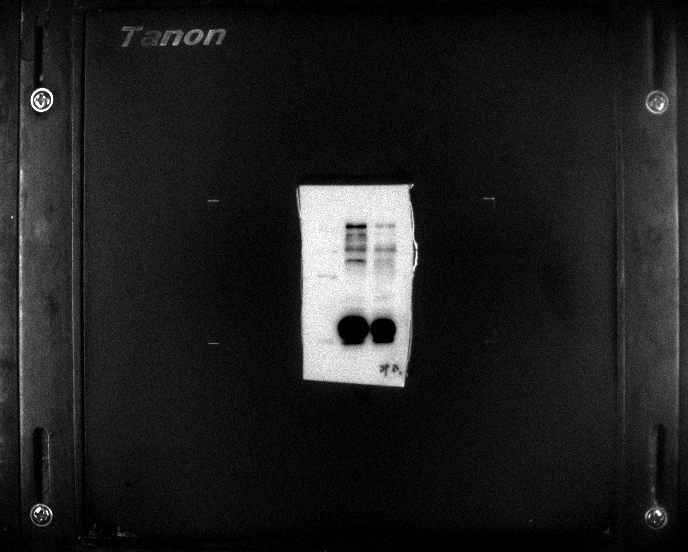

Supplement: Figure 3—source data 7. [file elife-88375-fig3-data7.zip › Figure 3-source data 7/MEG-01 IP BCR-ABL-IB BCR-ABL.tif]

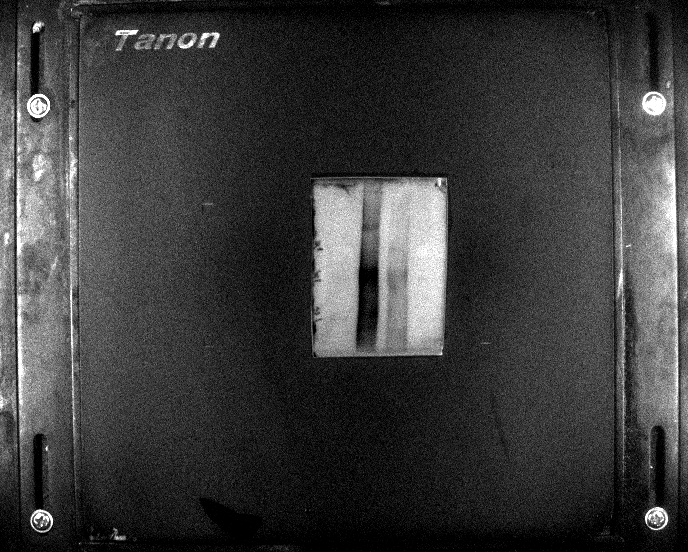

Supplement: Figure 3—source data 7. [file elife-88375-fig3-data7.zip › Figure 3-source data 7/MEG-01 IP BCR-ABL-IB NEDD8.tif]

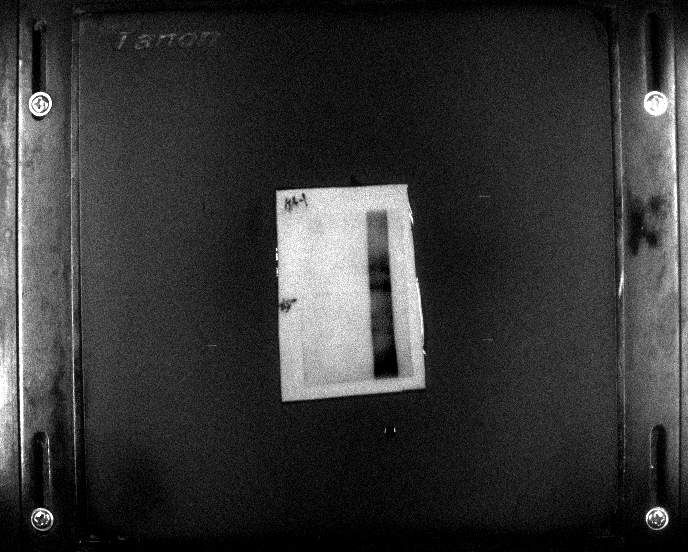

Supplement: Figure 3—source data 7. [file elife-88375-fig3-data7.zip › Figure 3-source data 7/MEG-01 IP BCR-ABL-IB Ub.tif]

D

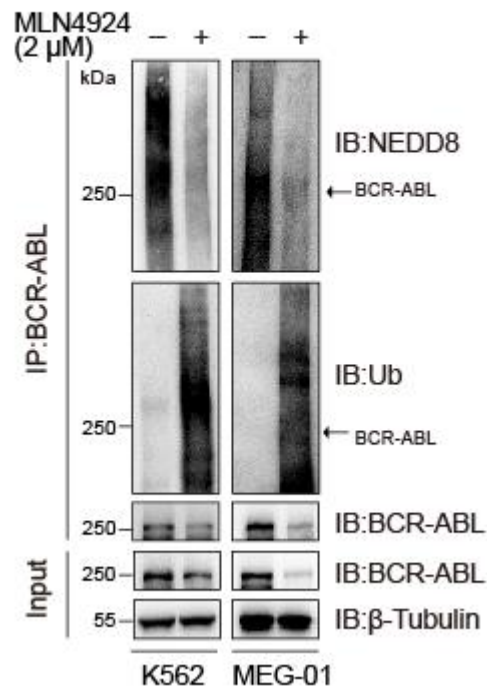

K562

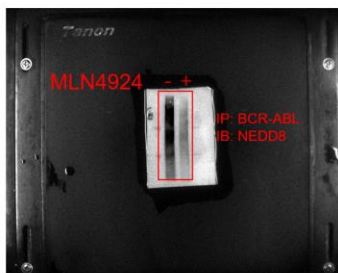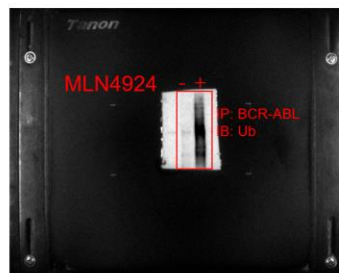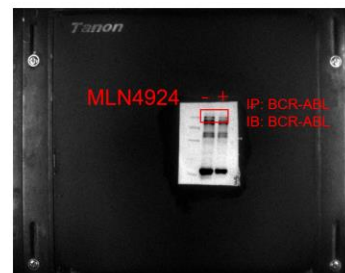

Input

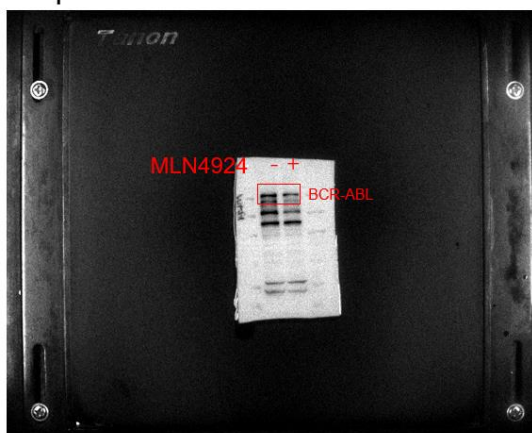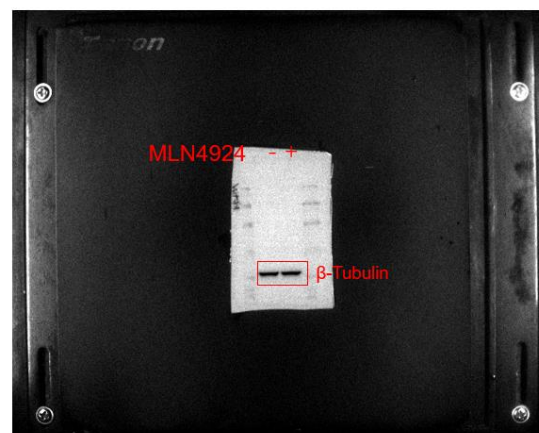

MEG-01

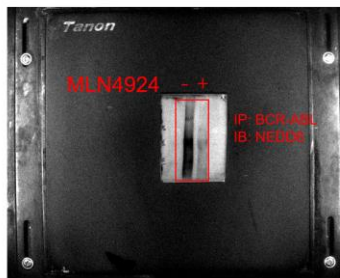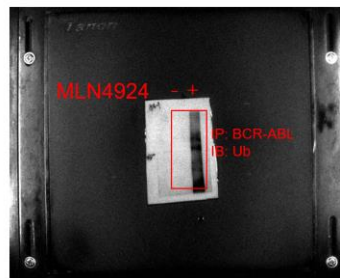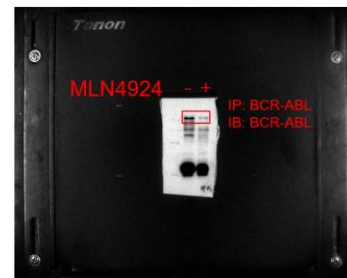

Input

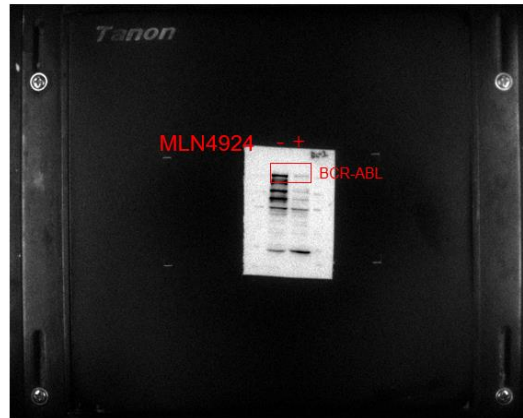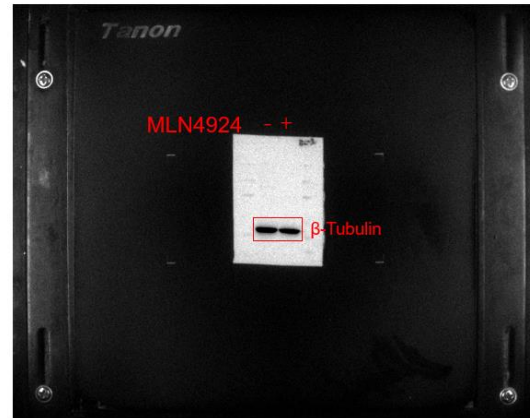

Supplement: Figure 3—source data 8. [file elife-88375-fig3-data8.zip › Figure 3-source data 8/Figure 3-source data 8.pdf]

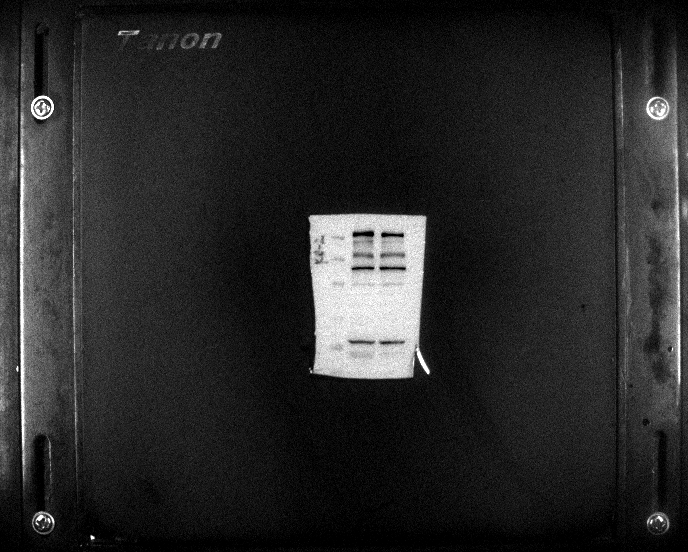

Supplement: Figure 3—source data 9. [file elife-88375-fig3-data9.zip › Figure 3-source data 9/Input BCR-ABL.tif]

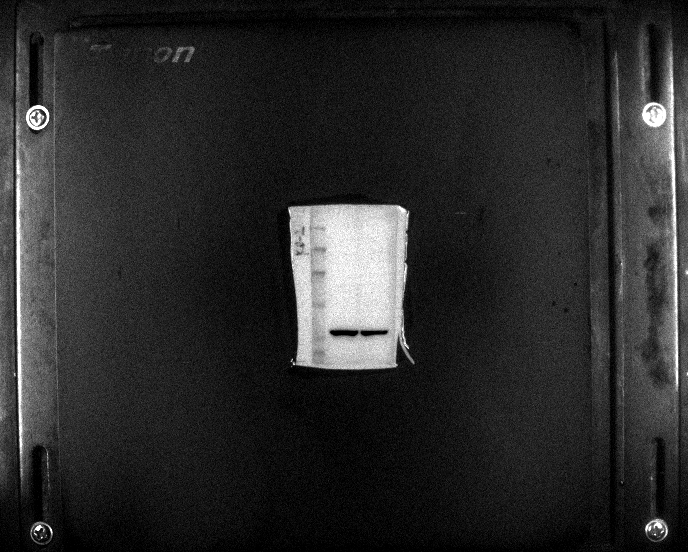

Supplement: Figure 3—source data 9. [file elife-88375-fig3-data9.zip › Figure 3-source data 9/Input a┬-Tubulin.tif]

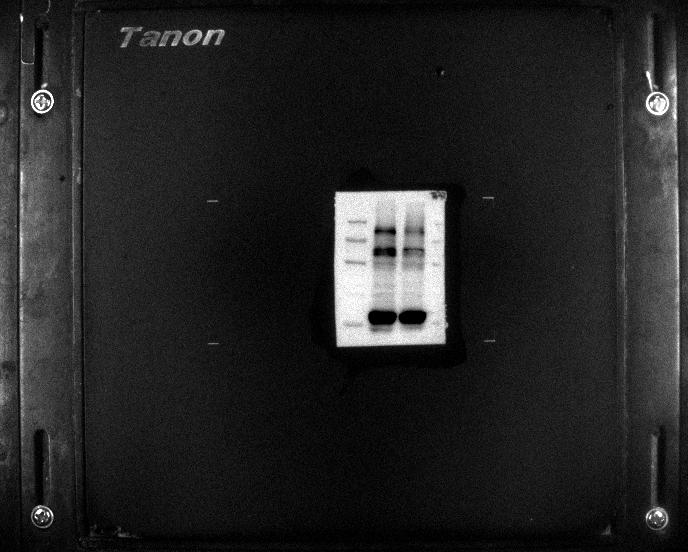

Supplement: Figure 3—source data 9. [file elife-88375-fig3-data9.zip › Figure 3-source data 9/IP BCR-ABL-IB BCR-ABL.tif]

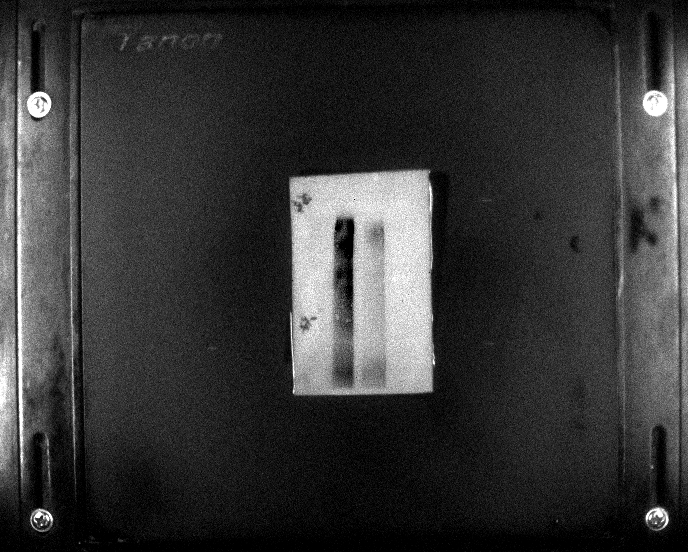

Supplement: Figure 3—source data 9. [file elife-88375-fig3-data9.zip › Figure 3-source data 9/IP BCR-ABL-IB NEDD8.tif]

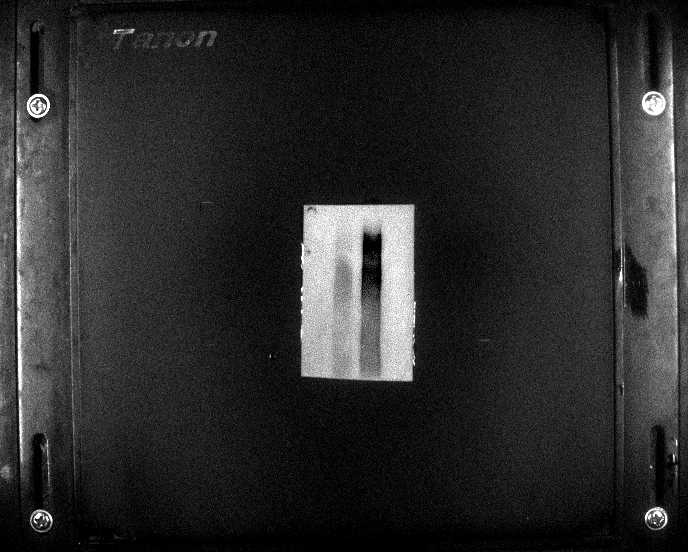

Supplement: Figure 3—source data 9. [file elife-88375-fig3-data9.zip › Figure 3-source data 9/IP BCR-ABL-IB Ub.tif]

E

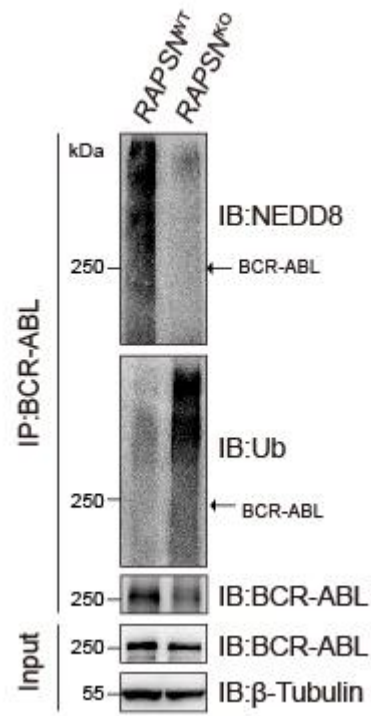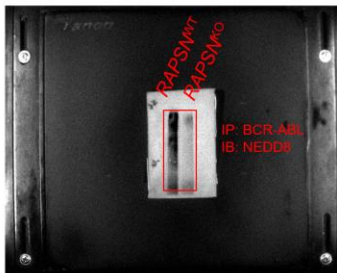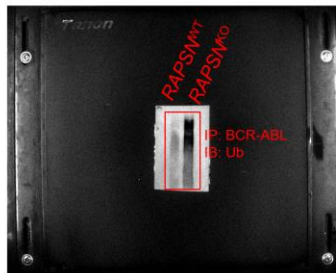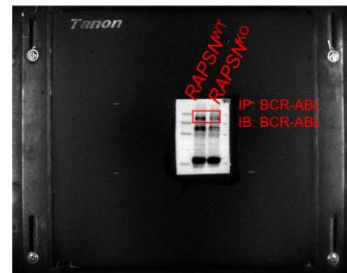

Input

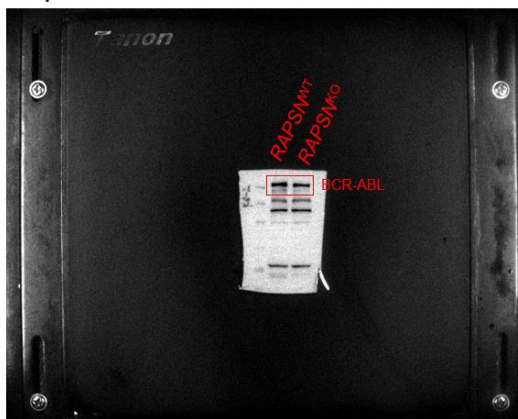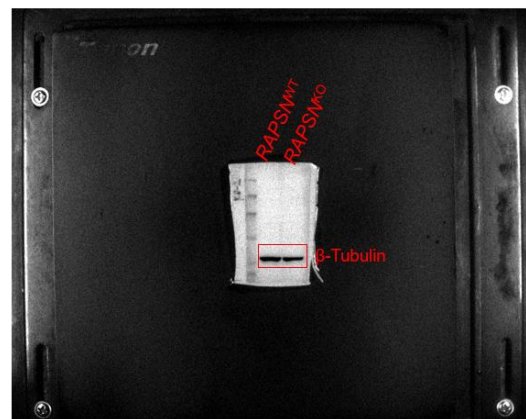

Supplement: Figure 3—source data 10. [file elife-88375-fig3-data10.zip › Figure 3-source data 10/Figure 3-source data 10.pdf]

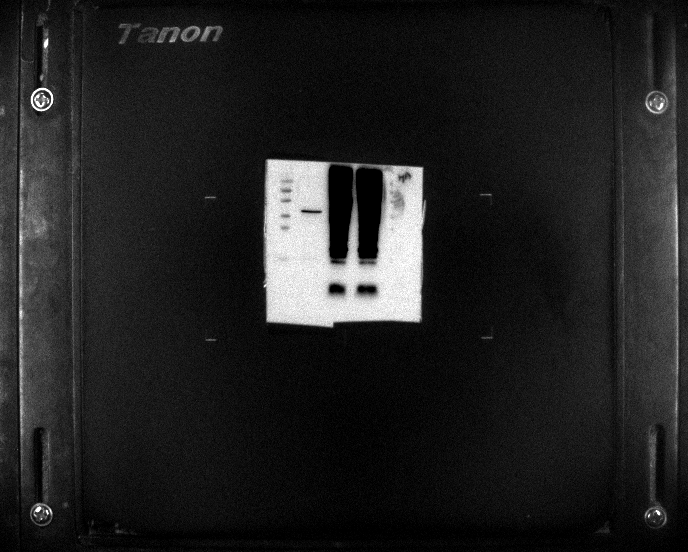

Supplement: Figure 3—source data 11. [file elife-88375-fig3-data11.zip › Figure 3-source data 11/Input HA.tif]

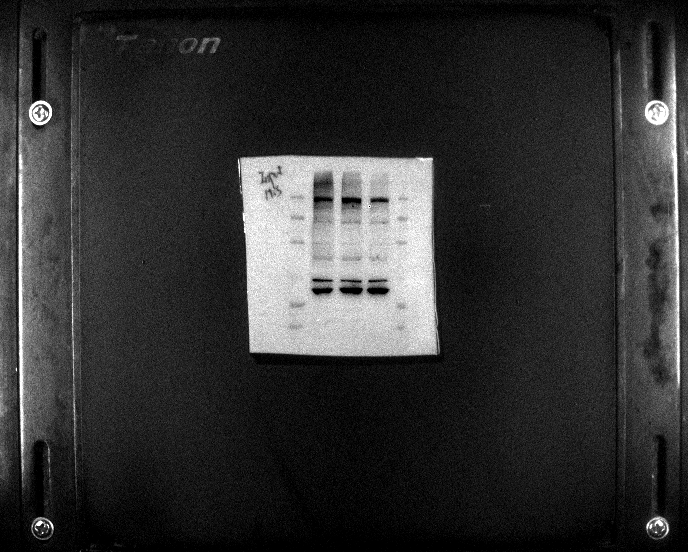

Supplement: Figure 3—source data 11. [file elife-88375-fig3-data11.zip › Figure 3-source data 11/Input His.tif]

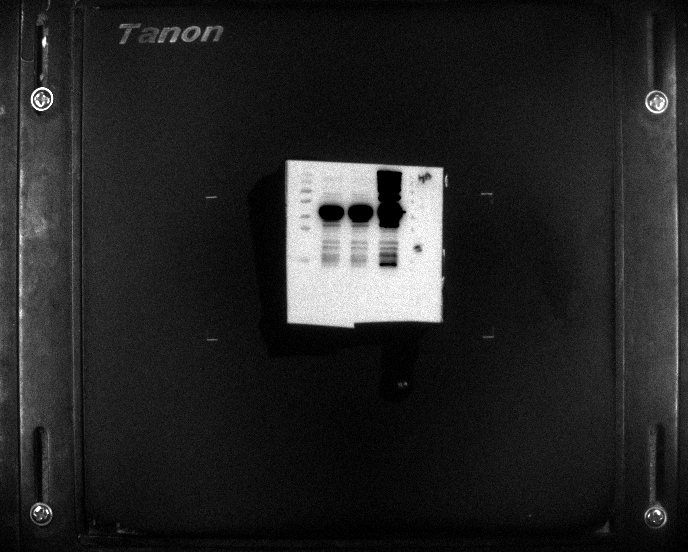

Supplement: Figure 3—source data 11. [file elife-88375-fig3-data11.zip › Figure 3-source data 11/Input Myc.tif]

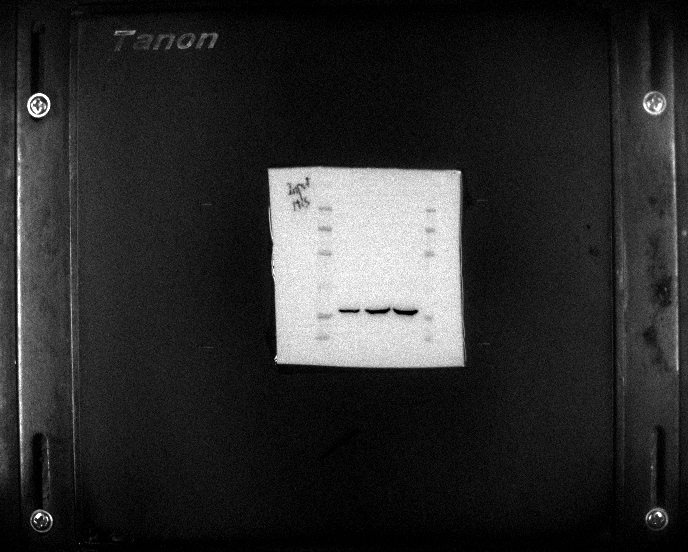

Supplement: Figure 3—source data 11. [file elife-88375-fig3-data11.zip › Figure 3-source data 11/Input a┬-Tubulin-1.tif]

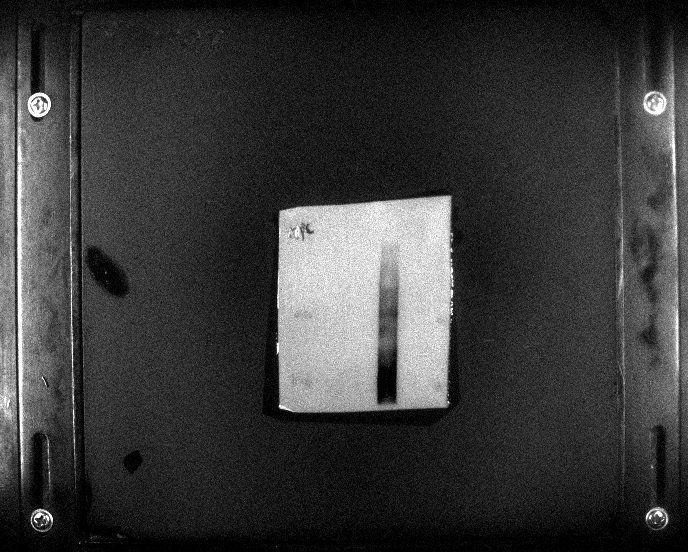

Supplement: Figure 3—source data 11. [file elife-88375-fig3-data11.zip › Figure 3-source data 11/IP His IB Myc.tif]

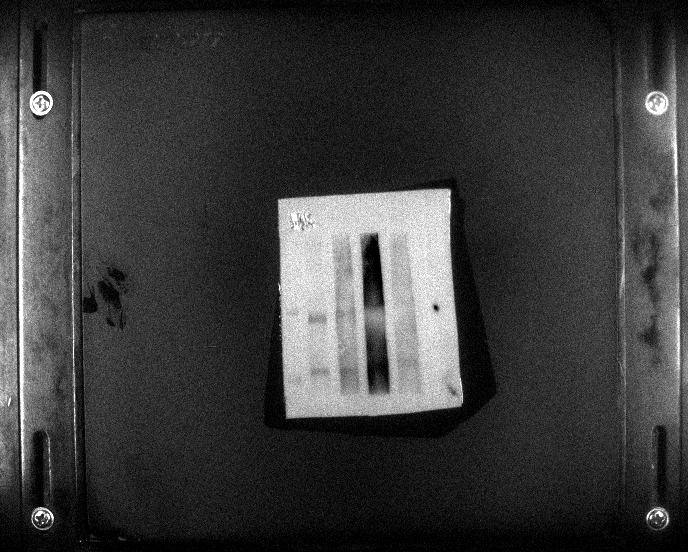

Supplement: Figure 3—source data 11. [file elife-88375-fig3-data11.zip › Figure 3-source data 11/IP His IB-HA.tif]

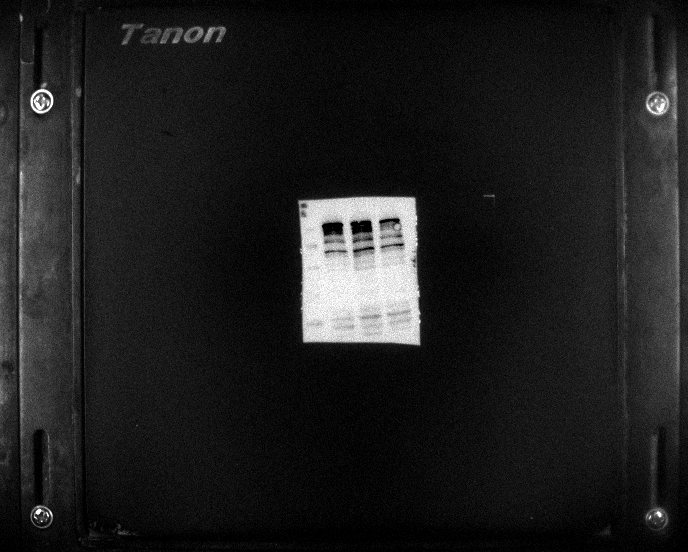

Supplement: Figure 3—source data 11. [file elife-88375-fig3-data11.zip › Figure 3-source data 11/IP His-IB His.tif]

F

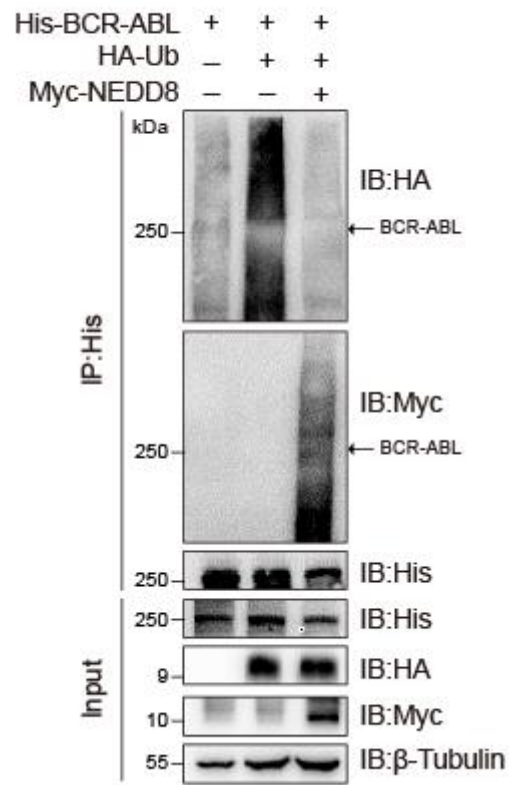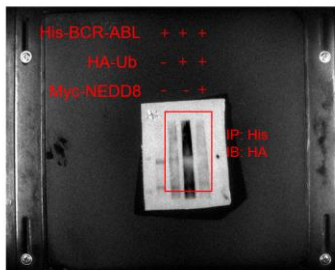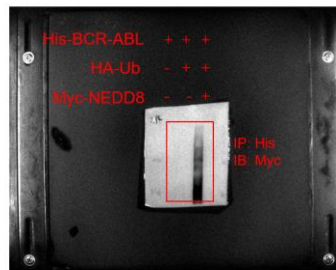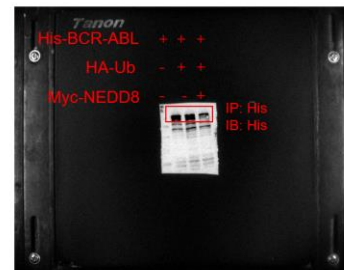

# Input

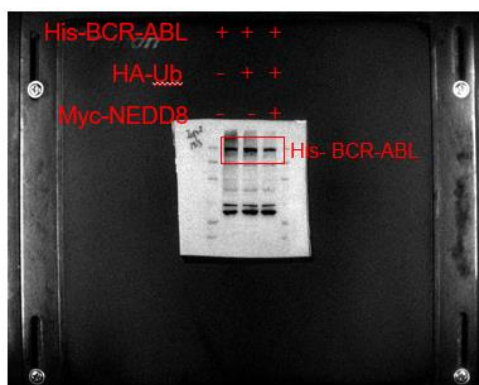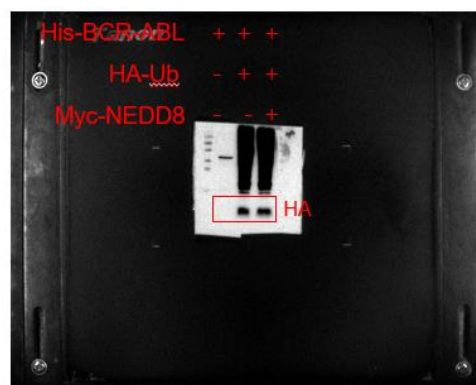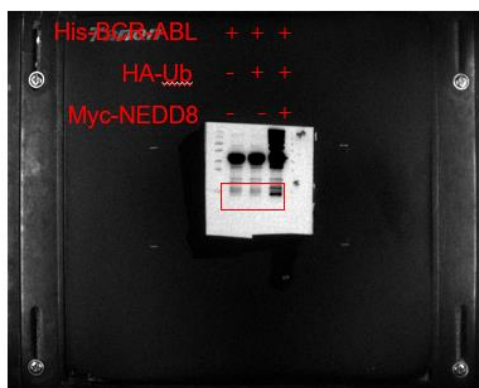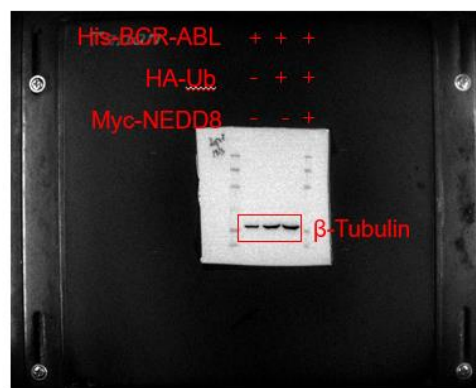

Supplement: Figure 3—source data 12. [file elife-88375-fig3-data12.zip › Figure 3-source data 12/Figure 3-source data 12.pdf]

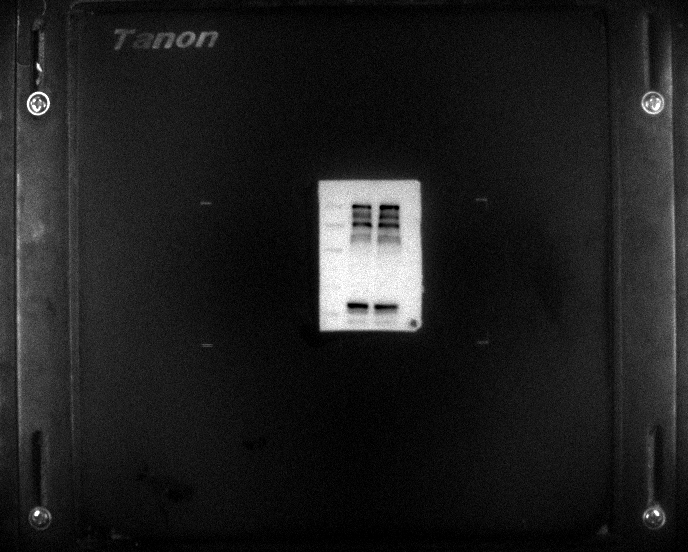

Supplement: Figure 3—source data 13. [file elife-88375-fig3-data13.zip › Figure 3-source data 13/K562 Input BCR-ABL.tif]

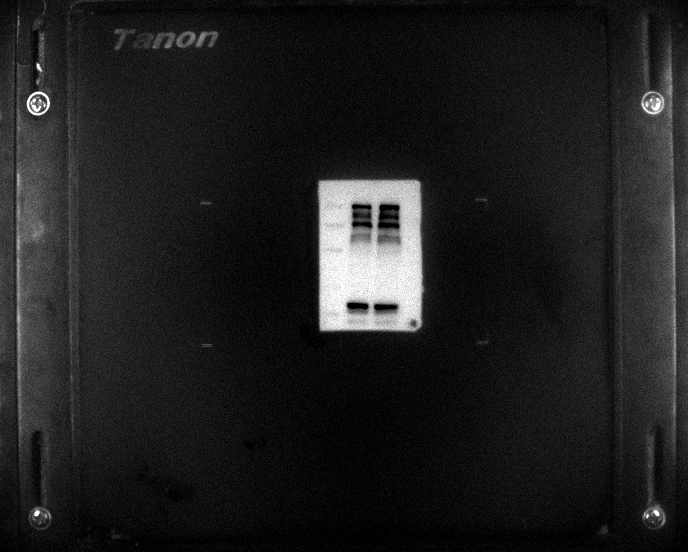

Supplement: Figure 3—source data 13. [file elife-88375-fig3-data13.zip › Figure 3-source data 13/K562 Input a┬-Tubulin.tif]

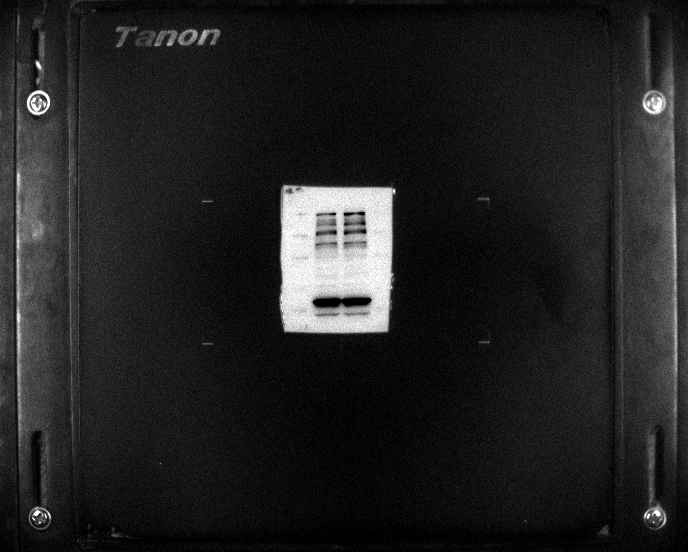

Supplement: Figure 3—source data 13. [file elife-88375-fig3-data13.zip › Figure 3-source data 13/K562 IP BCR-ABL-IB BCR-ABL.tif]

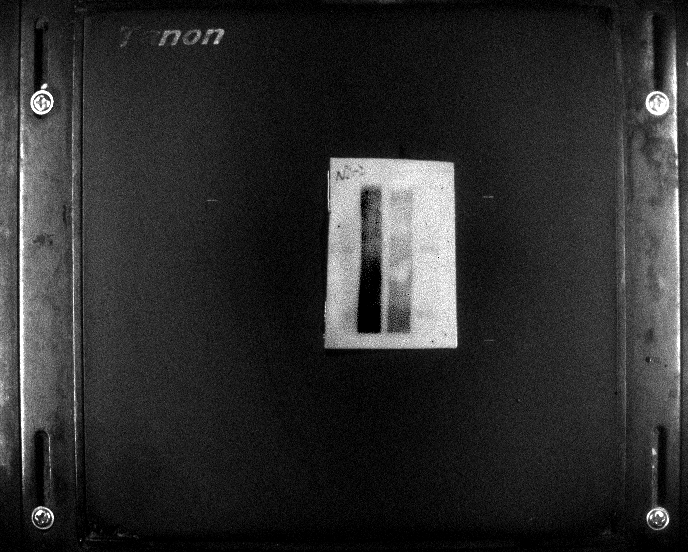

Supplement: Figure 3—source data 13. [file elife-88375-fig3-data13.zip › Figure 3-source data 13/K562 IP BCR-ABL-IB NEDD8.tif]

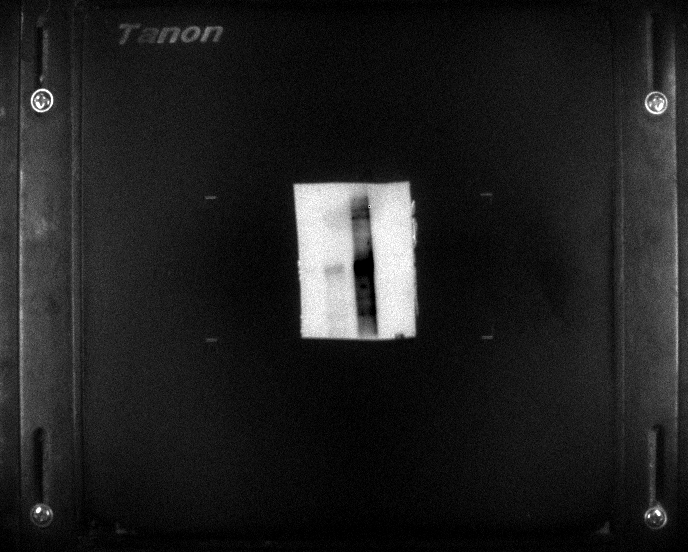

Supplement: Figure 3—source data 13. [file elife-88375-fig3-data13.zip › Figure 3-source data 13/K562 IP BCR-ABL-IB Ub.tif]

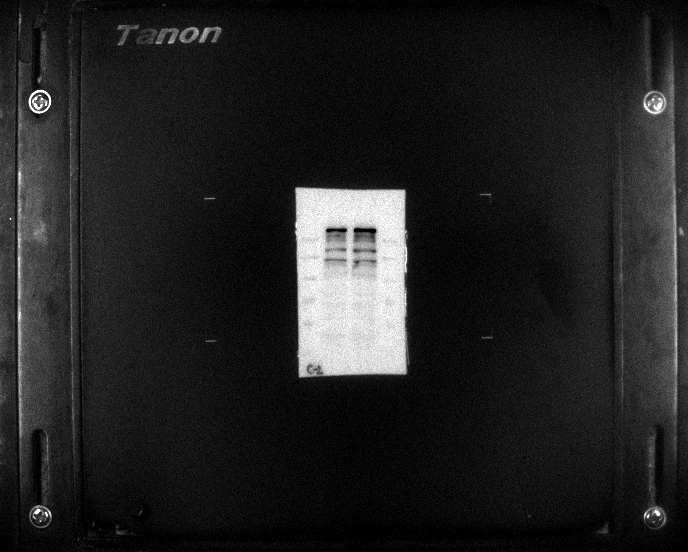

Supplement: Figure 3—source data 13. [file elife-88375-fig3-data13.zip › Figure 3-source data 13/MEG-01 Input BCR-ABL.tif]

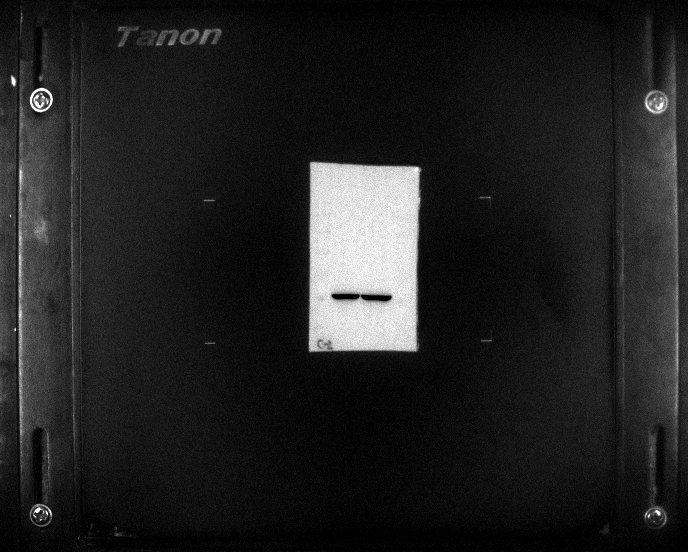

Supplement: Figure 3—source data 13. [file elife-88375-fig3-data13.zip › Figure 3-source data 13/MEG-01 Input a┬-Tubulin.tif]

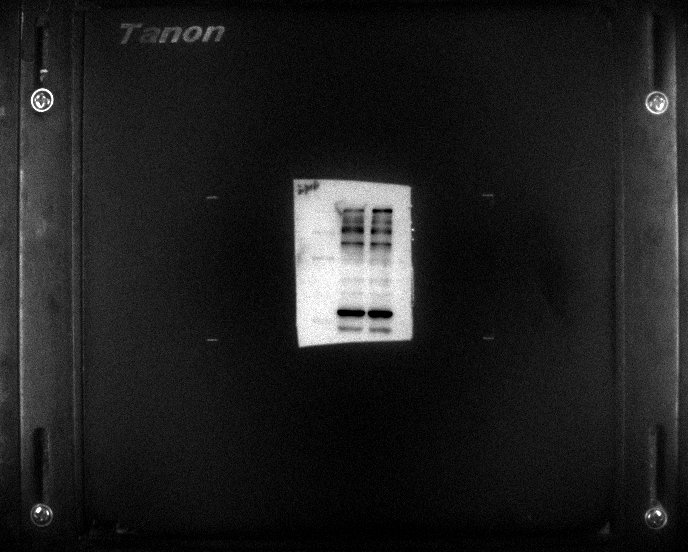

Supplement: Figure 3—source data 13. [file elife-88375-fig3-data13.zip › Figure 3-source data 13/MEG-01 IP BCR-ABL-IB BCR-ABL.tif]

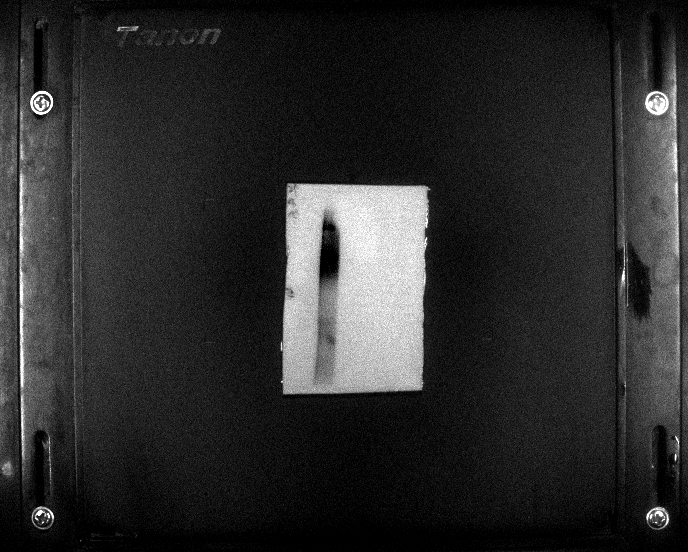

Supplement: Figure 3—source data 13. [file elife-88375-fig3-data13.zip › Figure 3-source data 13/MEG-01 IP BCR-ABL-IB NEDD8.tif]

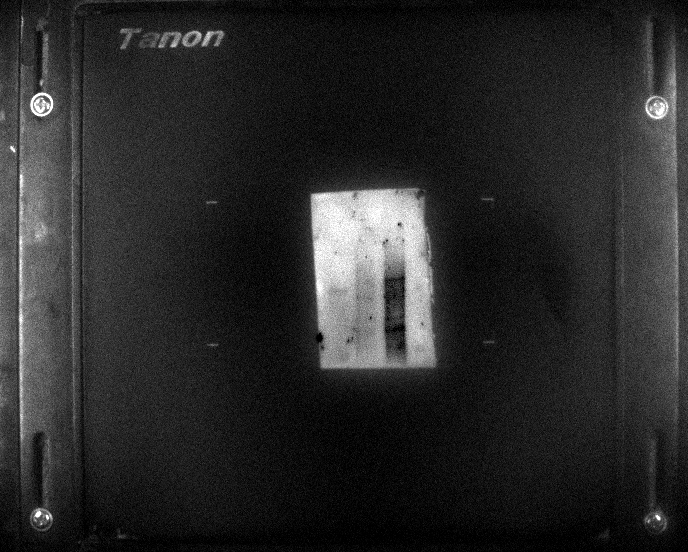

Supplement: Figure 3—source data 13. [file elife-88375-fig3-data13.zip › Figure 3-source data 13/MEG-01 IP BCR-ABL-IB Ub.tif]

G

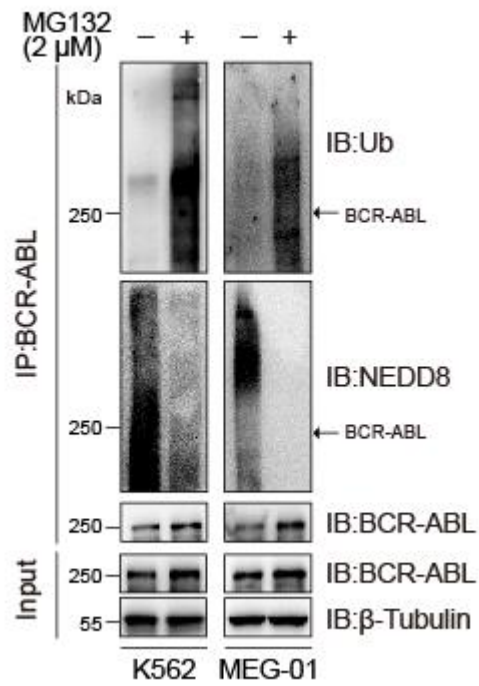

K562

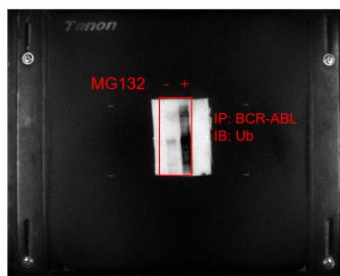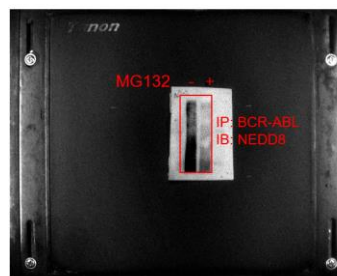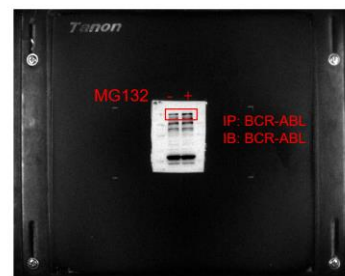

Input

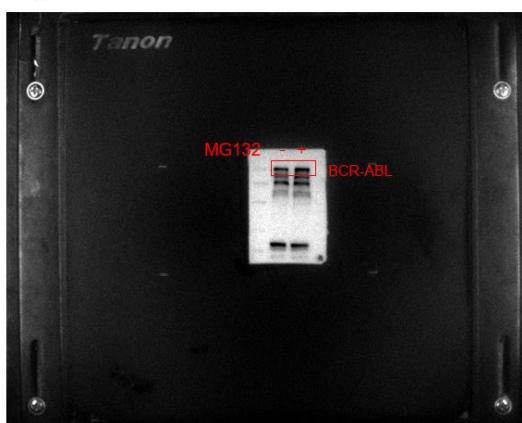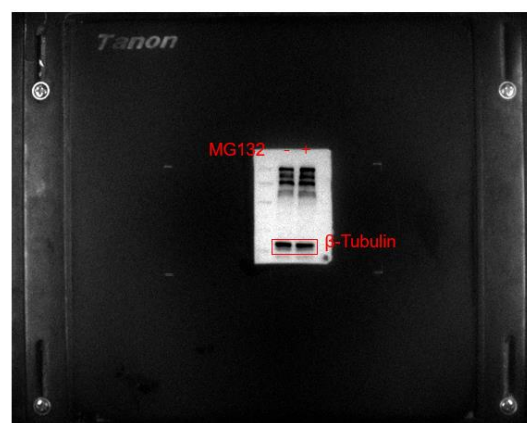

## MEG-01

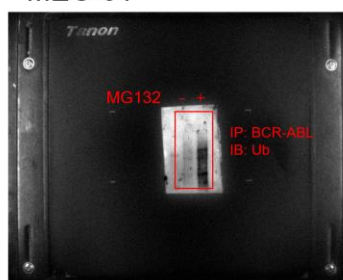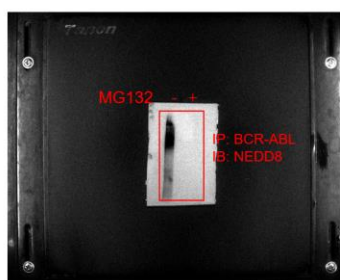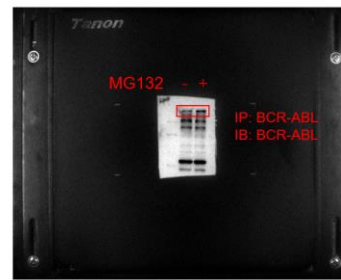

## Input

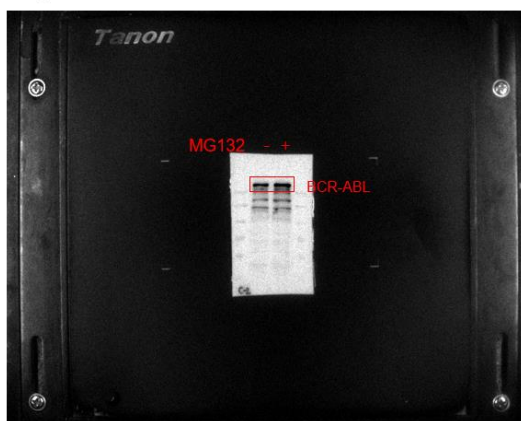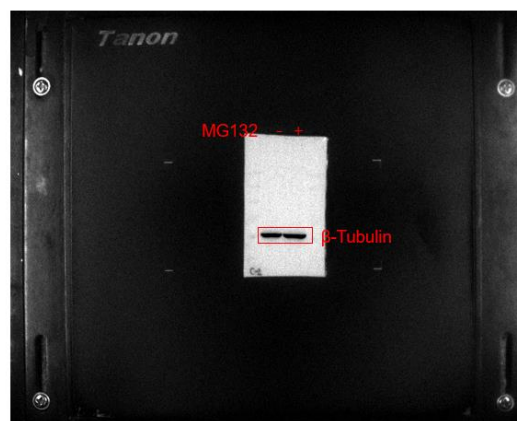

Supplement: Figure 3—source data 14. [file elife-88375-fig3-data14.zip › Figure 3-source data 14/Figure 3-source data 14.pdf]

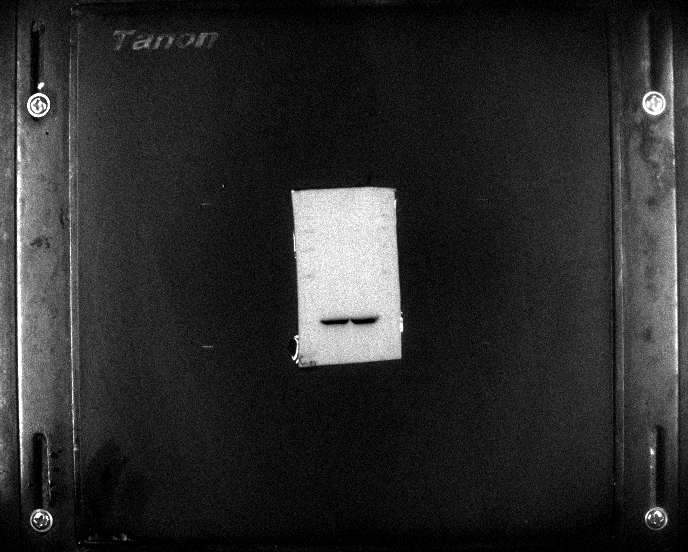

Supplement: Figure 3—source data 15. [file elife-88375-fig3-data15.zip › Figure 3-source data 15/K562 Input a┬-Tubulin.tif]

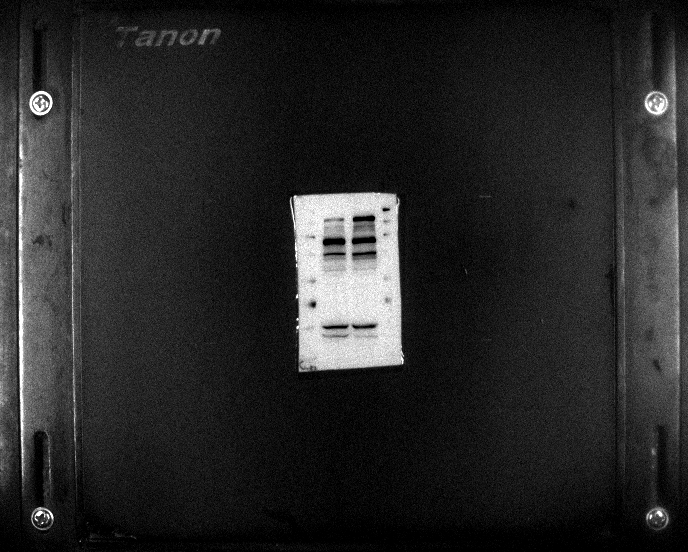

Supplement: Figure 3—source data 15. [file elife-88375-fig3-data15.zip › Figure 3-source data 15/K562 Input BCR-ABL.tif]

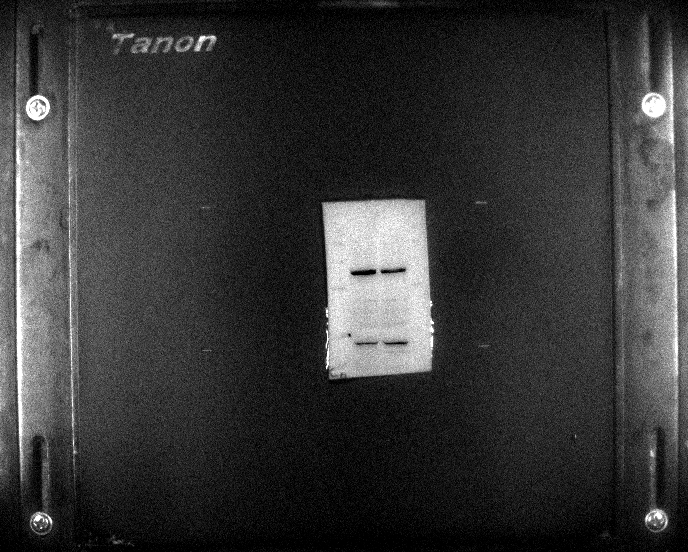

Supplement: Figure 3—source data 15. [file elife-88375-fig3-data15.zip › Figure 3-source data 15/K562 Input C-CBL.tif]

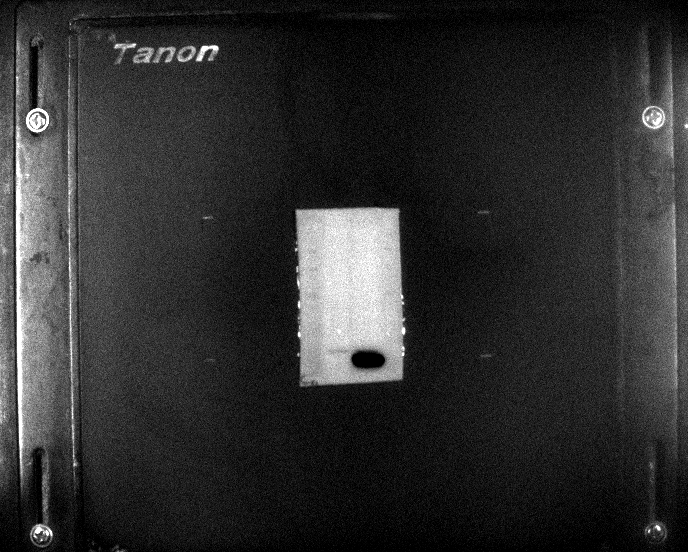

Supplement: Figure 3—source data 15. [file elife-88375-fig3-data15.zip › Figure 3-source data 15/K562 Input RAPSYN.tif]

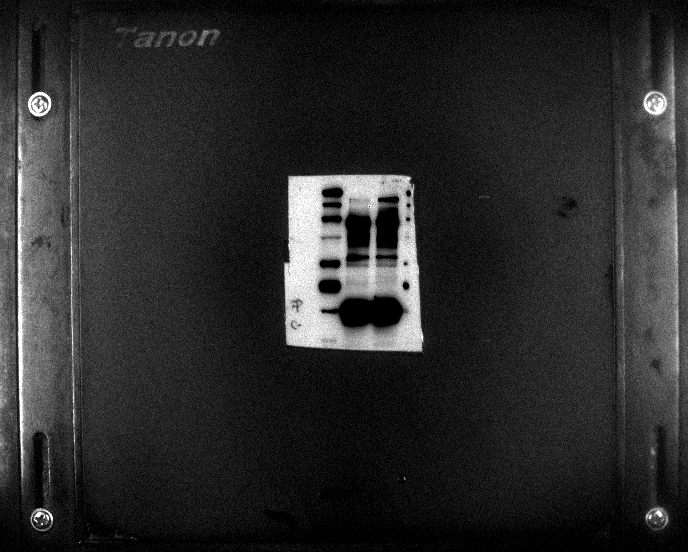

Supplement: Figure 3—source data 15. [file elife-88375-fig3-data15.zip › Figure 3-source data 15/K562 IP BCR-ABL-IB BCR-ABL.tif]

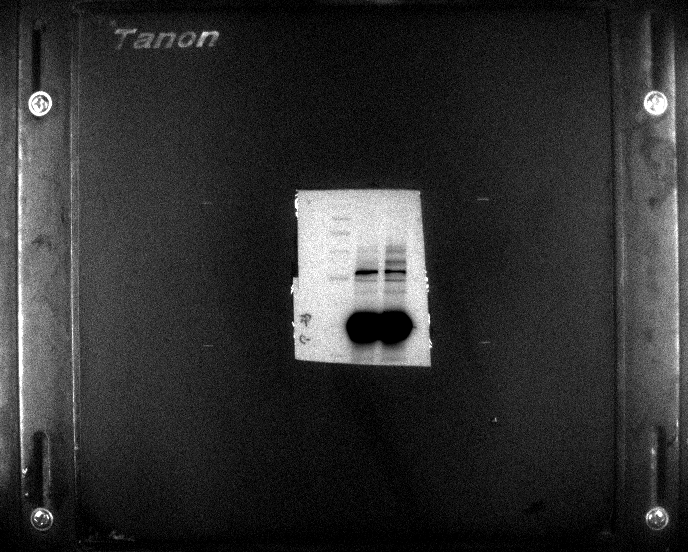

Supplement: Figure 3—source data 15. [file elife-88375-fig3-data15.zip › Figure 3-source data 15/K562 IP BCR-ABL-IB C-CBL.tif]

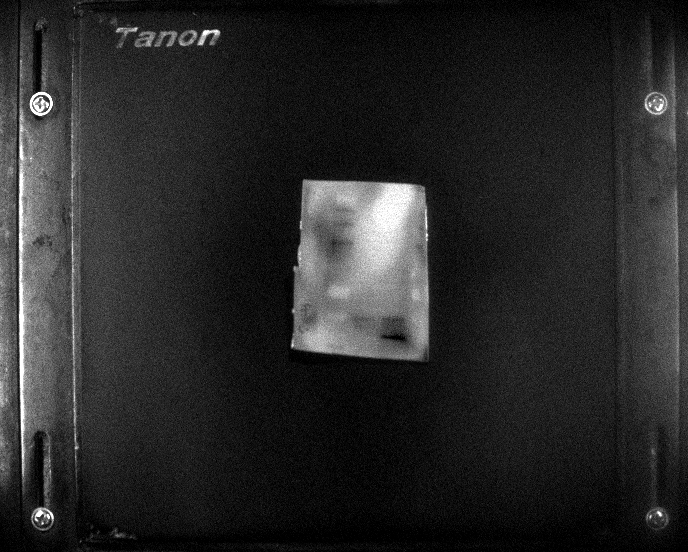

Supplement: Figure 3—source data 15. [file elife-88375-fig3-data15.zip › Figure 3-source data 15/K562 IP BCR-ABL-IB RAPSYN.tif]

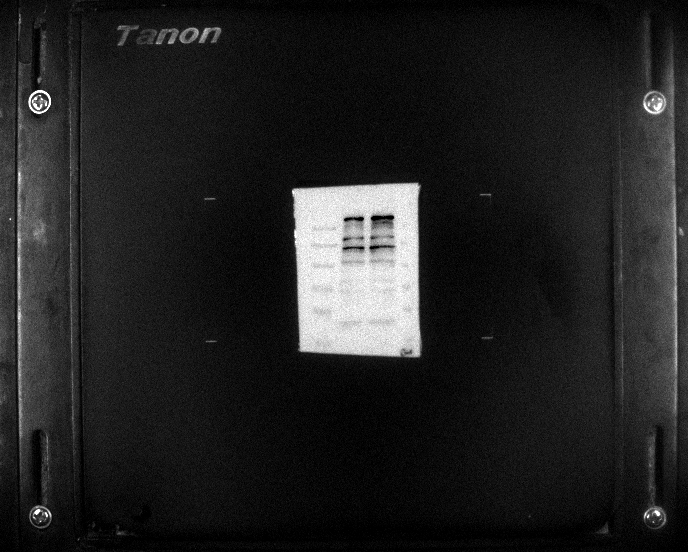

Supplement: Figure 3—source data 15. [file elife-88375-fig3-data15.zip › Figure 3-source data 15/MEG-01 Input BCR-ABL.tif]

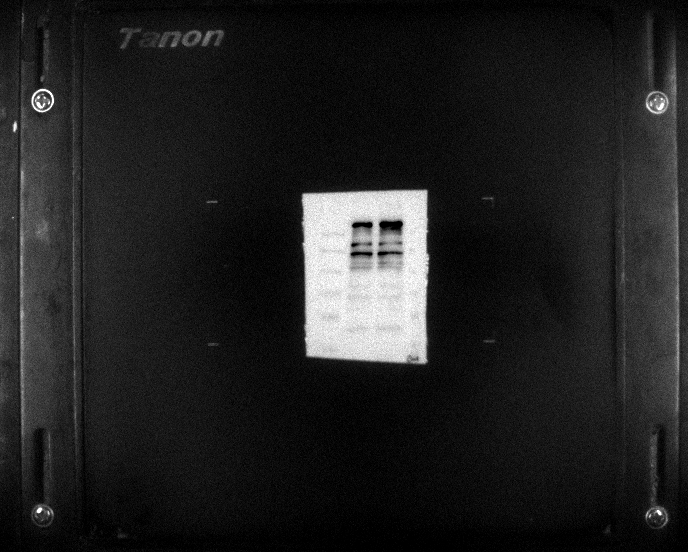

Supplement: Figure 3—source data 15. [file elife-88375-fig3-data15.zip › Figure 3-source data 15/MEG-01 Input C-CBL.tif]

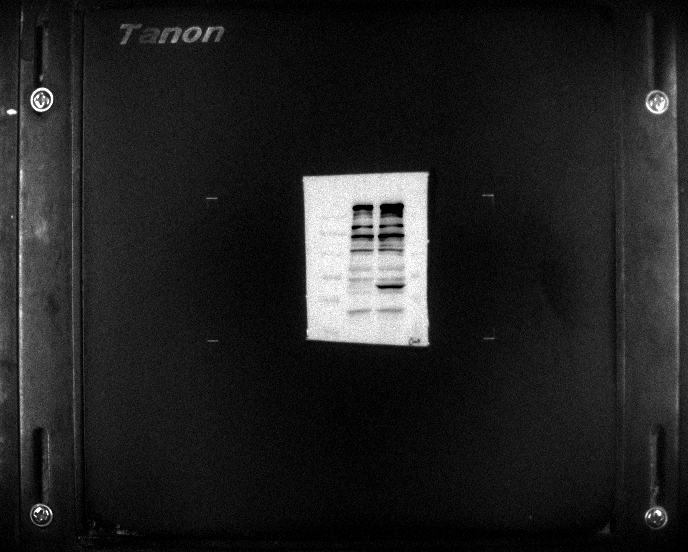

Supplement: Figure 3—source data 15. [file elife-88375-fig3-data15.zip › Figure 3-source data 15/MEG-01 Input RAPSYN.tif]

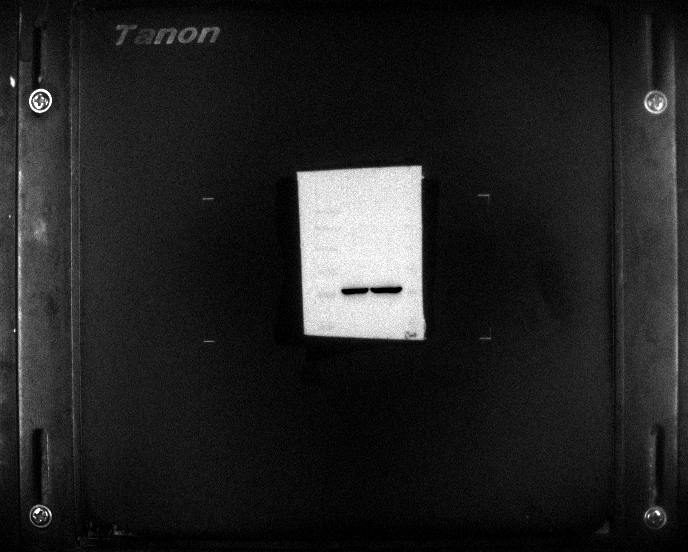

Supplement: Figure 3—source data 15. [file elife-88375-fig3-data15.zip › Figure 3-source data 15/MEG-01 Input a┬-Tubulin.tif]

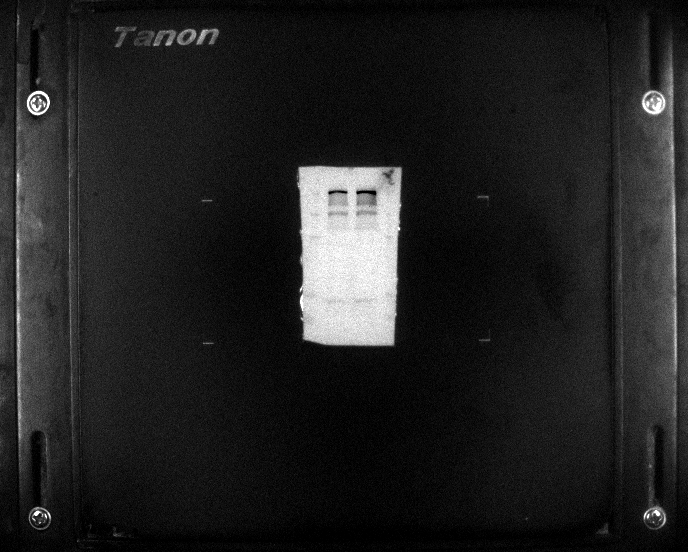

Supplement: Figure 3—source data 15. [file elife-88375-fig3-data15.zip › Figure 3-source data 15/MEG-01 IP BCR-ABL-IB BCR-ABL.tif]

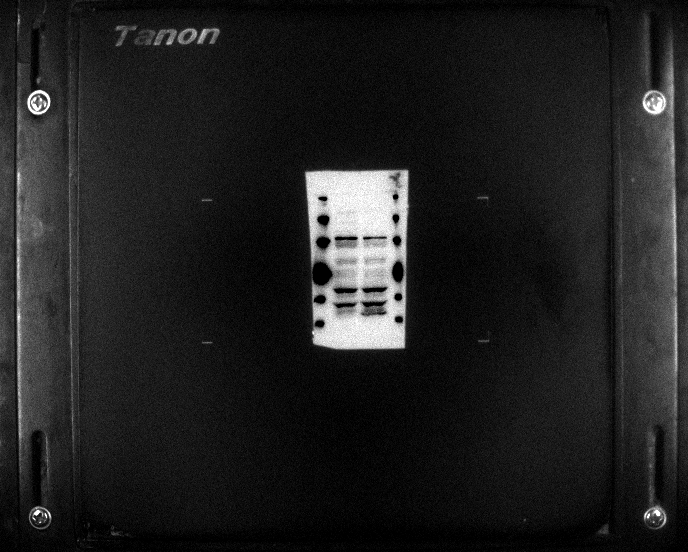

Supplement: Figure 3—source data 15. [file elife-88375-fig3-data15.zip › Figure 3-source data 15/MEG-01 IP BCR-ABL-IB C-CBL.tif]

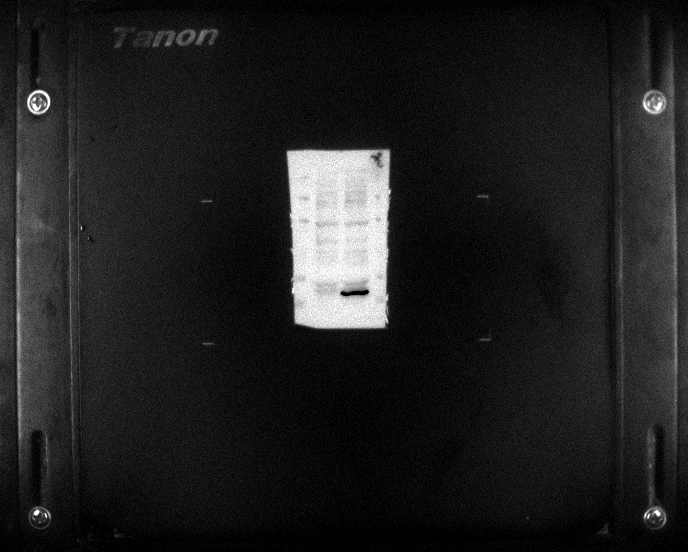

Supplement: Figure 3—source data 15. [file elife-88375-fig3-data15.zip › Figure 3-source data 15/MEG-01 IP BCR-ABL-IB RAPSYN.tif]

H

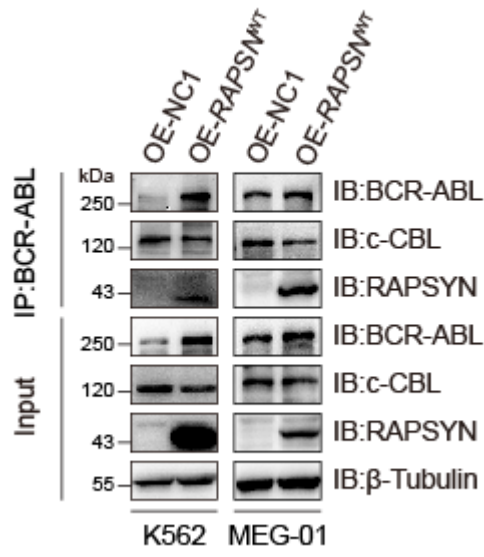

K562

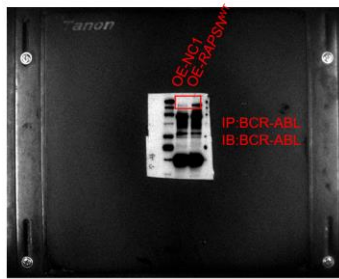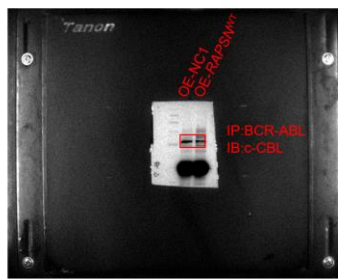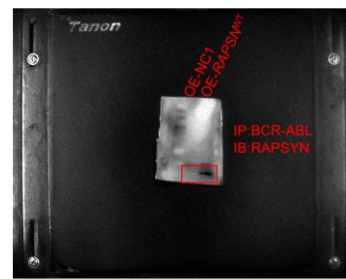

## Input

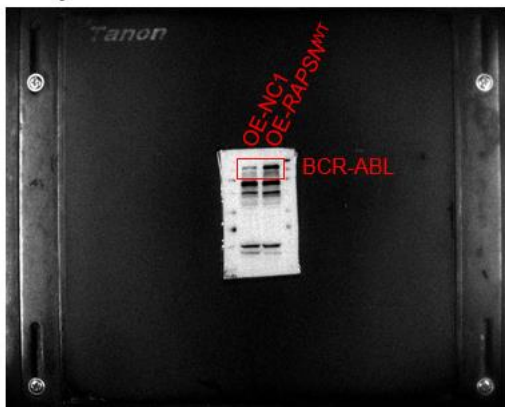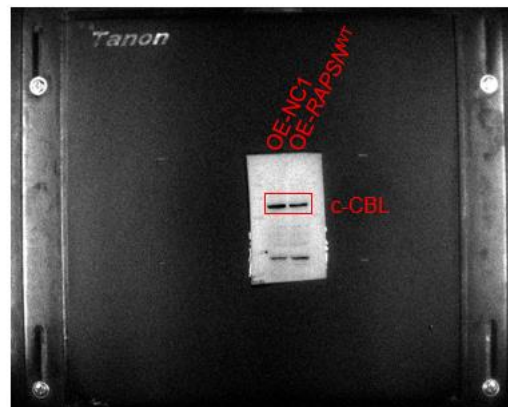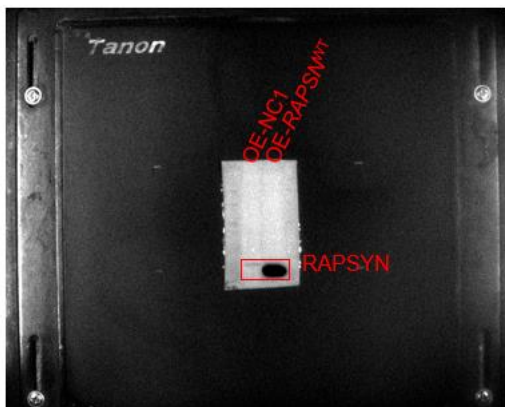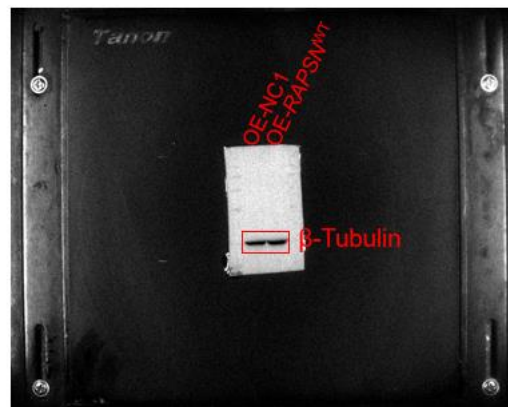

## MEG-01

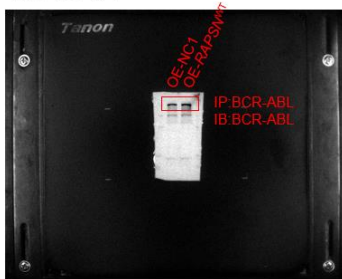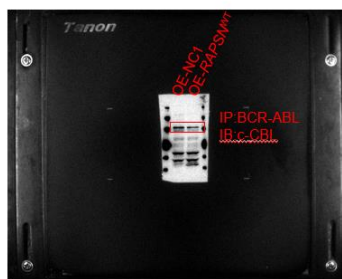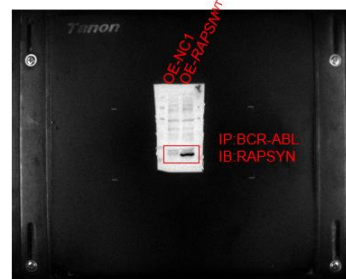

## Input

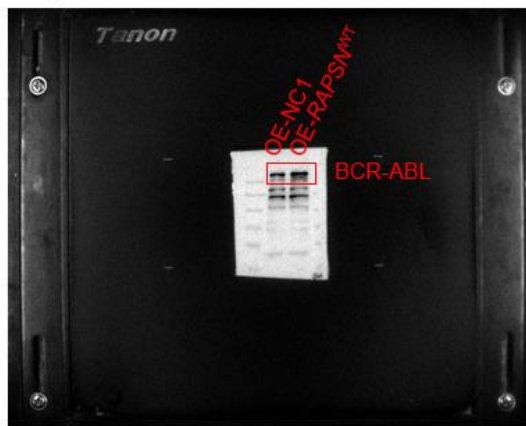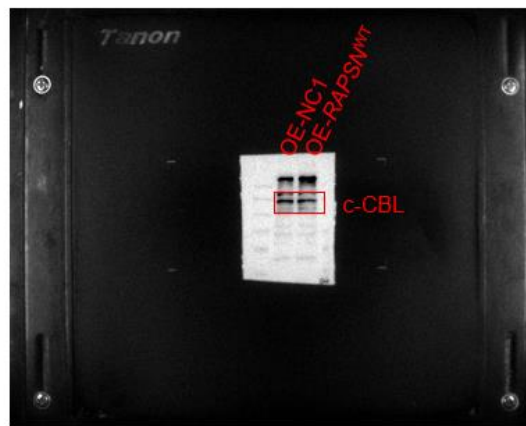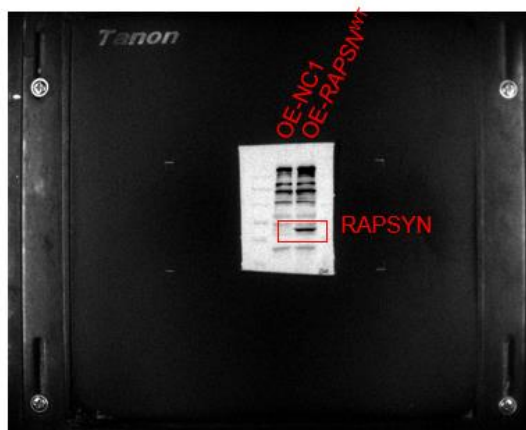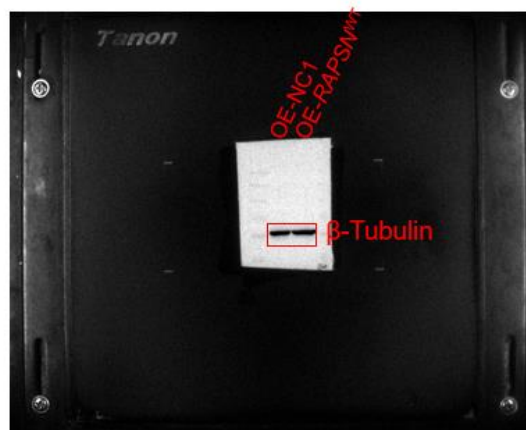

Supplement: Figure 3—source data 16. [file elife-88375-fig3-data16.zip › Figure 3-source data 16/Figure 3-source data 16.pdf]

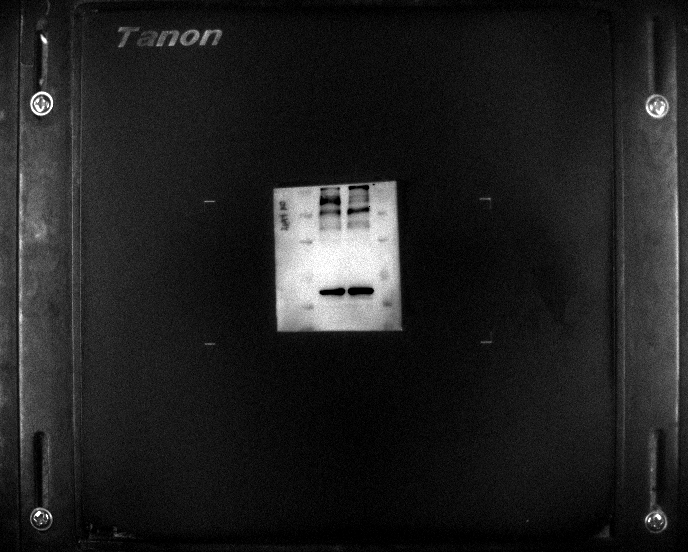

Supplement: Figure 3—source data 17. [file elife-88375-fig3-data17.zip › Figure 3-source data 17/Input BCR-ABL.tif]

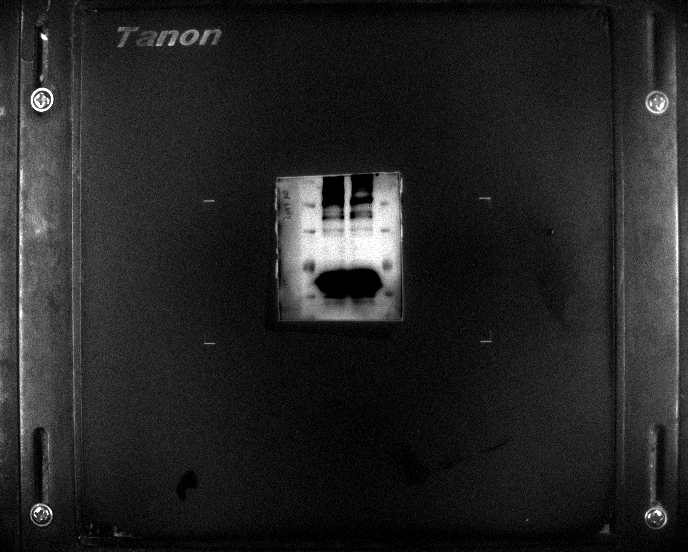

Supplement: Figure 3—source data 17. [file elife-88375-fig3-data17.zip › Figure 3-source data 17/Input c-CBL.tif]

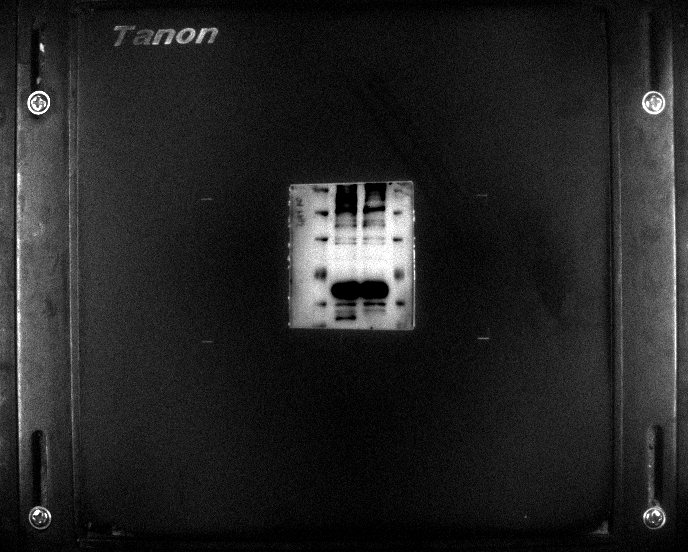

Supplement: Figure 3—source data 17. [file elife-88375-fig3-data17.zip › Figure 3-source data 17/Input RAPSYN.tif]

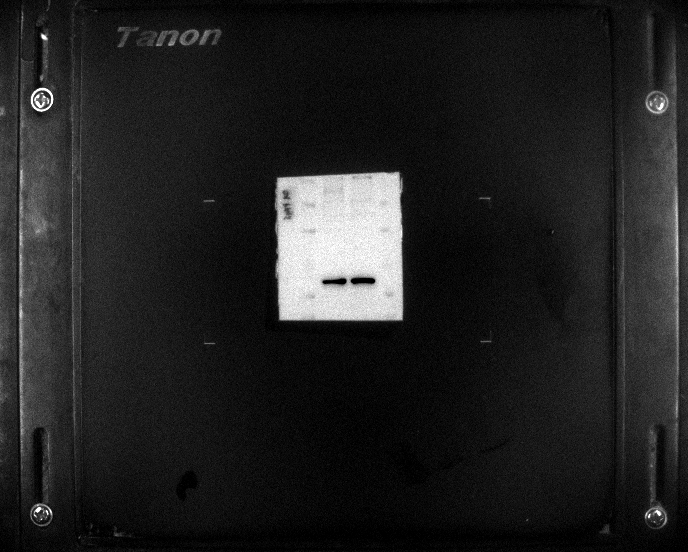

Supplement: Figure 3—source data 17. [file elife-88375-fig3-data17.zip › Figure 3-source data 17/Input a┬-Tubulin.tif]

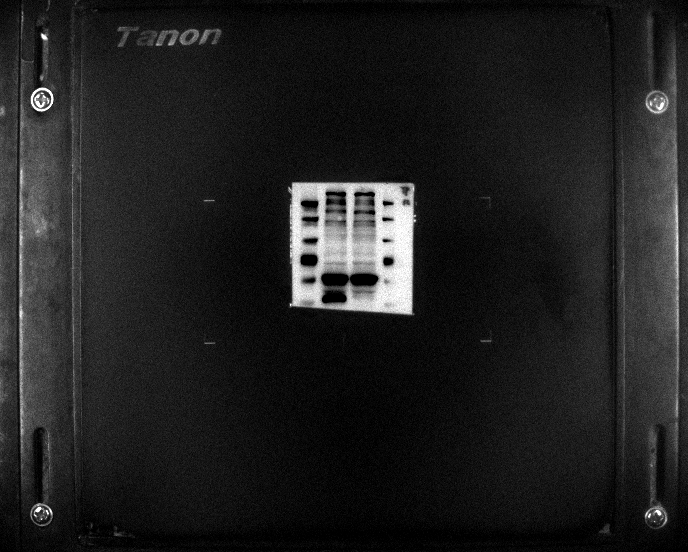

Supplement: Figure 3—source data 17. [file elife-88375-fig3-data17.zip › Figure 3-source data 17/IP BCR-ABL-IB BCR-ABL.tif]

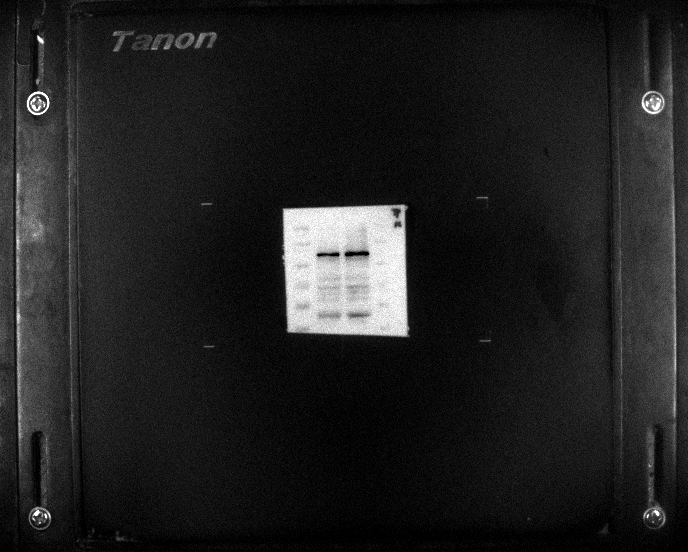

Supplement: Figure 3—source data 17. [file elife-88375-fig3-data17.zip › Figure 3-source data 17/IP BCR-ABL-IB C-CBL.tif]

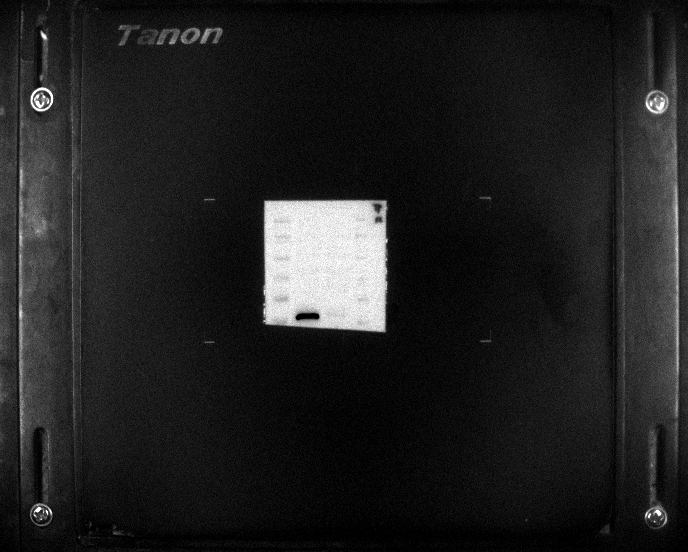

Supplement: Figure 3—source data 17. [file elife-88375-fig3-data17.zip › Figure 3-source data 17/IP BCR-ABL-IB RAPSYN.tif]

I

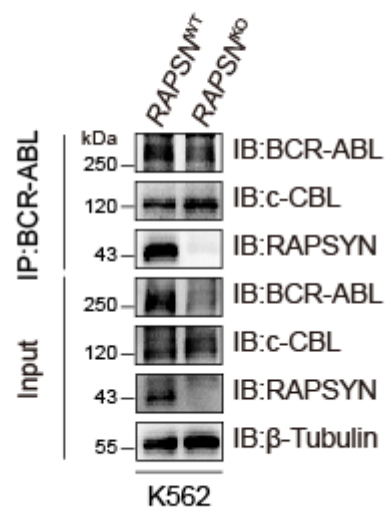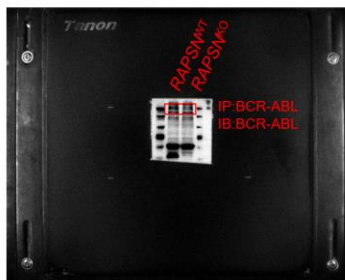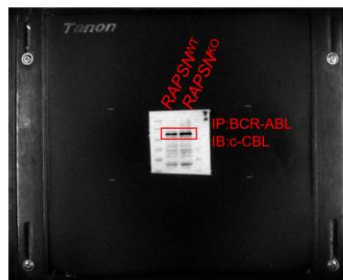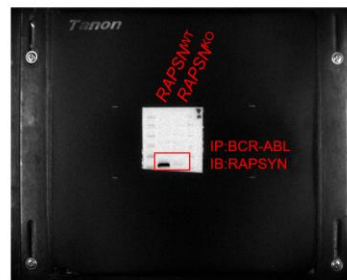

## Input

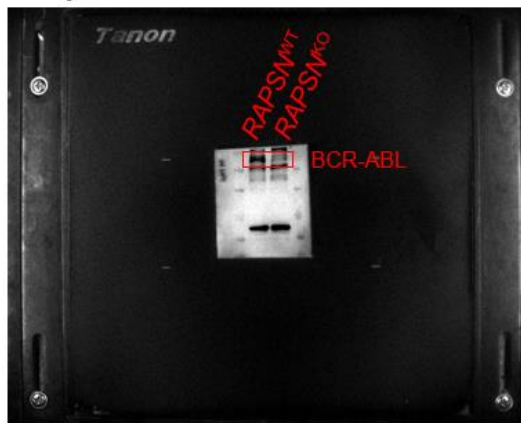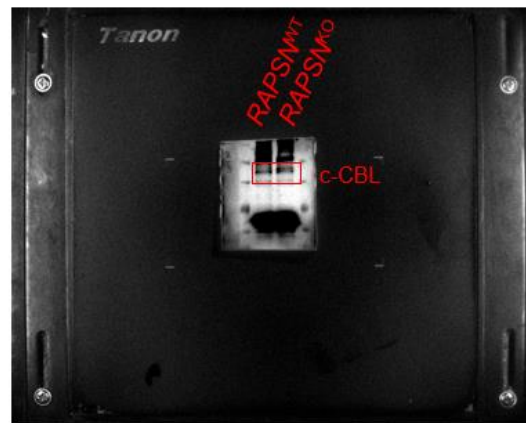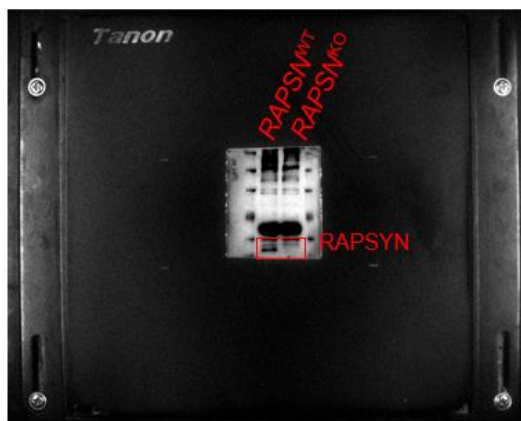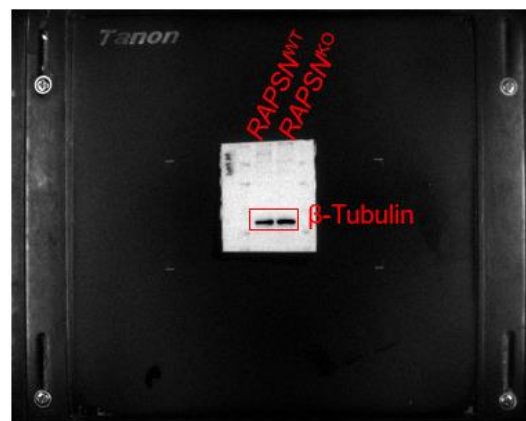

Supplement: Figure 3—source data 18. [file elife-88375-fig3-data18.zip › Figure 3-source data 18/Figure 3-source data 18.pdf]

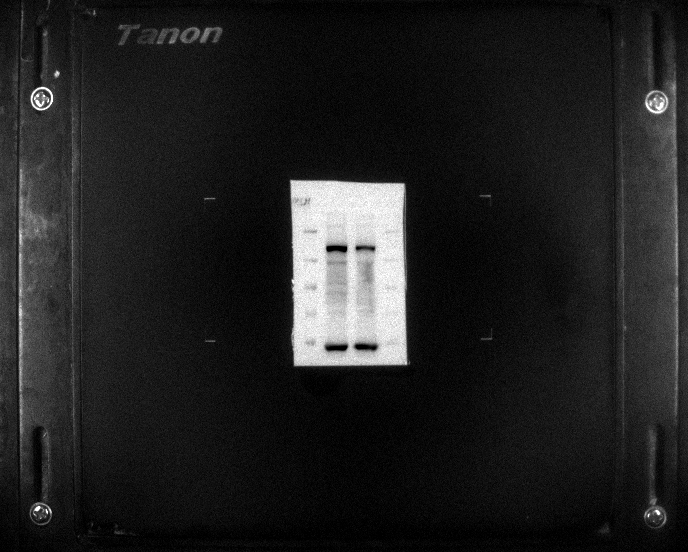

Supplement: Figure 3—figure supplement 1—source data 1. [file elife-88375-fig3-figsupp1-data1.zip › Figure supplement 4-source data 1/Jurkat BCR-ABL.tif]

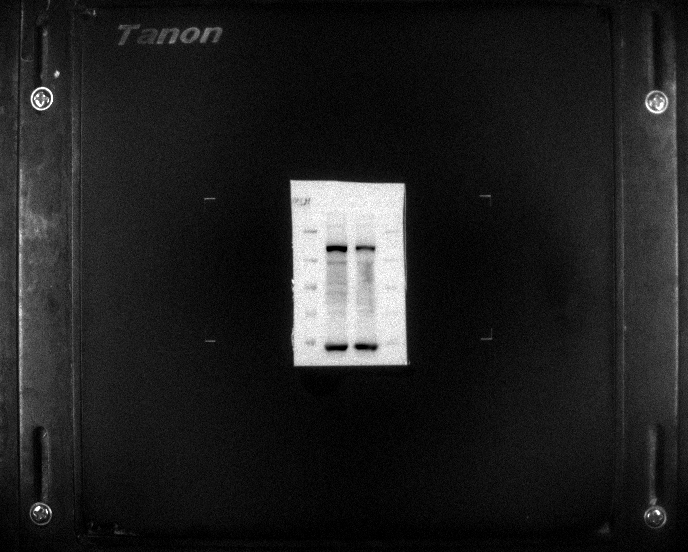

Supplement: Figure 3—figure supplement 1—source data 1. [file elife-88375-fig3-figsupp1-data1.zip › Figure supplement 4-source data 1/Jurkat a┬-Tubulin.tif]

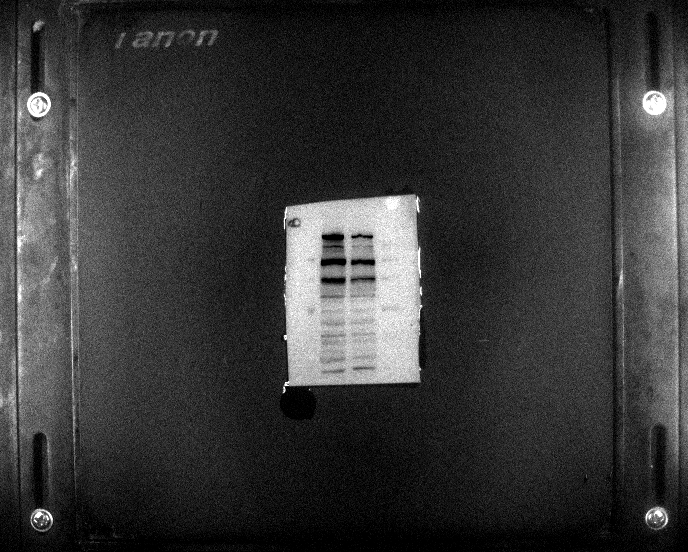

Supplement: Figure 3—figure supplement 1—source data 1. [file elife-88375-fig3-figsupp1-data1.zip › Figure supplement 4-source data 1/KU812 BCR-ABL.tif]

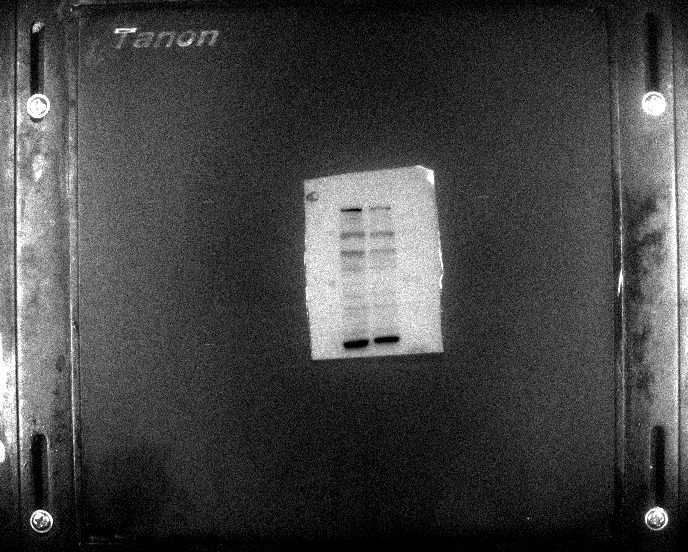

Supplement: Figure 3—figure supplement 1—source data 1. [file elife-88375-fig3-figsupp1-data1.zip › Figure supplement 4-source data 1/KU812 a┬-Tubulin.tif]

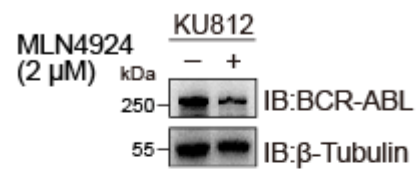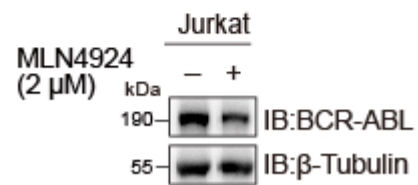

KU812

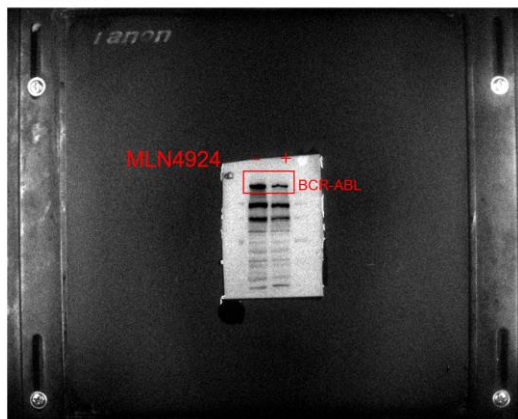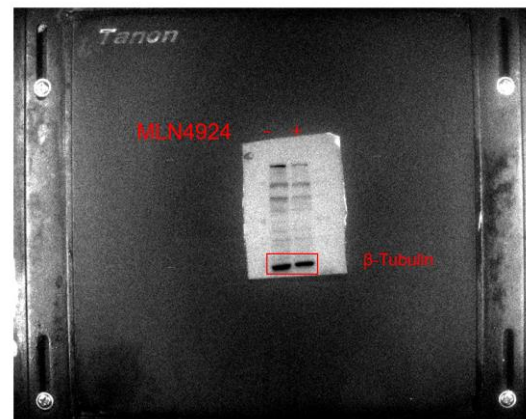

Jurkat

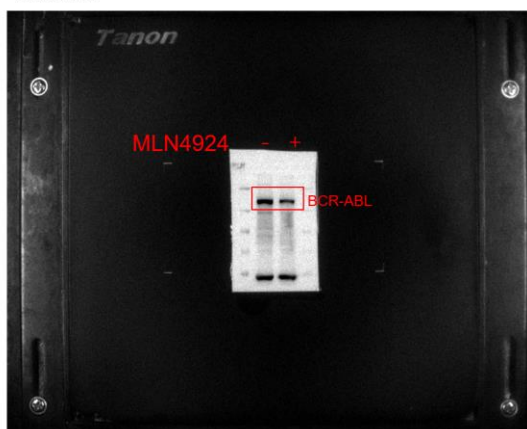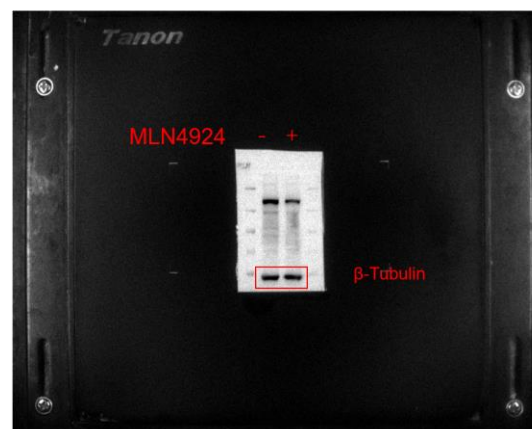

Supplement: Figure 3—figure supplement 1—source data 2. [file elife-88375-fig3-figsupp1-data2.zip › Figure supplement 4-source data 2/Figure supplement 4-source data 2.pdf]

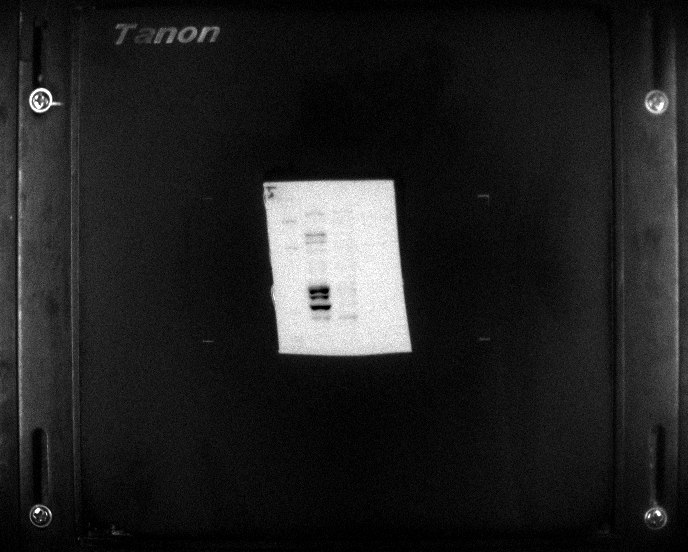

Supplement: Figure 4—source data 1. [file elife-88375-fig4-data1.zip › Figure 4-source data 1/K562 Input P-SRC.tif]

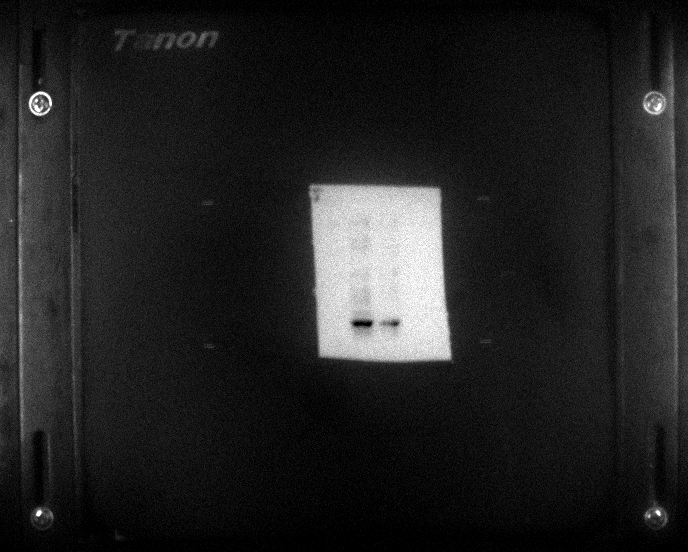

Supplement: Figure 4—source data 1. [file elife-88375-fig4-data1.zip › Figure 4-source data 1/K562 Input RAPSYN.tif]

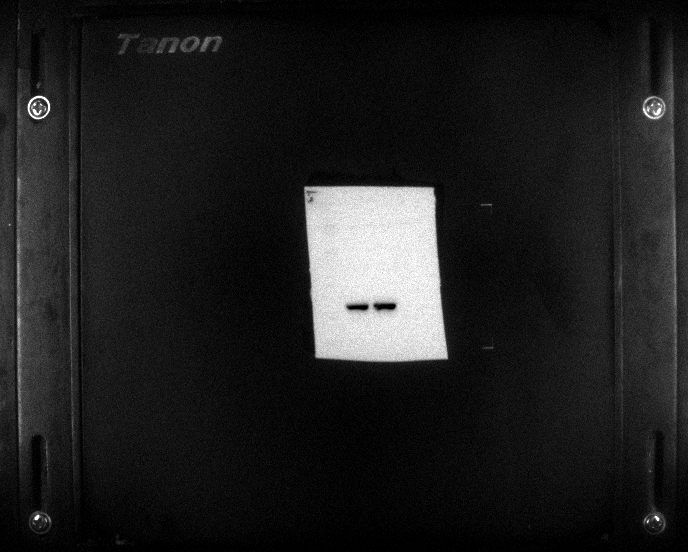

Supplement: Figure 4—source data 1. [file elife-88375-fig4-data1.zip › Figure 4-source data 1/K562 Input a┬-Tubulin.tif]

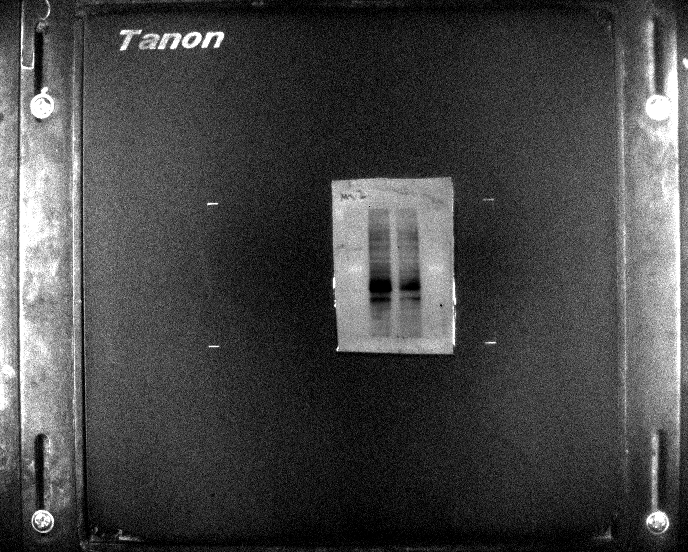

Supplement: Figure 4—source data 1. [file elife-88375-fig4-data1.zip › Figure 4-source data 1/K562 IP P-Tyr-IB RAPSYN.tif]

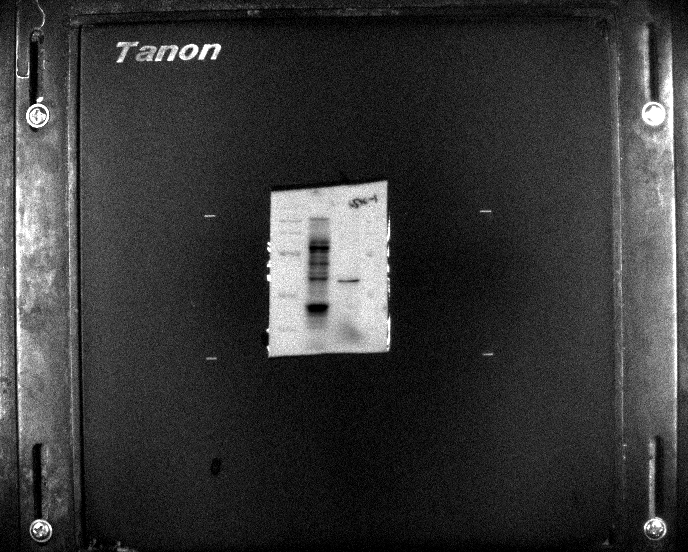

Supplement: Figure 4—source data 1. [file elife-88375-fig4-data1.zip › Figure 4-source data 1/K562 IP RAPSYN-IB P-Tyr.tif]

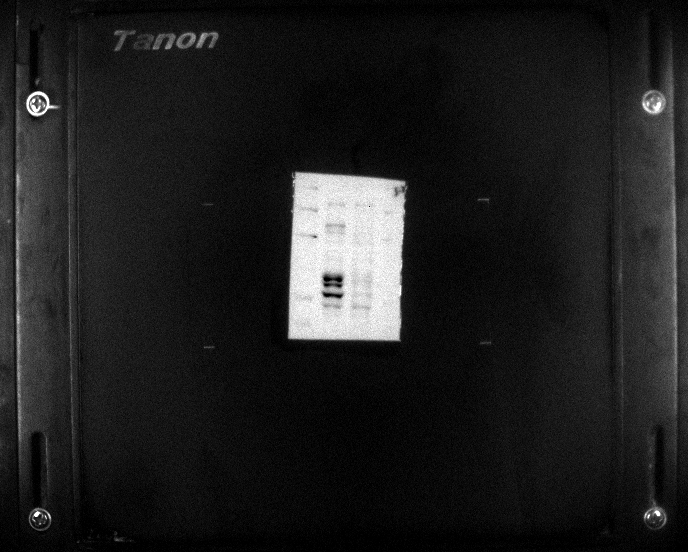

Supplement: Figure 4—source data 1. [file elife-88375-fig4-data1.zip › Figure 4-source data 1/MEG-01 Input P-SRC.tif]

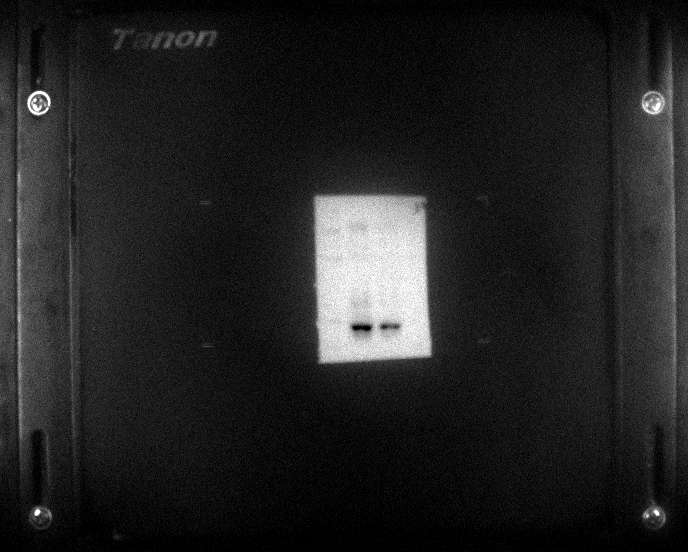

Supplement: Figure 4—source data 1. [file elife-88375-fig4-data1.zip › Figure 4-source data 1/MEG-01 Input RAPSYN.tif]

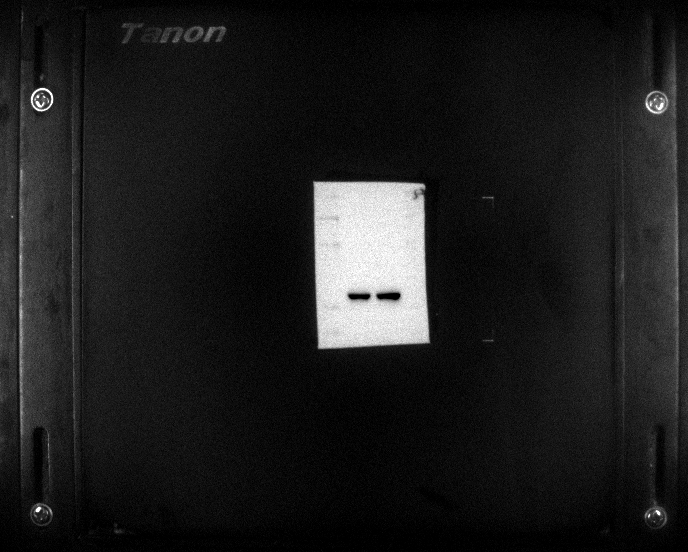

Supplement: Figure 4—source data 1. [file elife-88375-fig4-data1.zip › Figure 4-source data 1/MEG-01 Input a┬-Tubulin.tif]

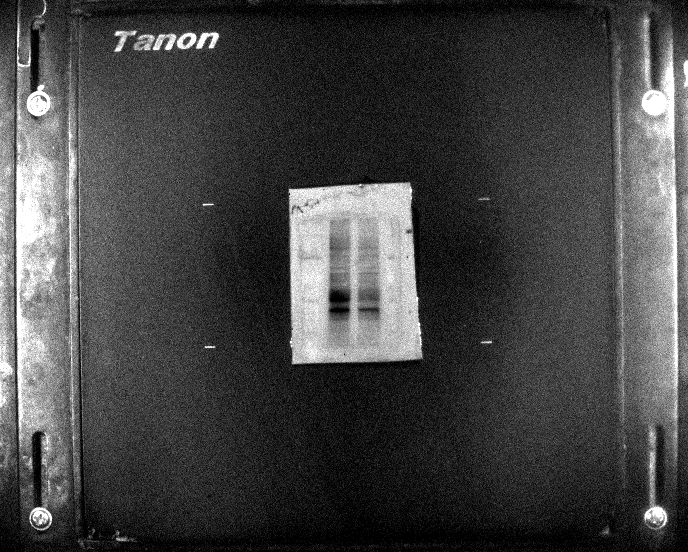

Supplement: Figure 4—source data 1. [file elife-88375-fig4-data1.zip › Figure 4-source data 1/MEG-01 IP p-Tyr-IB RAPSYN.tif]

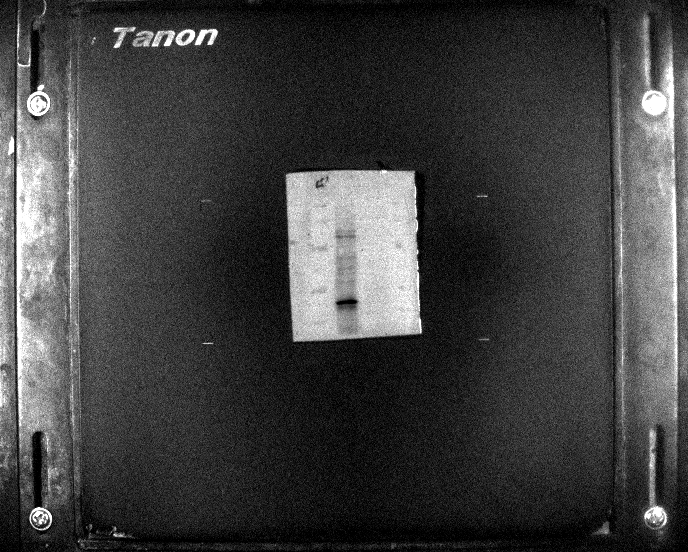

Supplement: Figure 4—source data 1. [file elife-88375-fig4-data1.zip › Figure 4-source data 1/MEG-01 IP RAPSYN-IB p-Tyr.tif]

A

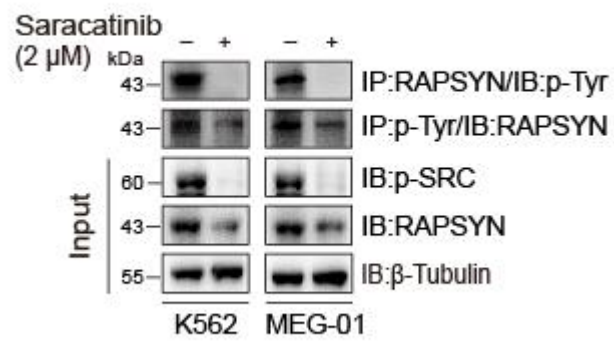

K562

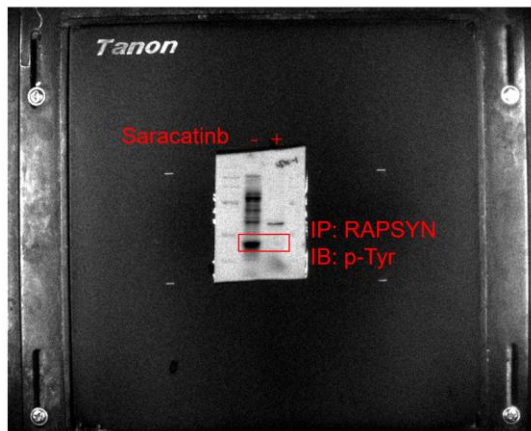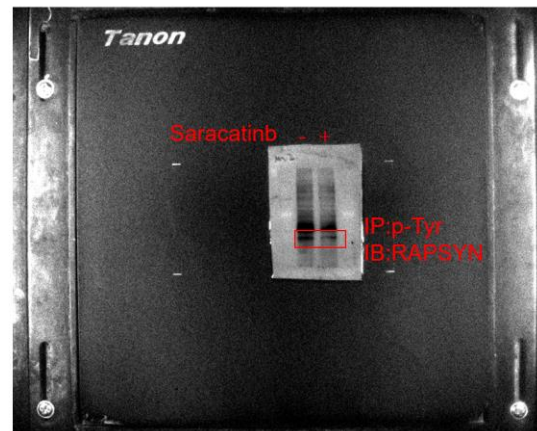

Input

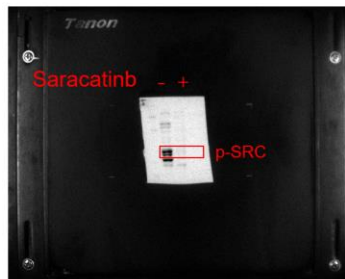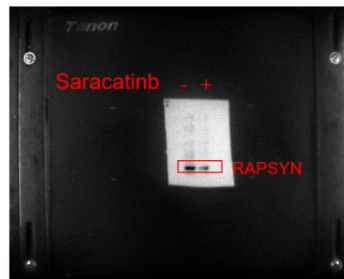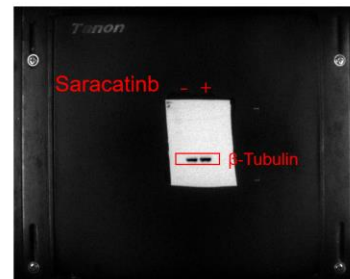

MEG-01

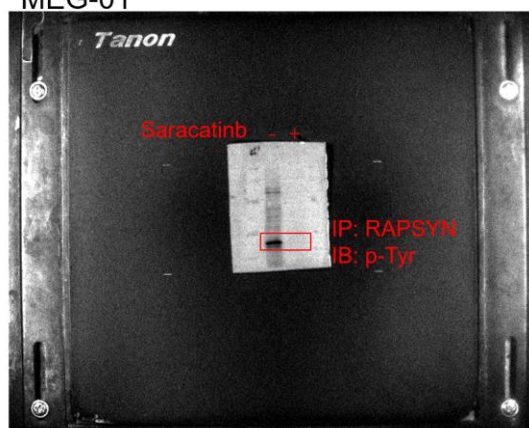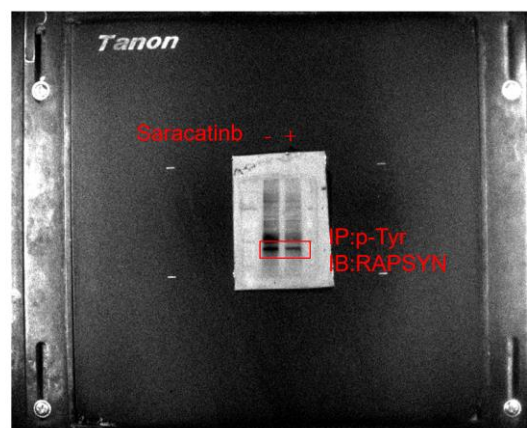

Input

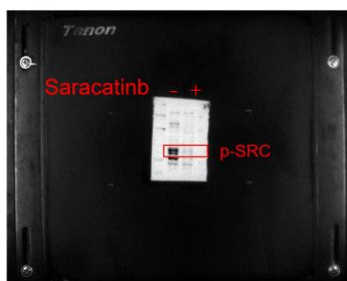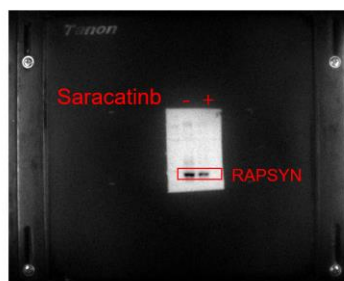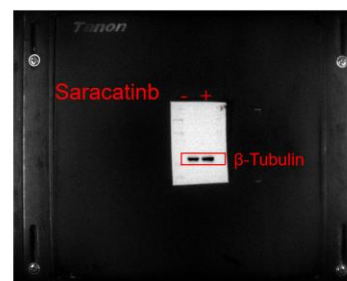

Supplement: Figure 4—source data 2. [file elife-88375-fig4-data2.zip › Figure 4-source data 2/Figure 4-source data 2.pdf]

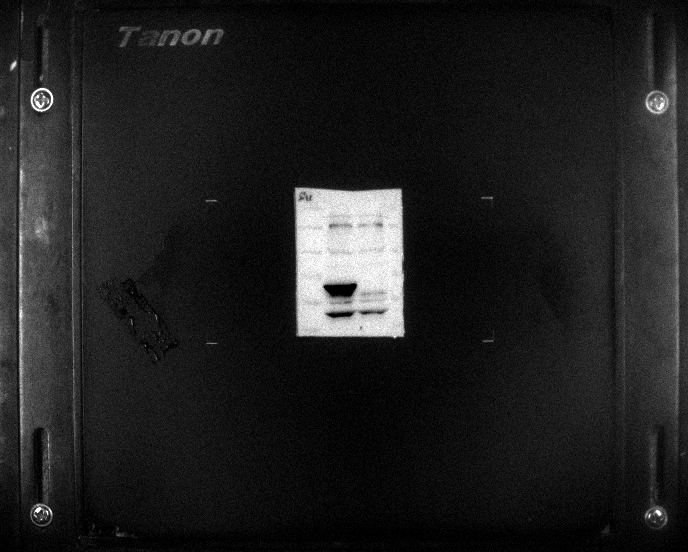

Supplement: Figure 4—source data 3. [file elife-88375-fig4-data3.zip › Figure 4-source data 3/K562 Input RAPSYN.tif]

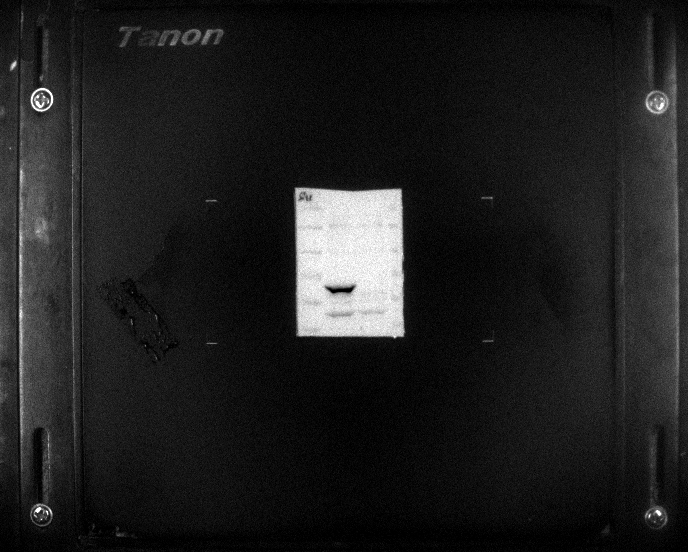

Supplement: Figure 4—source data 3. [file elife-88375-fig4-data3.zip › Figure 4-source data 3/K562 Input SRC.tif]

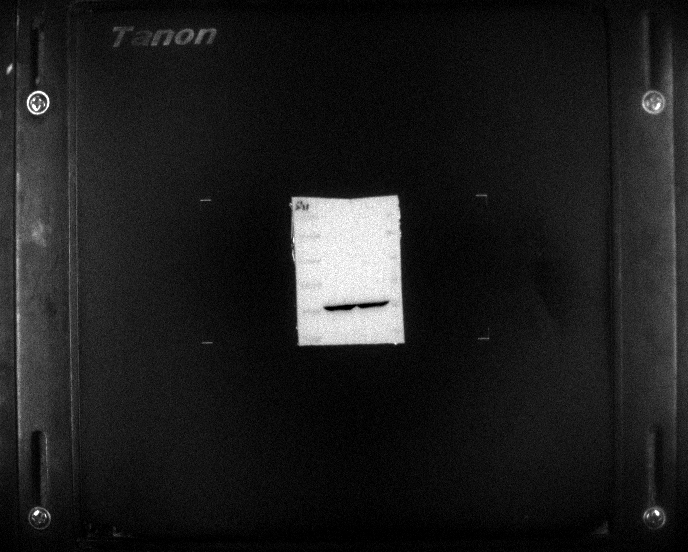

Supplement: Figure 4—source data 3. [file elife-88375-fig4-data3.zip › Figure 4-source data 3/K562 Input a┬-Tubulin.tif]

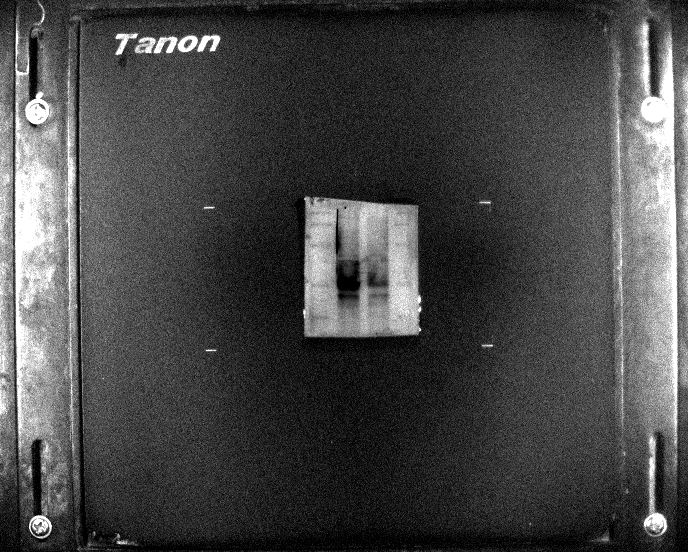

Supplement: Figure 4—source data 3. [file elife-88375-fig4-data3.zip › Figure 4-source data 3/K562 IP P-Tyr-IB-RAPSYN.tif]

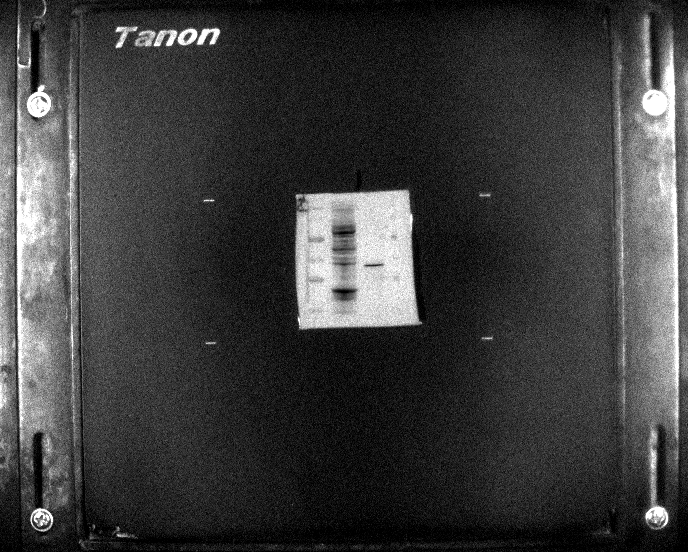

Supplement: Figure 4—source data 3. [file elife-88375-fig4-data3.zip › Figure 4-source data 3/K562 IP RAPSYN-IB P-Tyr.tif]

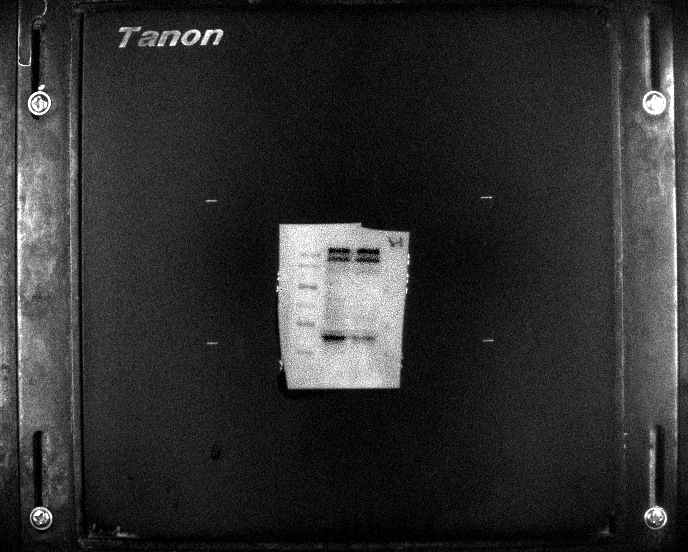

Supplement: Figure 4—source data 3. [file elife-88375-fig4-data3.zip › Figure 4-source data 3/MEG-01 Input RAPSYN.tif]

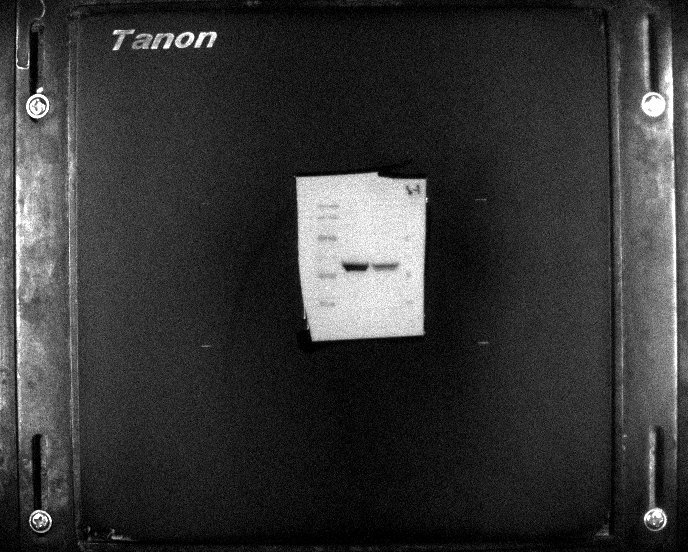

Supplement: Figure 4—source data 3. [file elife-88375-fig4-data3.zip › Figure 4-source data 3/MEG-01 Input SRC.tif]
