# Supplementary figures and images for: RAPSYN-mediated neddylation of BCR-ABL alternatively determines the fate of Philadelphia chromosome-positive leukemia (part 4 of 5)
Source: eLife. 2024 Jun 12;12:RP88375. doi: 10.7554/eLife.88375 (PMC11168747; doi:10.7554/eLife.88375)

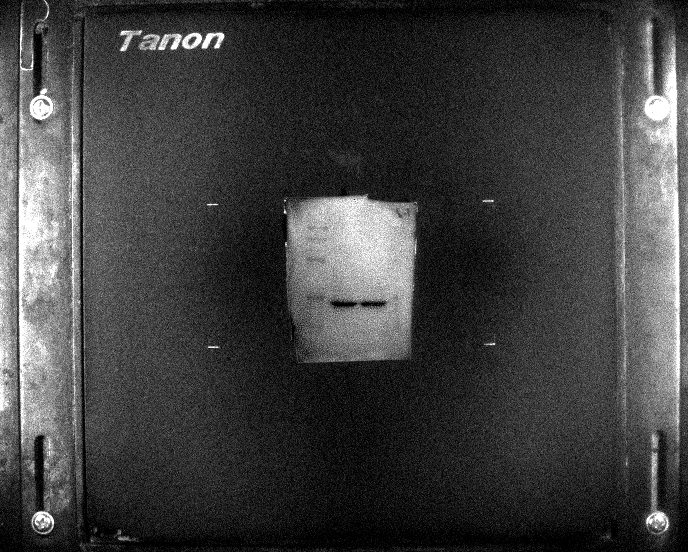

Supplement: Figure 4—source data 3. [file elife-88375-fig4-data3.zip › Figure 4-source data 3/MEG-01 Input a┬-Tubulin.tif]

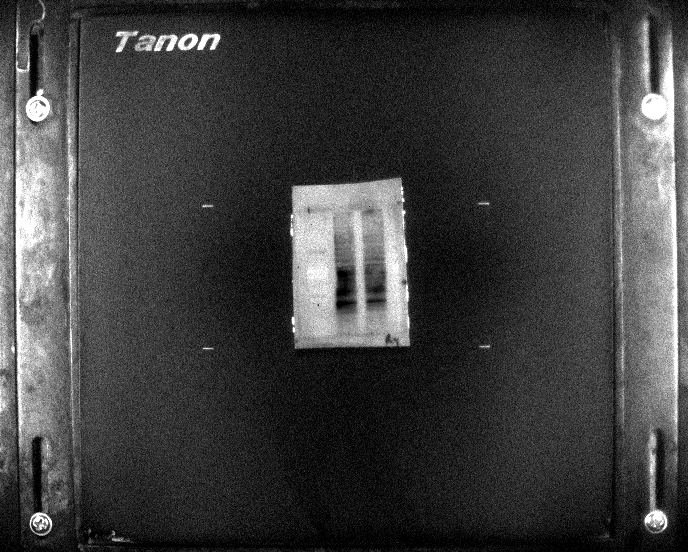

Supplement: Figure 4—source data 3. [file elife-88375-fig4-data3.zip › Figure 4-source data 3/MEG-01 IP P-Tyr-IB RAPSYN.tif]

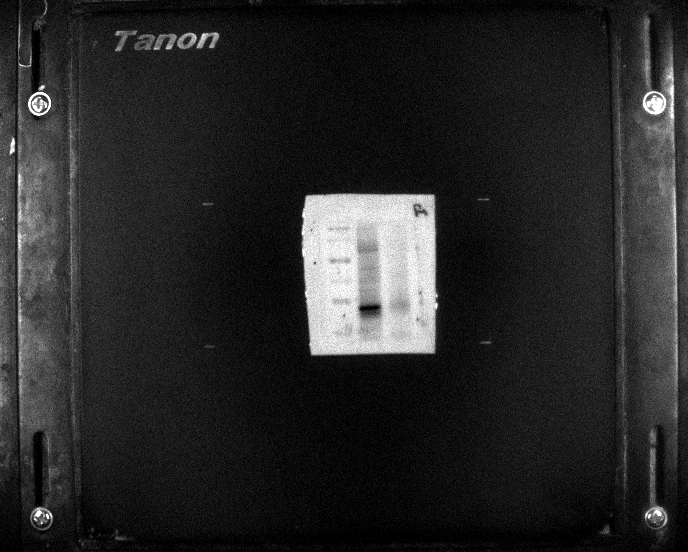

Supplement: Figure 4—source data 3. [file elife-88375-fig4-data3.zip › Figure 4-source data 3/MEG-01 IP RAPSYN-IB P-Tyr.tif]

B

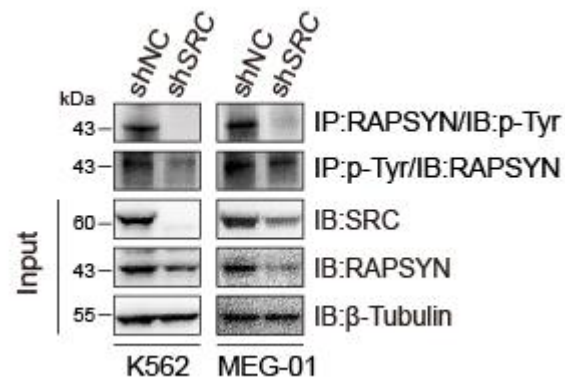

K562

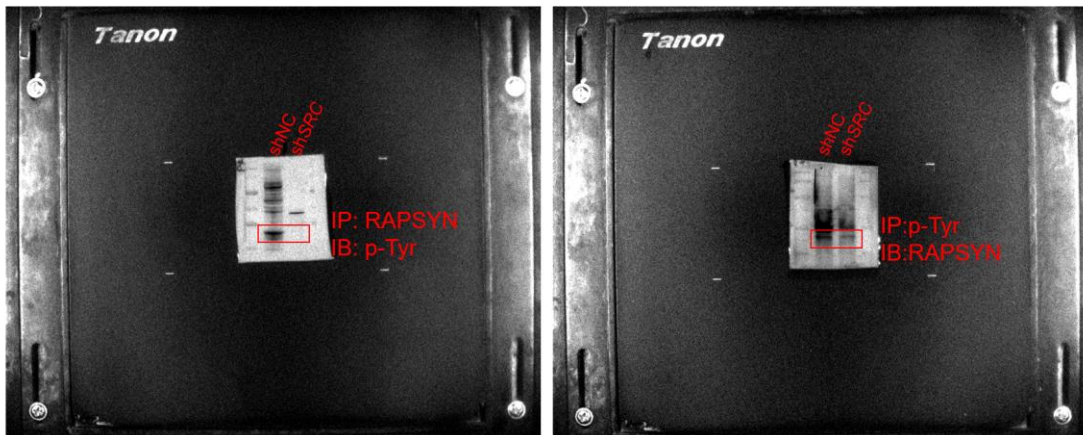

Input

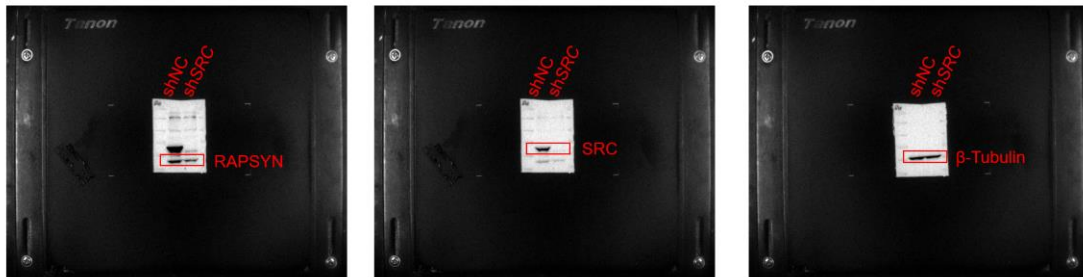

MEG-01

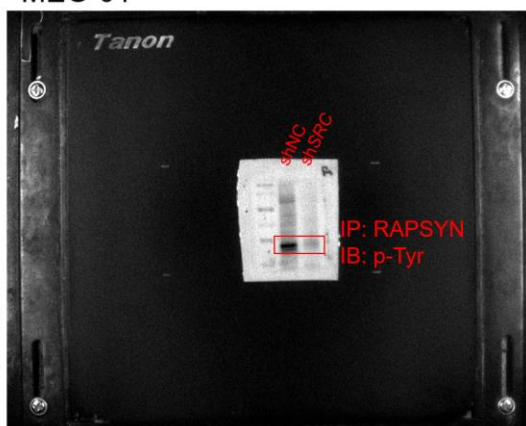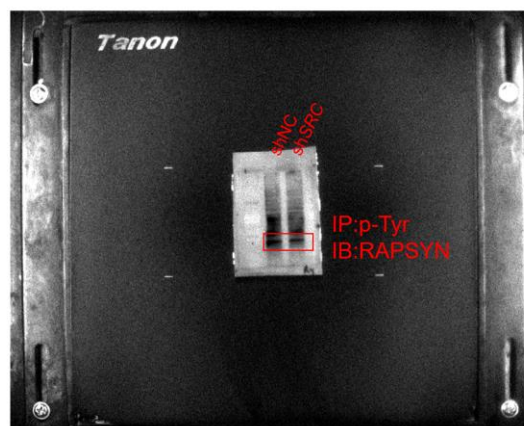

Input

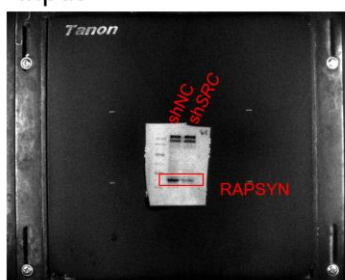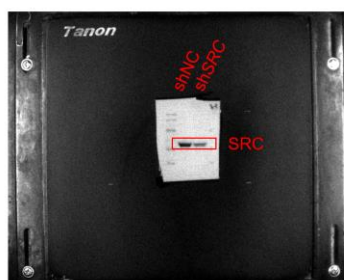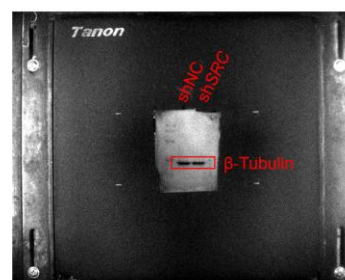

Supplement: Figure 4—source data 4. [file elife-88375-fig4-data4.zip › Figure 4-source data 4/Figure 4-source data 4.pdf]

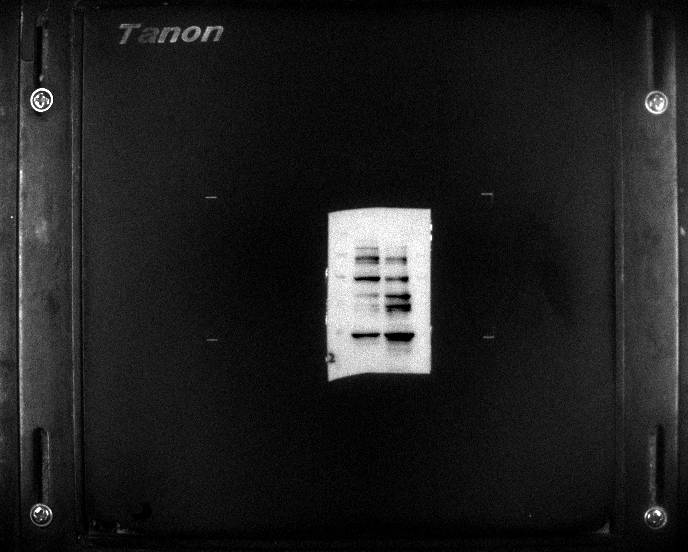

Supplement: Figure 4—source data 5. [file elife-88375-fig4-data5.zip › Figure 4-source data 5/K562 Input RAPSYN.tif]

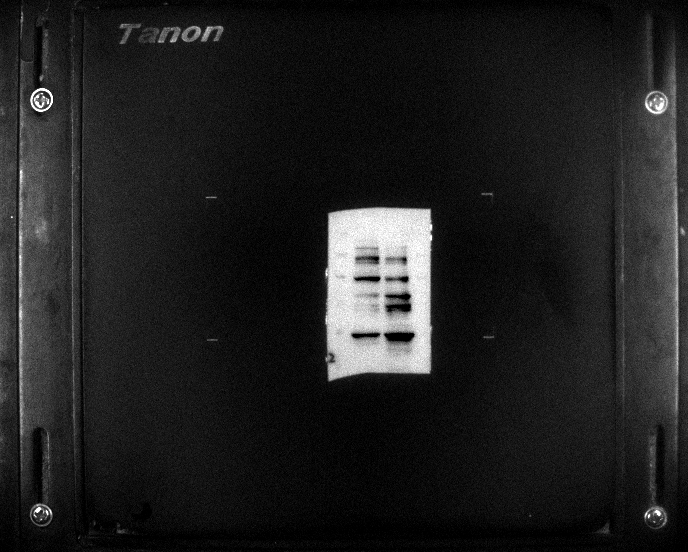

Supplement: Figure 4—source data 5. [file elife-88375-fig4-data5.zip › Figure 4-source data 5/K562 Input SRC.tif]

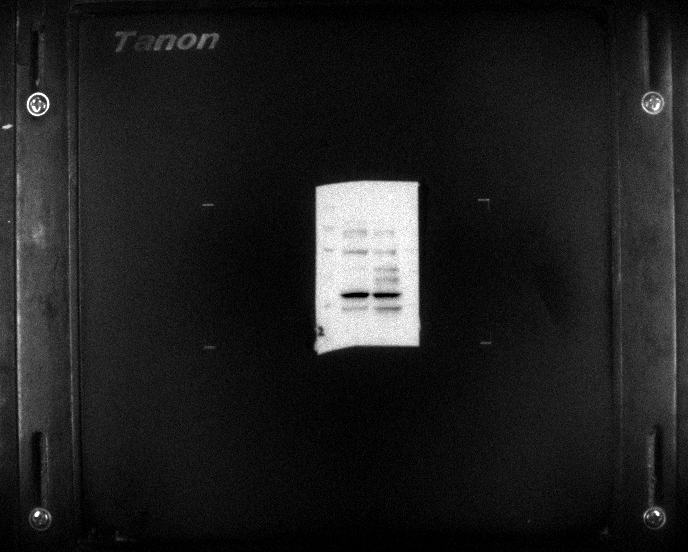

Supplement: Figure 4—source data 5. [file elife-88375-fig4-data5.zip › Figure 4-source data 5/K562 Input a┬-Tubulin.tif]

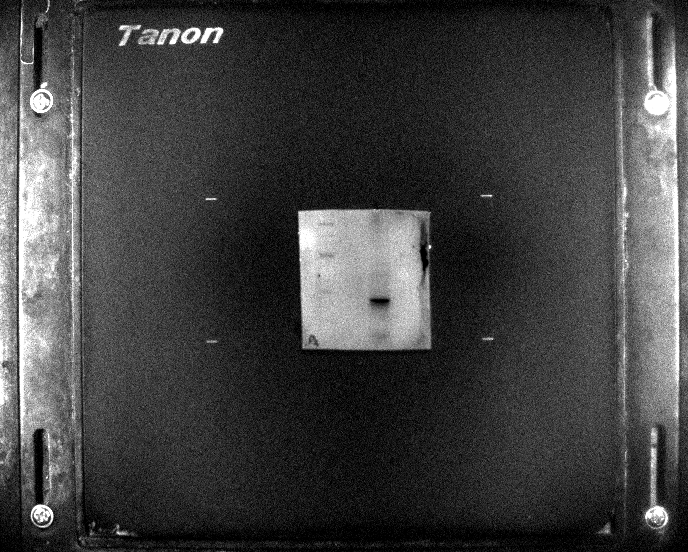

Supplement: Figure 4—source data 5. [file elife-88375-fig4-data5.zip › Figure 4-source data 5/K562 IP P-Tyr-IB RAPSYN.tif]

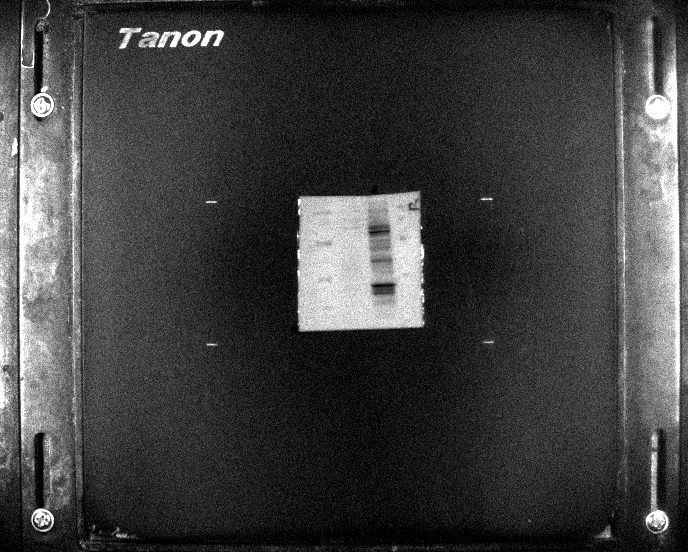

Supplement: Figure 4—source data 5. [file elife-88375-fig4-data5.zip › Figure 4-source data 5/K562 IP RAPSYN-IB P-Tyr.tif]

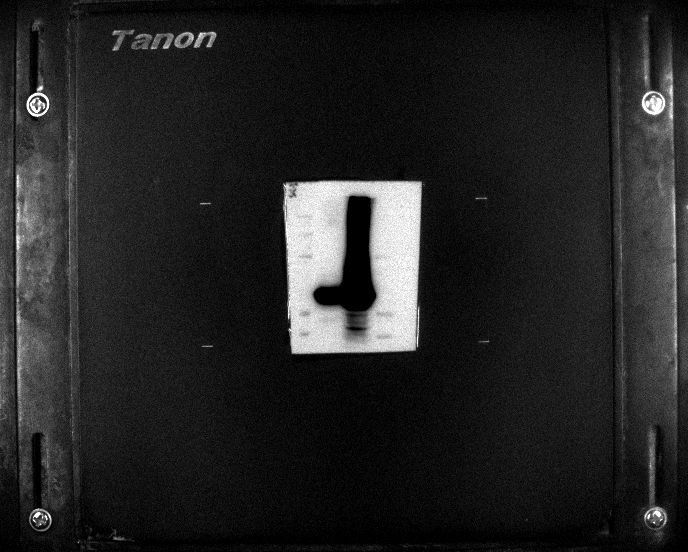

Supplement: Figure 4—source data 5. [file elife-88375-fig4-data5.zip › Figure 4-source data 5/MEG-01 Input RAPSYN.tif]

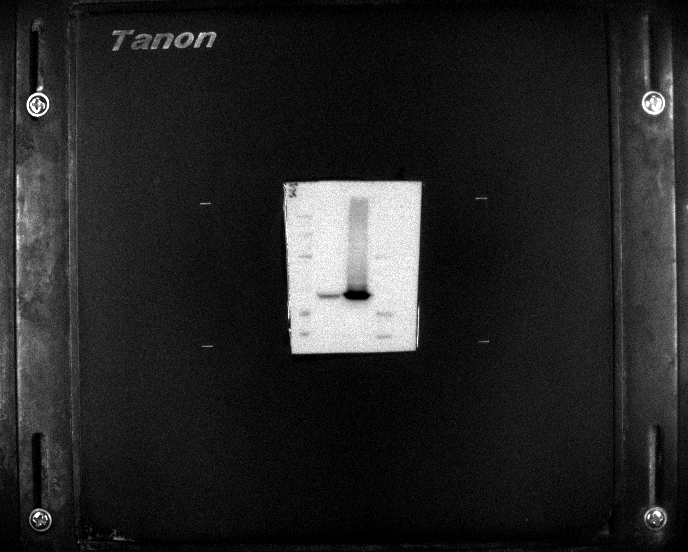

Supplement: Figure 4—source data 5. [file elife-88375-fig4-data5.zip › Figure 4-source data 5/MEG-01 Input SRC.tif]

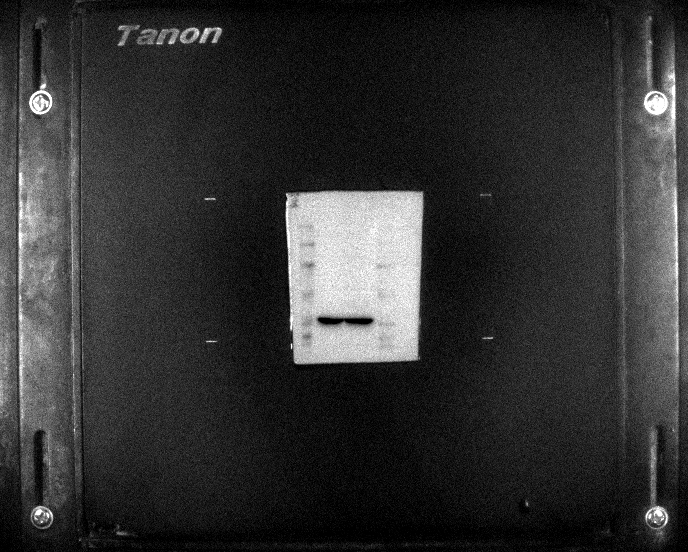

Supplement: Figure 4—source data 5. [file elife-88375-fig4-data5.zip › Figure 4-source data 5/MEG-01 Input a┬-Tubulin.tif]

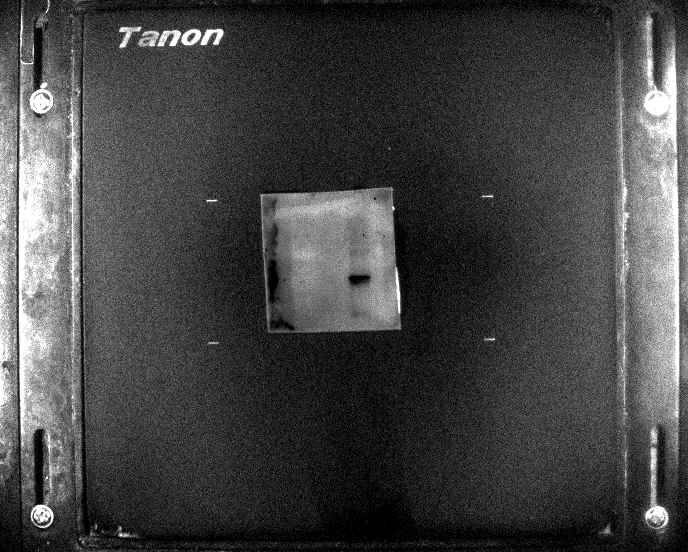

Supplement: Figure 4—source data 5. [file elife-88375-fig4-data5.zip › Figure 4-source data 5/MEG-01 IP P-Tyr-IB RAPSYN.tif]

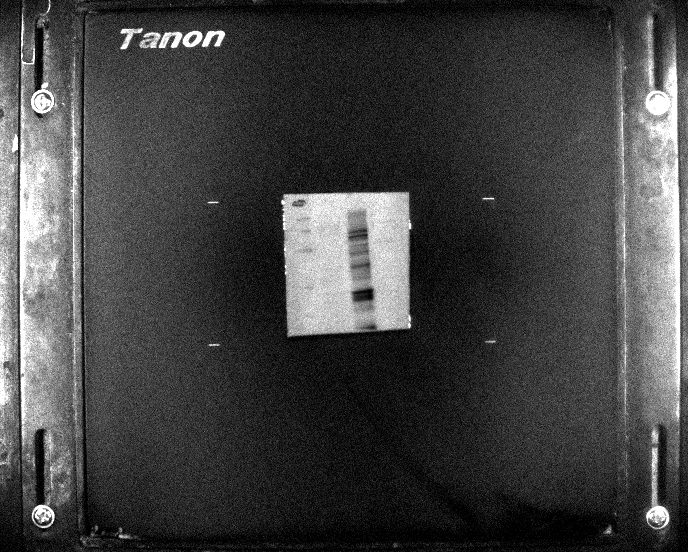

Supplement: Figure 4—source data 5. [file elife-88375-fig4-data5.zip › Figure 4-source data 5/MEG-01 IP RAPSYN-IB P-Tyr.tif]

C

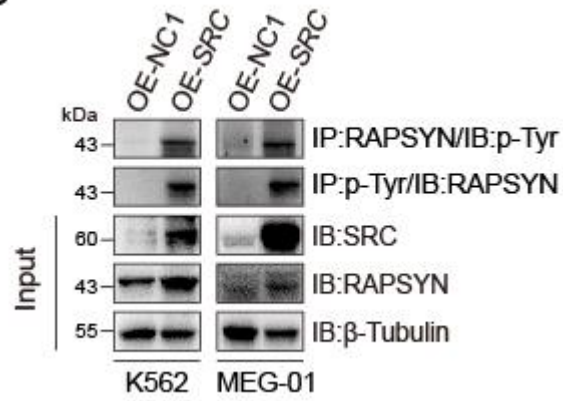

K562

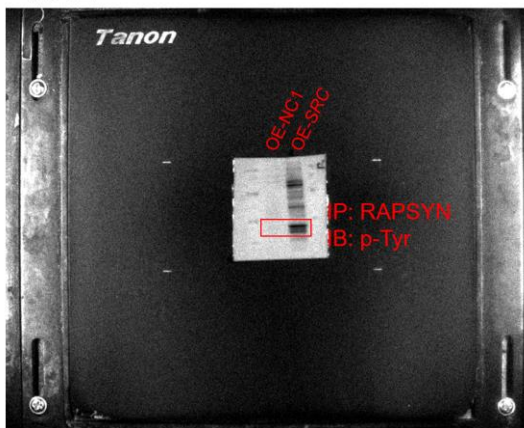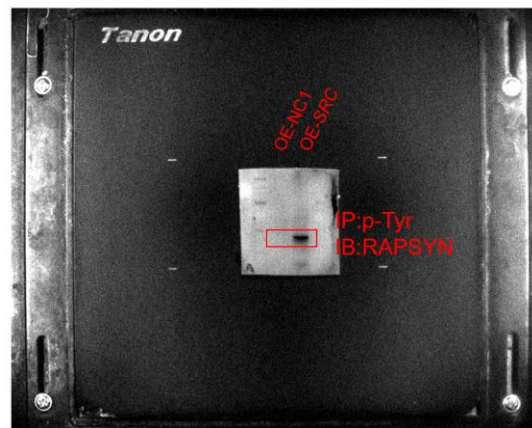

Input

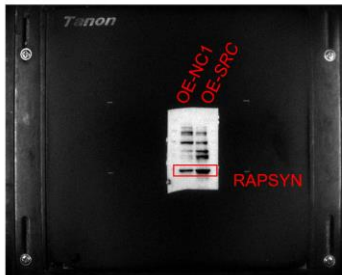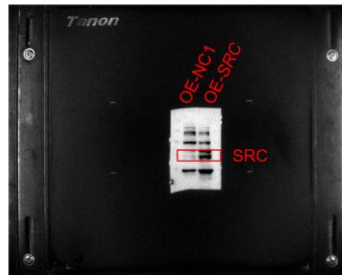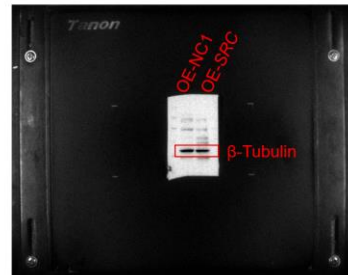

MEG-01

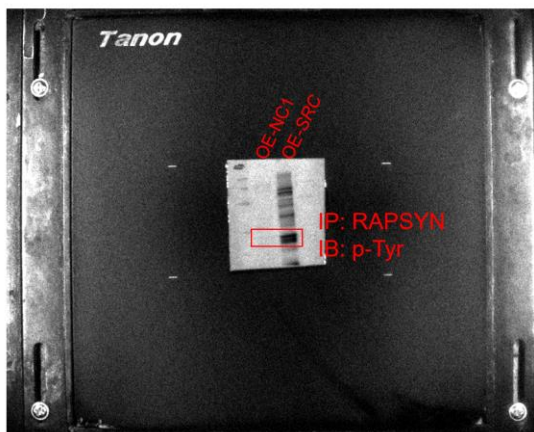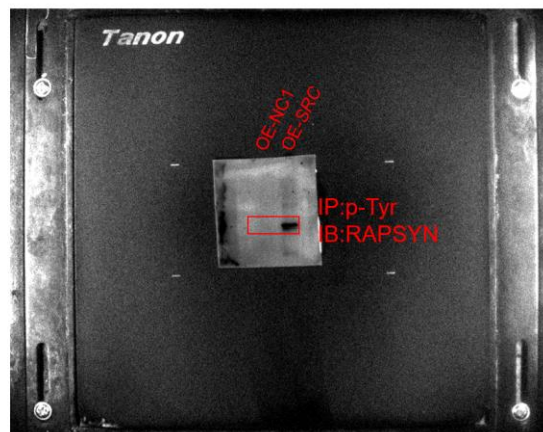

Input

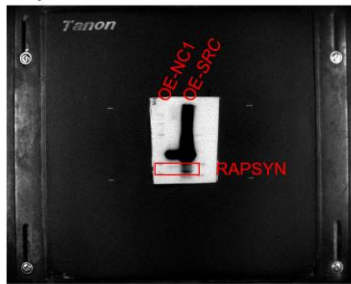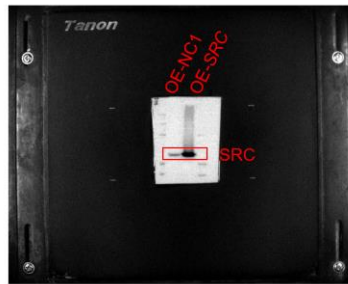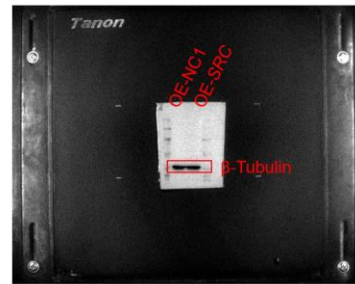

Supplement: Figure 4—source data 6. [file elife-88375-fig4-data6.zip › Figure 4-source data 6/Figure 4-source data 6.pdf]

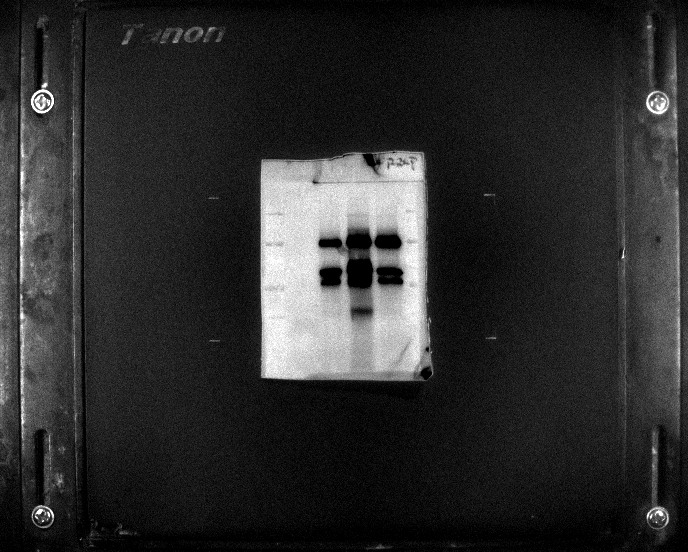

Supplement: Figure 4—source data 7. [file elife-88375-fig4-data7.zip › Figure 4-source data 7/p-Tyr.tif]

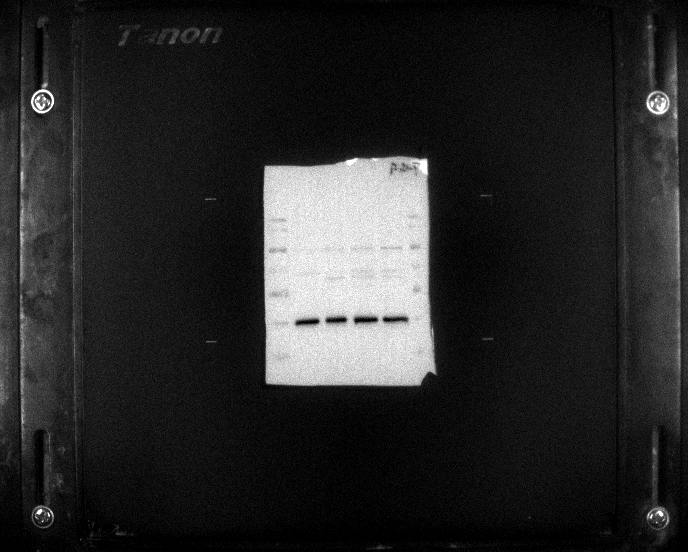

Supplement: Figure 4—source data 7. [file elife-88375-fig4-data7.zip › Figure 4-source data 7/RAPSYN.tif]

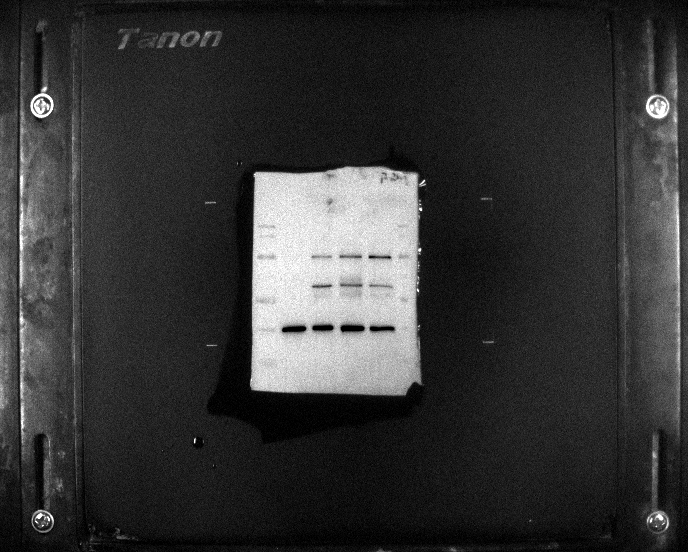

Supplement: Figure 4—source data 7. [file elife-88375-fig4-data7.zip › Figure 4-source data 7/SRC.tif]

D

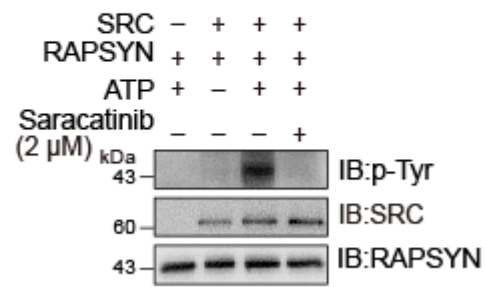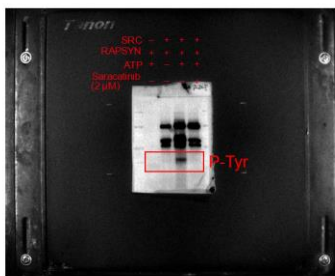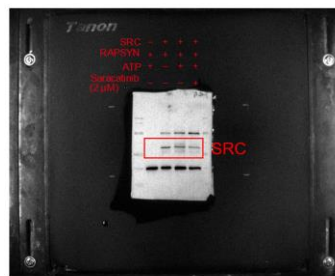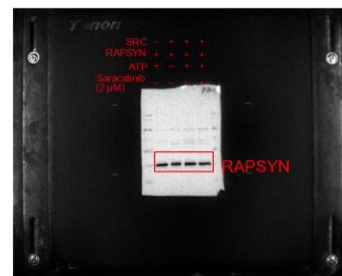

Supplement: Figure 4—source data 8. [file elife-88375-fig4-data8.zip › Figure 4-source data 8/Figure 4-source data 8.pdf]

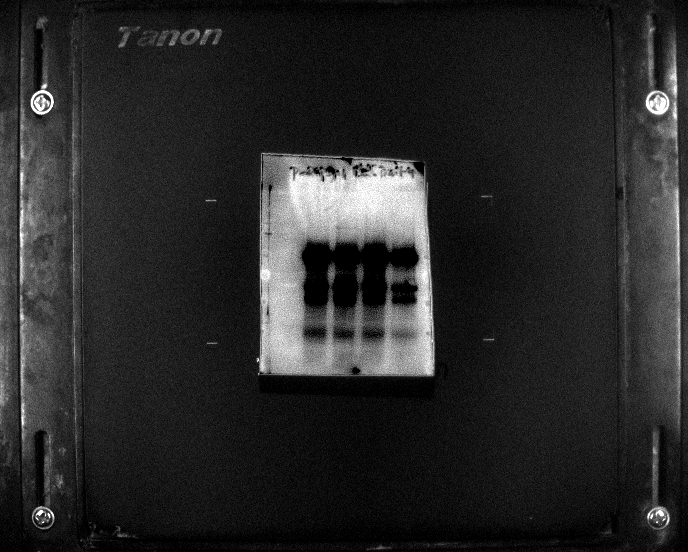

Supplement: Figure 4—source data 9. [file elife-88375-fig4-data9.zip › Figure 4-source data 9/p-Tyr.tif]

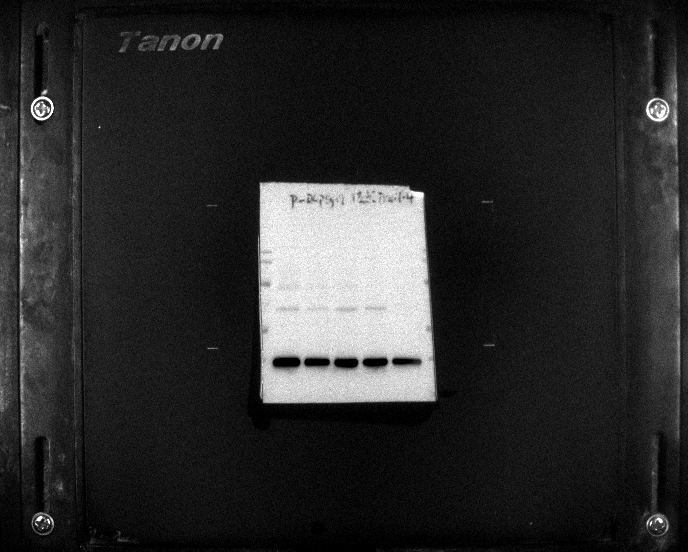

Supplement: Figure 4—source data 9. [file elife-88375-fig4-data9.zip › Figure 4-source data 9/RAPSYN.tif]

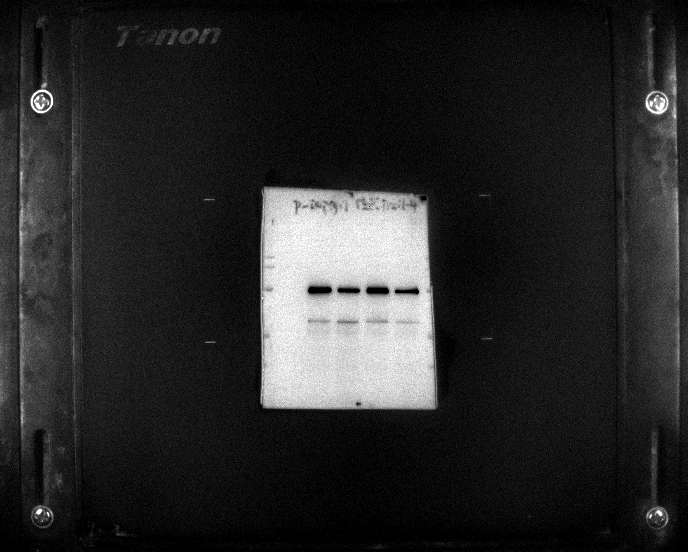

Supplement: Figure 4—source data 9. [file elife-88375-fig4-data9.zip › Figure 4-source data 9/SRC.tif]

E

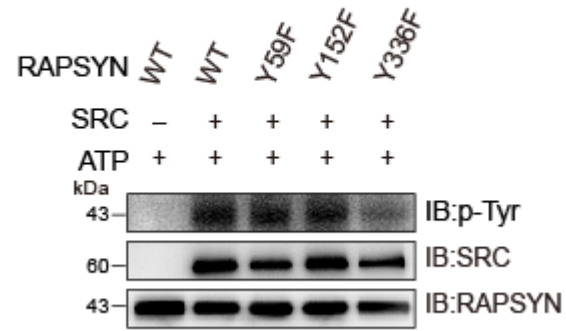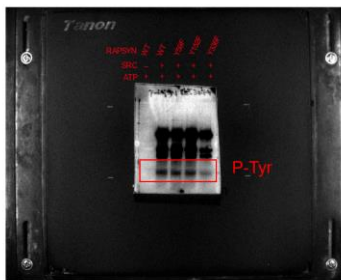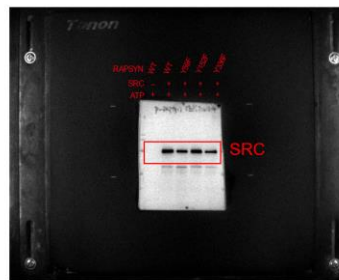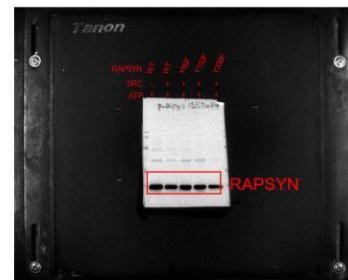

Supplement: Figure 4—source data 10. [file elife-88375-fig4-data10.zip › Figure 4-source data 10/Figure 4-source data 10.pdf]

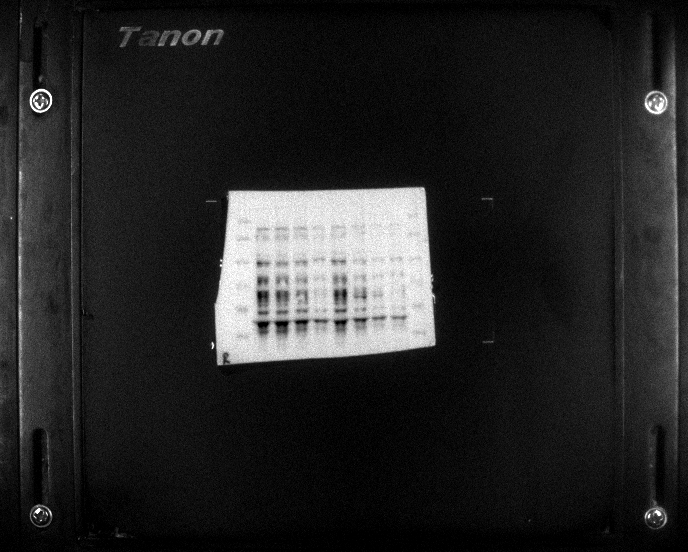

Supplement: Figure 4—source data 11. [file elife-88375-fig4-data11.zip › Figure 4-source data 11/K562 p-SRC.tif]

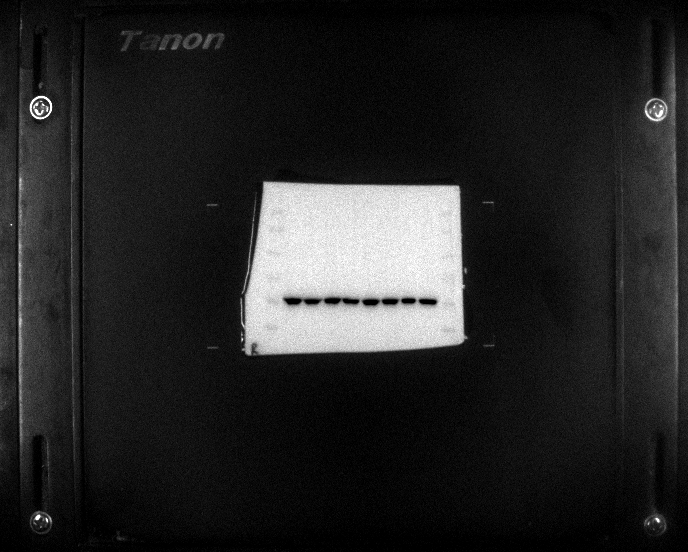

Supplement: Figure 4—source data 11. [file elife-88375-fig4-data11.zip › Figure 4-source data 11/K562 a┬-Tubulin.tif]

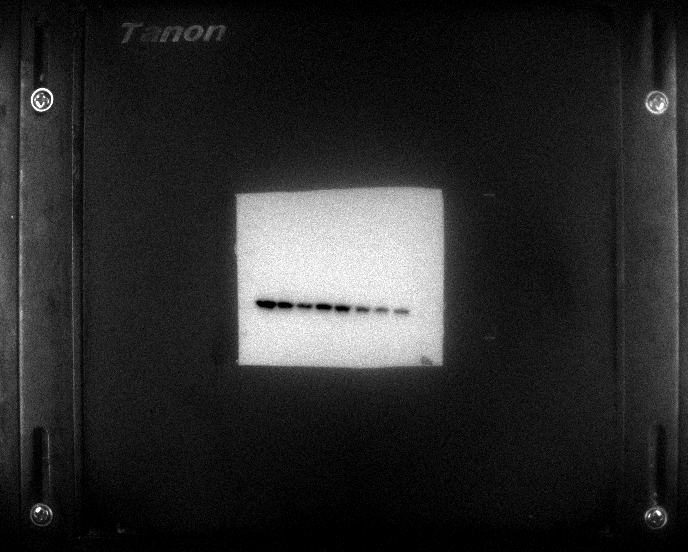

Supplement: Figure 4—source data 11. [file elife-88375-fig4-data11.zip › Figure 4-source data 11/MEG-01 P-SRC.tif]

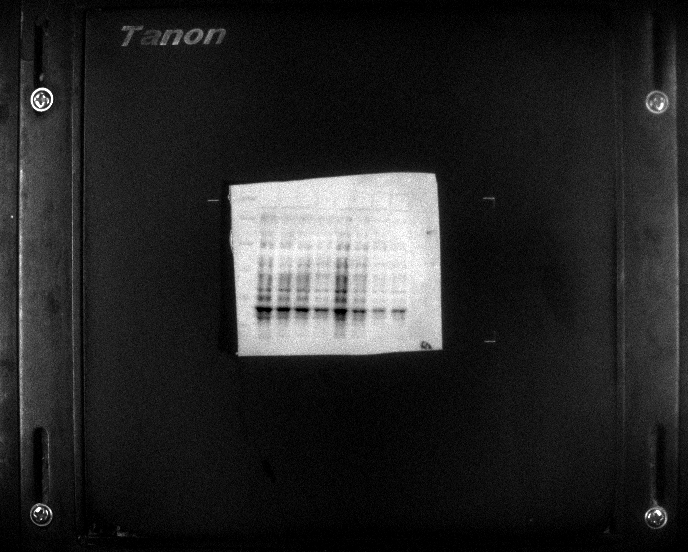

Supplement: Figure 4—source data 11. [file elife-88375-fig4-data11.zip › Figure 4-source data 11/MEG-01 RAPSYN.tif]

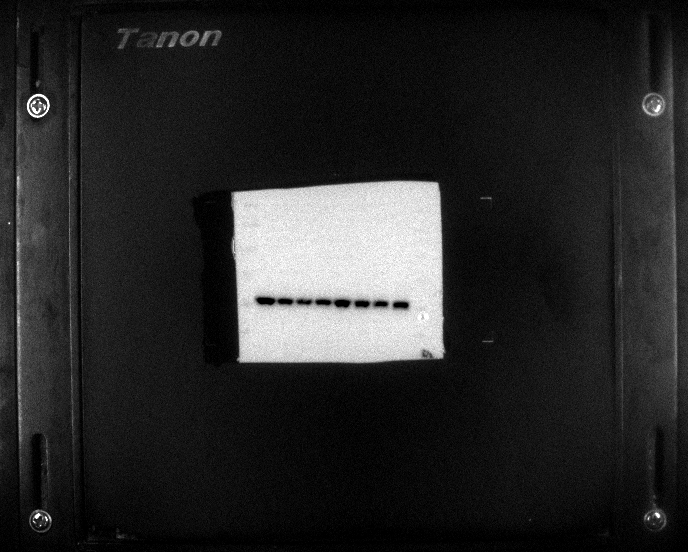

Supplement: Figure 4—source data 11. [file elife-88375-fig4-data11.zip › Figure 4-source data 11/MEG-01 a┬-Tubulin.tif]

K562

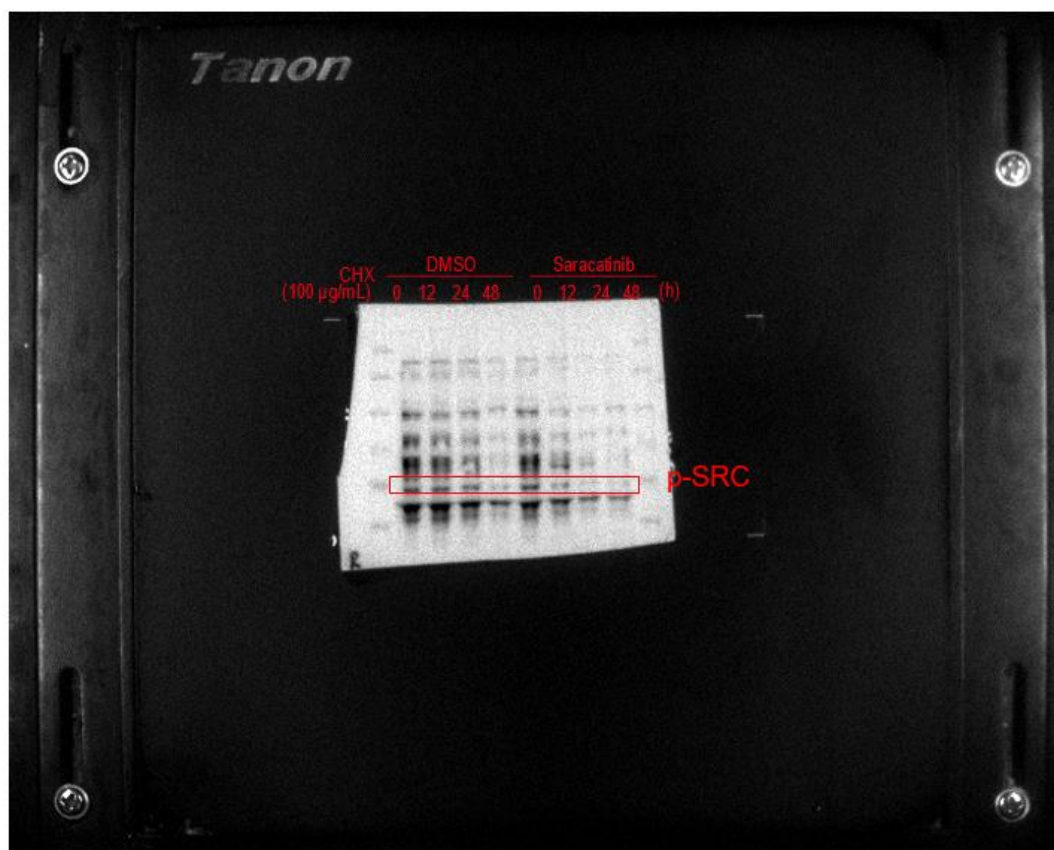

K562

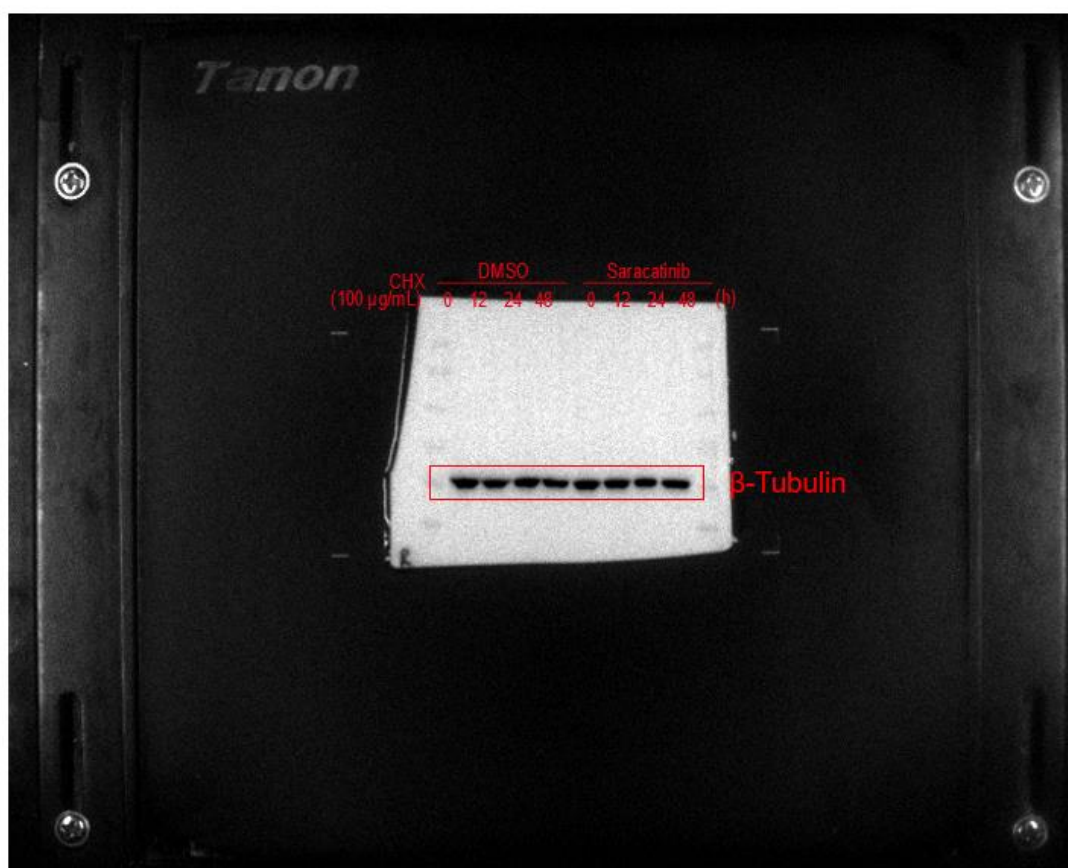

MEG-01

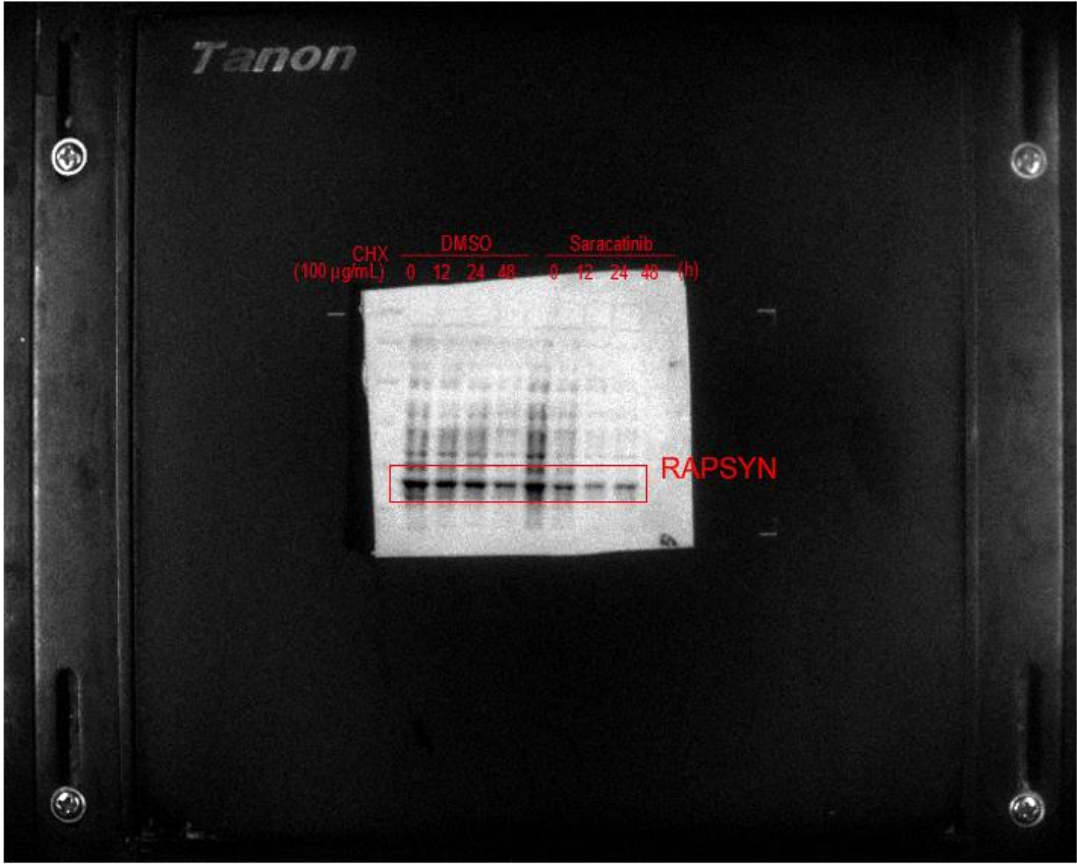

MEG-01

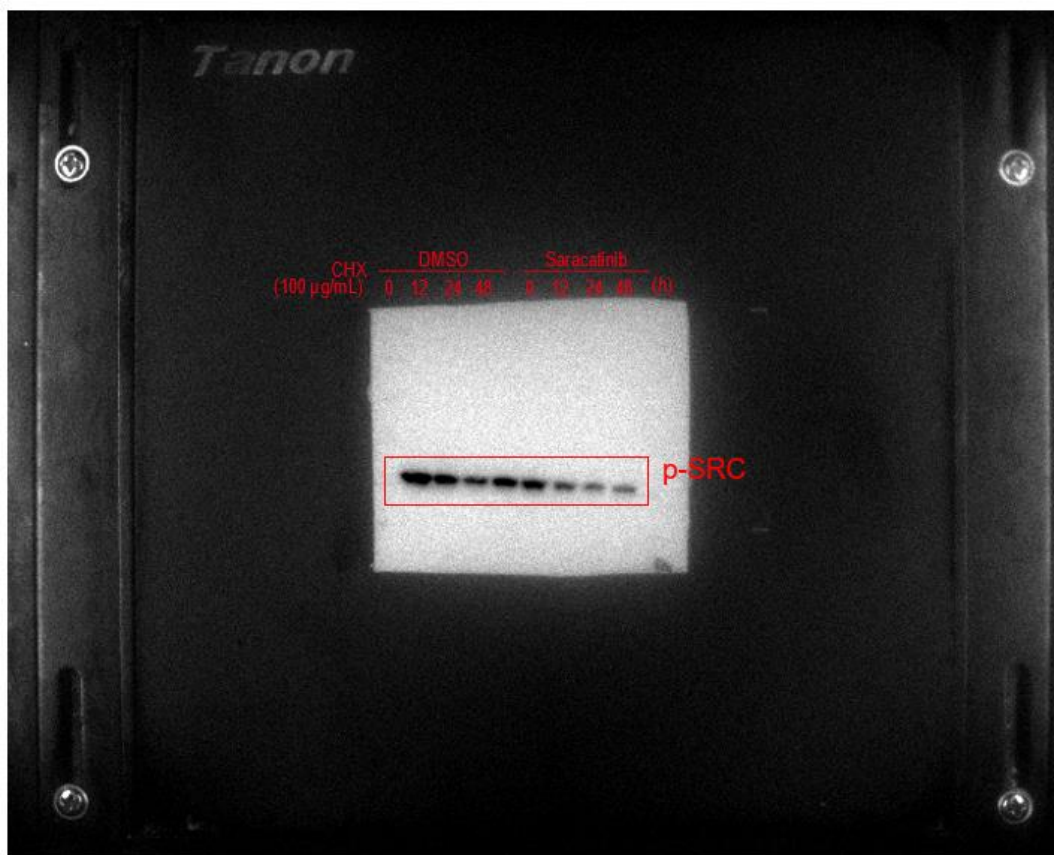

MEG-01

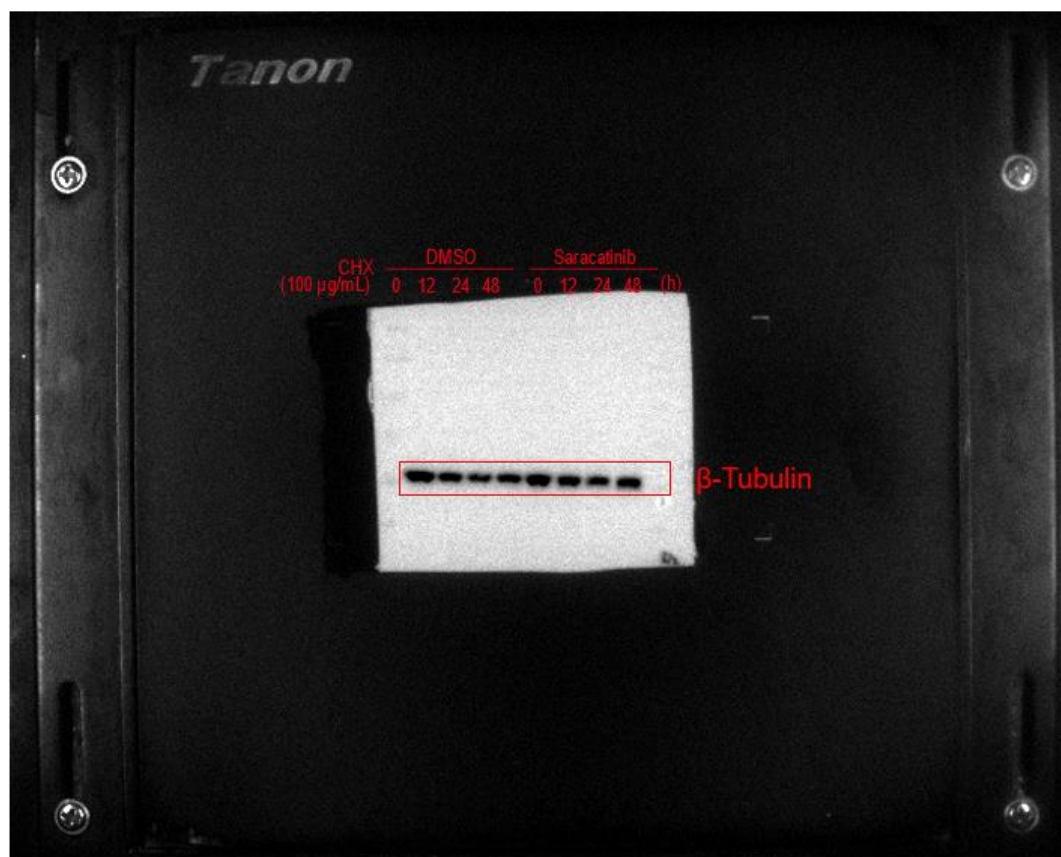

Supplement: Figure 4—source data 12. [file elife-88375-fig4-data12.zip › Figure 4-source data 12/Figure 4-source data 12.pdf]

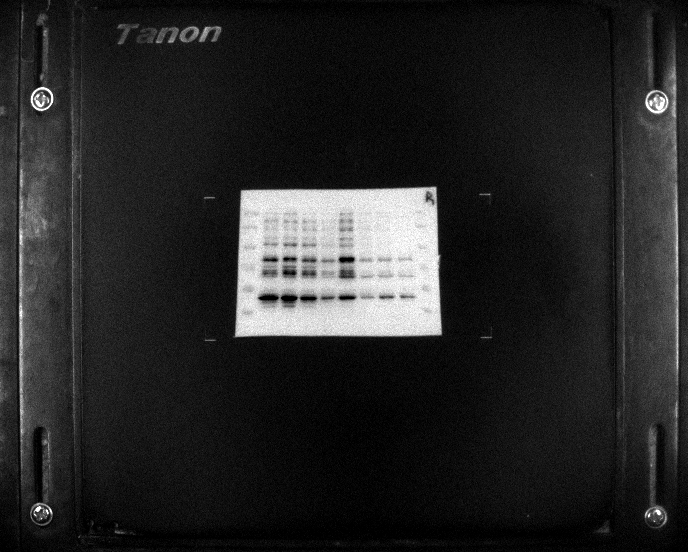

Supplement: Figure 4—source data 13. [file elife-88375-fig4-data13.zip › Figure 4-source data 13/K562 RAPSYN.tif]

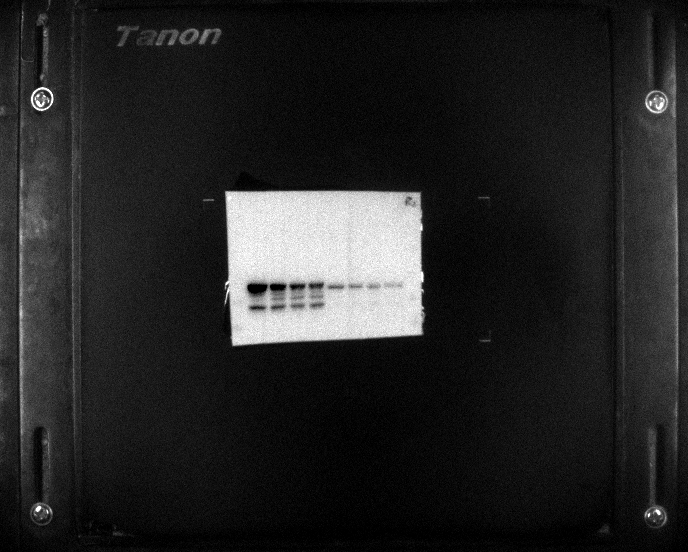

Supplement: Figure 4—source data 13. [file elife-88375-fig4-data13.zip › Figure 4-source data 13/K562 SRC.tif]

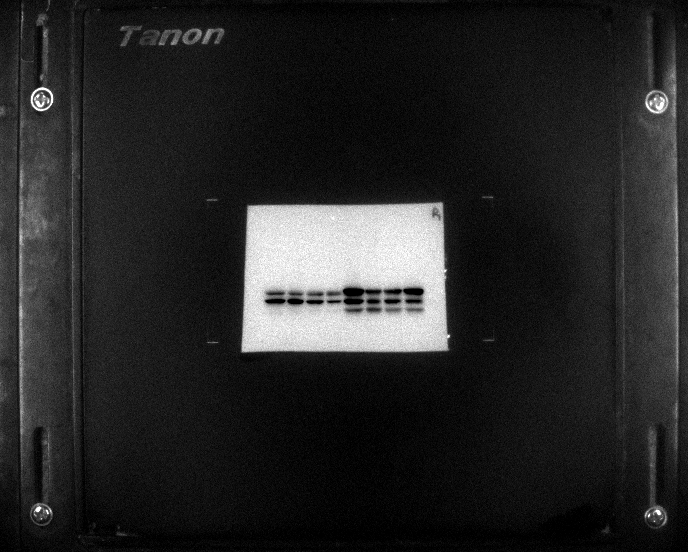

Supplement: Figure 4—source data 13. [file elife-88375-fig4-data13.zip › Figure 4-source data 13/K562 a┬-Tubulin.tif]

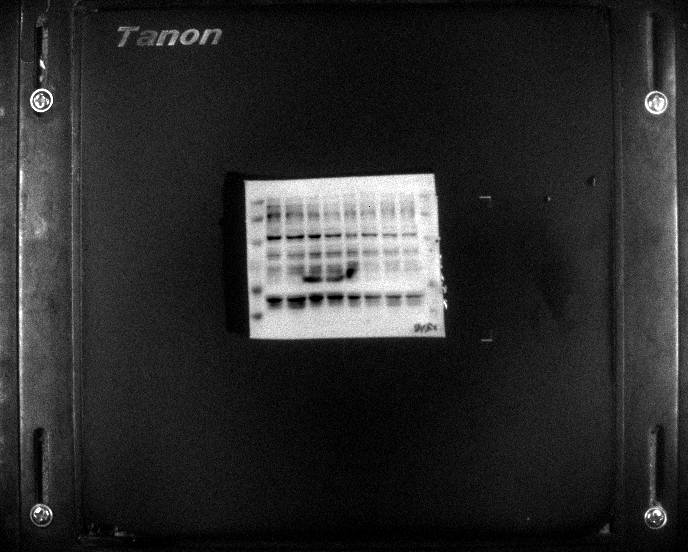

Supplement: Figure 4—source data 13. [file elife-88375-fig4-data13.zip › Figure 4-source data 13/MEG-01 RAPSYN.tif]

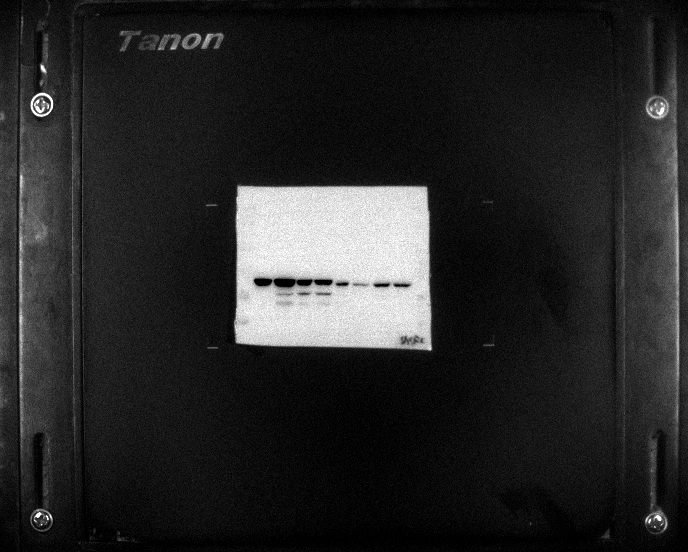

Supplement: Figure 4—source data 13. [file elife-88375-fig4-data13.zip › Figure 4-source data 13/MEG-01 SRC.tif]

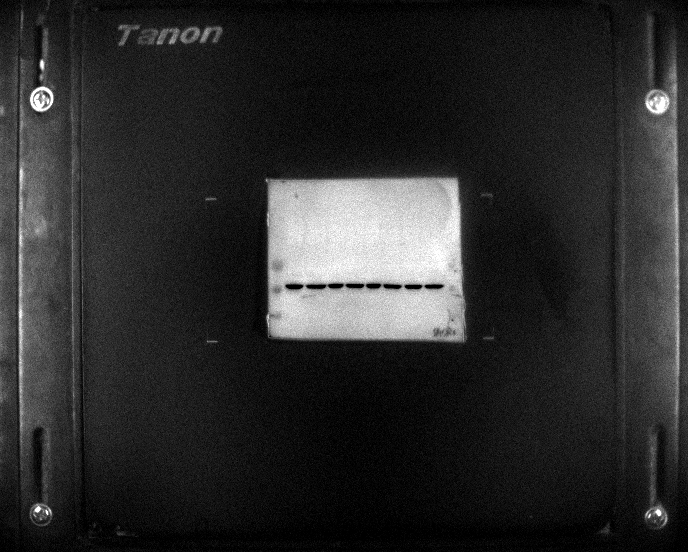

Supplement: Figure 4—source data 13. [file elife-88375-fig4-data13.zip › Figure 4-source data 13/MEG-01 a┬-Tubulin.tif]

**G**

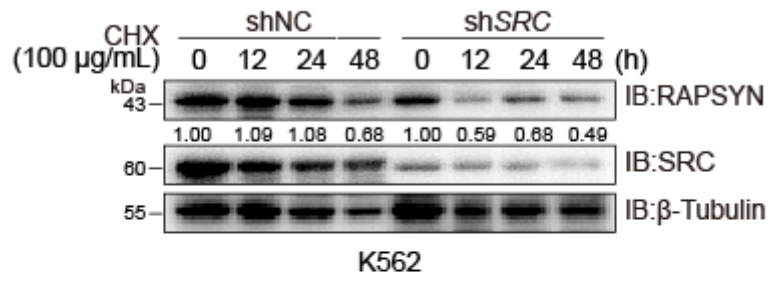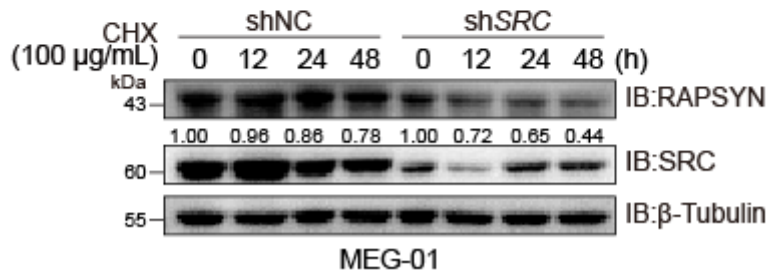

K562

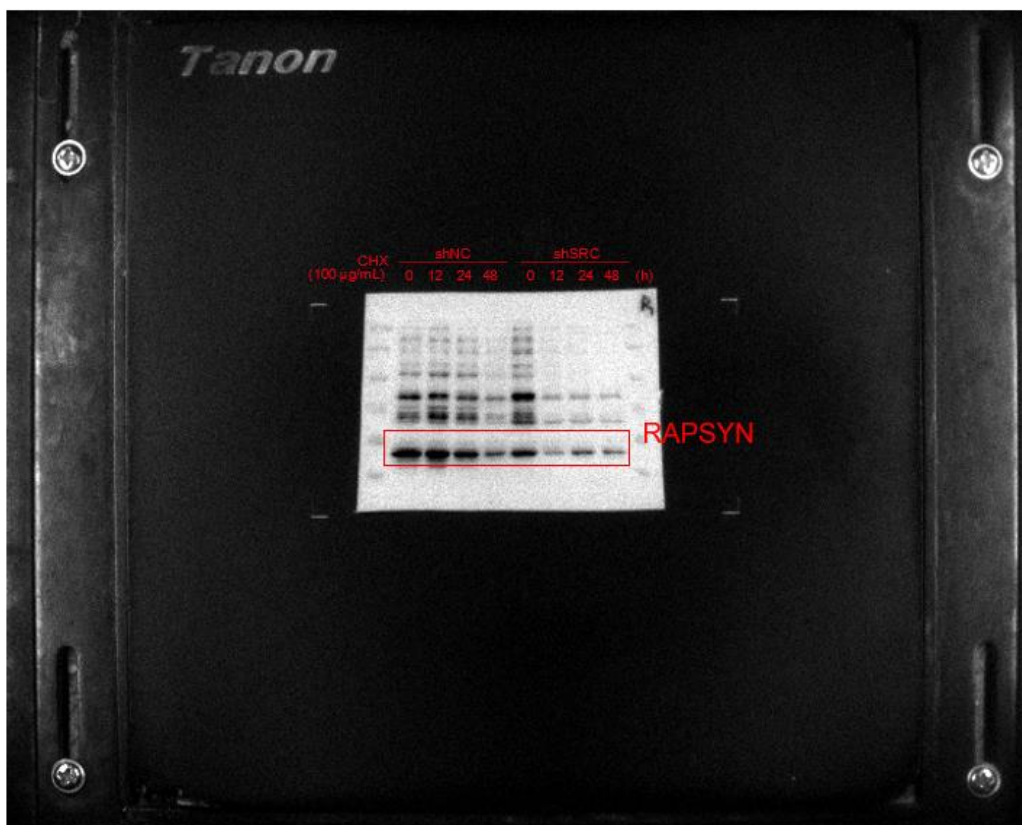

K562

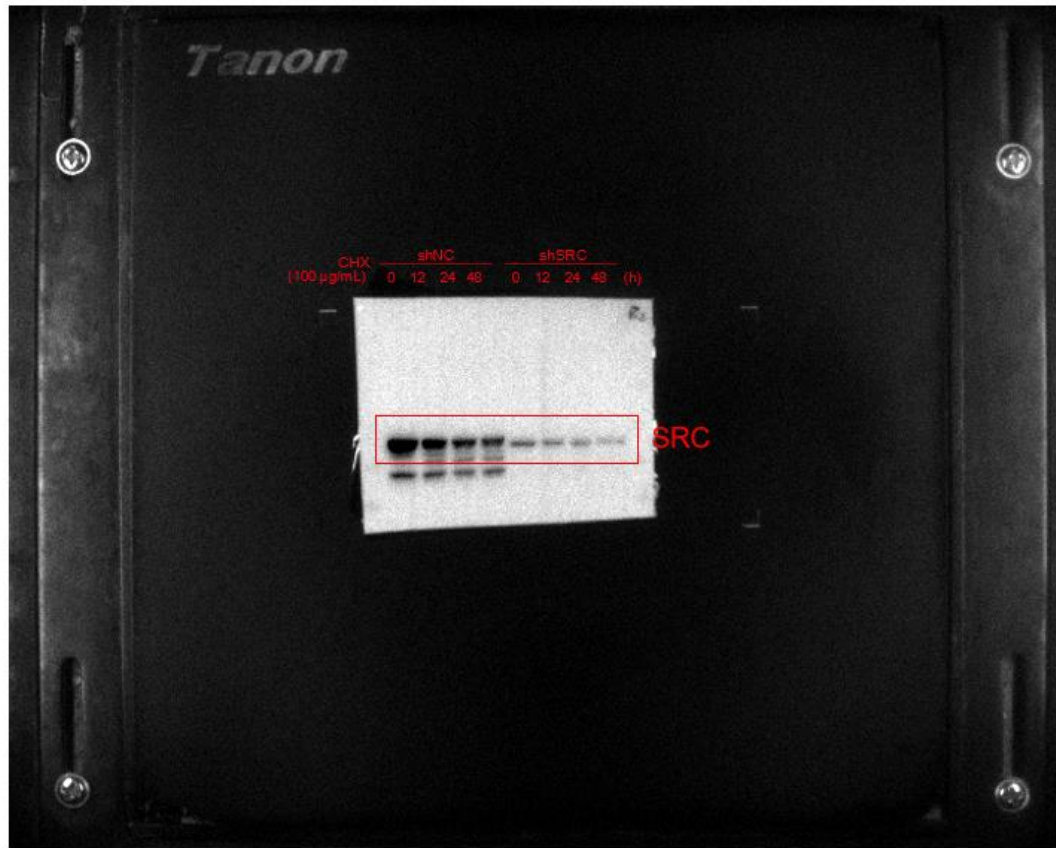

K562

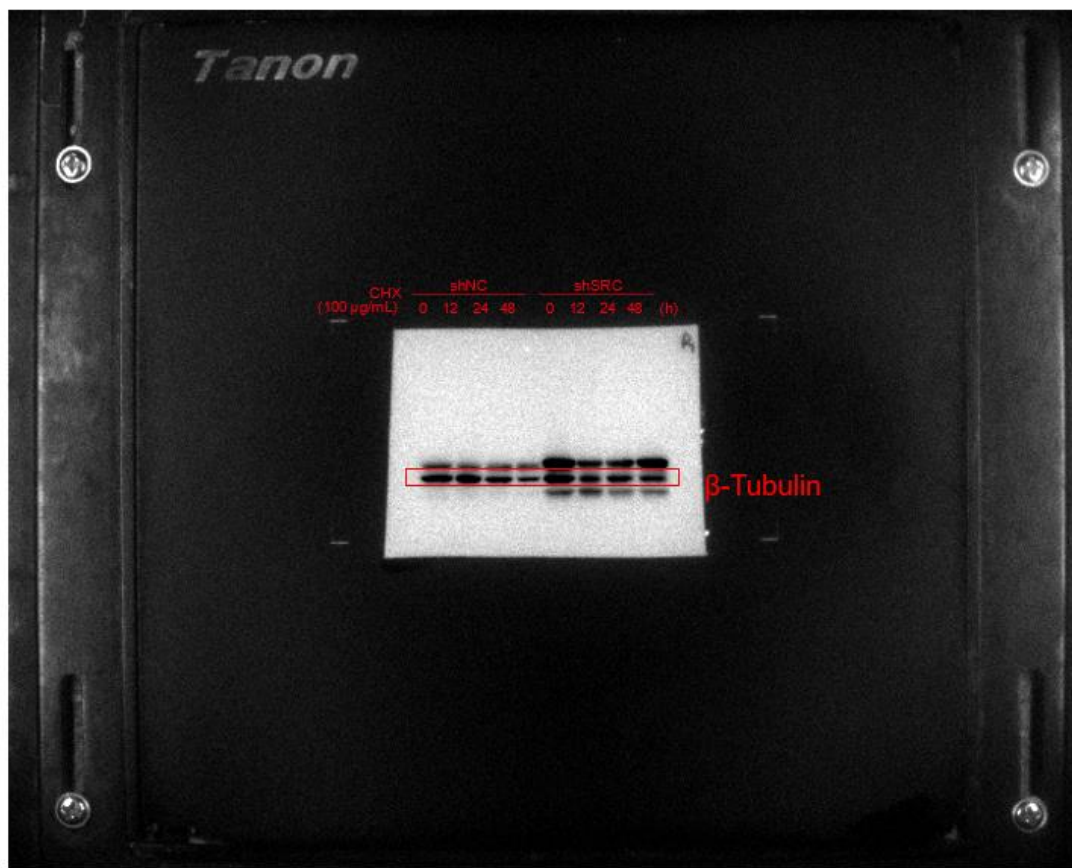

MEG-01

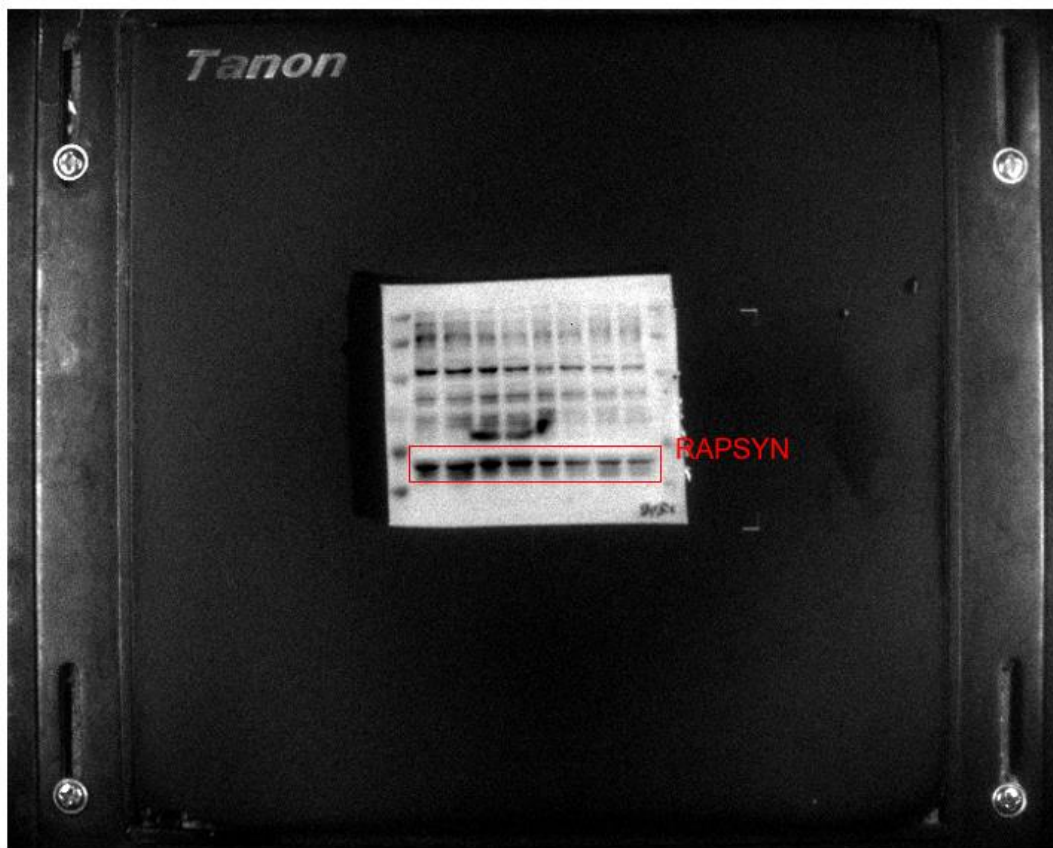

MEG-01

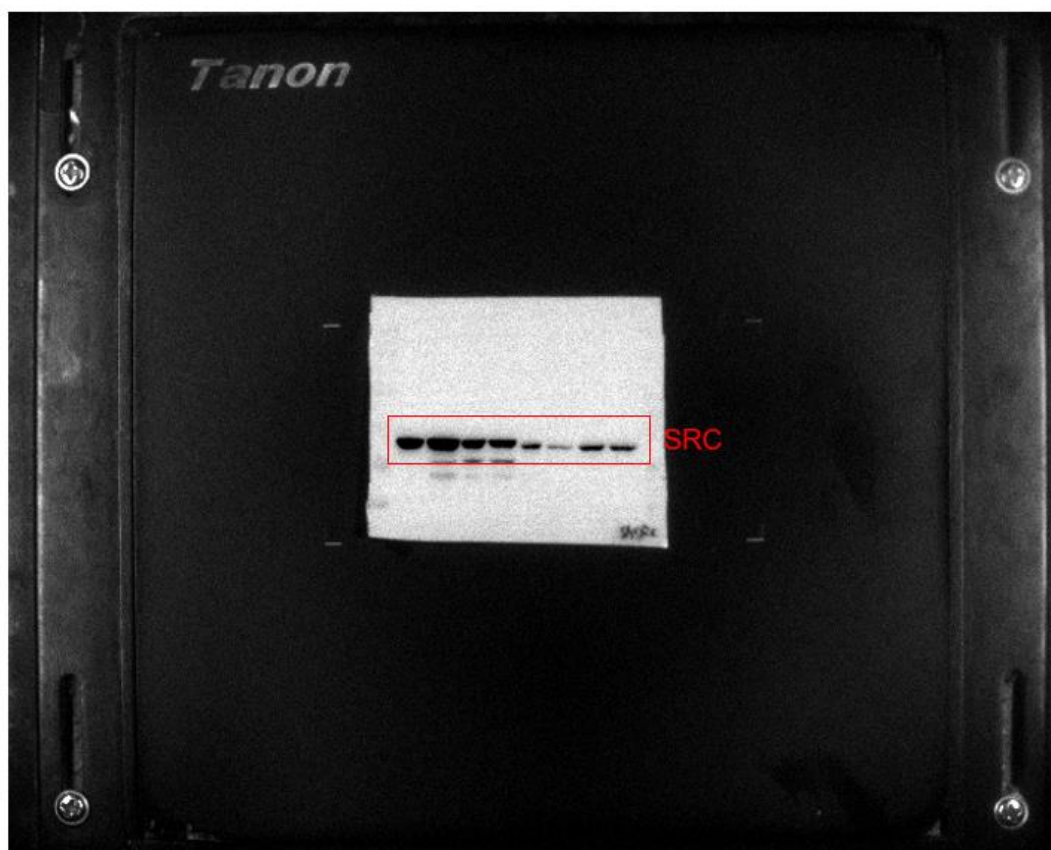

MEG-01

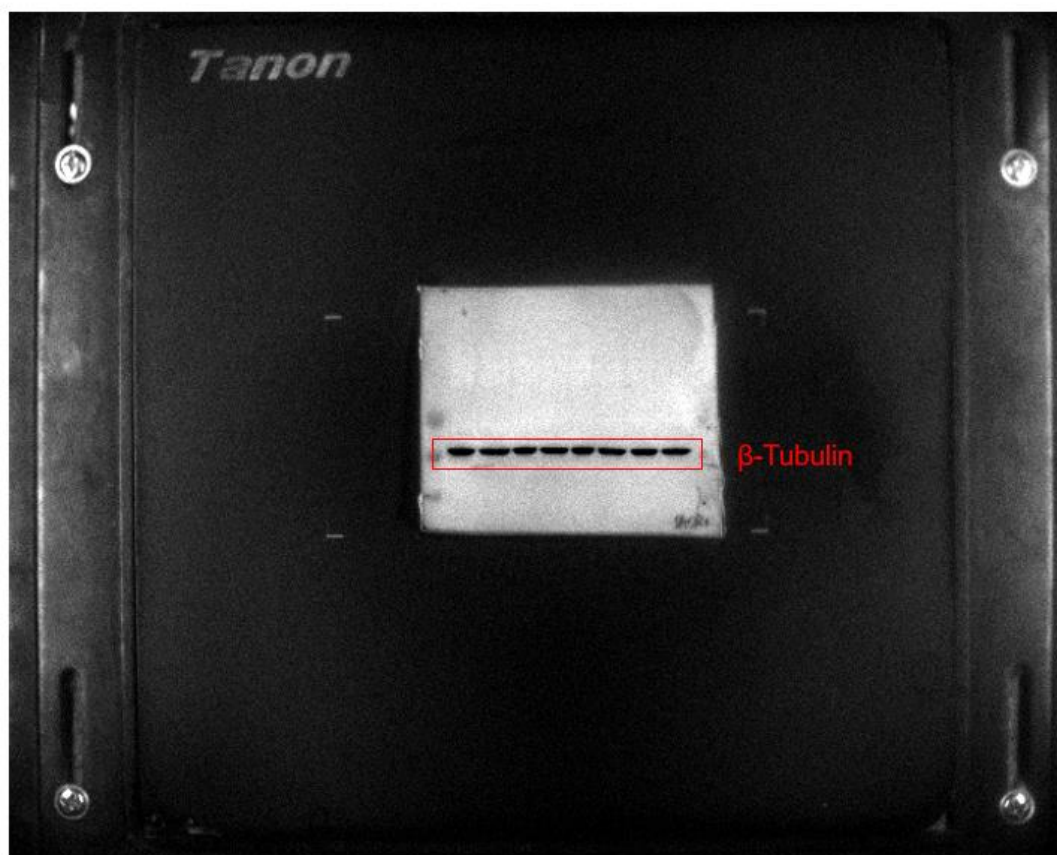

Supplement: Figure 4—source data 14. [file elife-88375-fig4-data14.zip › Figure 4-source data 14/Figure 4-source data 14.pdf]

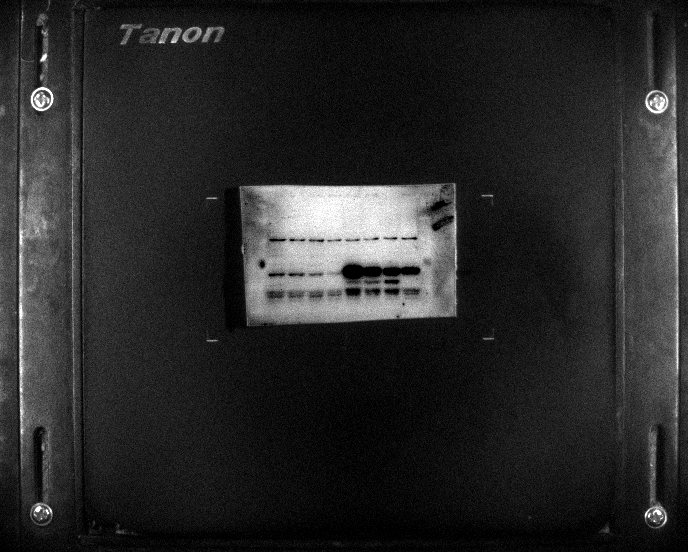

Supplement: Figure 4—source data 15. [file elife-88375-fig4-data15.zip › Figure 4-source data 15/K562 RAPSYN.tif]

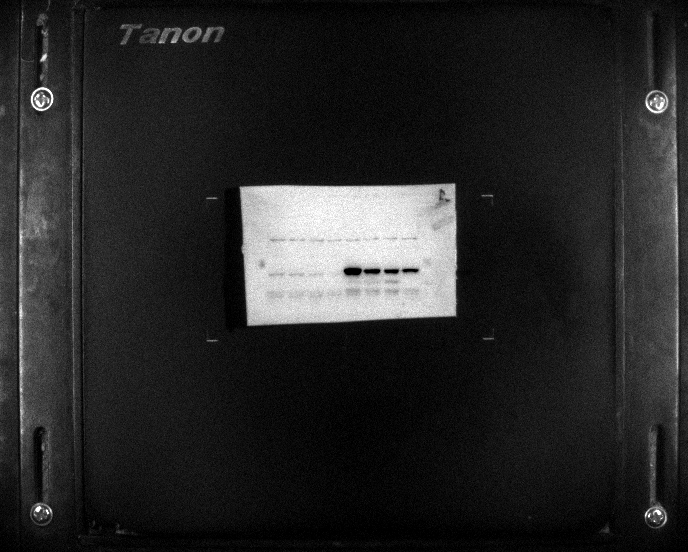

Supplement: Figure 4—source data 15. [file elife-88375-fig4-data15.zip › Figure 4-source data 15/K562 SRC.tif]

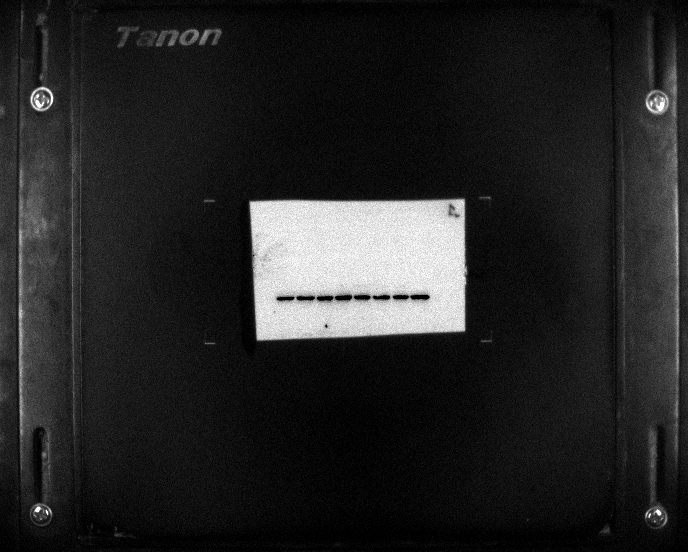

Supplement: Figure 4—source data 15. [file elife-88375-fig4-data15.zip › Figure 4-source data 15/K562 a┬-Tubulin.tif]

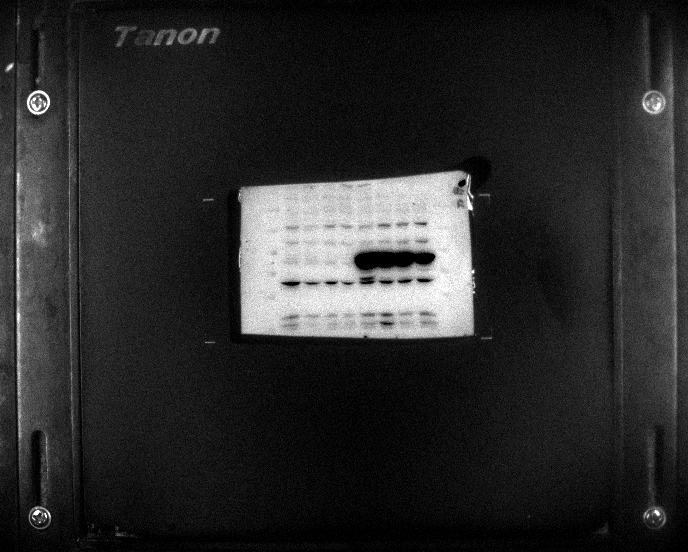

Supplement: Figure 4—source data 15. [file elife-88375-fig4-data15.zip › Figure 4-source data 15/MEG-01 RAPSYN.tif]

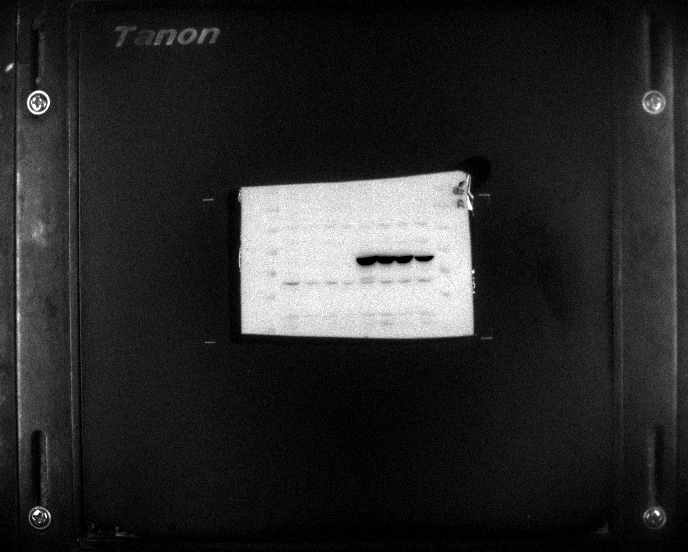

Supplement: Figure 4—source data 15. [file elife-88375-fig4-data15.zip › Figure 4-source data 15/MEG-01 SRC.tif]

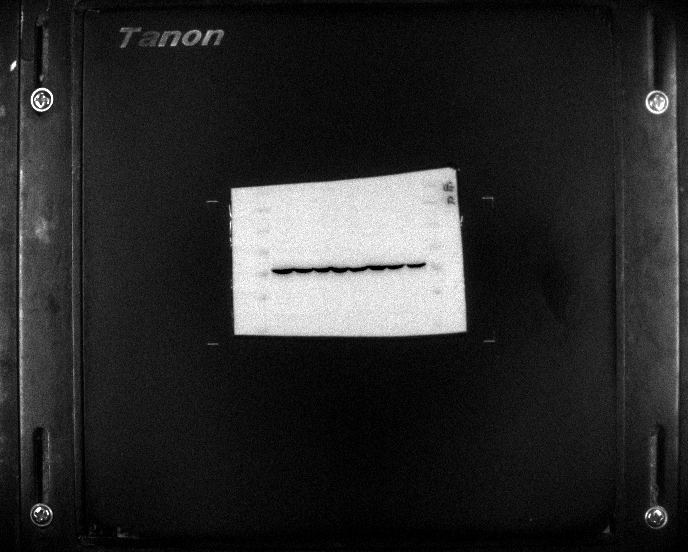

Supplement: Figure 4—source data 15. [file elife-88375-fig4-data15.zip › Figure 4-source data 15/MEG-01 a┬-Tubulin.tif]

H

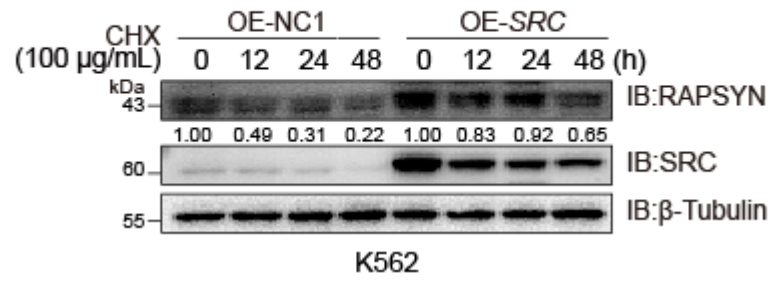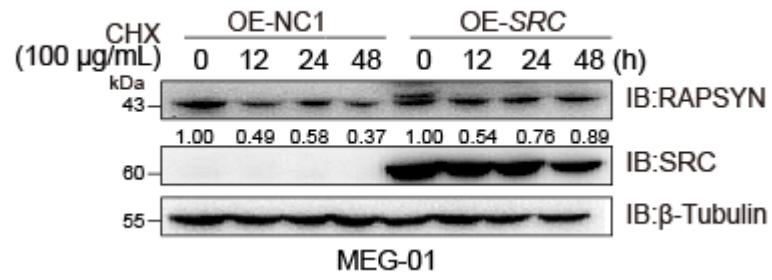

K562

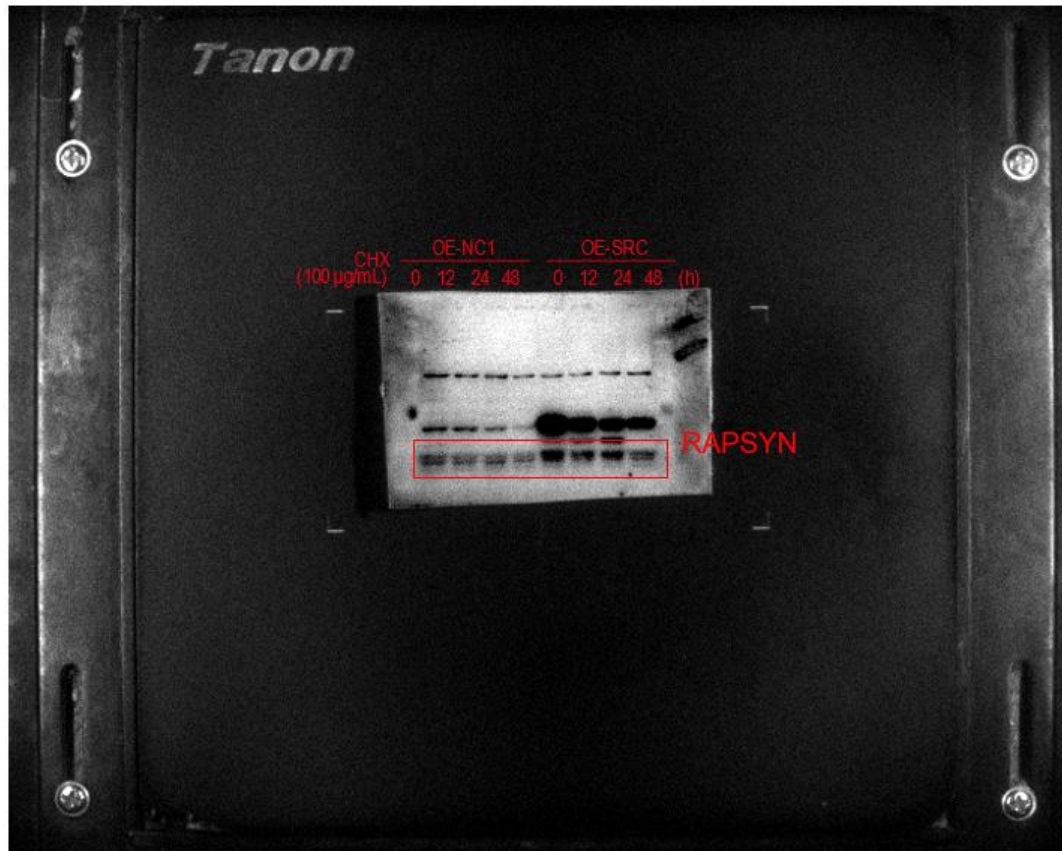

K562

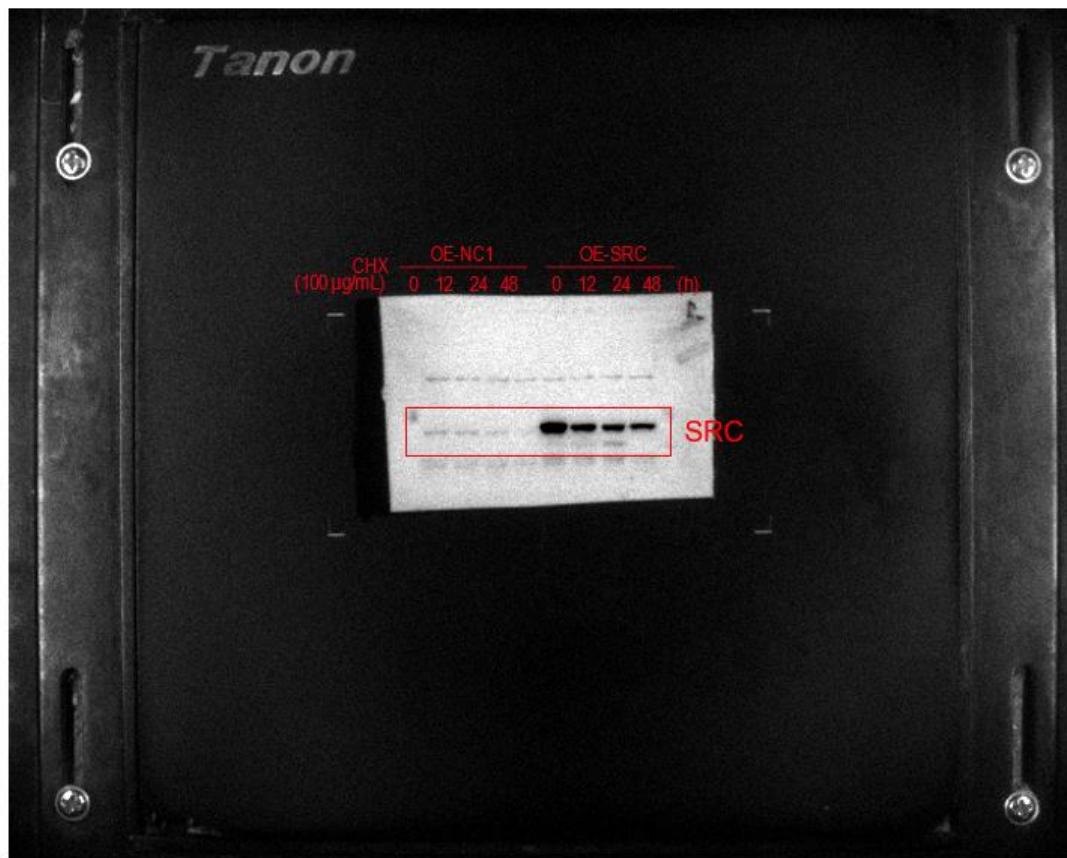

K562

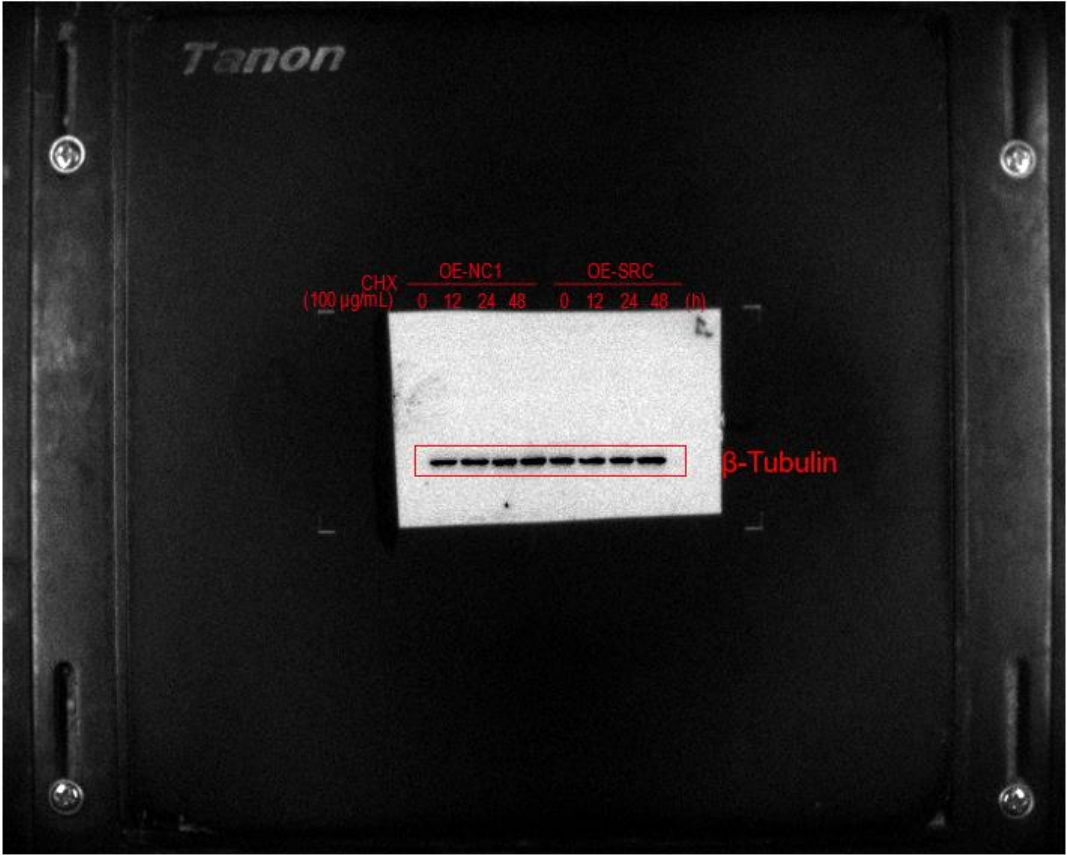

MEG-01

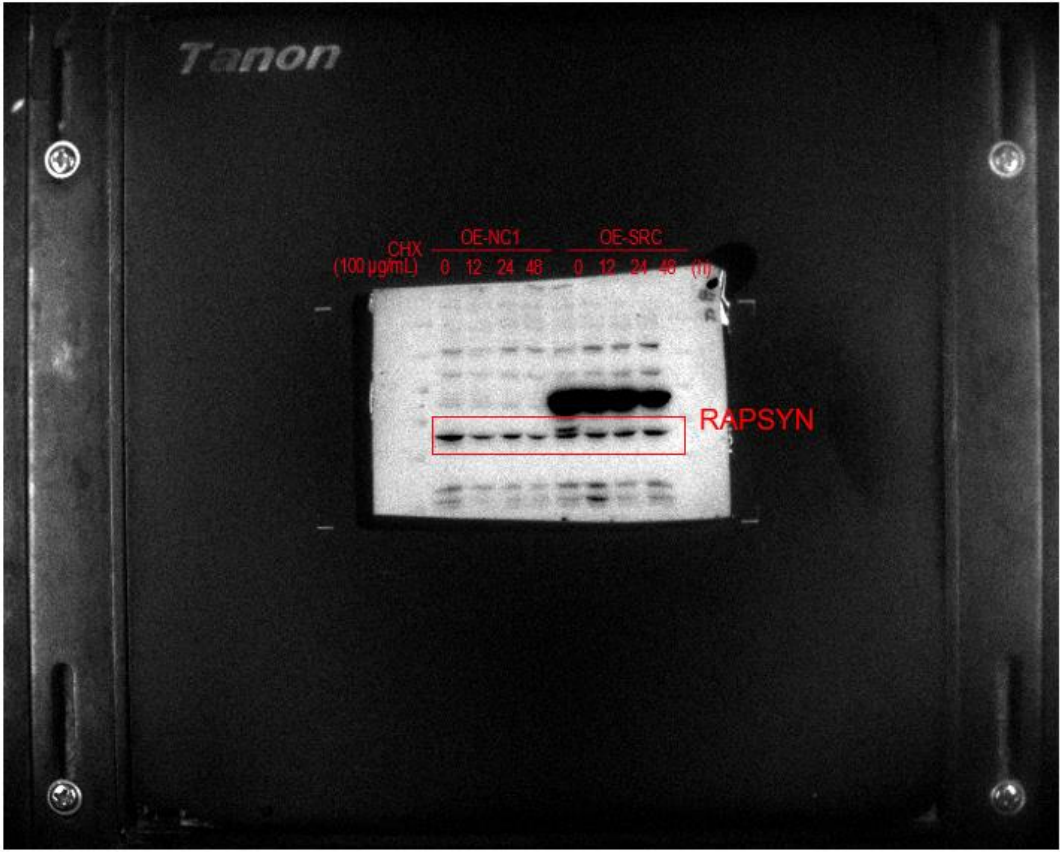

MEG-01

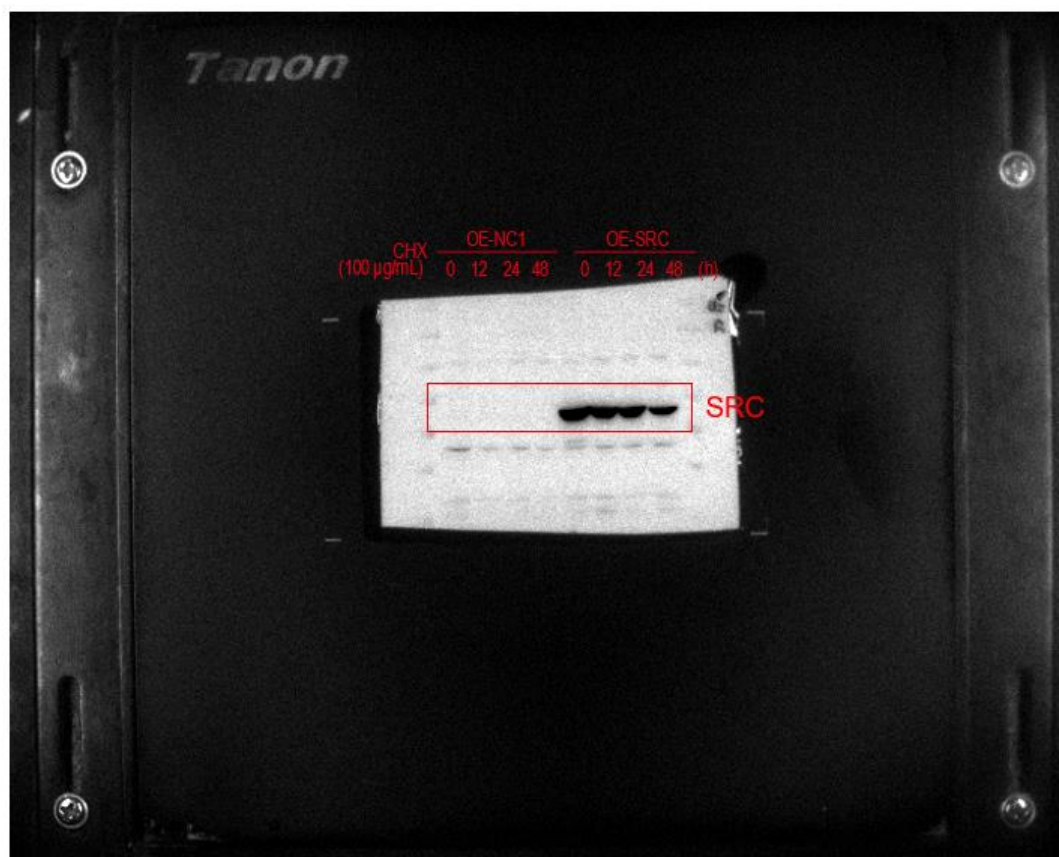

MEG-01

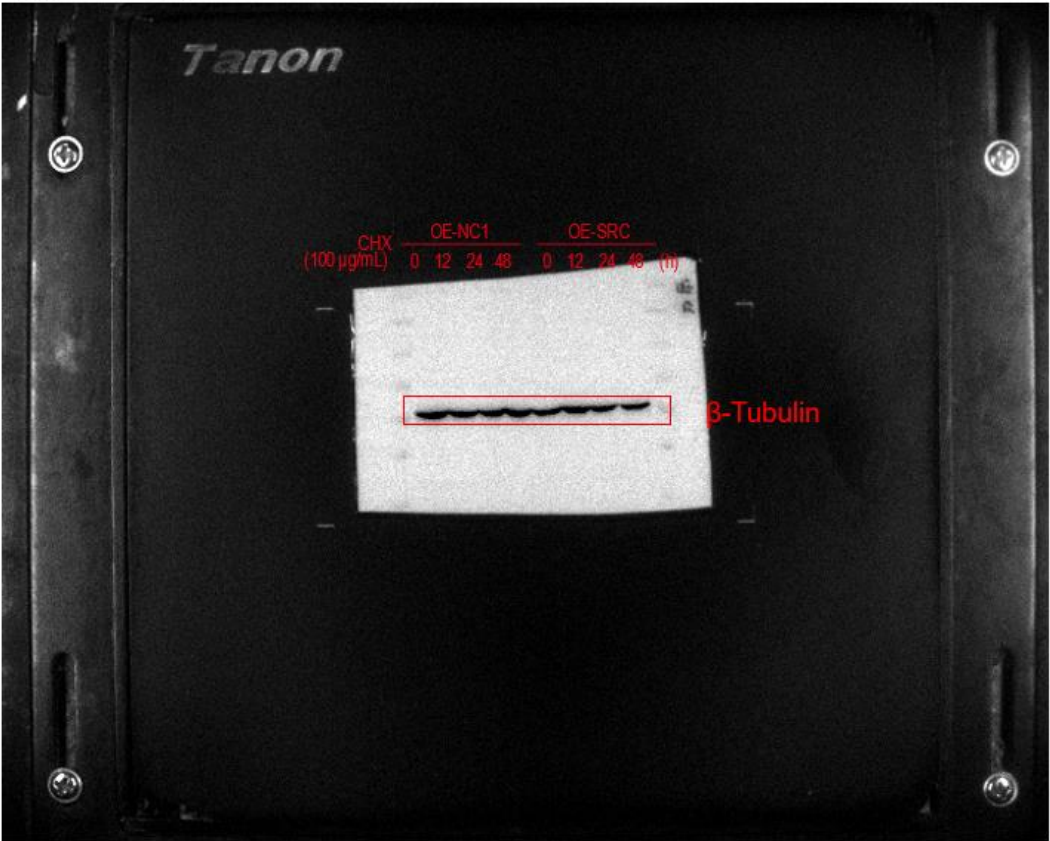

Supplement: Figure 4—source data 16. [file elife-88375-fig4-data16.zip › Figure 4-source data 16/Figure 4-source data 16.pdf]

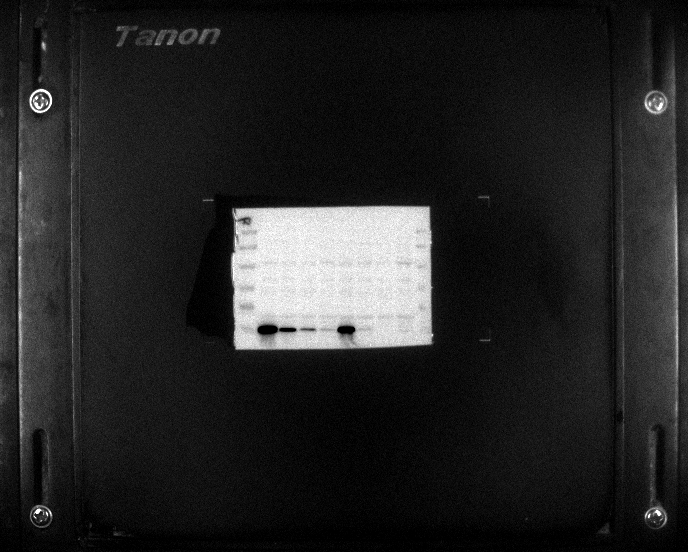

Supplement: Figure 4—source data 17. [file elife-88375-fig4-data17.zip › Figure 4-source data 17/K562 RAPSYN.tif]

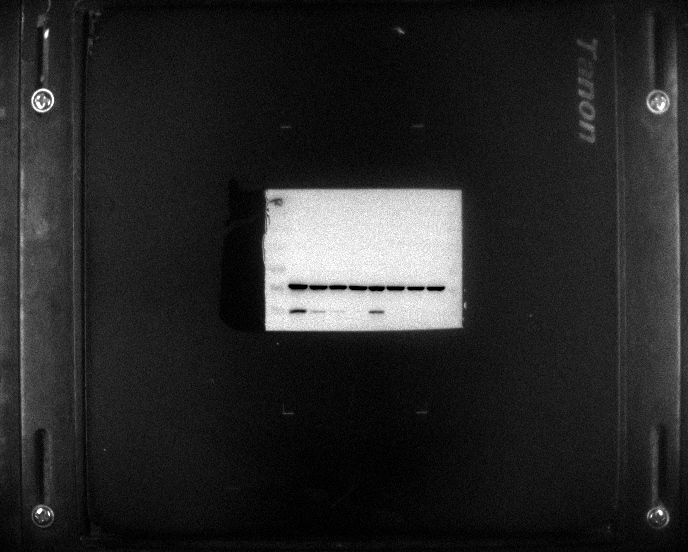

Supplement: Figure 4—source data 17. [file elife-88375-fig4-data17.zip › Figure 4-source data 17/K562 a┬-Tubulin.tif]

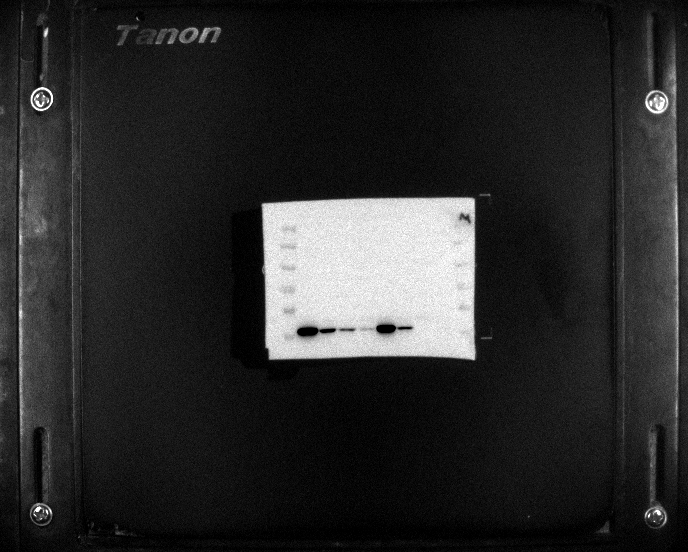

Supplement: Figure 4—source data 17. [file elife-88375-fig4-data17.zip › Figure 4-source data 17/MEG-01 RAPSYN.tif]

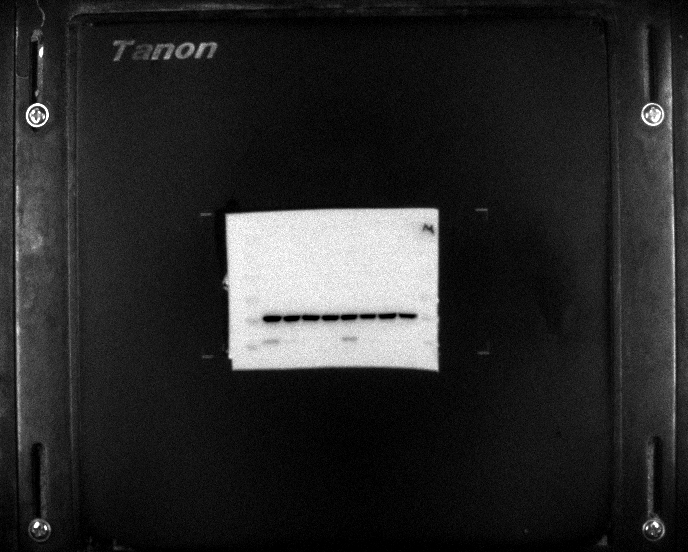

Supplement: Figure 4—source data 17. [file elife-88375-fig4-data17.zip › Figure 4-source data 17/MEG-01 a┬-Tubulin.tif]

I

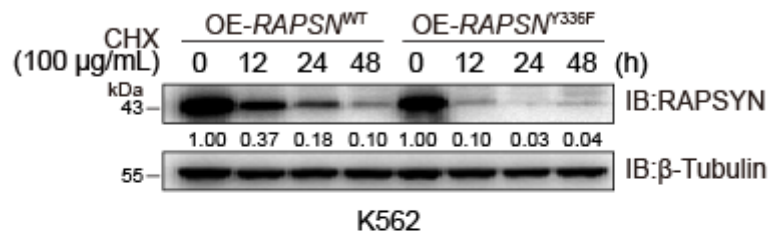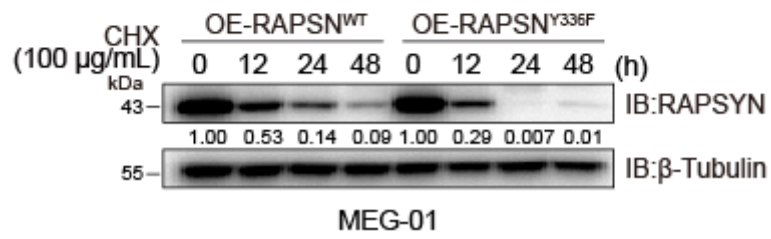

K562

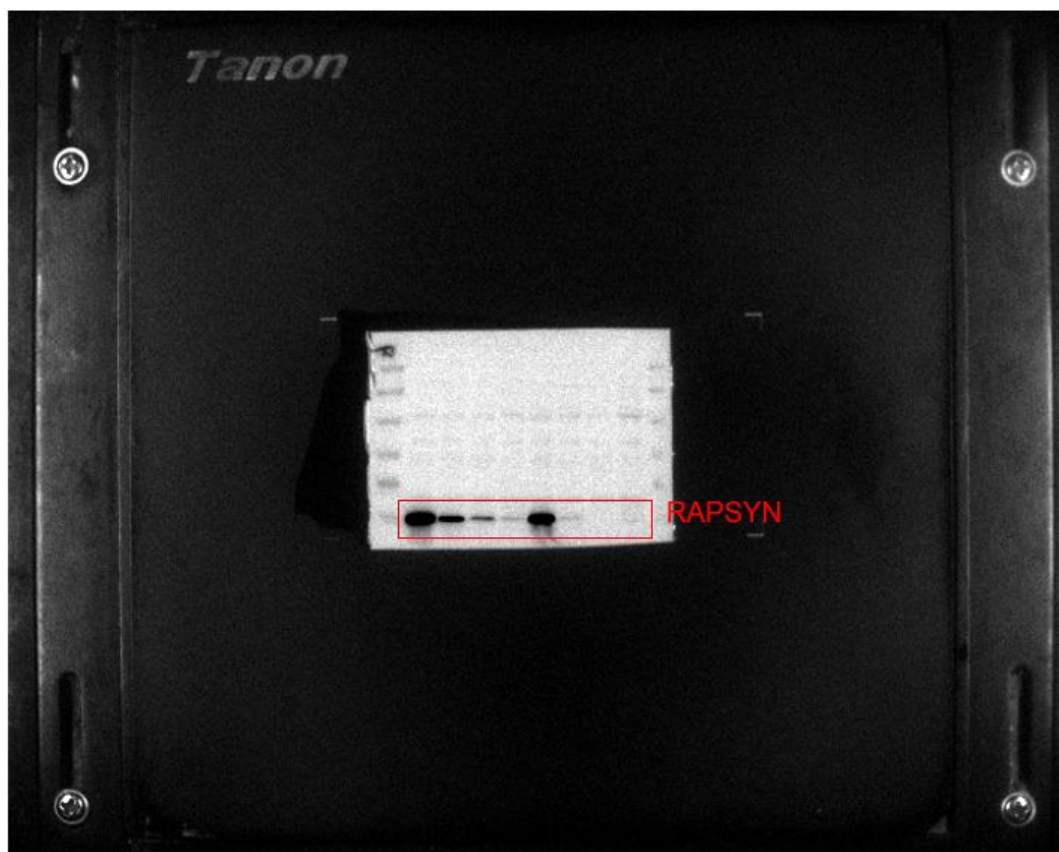

K562

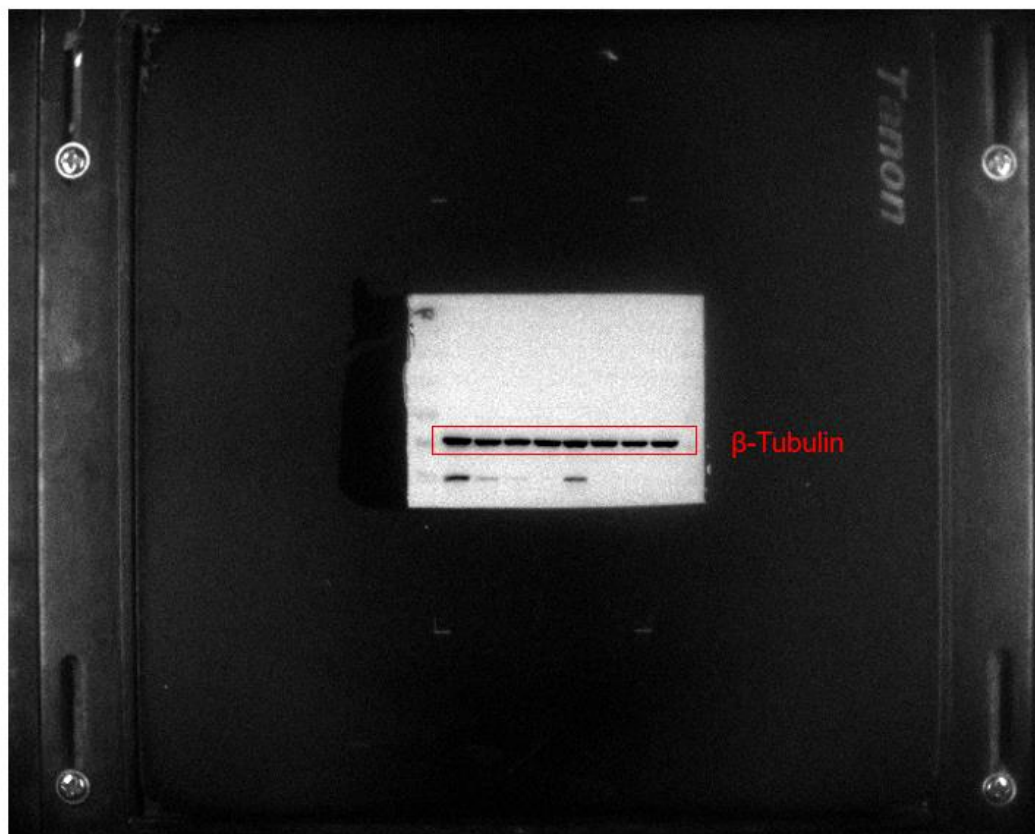

MEG-01

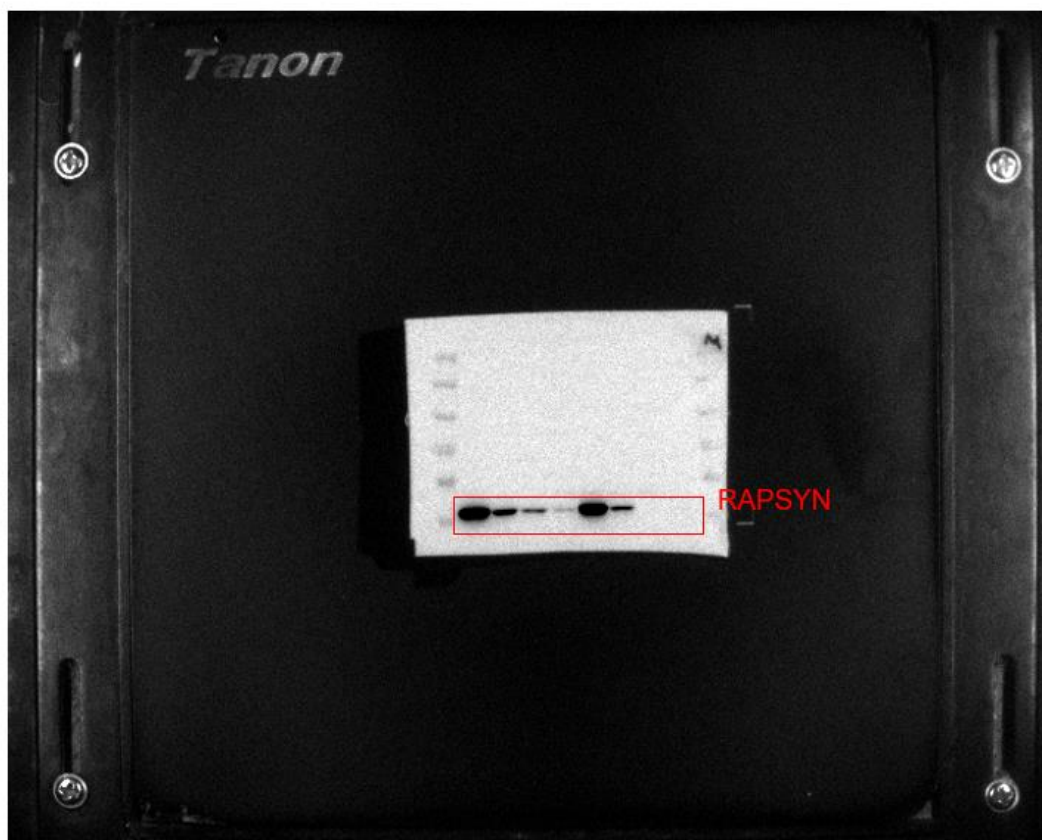

MEG-01

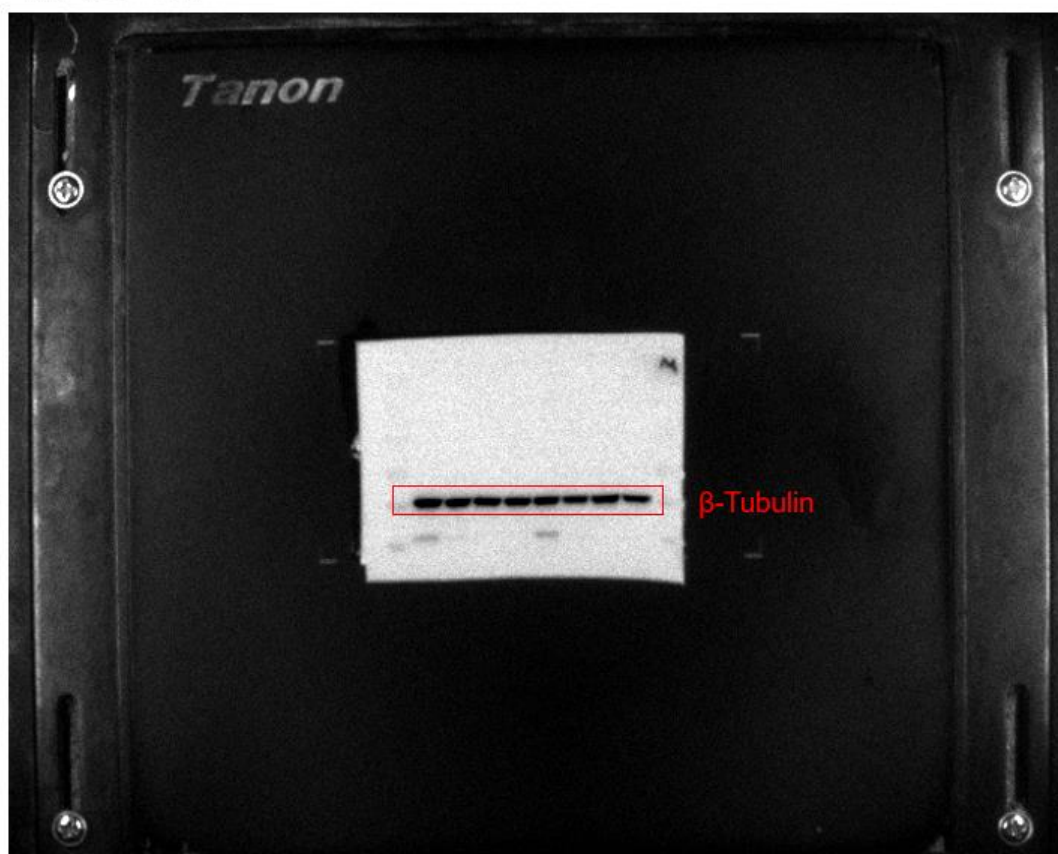

Supplement: Figure 4—source data 18. [file elife-88375-fig4-data18.zip › Figure 4-source data 18/Figure 4-source data 18.pdf]

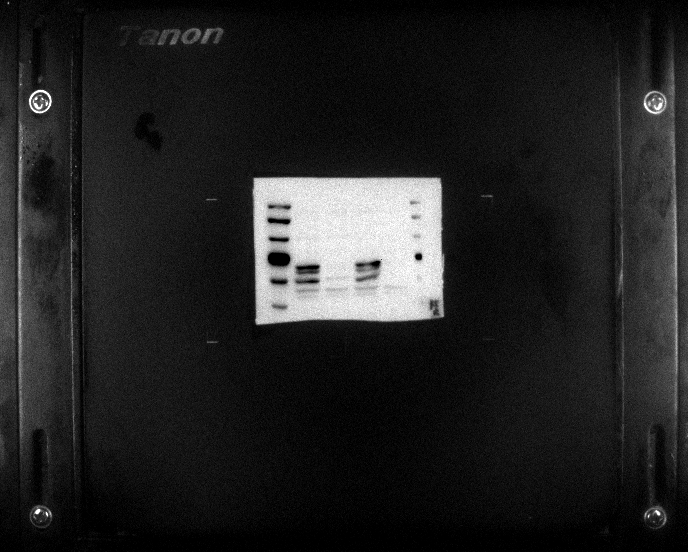

Supplement: Figure 4—source data 19. [file elife-88375-fig4-data19.zip › Figure 4-source data 19/K562 p-SRC.tif]

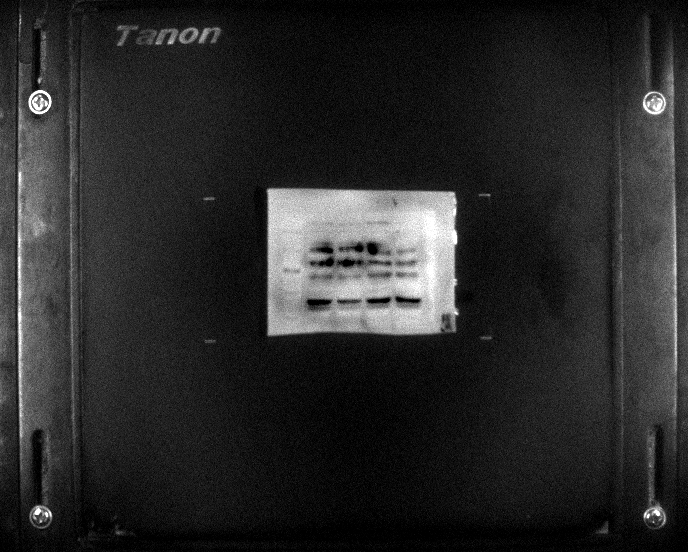

Supplement: Figure 4—source data 19. [file elife-88375-fig4-data19.zip › Figure 4-source data 19/K562 RAPSYN.tif]

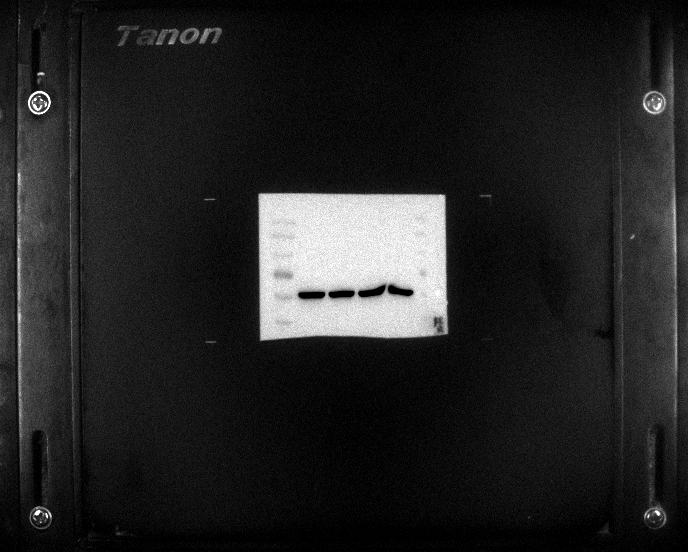

Supplement: Figure 4—source data 19. [file elife-88375-fig4-data19.zip › Figure 4-source data 19/K562 a┬-Tubulin.tif]

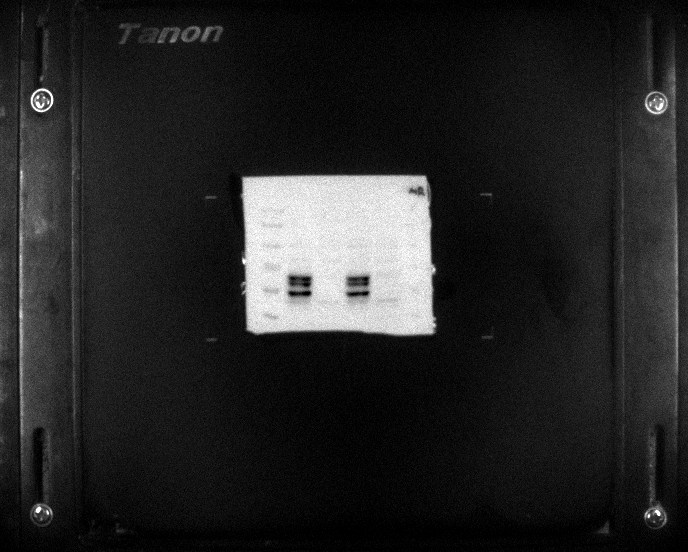

Supplement: Figure 4—source data 19. [file elife-88375-fig4-data19.zip › Figure 4-source data 19/MEG-01 p-SRC.tif]

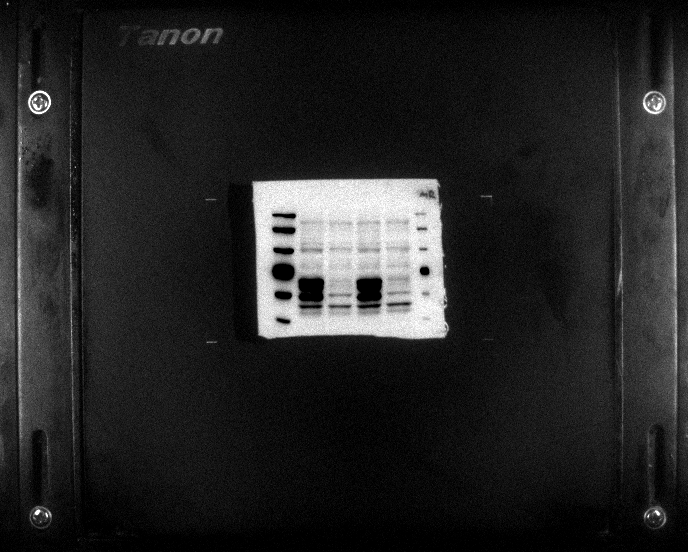

Supplement: Figure 4—source data 19. [file elife-88375-fig4-data19.zip › Figure 4-source data 19/MEG-01 RAPSYN.tif]

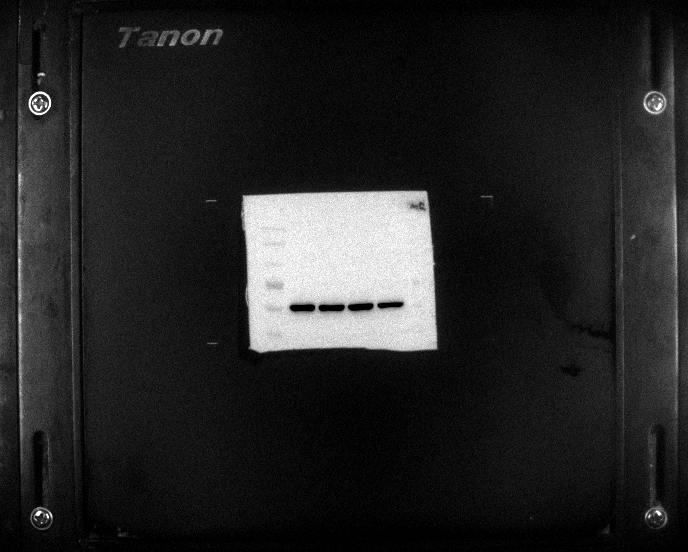

Supplement: Figure 4—source data 19. [file elife-88375-fig4-data19.zip › Figure 4-source data 19/MEG-01 a┬-Tubulin.tif]

J

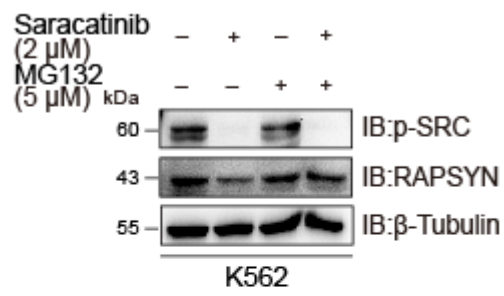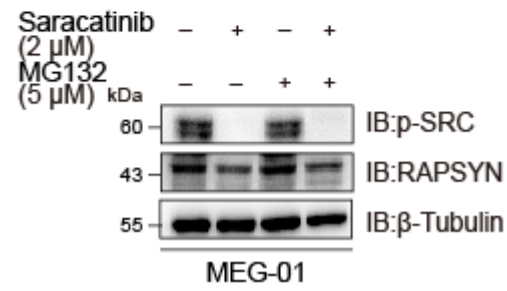

K562

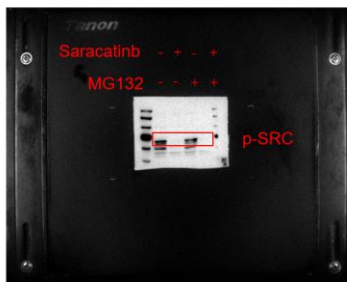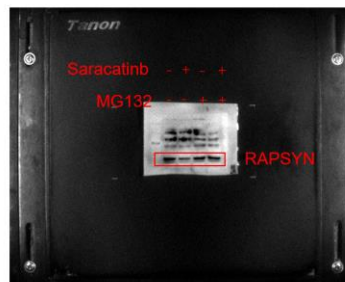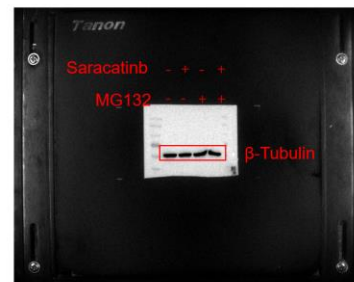

MEG-01

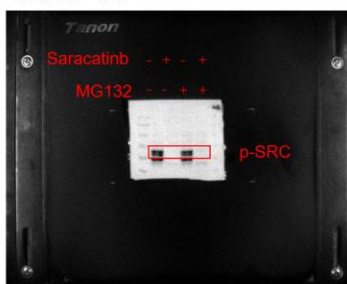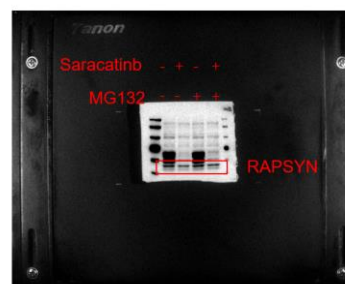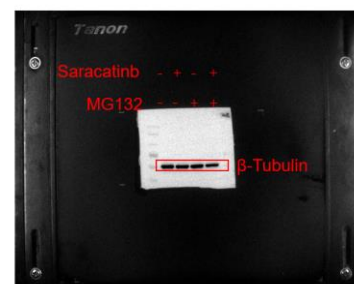

Supplement: Figure 4—source data 20. [file elife-88375-fig4-data20.zip › Figure 4-source data 20/Figure 4-source data 20.pdf]

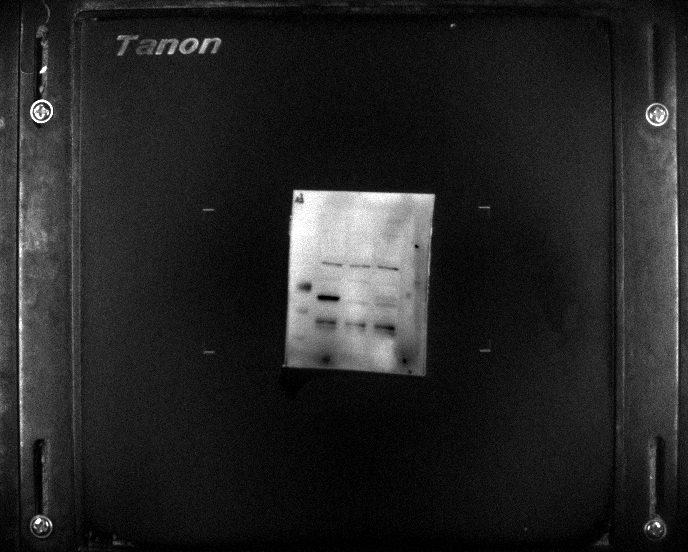

Supplement: Figure 4—source data 21. [file elife-88375-fig4-data21.zip › Figure 4-source data 21/K562 SRC.tif]

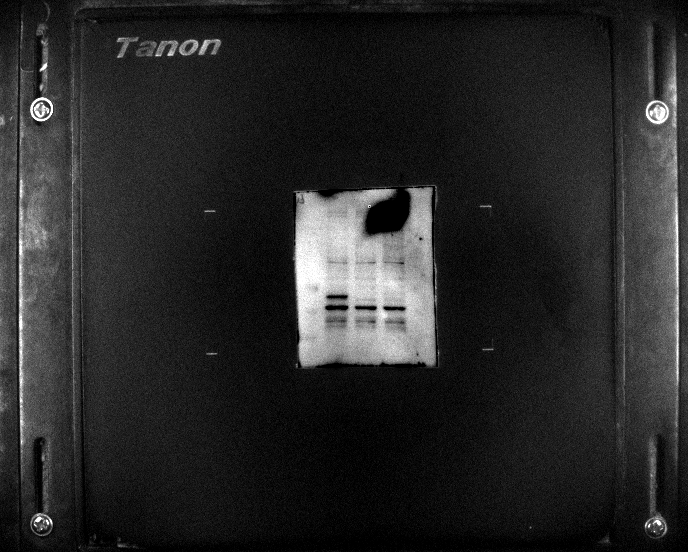

Supplement: Figure 4—source data 21. [file elife-88375-fig4-data21.zip › Figure 4-source data 21/K562 a┬-Tubulin.tif]

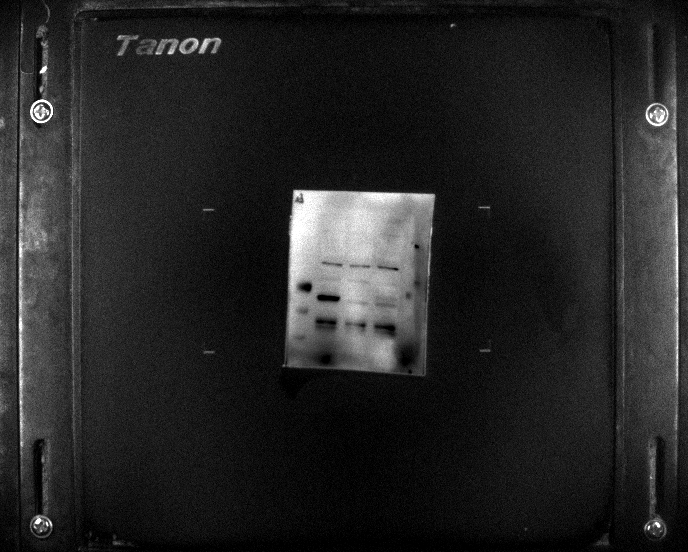

Supplement: Figure 4—source data 21. [file elife-88375-fig4-data21.zip › Figure 4-source data 21/K563 RAPSYN.tif]

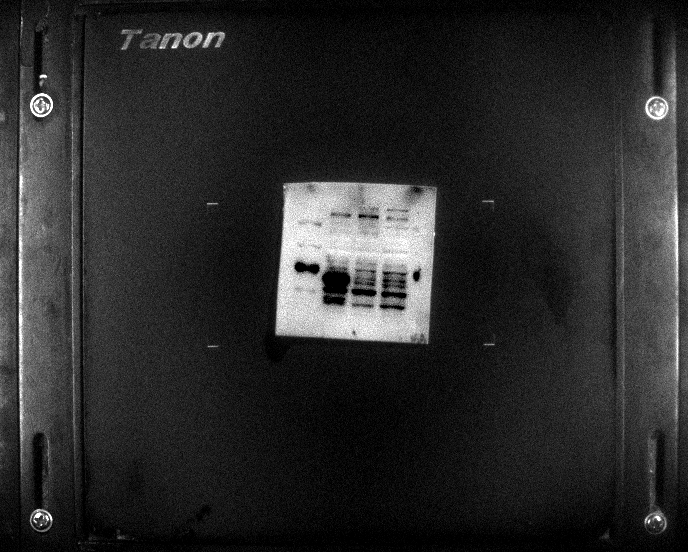

Supplement: Figure 4—source data 21. [file elife-88375-fig4-data21.zip › Figure 4-source data 21/MEG-01 RAPSYN.tif]

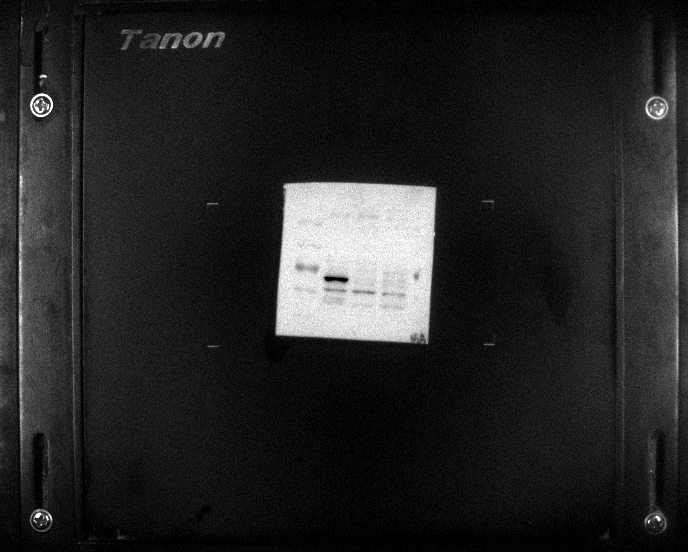

Supplement: Figure 4—source data 21. [file elife-88375-fig4-data21.zip › Figure 4-source data 21/MEG-01 SRC.tif]

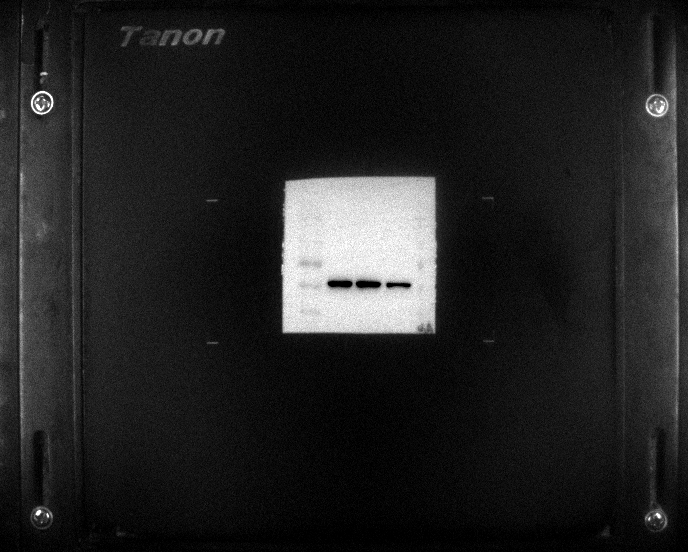

Supplement: Figure 4—source data 21. [file elife-88375-fig4-data21.zip › Figure 4-source data 21/MEG-01 a┬-Tubulin.tif]

K

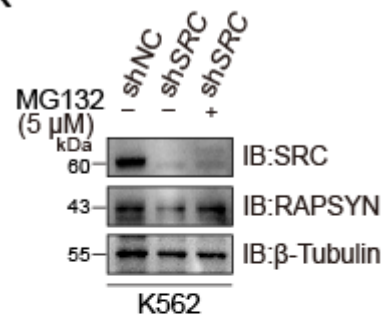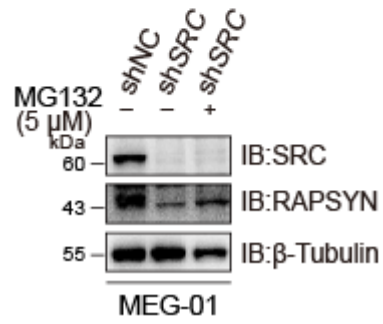

K562

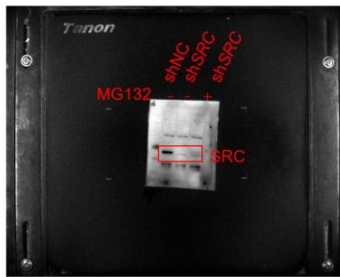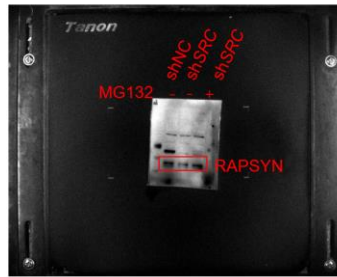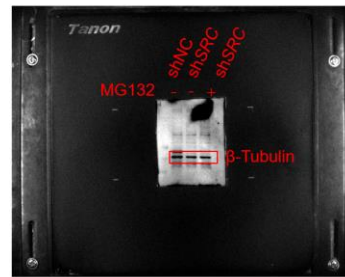

MEG-01

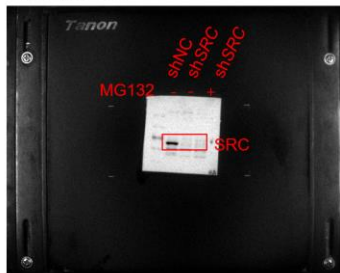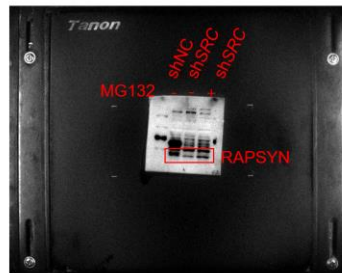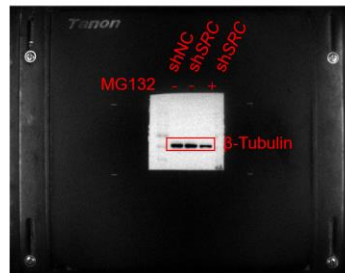

Supplement: Figure 4—source data 22. [file elife-88375-fig4-data22.zip › Figure 4-source data 22/Figure 4-source data 22.pdf]

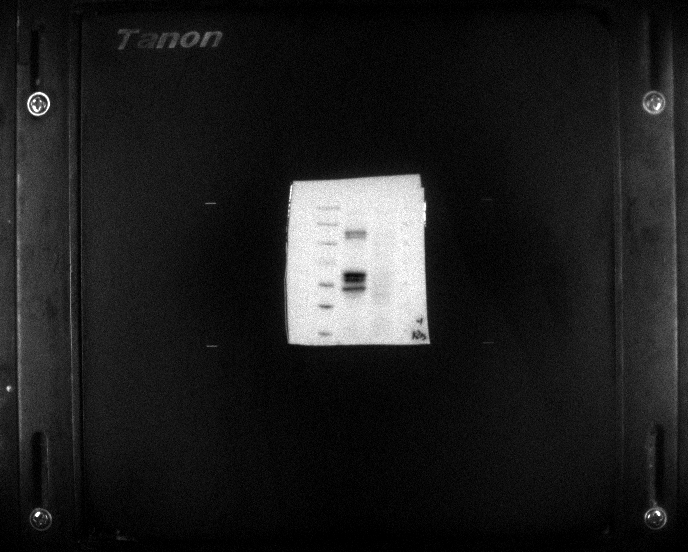

Supplement: Figure 4—figure supplement 1—source data 1. [file elife-88375-fig4-figsupp1-data1.zip › Figure supplement 5-source data 1/Input P-SRC.tif]

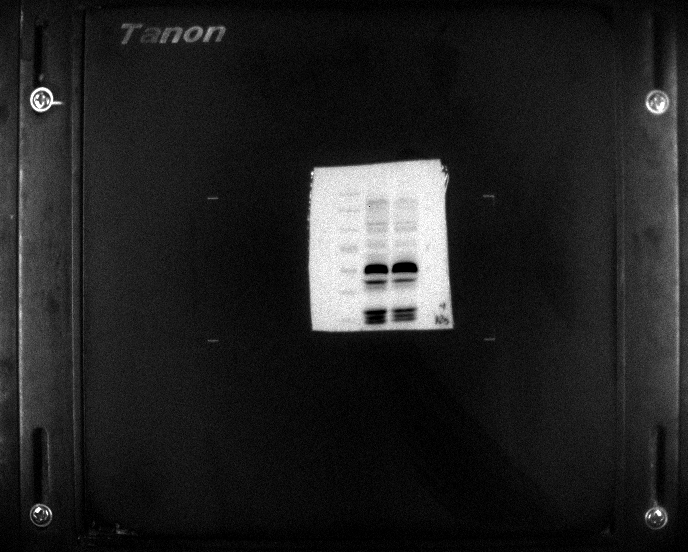

Supplement: Figure 4—figure supplement 1—source data 1. [file elife-88375-fig4-figsupp1-data1.zip › Figure supplement 5-source data 1/Input RAPSYN.tif]

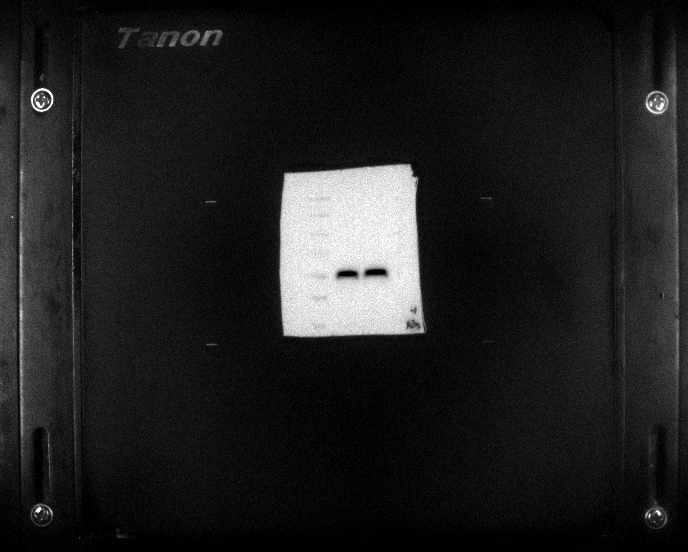

Supplement: Figure 4—figure supplement 1—source data 1. [file elife-88375-fig4-figsupp1-data1.zip › Figure supplement 5-source data 1/Input a┬-Tubulin.tif]

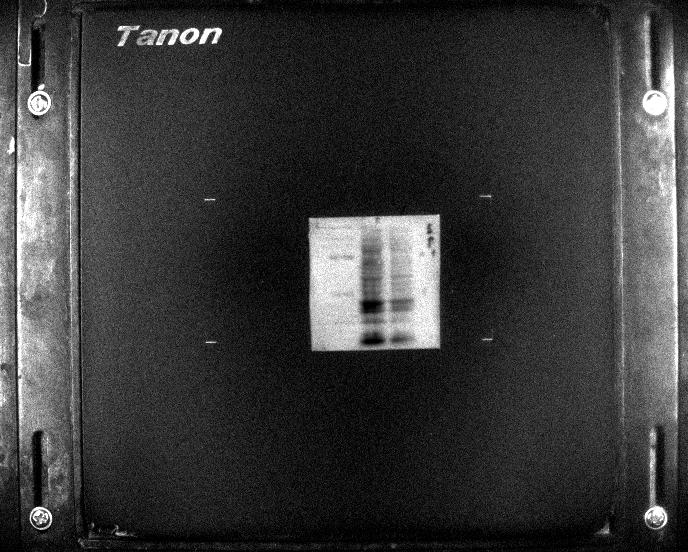

Supplement: Figure 4—figure supplement 1—source data 1. [file elife-88375-fig4-figsupp1-data1.zip › Figure supplement 5-source data 1/IP P-Tyr-IB RAPSYN.tif]

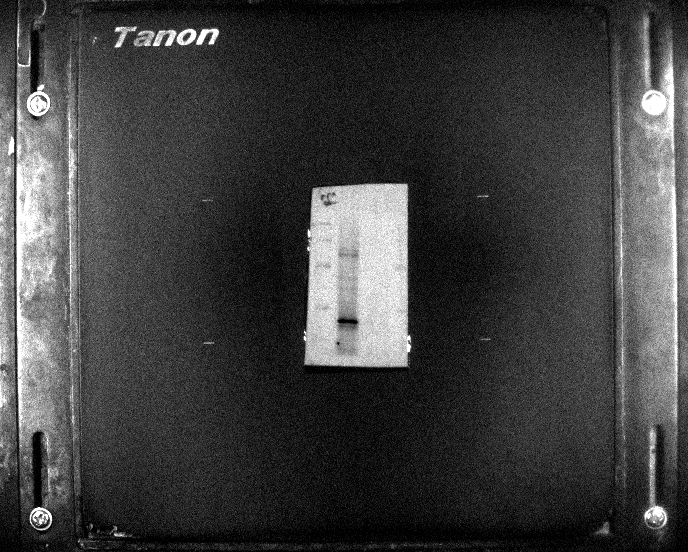

Supplement: Figure 4—figure supplement 1—source data 1. [file elife-88375-fig4-figsupp1-data1.zip › Figure supplement 5-source data 1/IP RAPSYN-IB P-Tyr.tif]

A

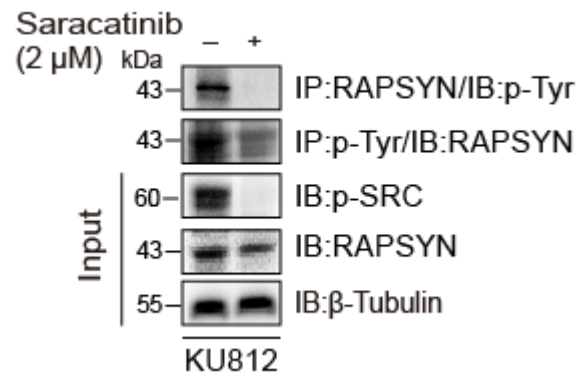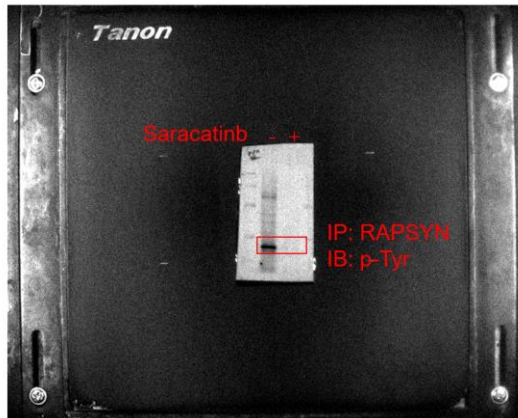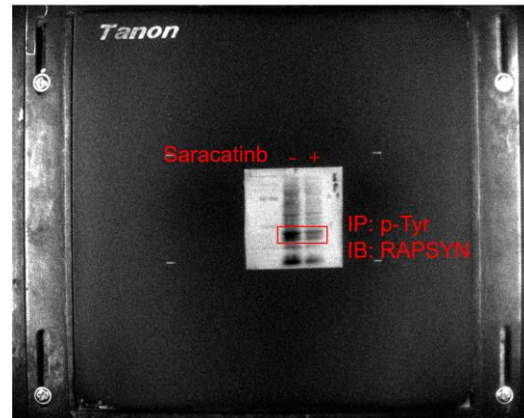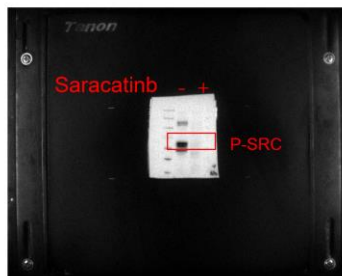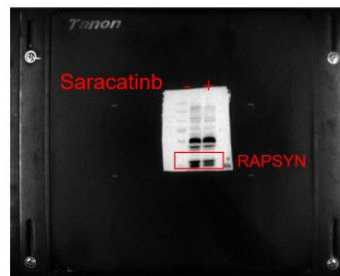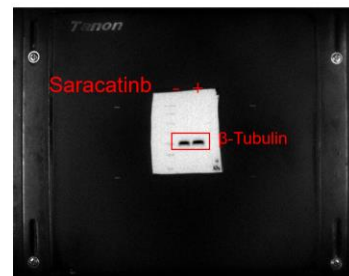

Supplement: Figure 4—figure supplement 1—source data 2. [file elife-88375-fig4-figsupp1-data2.zip › Figure supplement 5-source data 2/Figure supplement 5-source data 2.pdf]

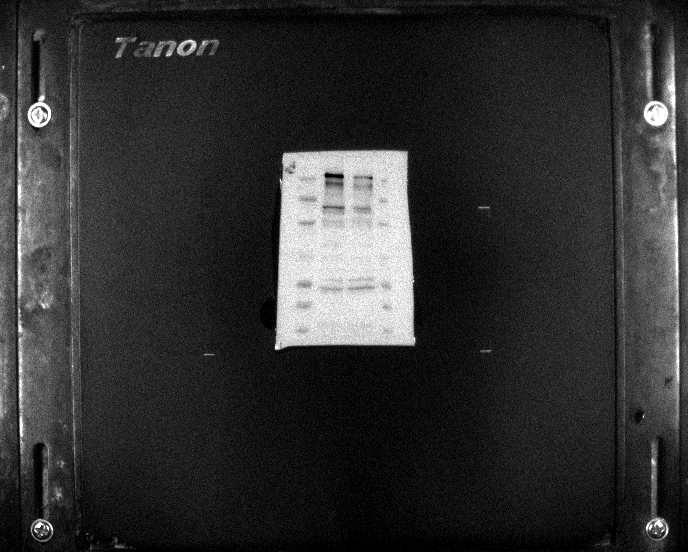

Supplement: Figure 5—source data 1. [file elife-88375-fig5-data1.zip › Figure 5-source data 1/K562 Input BCR-ABL.tif]

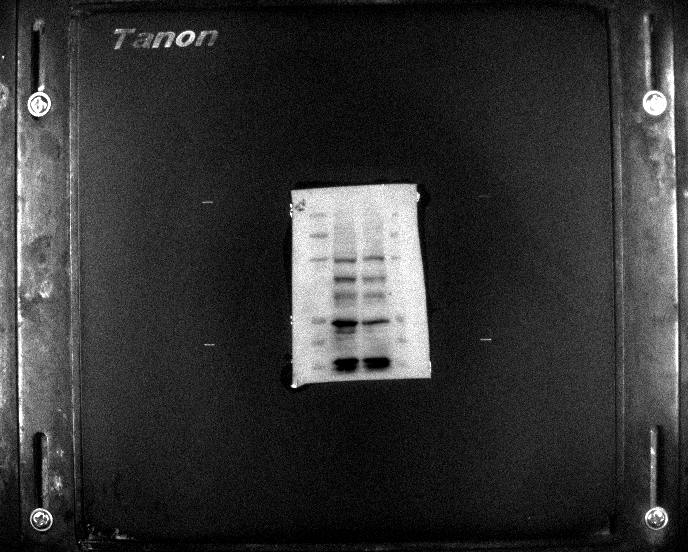

Supplement: Figure 5—source data 1. [file elife-88375-fig5-data1.zip › Figure 5-source data 1/K562 Input RAPSYN.tif]

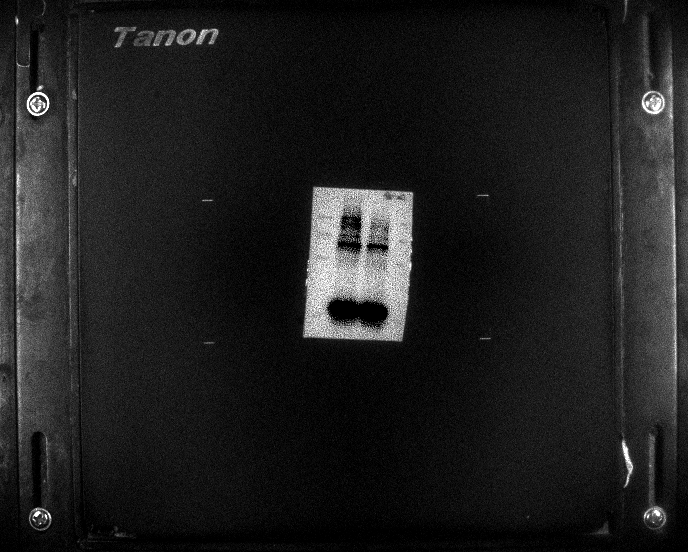

Supplement: Figure 5—source data 1. [file elife-88375-fig5-data1.zip › Figure 5-source data 1/K562 IP BCR-ABL-IB BCR-ABL.tif]

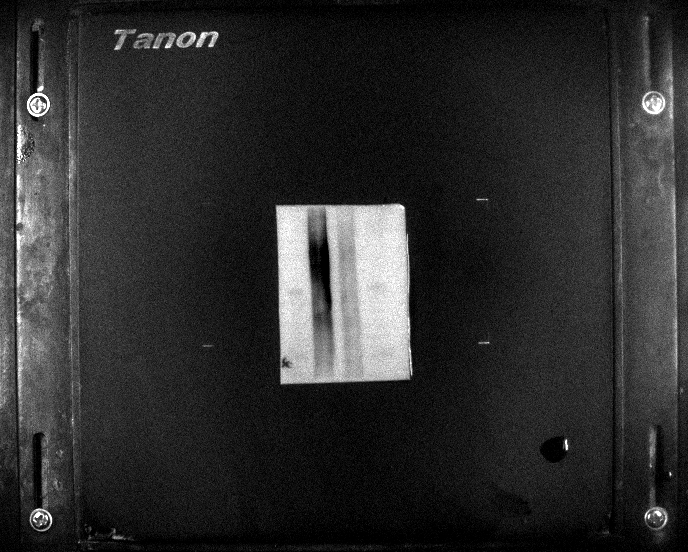

Supplement: Figure 5—source data 1. [file elife-88375-fig5-data1.zip › Figure 5-source data 1/K562 IP BCR-ABL-IB NEDD8.tif]

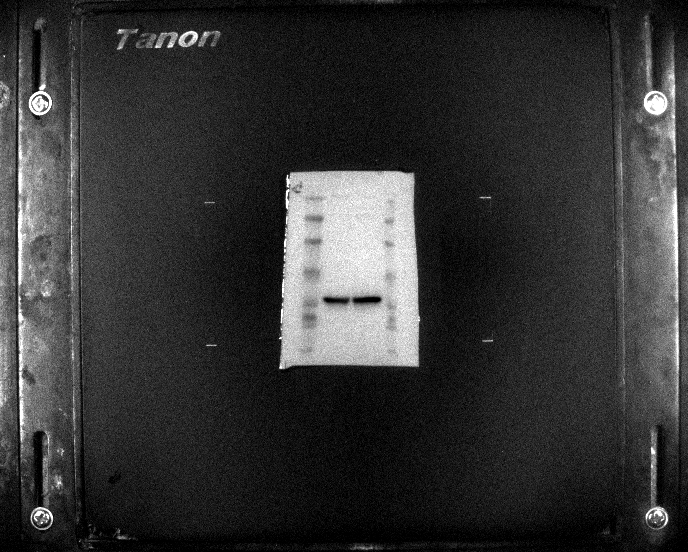

Supplement: Figure 5—source data 1. [file elife-88375-fig5-data1.zip › Figure 5-source data 1/K562 a┬-Tubulin.tif]

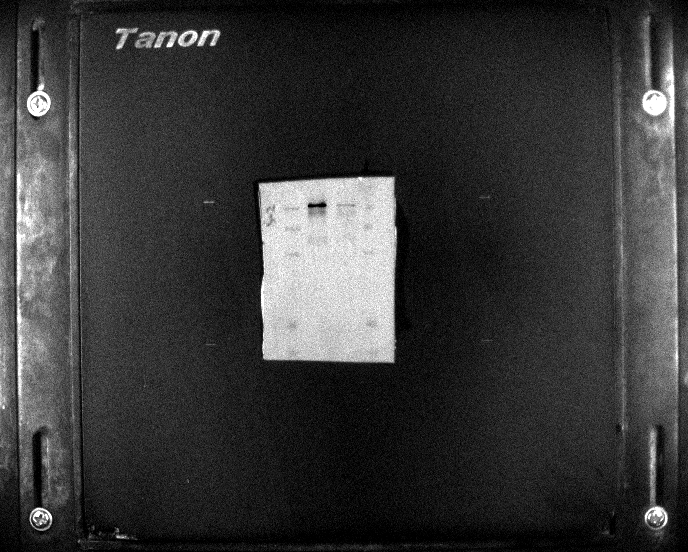

Supplement: Figure 5—source data 1. [file elife-88375-fig5-data1.zip › Figure 5-source data 1/MEG-01 Input BCR-ABL.tif]

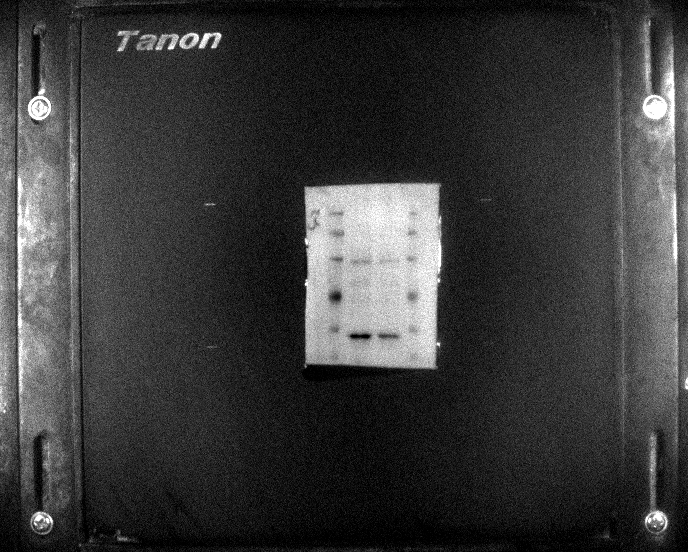

Supplement: Figure 5—source data 1. [file elife-88375-fig5-data1.zip › Figure 5-source data 1/MEG-01 Input RAPSYN.tif]

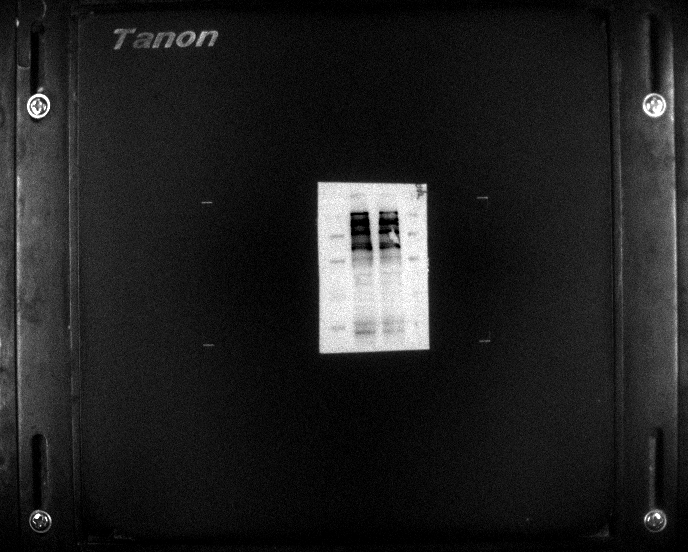

Supplement: Figure 5—source data 1. [file elife-88375-fig5-data1.zip › Figure 5-source data 1/MEG-01 IP BCR-ABL-IB BCR-ABL.tif]

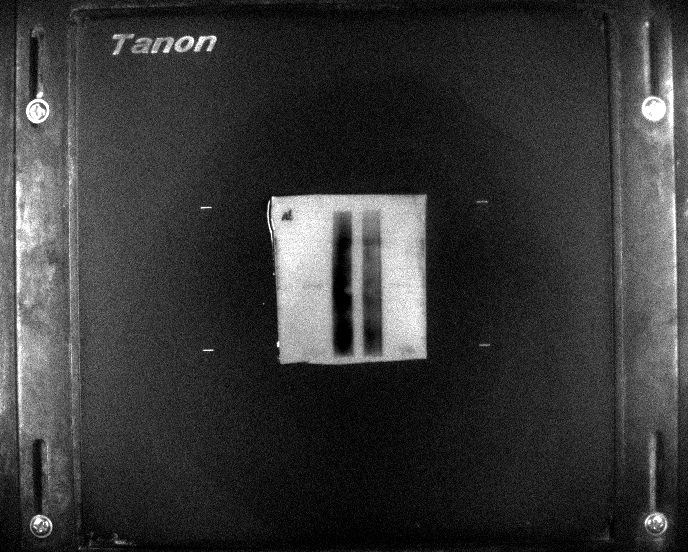

Supplement: Figure 5—source data 1. [file elife-88375-fig5-data1.zip › Figure 5-source data 1/MEG-01 IP BCR-ABL-IB NEDD8.tif]

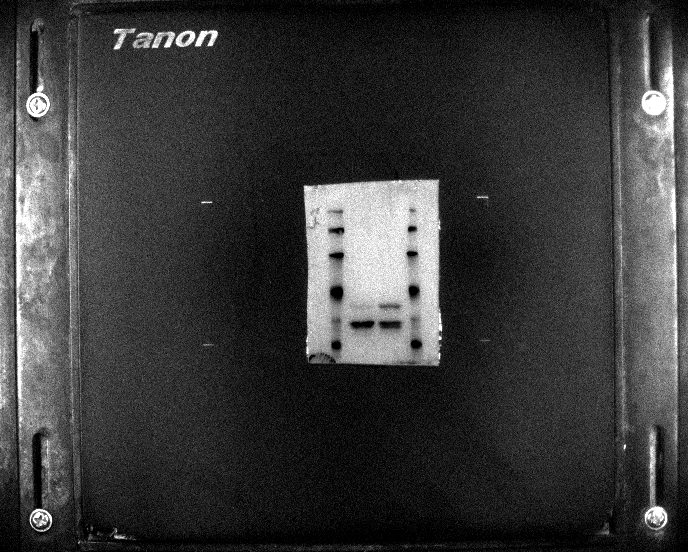

Supplement: Figure 5—source data 1. [file elife-88375-fig5-data1.zip › Figure 5-source data 1/MEG-01 a┬-Tubulin.tif]

A

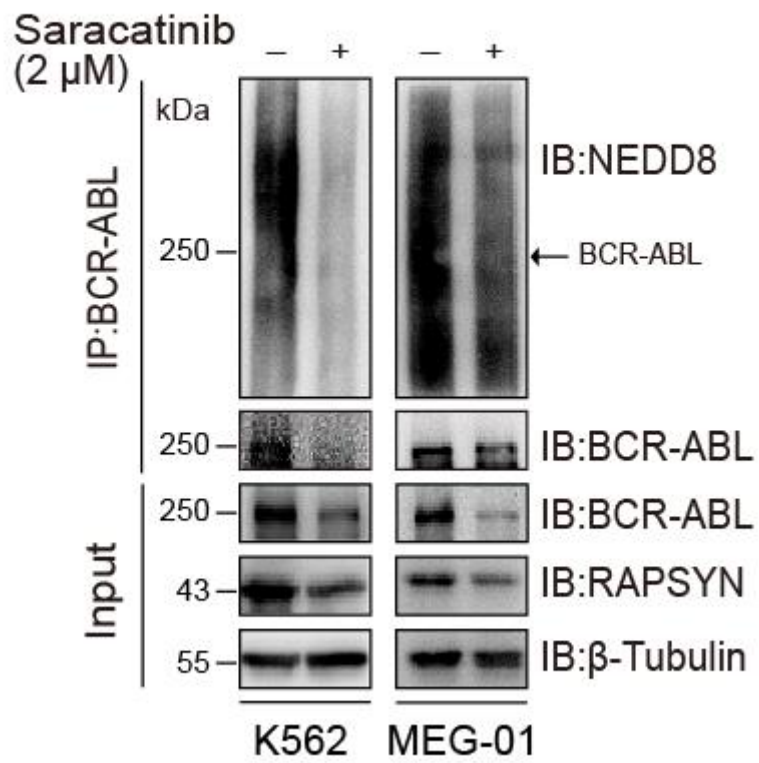

K562

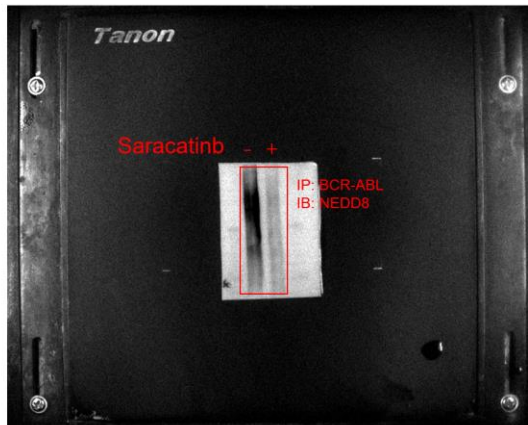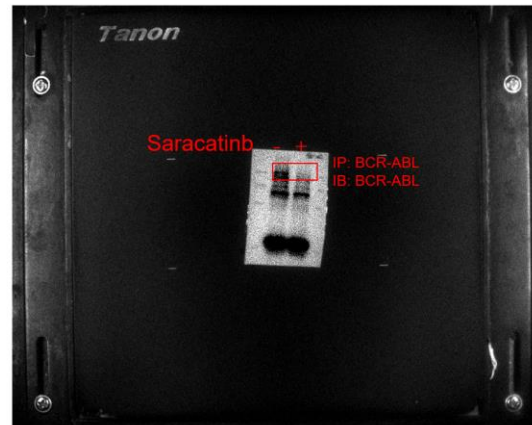

Input

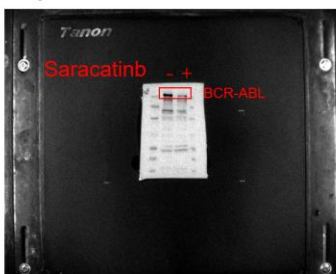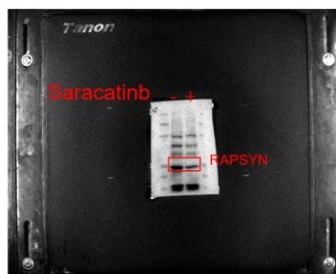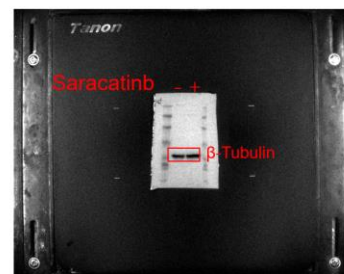

MEG-01

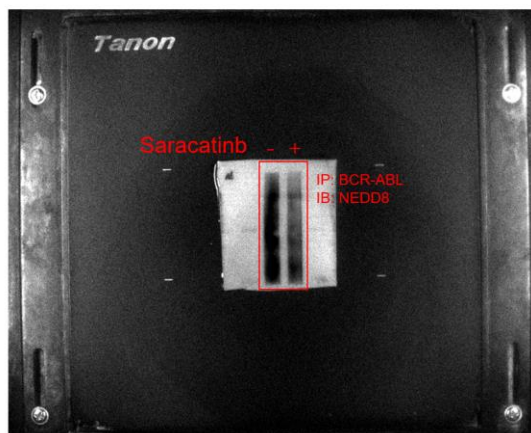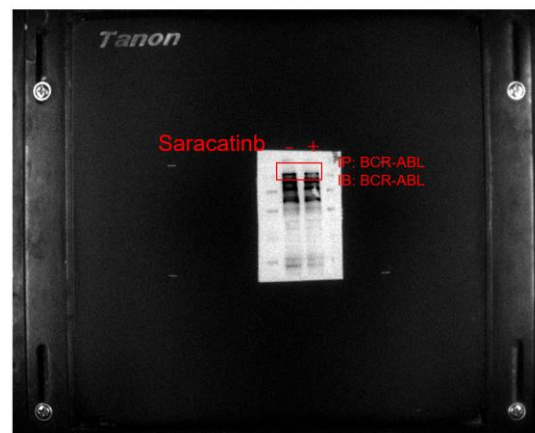

Input

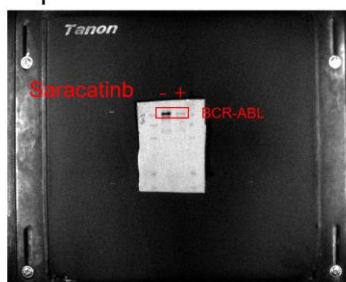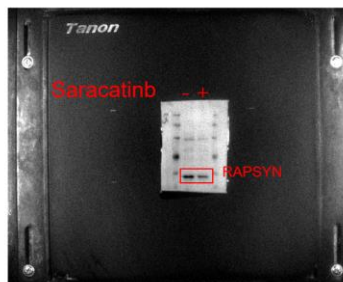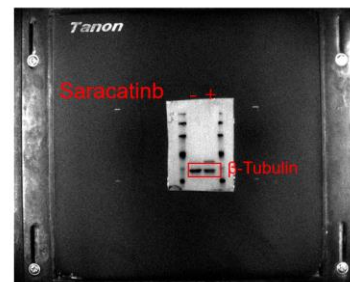

Supplement: Figure 5—source data 2. [file elife-88375-fig5-data2.zip › Figure 5-source data 2/Figure 5-source data 2.pdf]

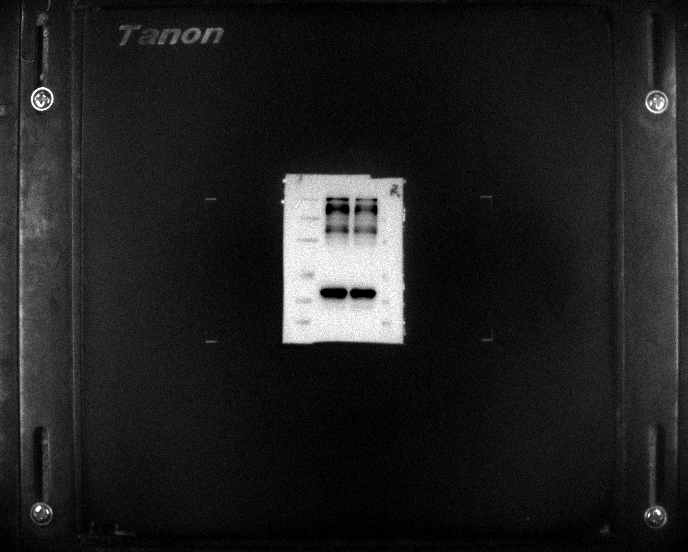

Supplement: Figure 5—source data 3. [file elife-88375-fig5-data3.zip › Figure 5-source data 3/K562 Input BCR-ABL-2.tif]

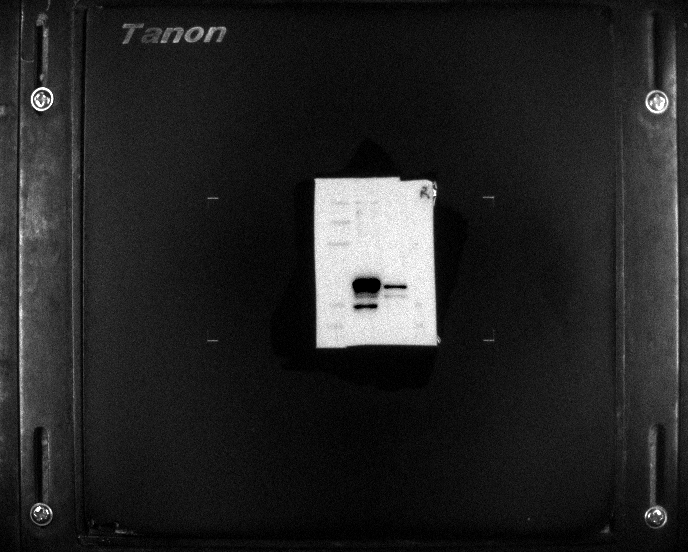

Supplement: Figure 5—source data 3. [file elife-88375-fig5-data3.zip › Figure 5-source data 3/K562 Input RAPSYN.tif]

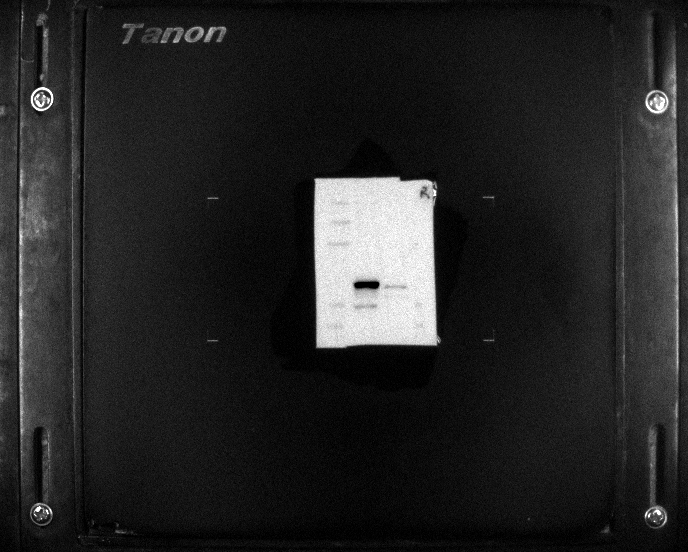

Supplement: Figure 5—source data 3. [file elife-88375-fig5-data3.zip › Figure 5-source data 3/K562 Input SRC.tif]

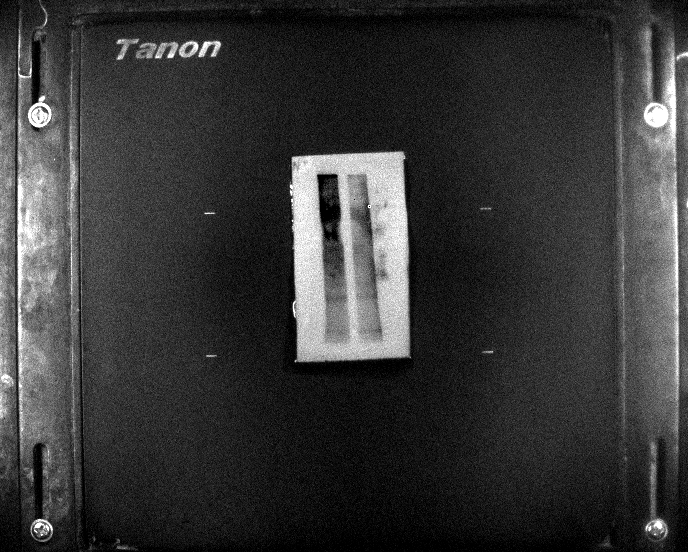

Supplement: Figure 5—source data 3. [file elife-88375-fig5-data3.zip › Figure 5-source data 3/K562 IP BCR-ABL-IB NEDD8.tif]

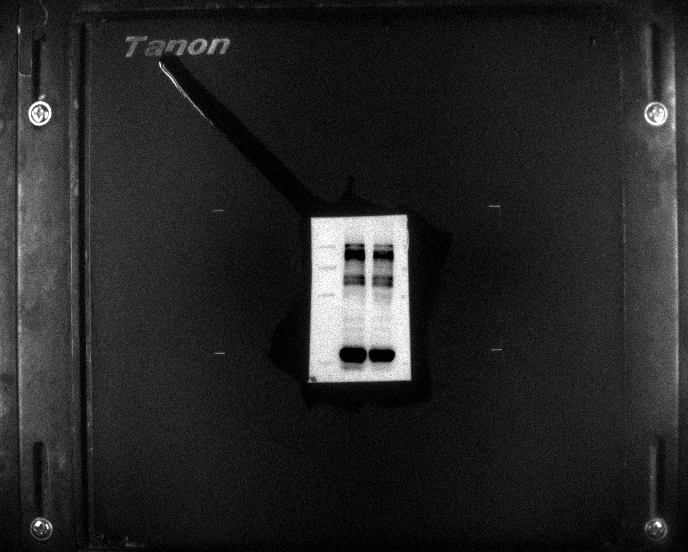

Supplement: Figure 5—source data 3. [file elife-88375-fig5-data3.zip › Figure 5-source data 3/K562 IP BCR-ABL-IB BCR-ABL.tif]

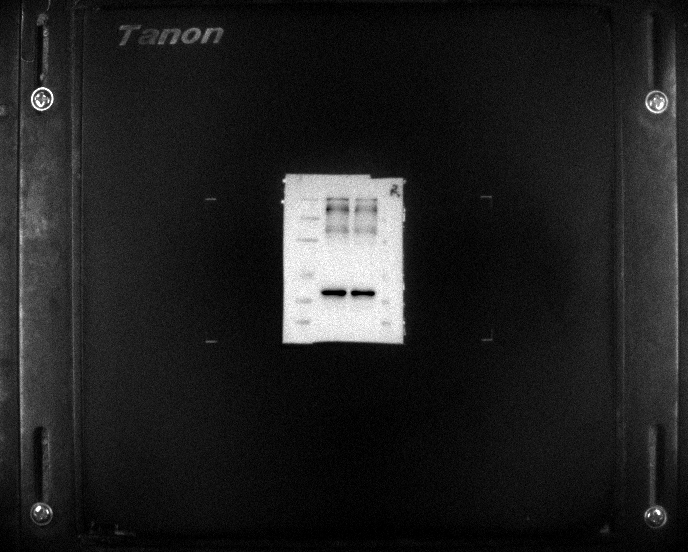

Supplement: Figure 5—source data 3. [file elife-88375-fig5-data3.zip › Figure 5-source data 3/K562 a┬-Tubulin.tif]

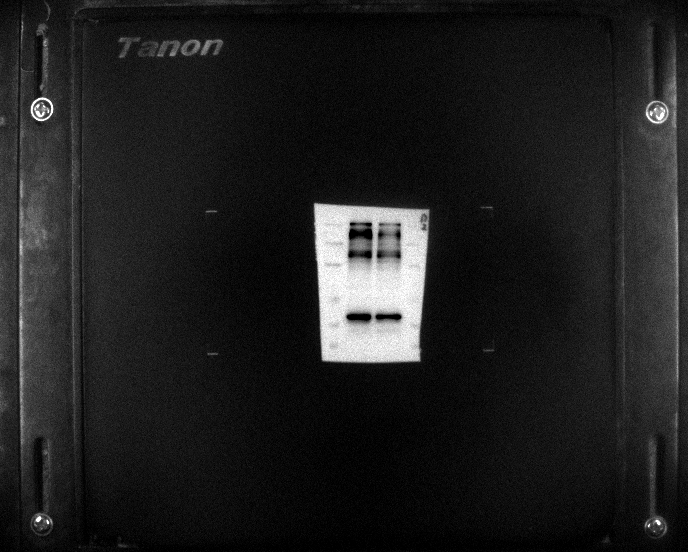

Supplement: Figure 5—source data 3. [file elife-88375-fig5-data3.zip › Figure 5-source data 3/MEG-01 Input BCR-ABL.tif]

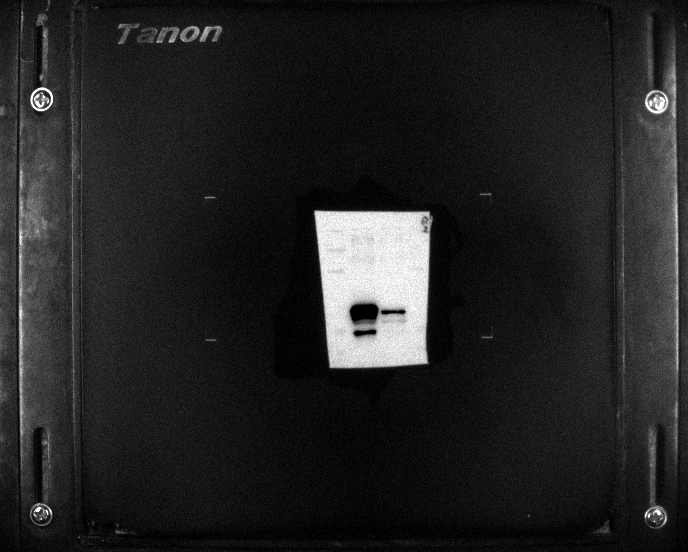

Supplement: Figure 5—source data 3. [file elife-88375-fig5-data3.zip › Figure 5-source data 3/MEG-01 Input RAPSYN.tif]

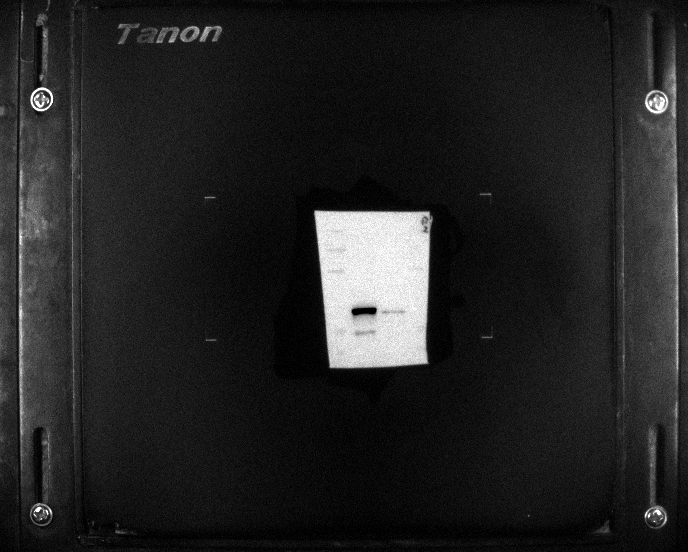

Supplement: Figure 5—source data 3. [file elife-88375-fig5-data3.zip › Figure 5-source data 3/MEG-01 Input SRC.tif]

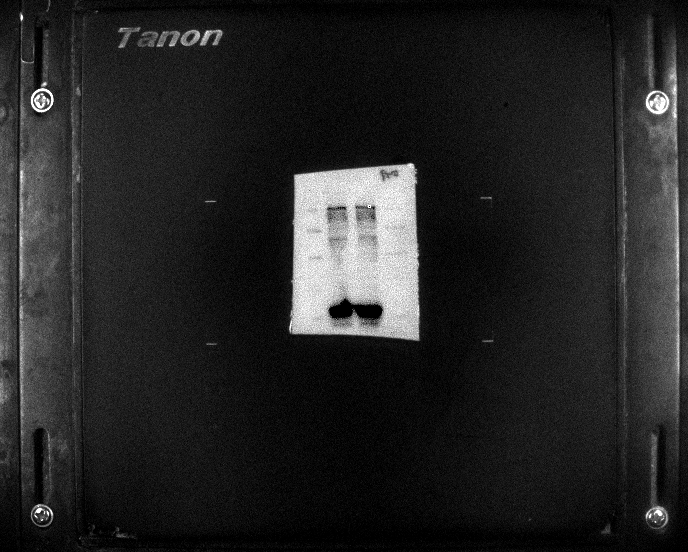

Supplement: Figure 5—source data 3. [file elife-88375-fig5-data3.zip › Figure 5-source data 3/MEG-01 IP BCR-ABL-IB BCR-ABL.tif]

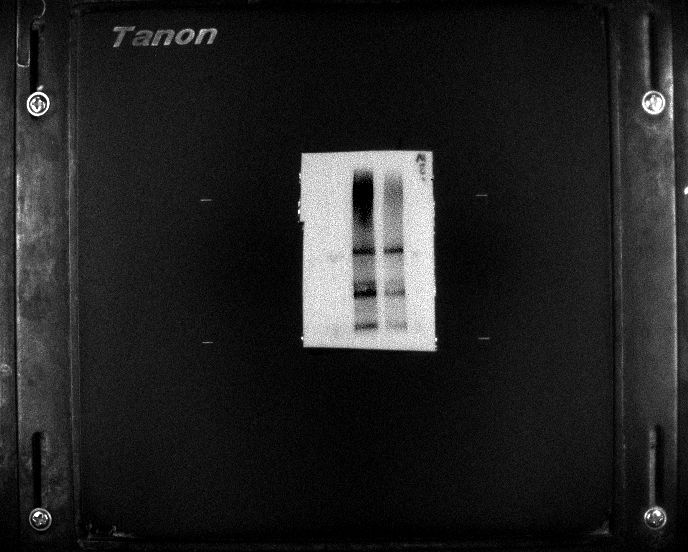

Supplement: Figure 5—source data 3. [file elife-88375-fig5-data3.zip › Figure 5-source data 3/MEG-01 IP BCR-ABL-IB NEDD8.tif]

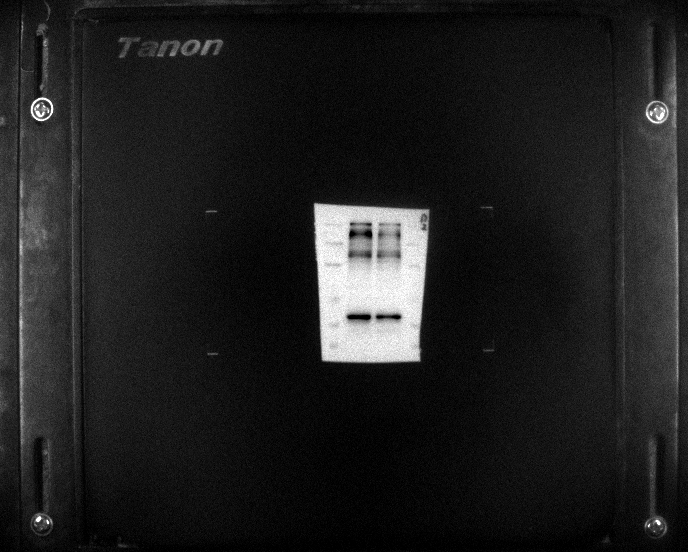

Supplement: Figure 5—source data 3. [file elife-88375-fig5-data3.zip › Figure 5-source data 3/MEG-01 a┬-Tubulin.tif]

**B**

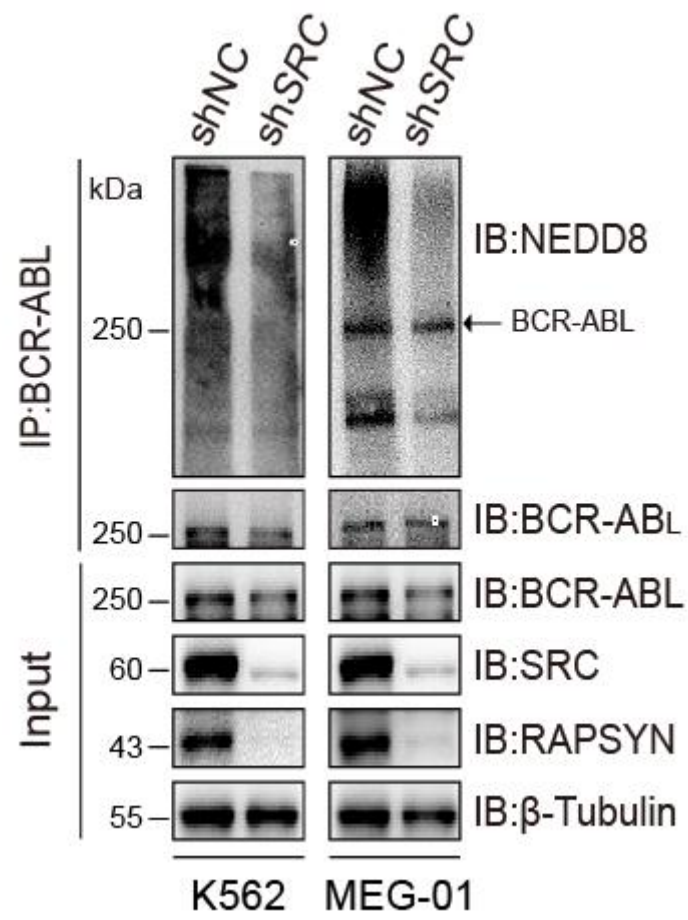

K562

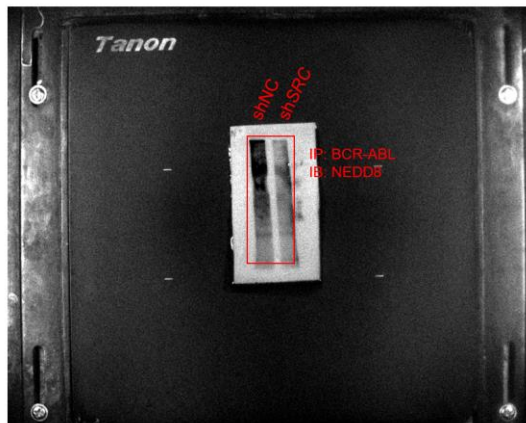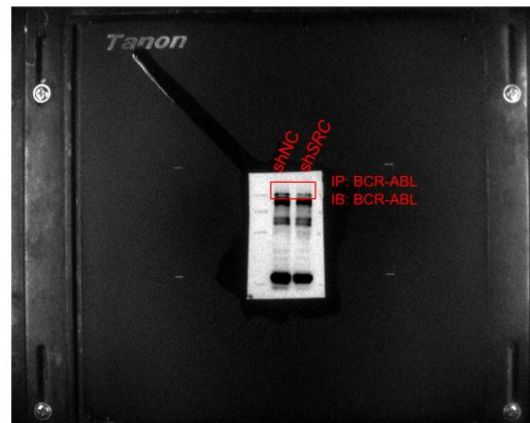

## Input

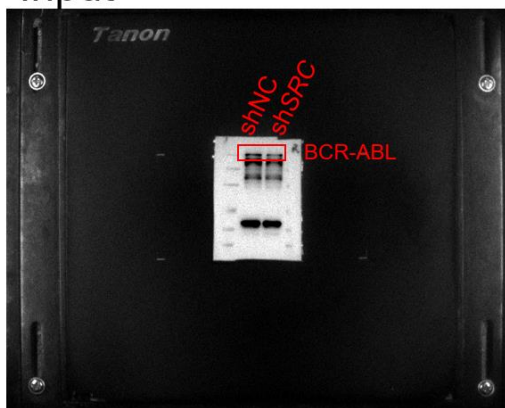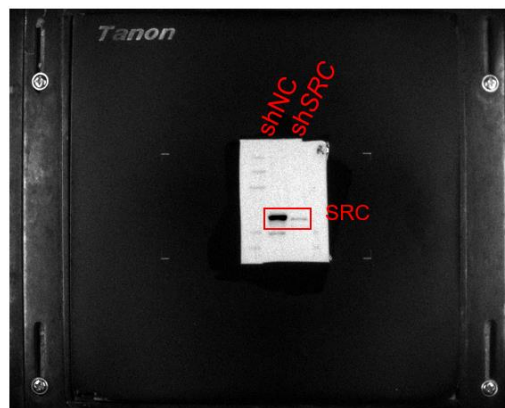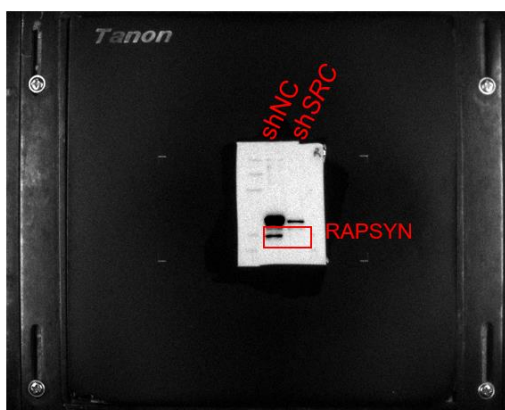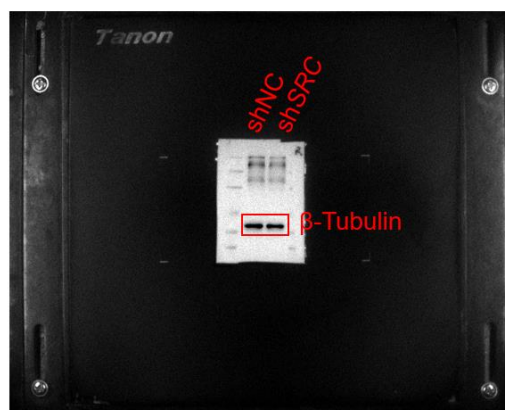

## MEG-01

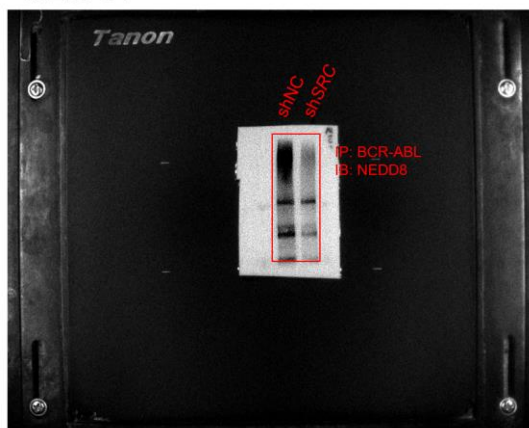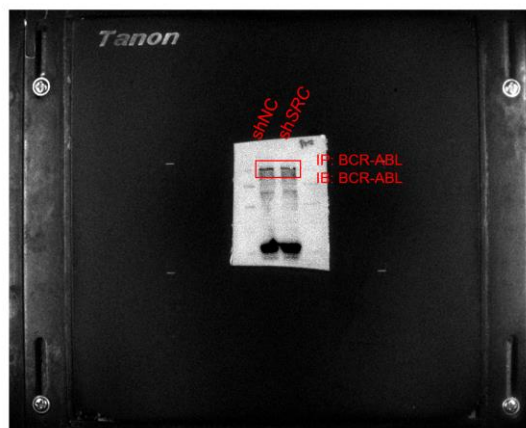

## Input

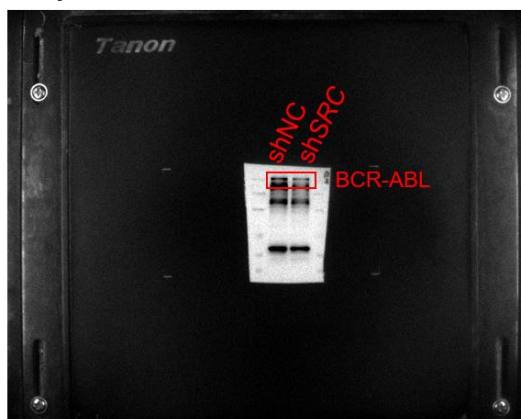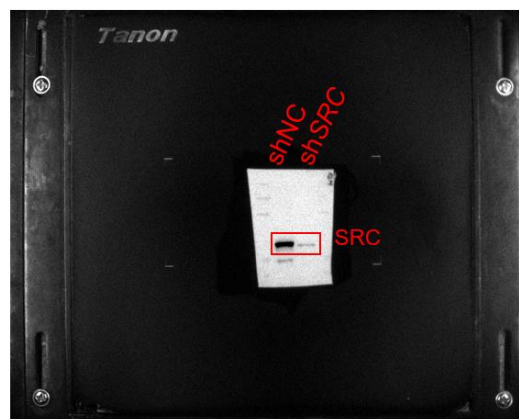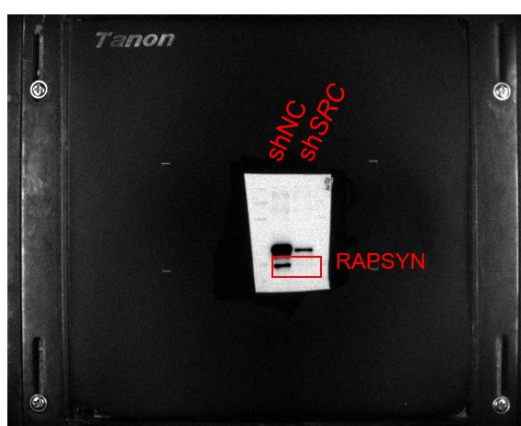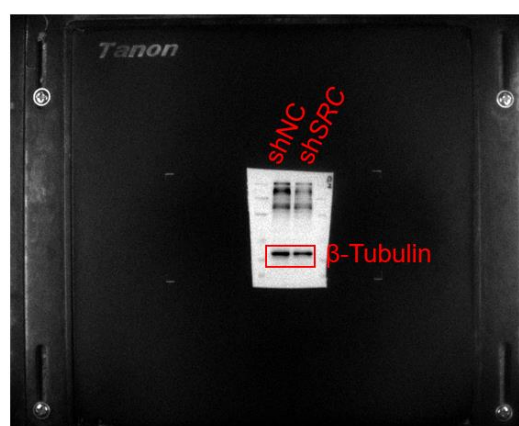

Supplement: Figure 5—source data 4. [file elife-88375-fig5-data4.zip › Figure 5-source data 4/Figure 5-source data 4.pdf]

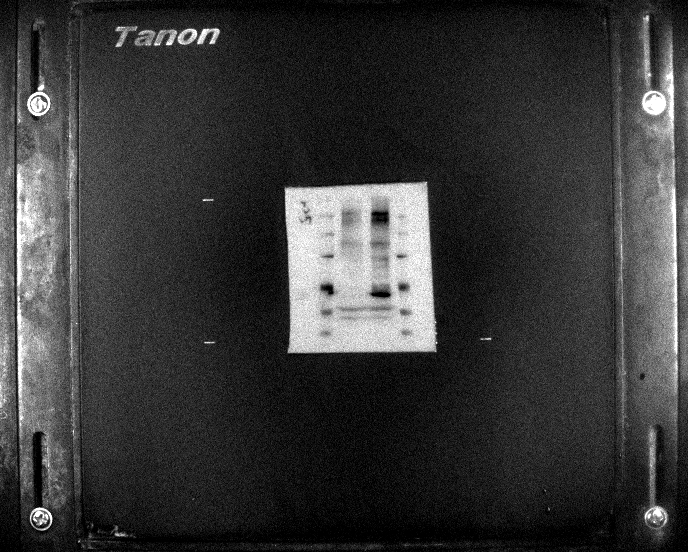

Supplement: Figure 5—source data 5. [file elife-88375-fig5-data5.zip › Figure 5-source data 5/K562 Input BCR-ABL.tif]

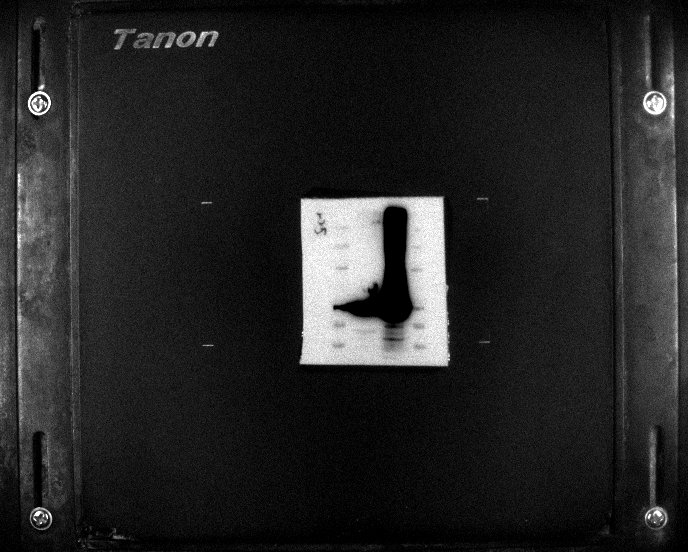

Supplement: Figure 5—source data 5. [file elife-88375-fig5-data5.zip › Figure 5-source data 5/K562 Input RAPSYN.tif]

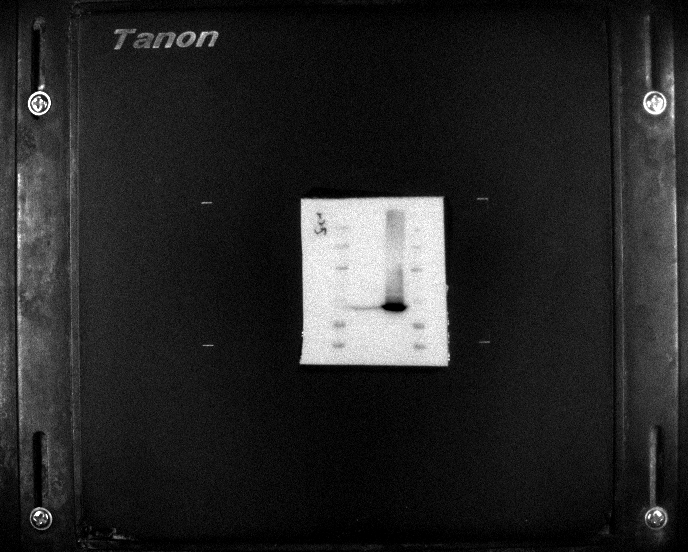

Supplement: Figure 5—source data 5. [file elife-88375-fig5-data5.zip › Figure 5-source data 5/K562 Input SRC.tif]

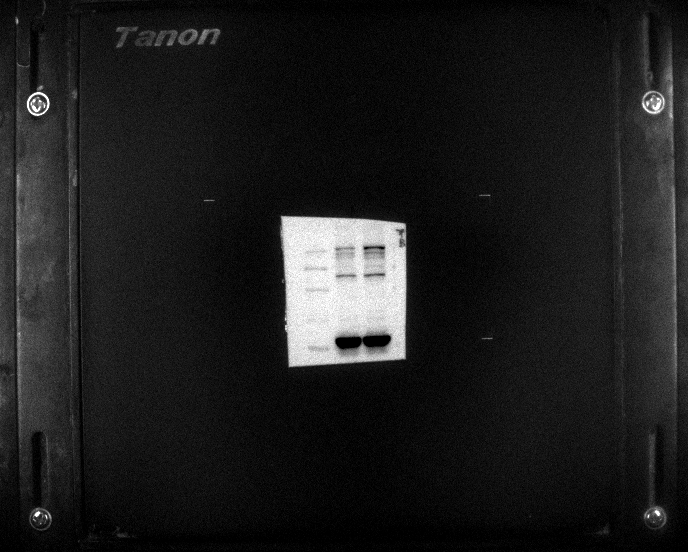

Supplement: Figure 5—source data 5. [file elife-88375-fig5-data5.zip › Figure 5-source data 5/K562 IP BCR-ABL-IB BCR-ABL.tif]

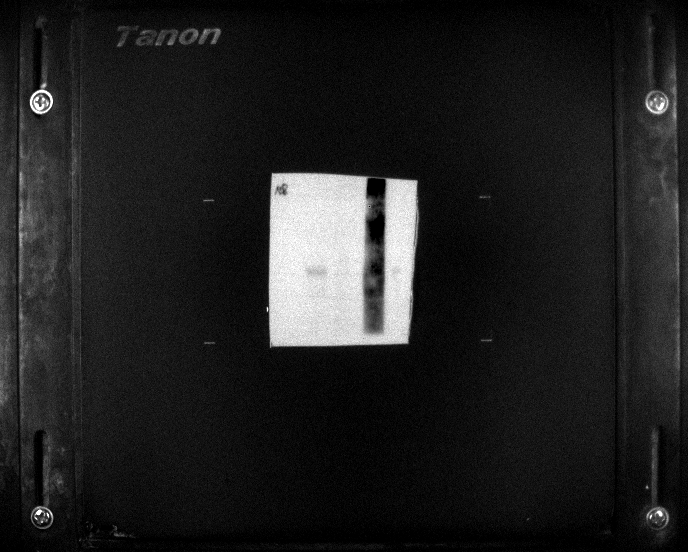

Supplement: Figure 5—source data 5. [file elife-88375-fig5-data5.zip › Figure 5-source data 5/K562 IP BCR-ABL-IB NEDD8.tif]

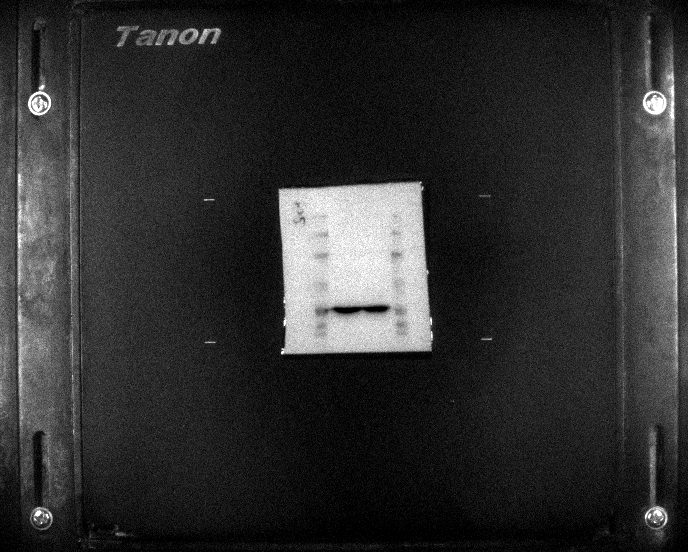

Supplement: Figure 5—source data 5. [file elife-88375-fig5-data5.zip › Figure 5-source data 5/K562 a┬-Tubulin.tif]

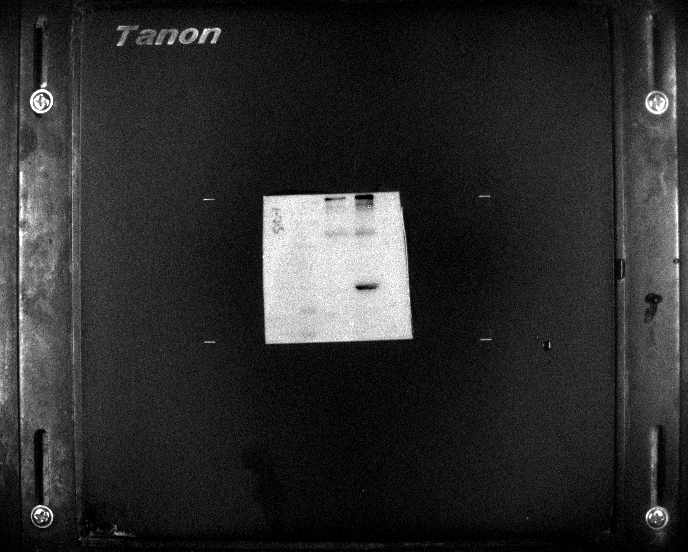

Supplement: Figure 5—source data 5. [file elife-88375-fig5-data5.zip › Figure 5-source data 5/MEG-01 Input BCR-ABL.tif]

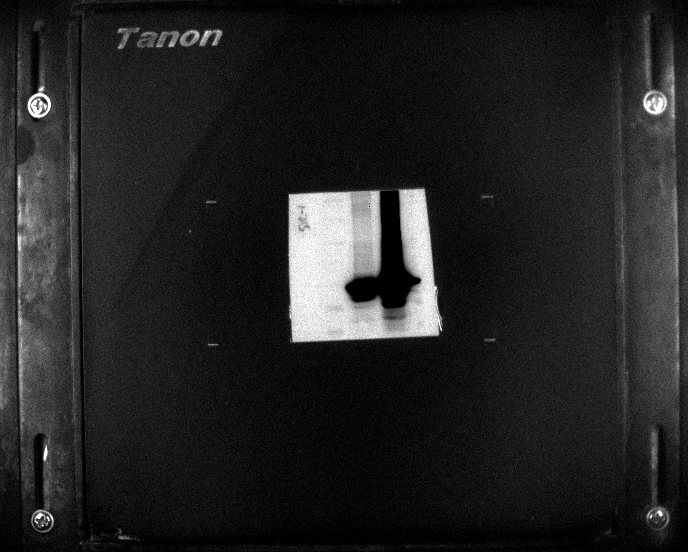

Supplement: Figure 5—source data 5. [file elife-88375-fig5-data5.zip › Figure 5-source data 5/MEG-01 Input RAPSYN.tif]
