# Supplementary figures and images for: RAPSYN-mediated neddylation of BCR-ABL alternatively determines the fate of Philadelphia chromosome-positive leukemia (part 5 of 5)
Source: eLife. 2024 Jun 12;12:RP88375. doi: 10.7554/eLife.88375 (PMC11168747; doi:10.7554/eLife.88375)

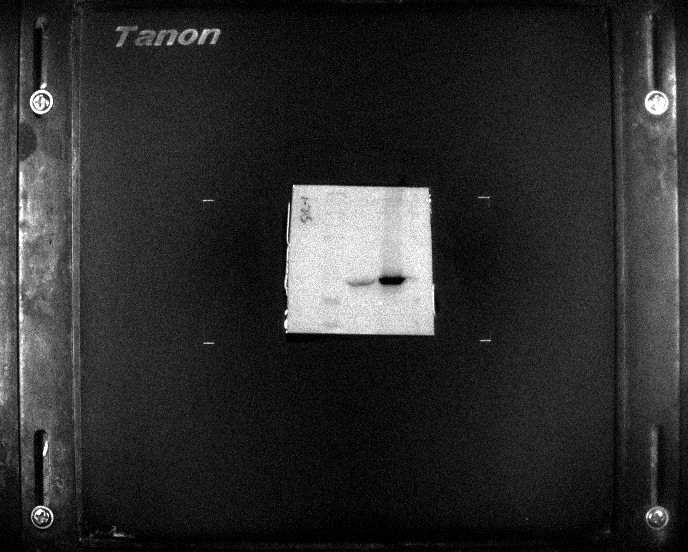

Supplement: Figure 5—source data 5. [file elife-88375-fig5-data5.zip › Figure 5-source data 5/MEG-01 Input SRC.tif]

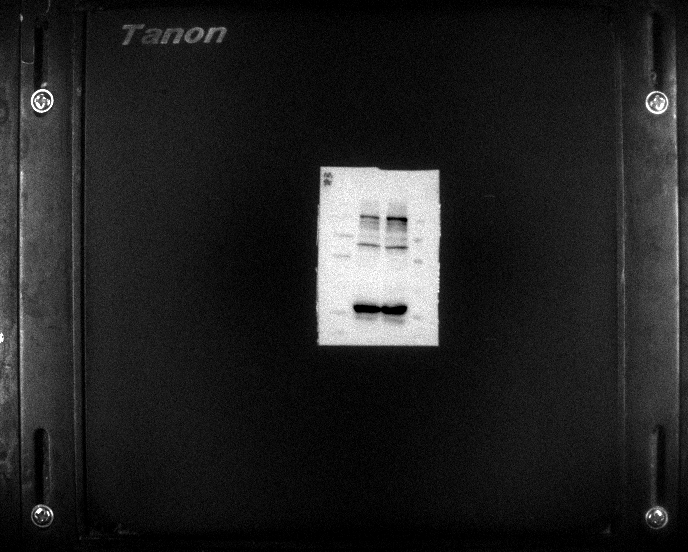

Supplement: Figure 5—source data 5. [file elife-88375-fig5-data5.zip › Figure 5-source data 5/MEG-01 IP BCR-ABL-IB BCR-ABL.tif]

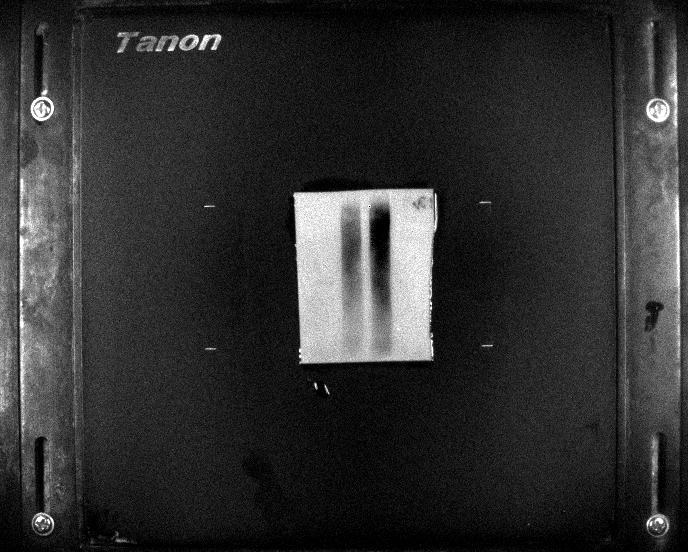

Supplement: Figure 5—source data 5. [file elife-88375-fig5-data5.zip › Figure 5-source data 5/MEG-01 IP BCR-ABL-IB NEDD8.tif]

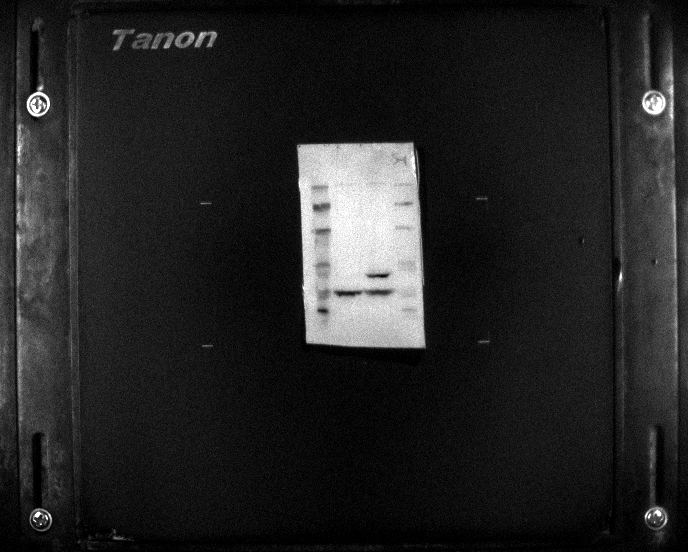

Supplement: Figure 5—source data 5. [file elife-88375-fig5-data5.zip › Figure 5-source data 5/MEG-01 a┬-Tubulin.tif]

C

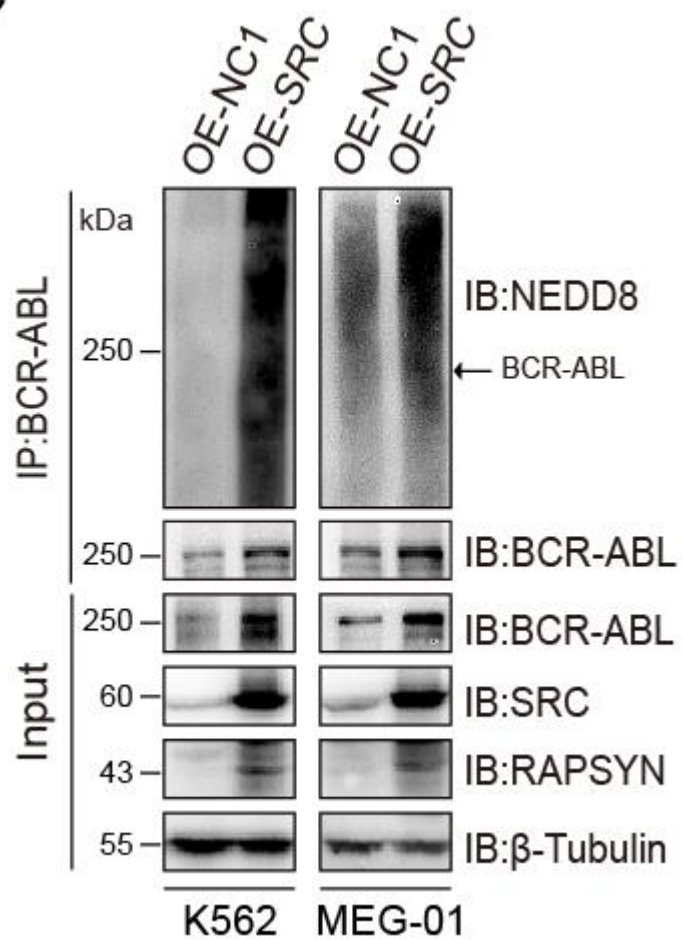

K562

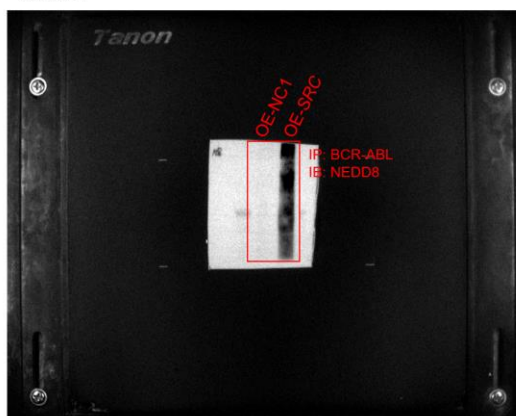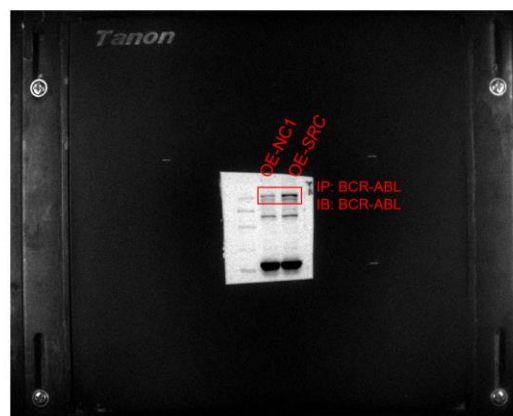

## Input

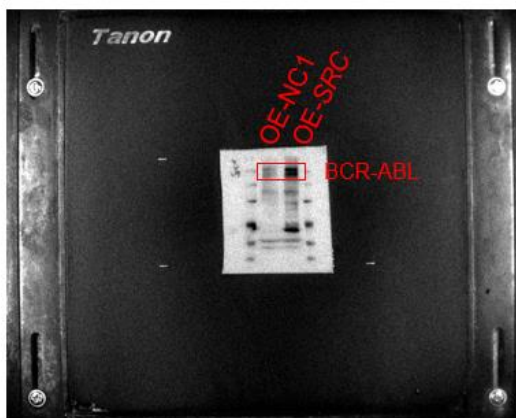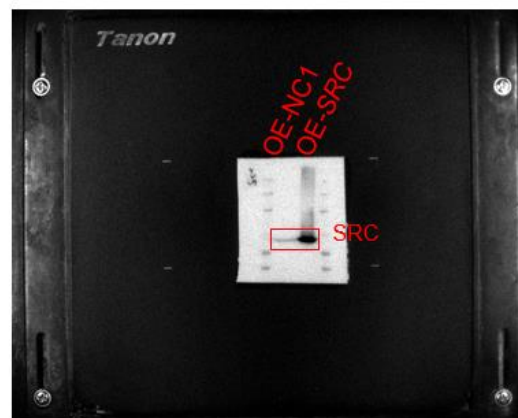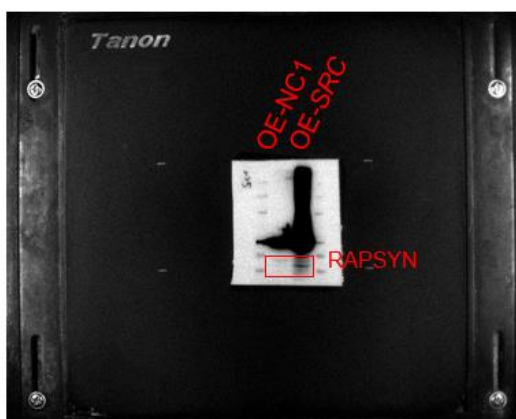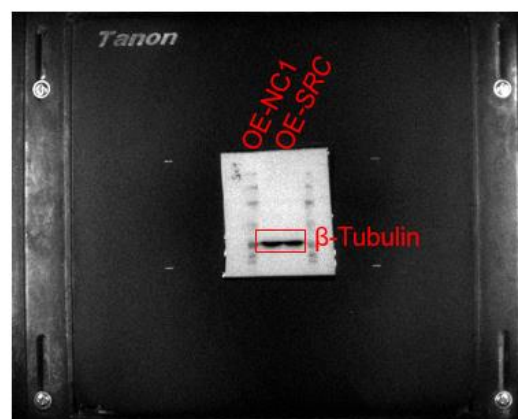

## MEG-01

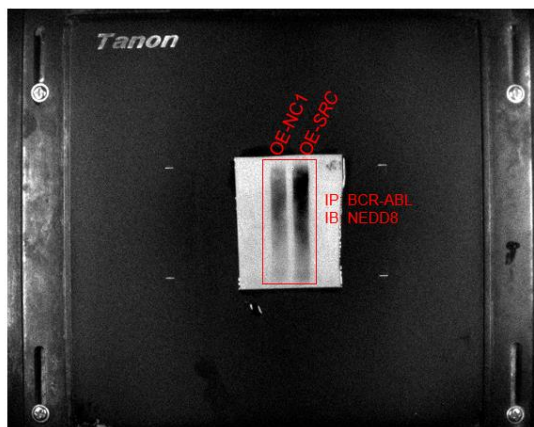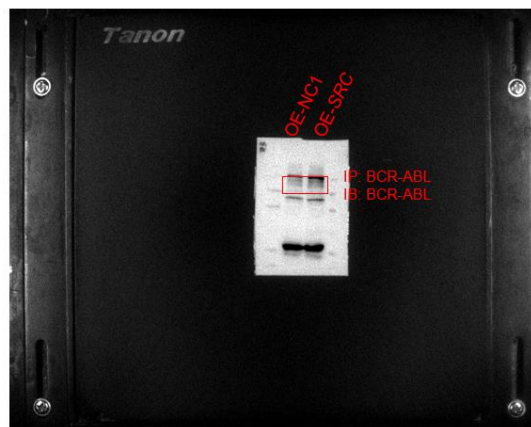

## Input

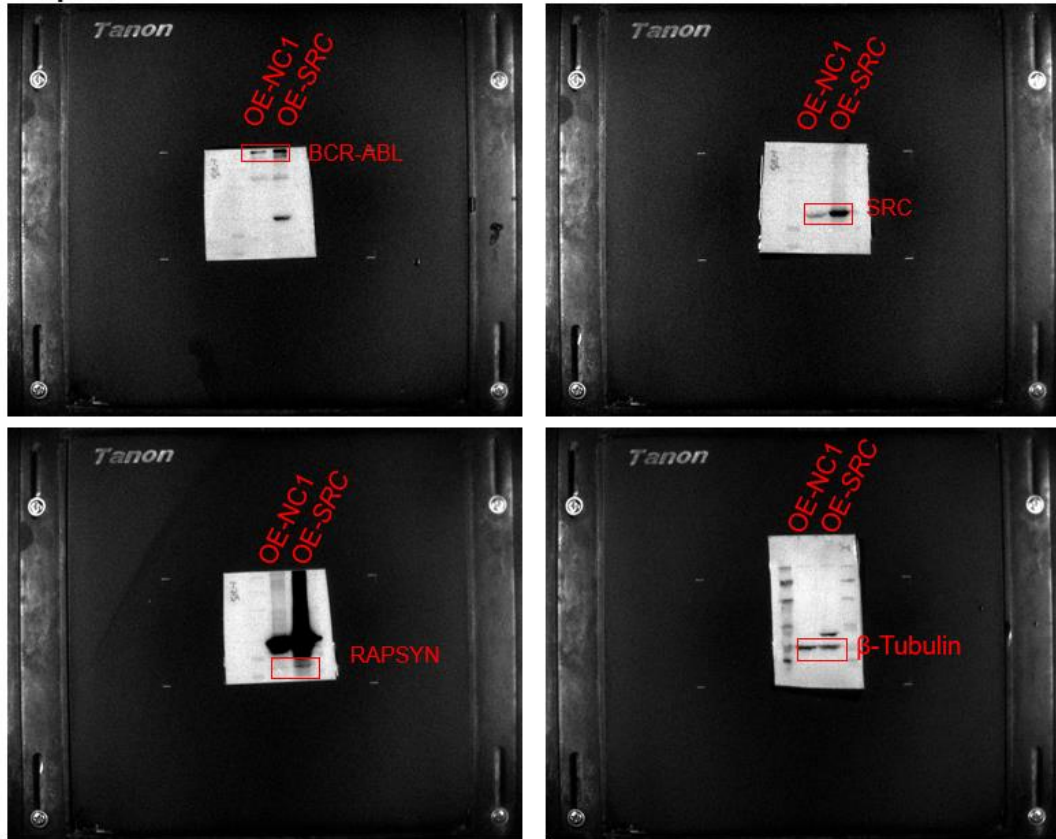

Supplement: Figure 5—source data 6. [file elife-88375-fig5-data6.zip › Figure 5-source data 6/Figure 5-source data 6.pdf]

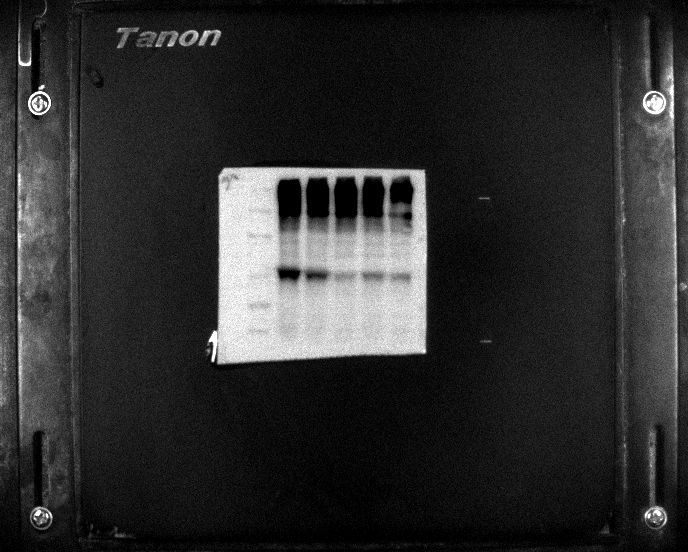

Supplement: Figure 5—source data 7. [file elife-88375-fig5-data7.zip › Figure 5-source data 7/Input GFP-2.tif]

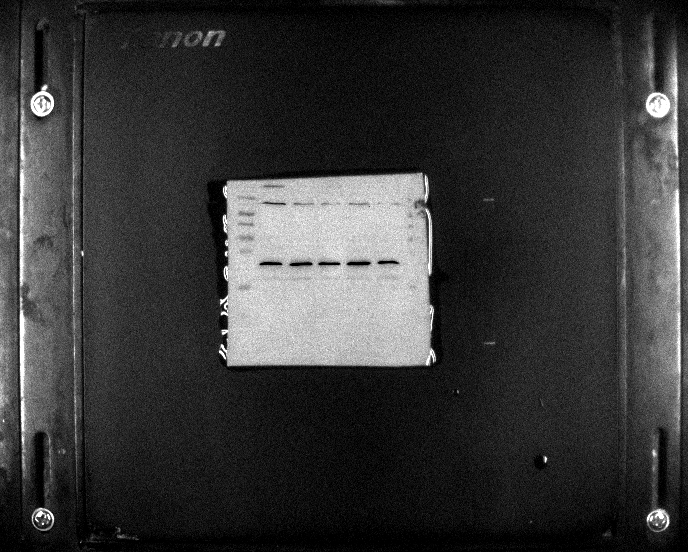

Supplement: Figure 5—source data 7. [file elife-88375-fig5-data7.zip › Figure 5-source data 7/Input HA-2.tif]

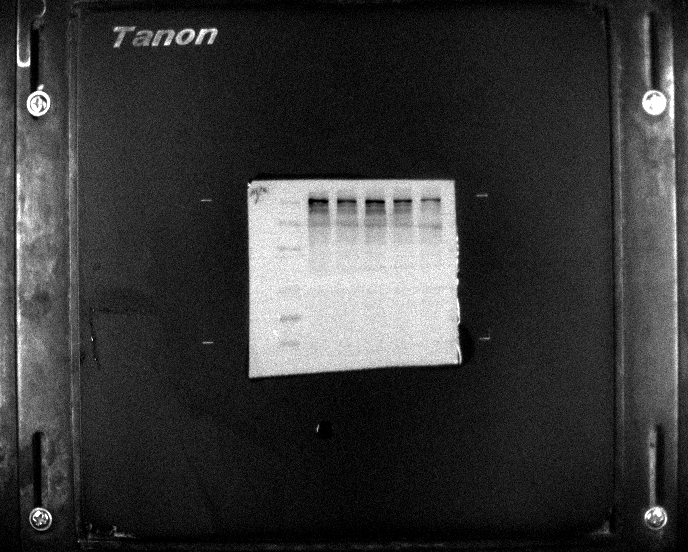

Supplement: Figure 5—source data 7. [file elife-88375-fig5-data7.zip › Figure 5-source data 7/Input His-2.tif]

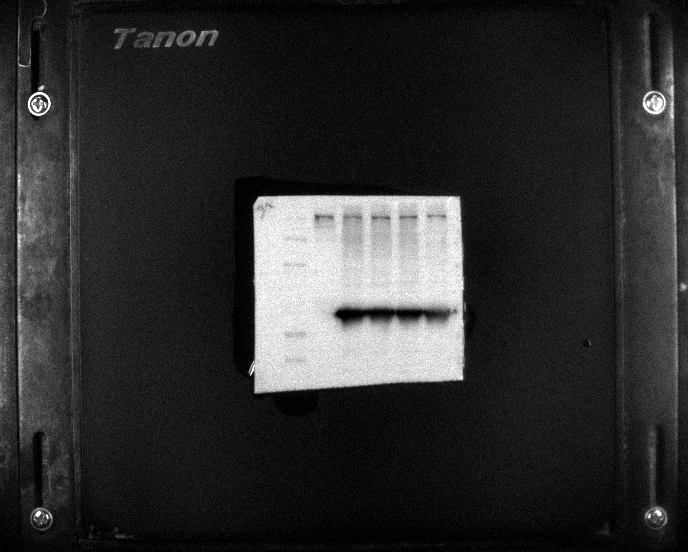

Supplement: Figure 5—source data 7. [file elife-88375-fig5-data7.zip › Figure 5-source data 7/Input Myc-4.tif]

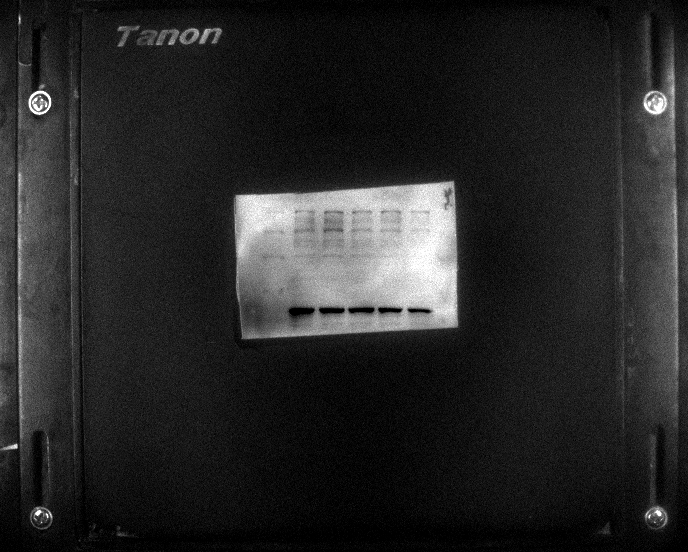

Supplement: Figure 5—source data 7. [file elife-88375-fig5-data7.zip › Figure 5-source data 7/IP His-IB His.tif]

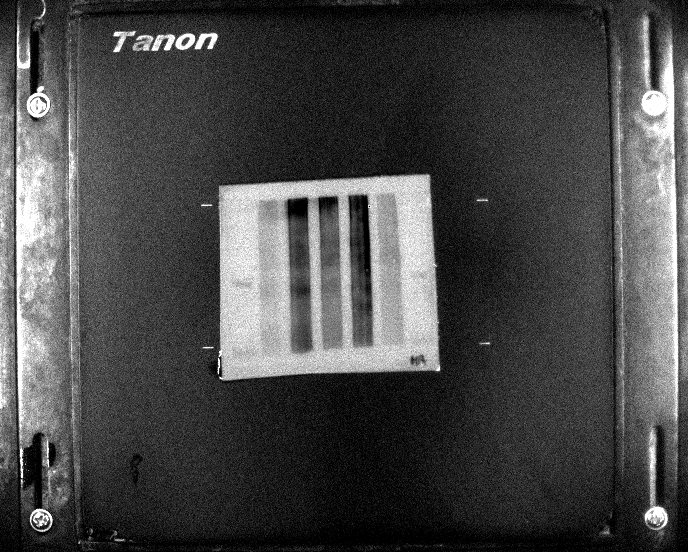

Supplement: Figure 5—source data 7. [file elife-88375-fig5-data7.zip › Figure 5-source data 7/IP His-IBHA.tif]

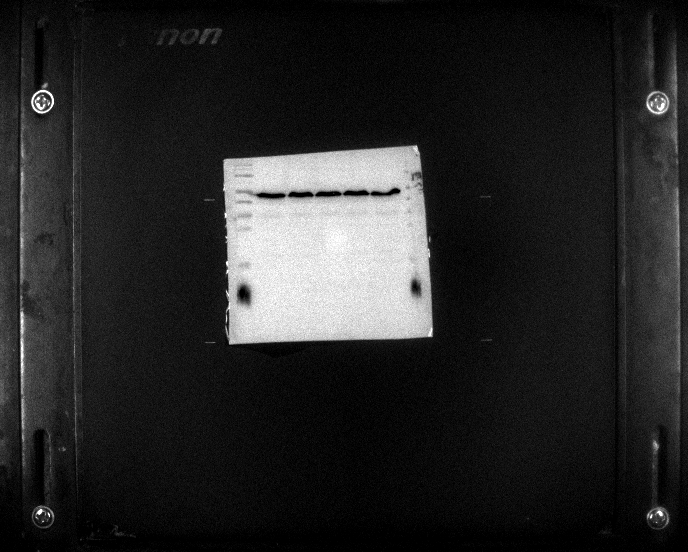

Supplement: Figure 5—source data 7. [file elife-88375-fig5-data7.zip › Figure 5-source data 7/Tubulin-6.tif]

D

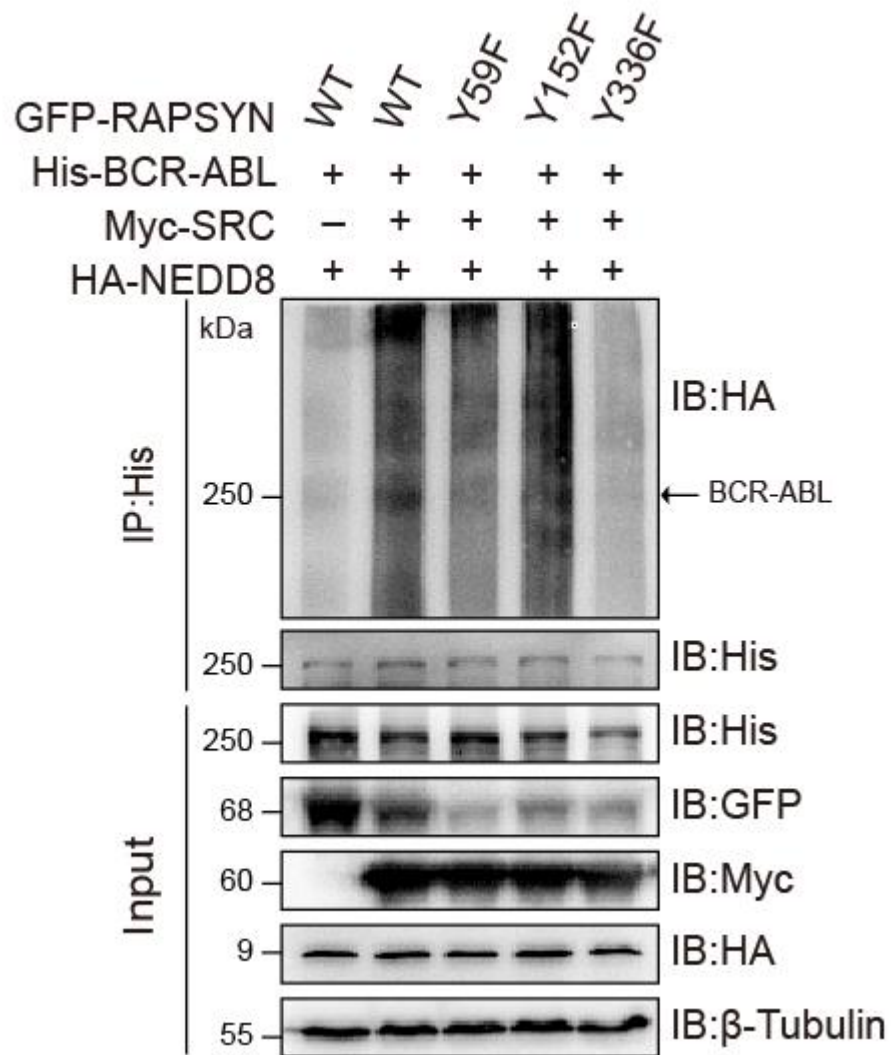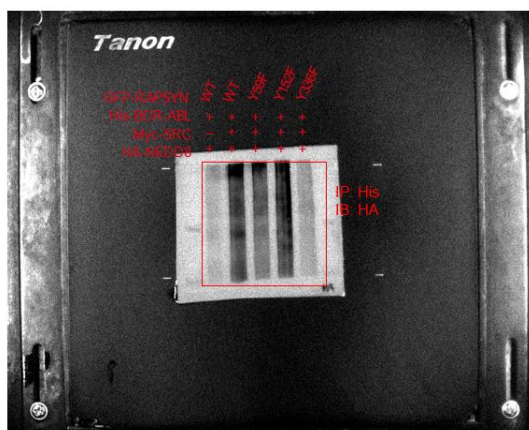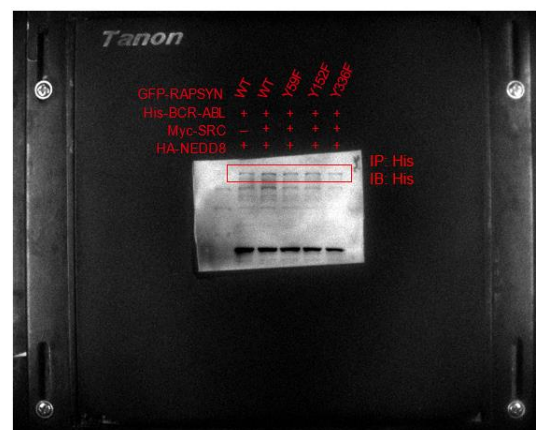

## Input

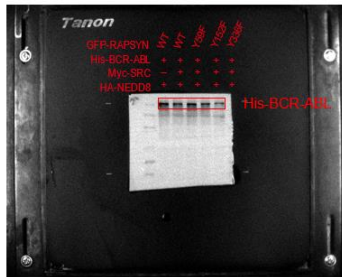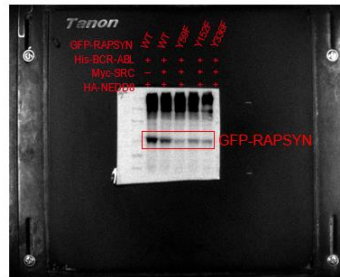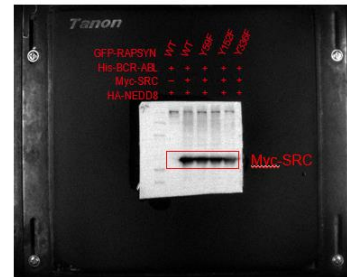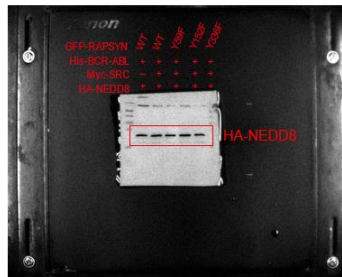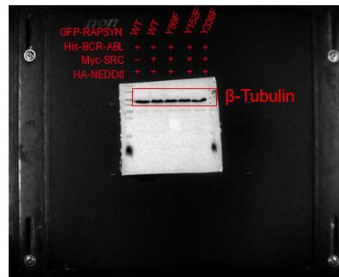

Supplement: Figure 5—source data 8. [file elife-88375-fig5-data8.zip › Figure 5-spurce data 8/Figure 5-source data 8.pdf]

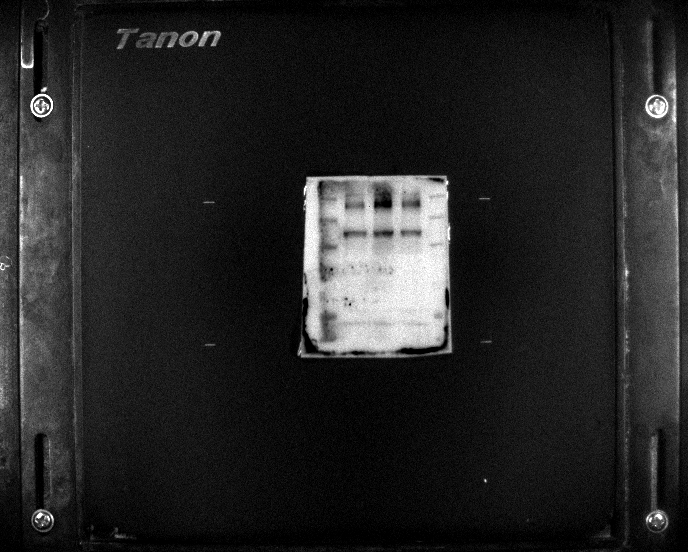

Supplement: Figure 5—source data 9. [file elife-88375-fig5-data9.zip › Figure 5-source data 9/K562 Input BCR-ABL.tif]

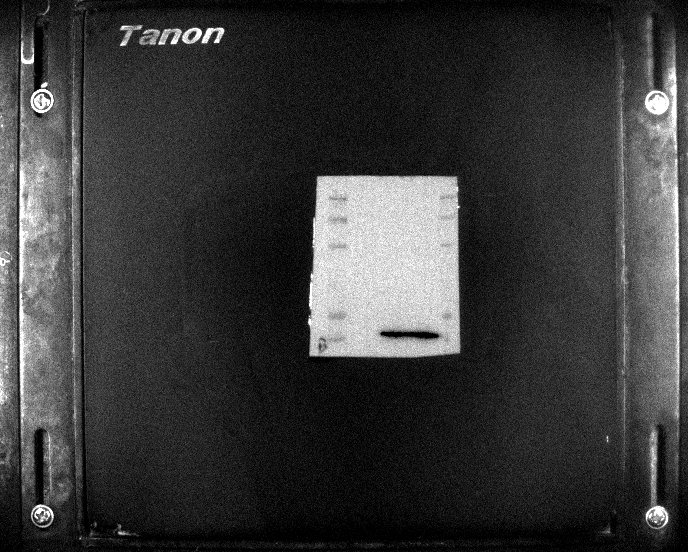

Supplement: Figure 5—source data 9. [file elife-88375-fig5-data9.zip › Figure 5-source data 9/K562 Input RAPSYN.tif]

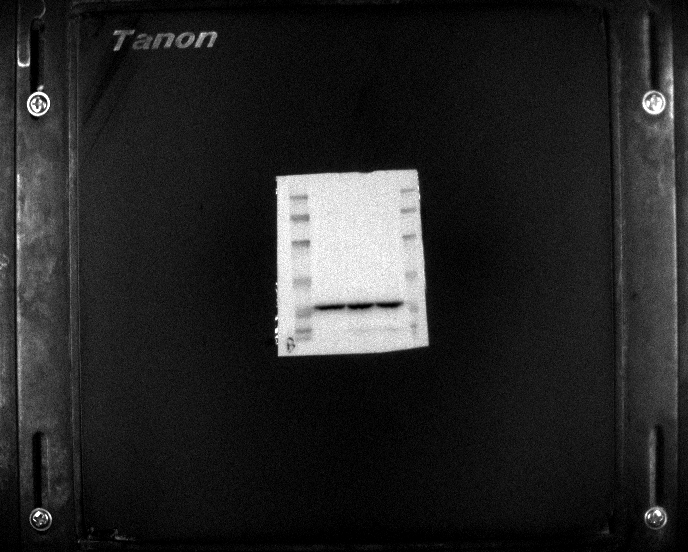

Supplement: Figure 5—source data 9. [file elife-88375-fig5-data9.zip › Figure 5-source data 9/K562 Input a┬-Tubulin.tif]

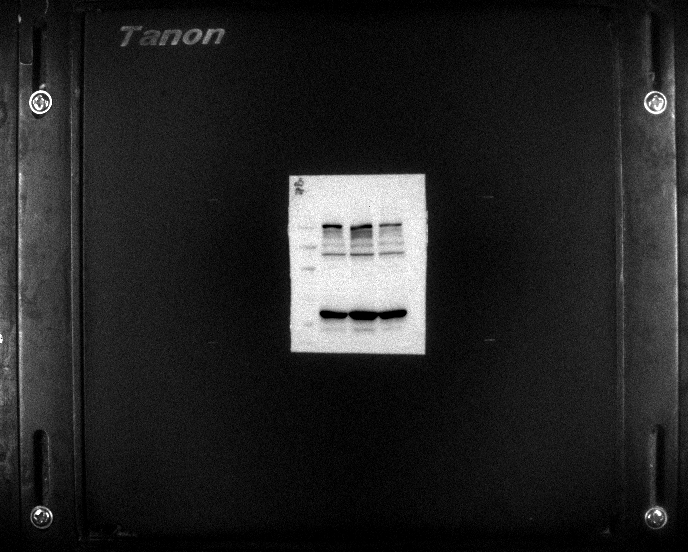

Supplement: Figure 5—source data 9. [file elife-88375-fig5-data9.zip › Figure 5-source data 9/K562 IP BCR-ABL-IB BCR-ABL.tif]

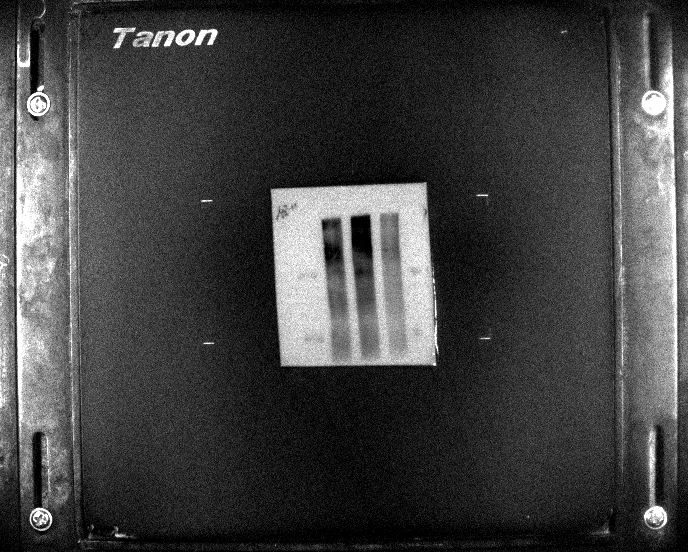

Supplement: Figure 5—source data 9. [file elife-88375-fig5-data9.zip › Figure 5-source data 9/K562 IP BCR-ABL-IB NEDD8.tif]

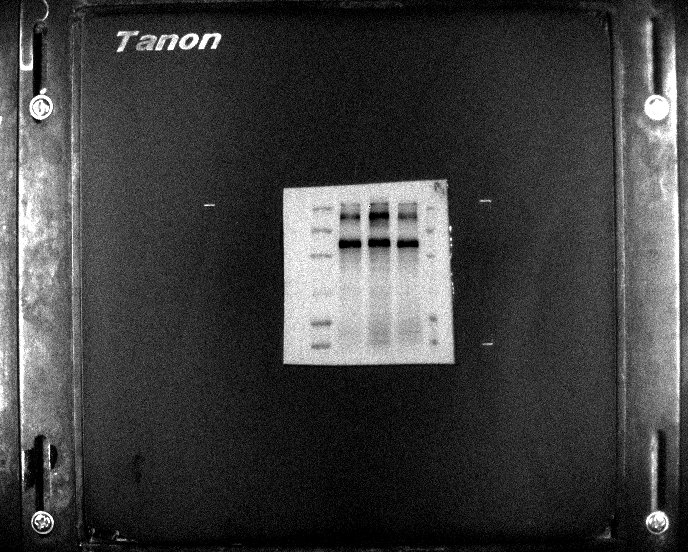

Supplement: Figure 5—source data 9. [file elife-88375-fig5-data9.zip › Figure 5-source data 9/MEG-01 Input BCR-ABL.tif]

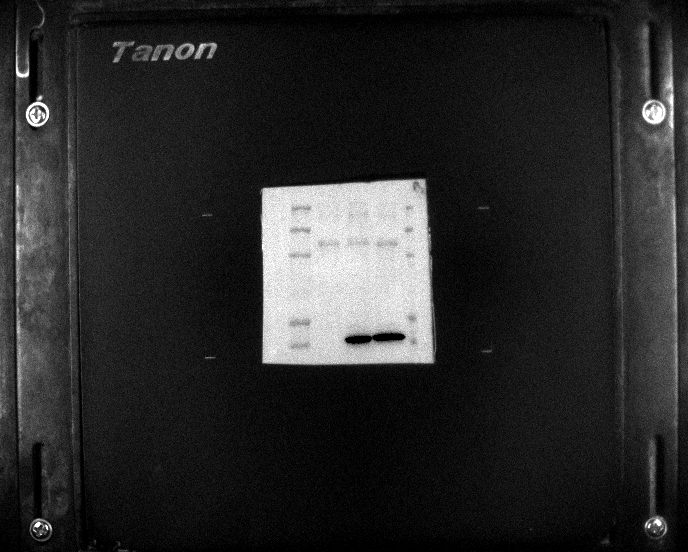

Supplement: Figure 5—source data 9. [file elife-88375-fig5-data9.zip › Figure 5-source data 9/MEG-01 Input RAPSYN.tif]

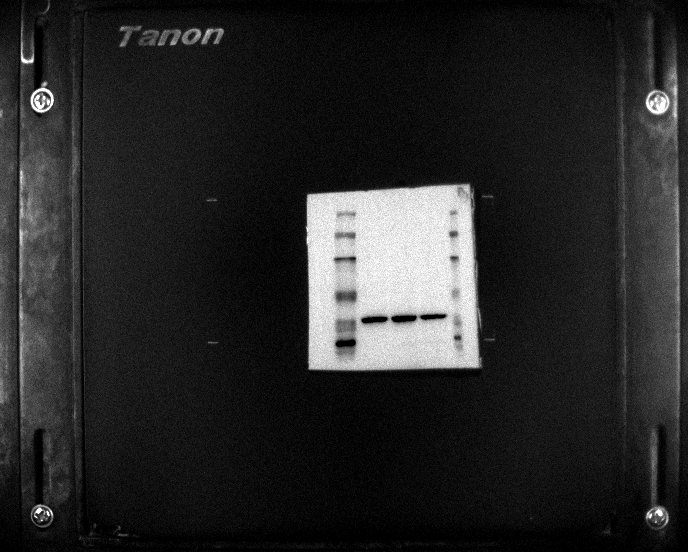

Supplement: Figure 5—source data 9. [file elife-88375-fig5-data9.zip › Figure 5-source data 9/MEG-01 Input a┬-Tubulin.tif]

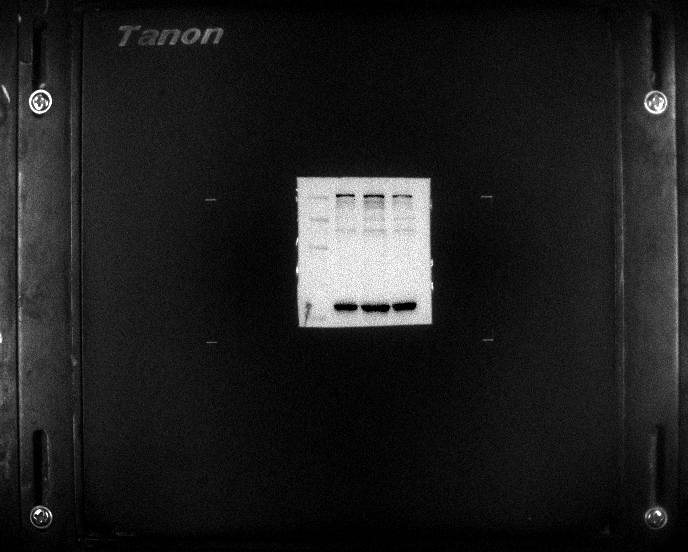

Supplement: Figure 5—source data 9. [file elife-88375-fig5-data9.zip › Figure 5-source data 9/MEG-01 IP BCR-ABL-IB BCR-ABL.tif]

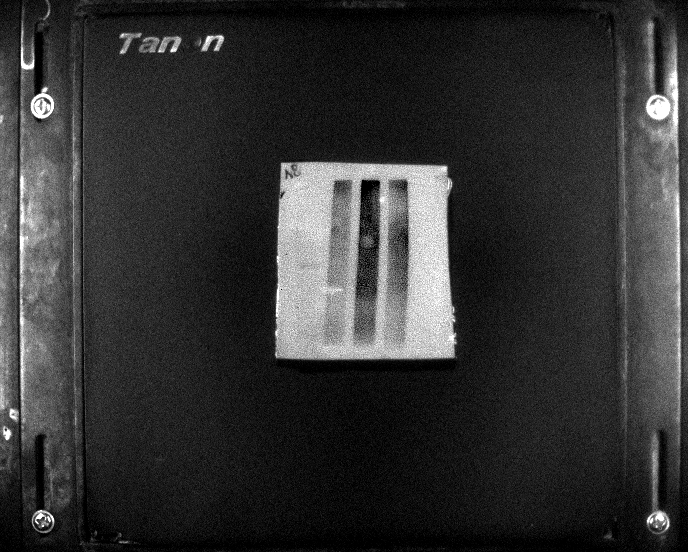

Supplement: Figure 5—source data 9. [file elife-88375-fig5-data9.zip › Figure 5-source data 9/MEG-01 IP BCR-ABL-IB NEDD8.tif]

E

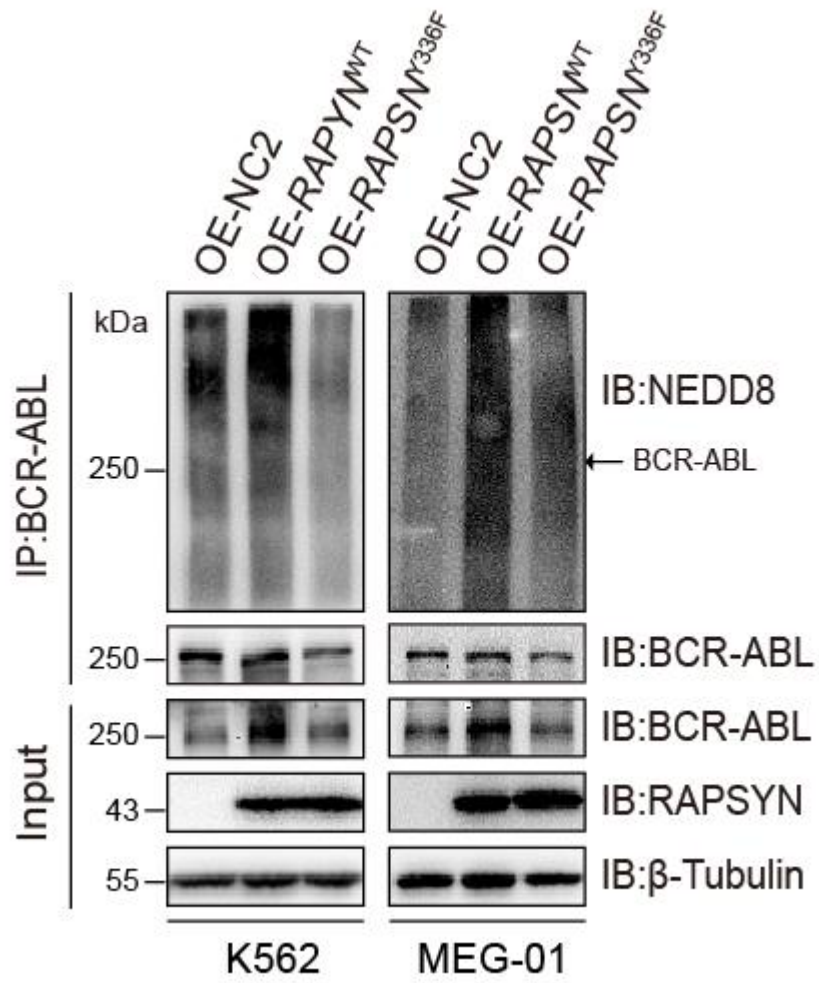

K562

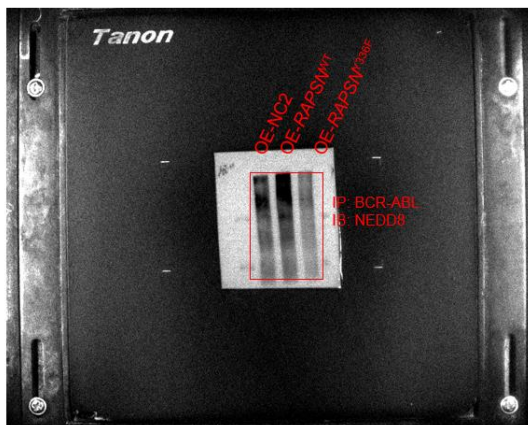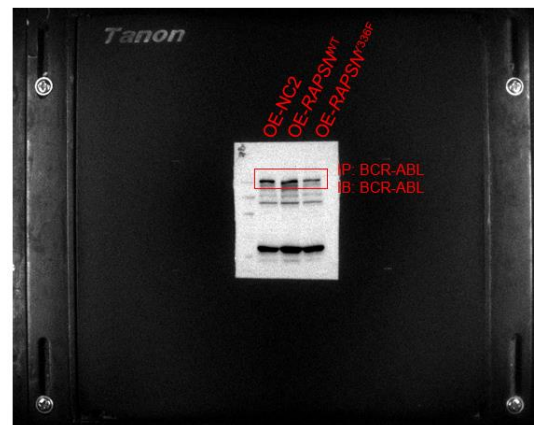

Input

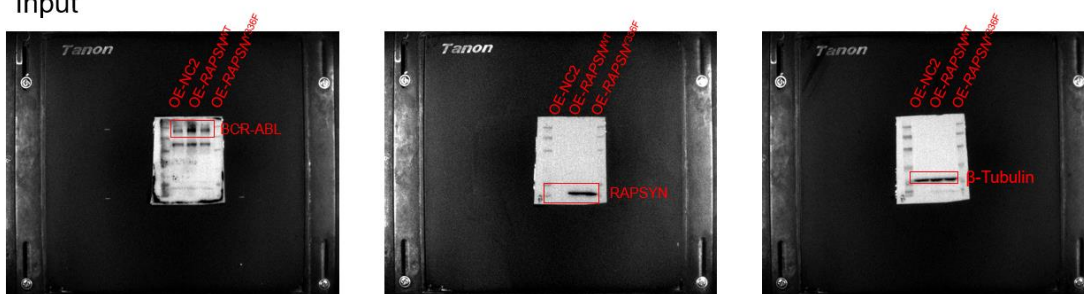

MEG-01

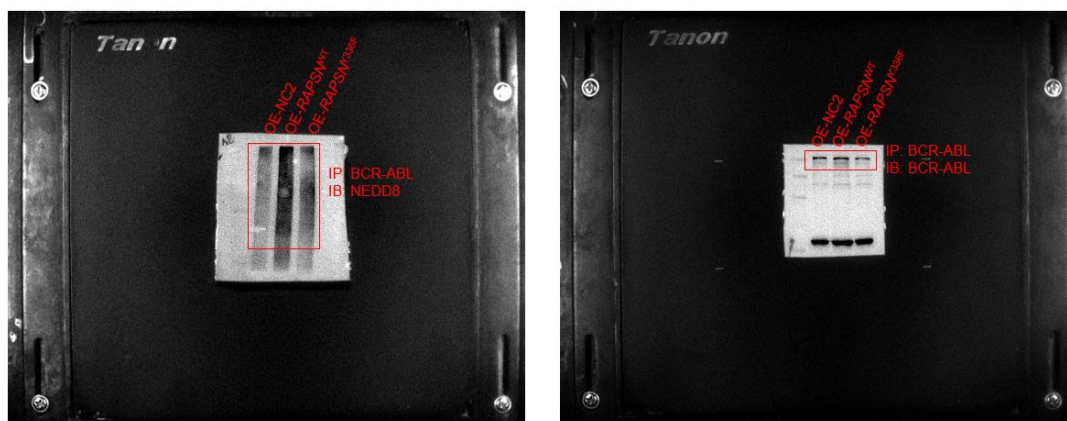

Input

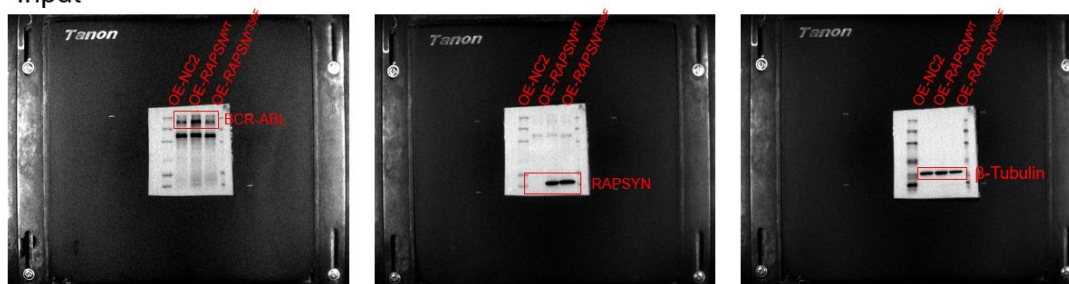

Supplement: Figure 5—source data 10. [file elife-88375-fig5-data10.zip › Figure 5-source data 10/Figure 5-source data 10.pdf]

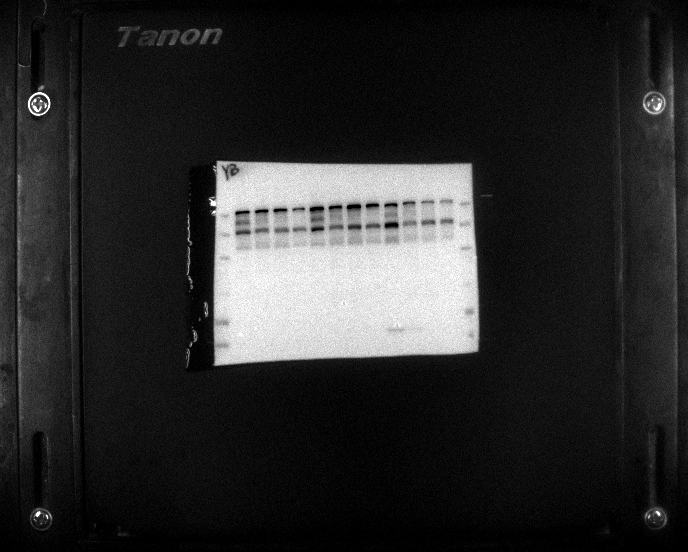

Supplement: Figure 5—source data 11. [file elife-88375-fig5-data11.zip › Figure 5-source data 11/K562 BCR-ABL.tif]

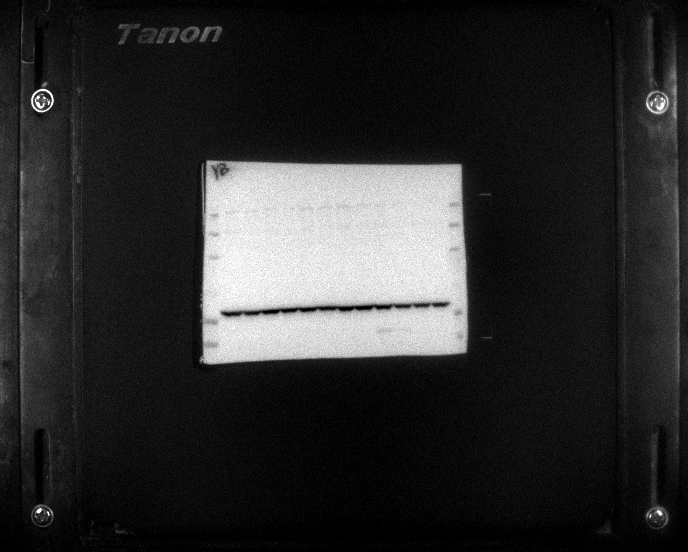

Supplement: Figure 5—source data 11. [file elife-88375-fig5-data11.zip › Figure 5-source data 11/K562 a┬-Tubulin.tif]

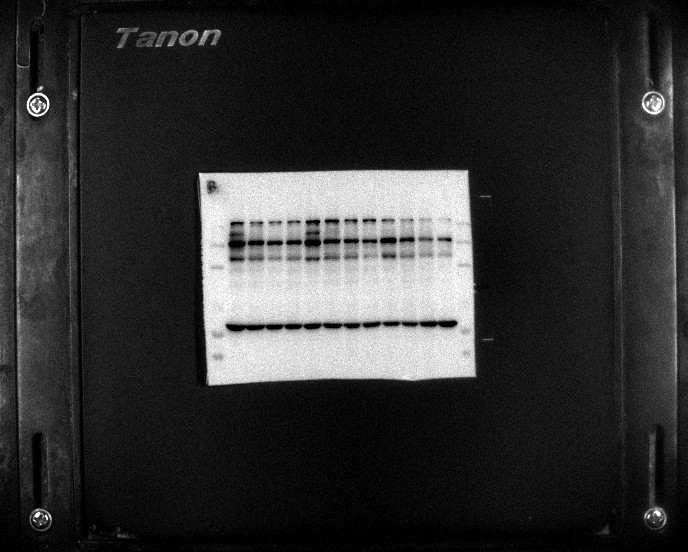

Supplement: Figure 5—source data 11. [file elife-88375-fig5-data11.zip › Figure 5-source data 11/MEG-01 BCR-ABL.tif]

F

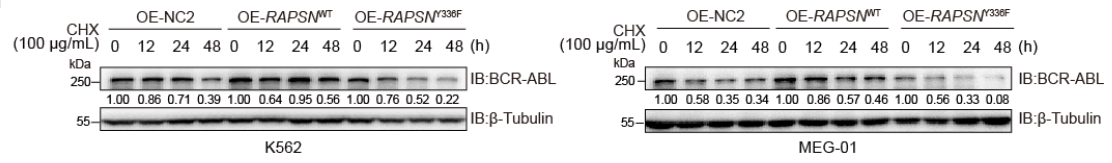

K562

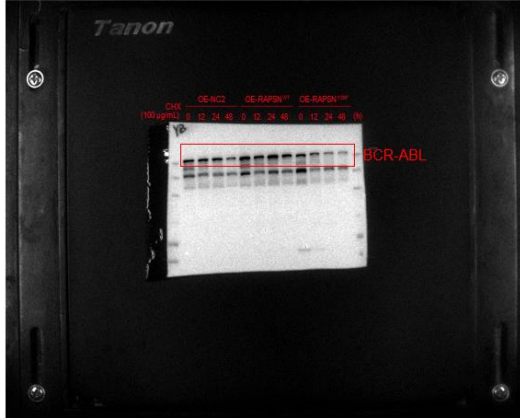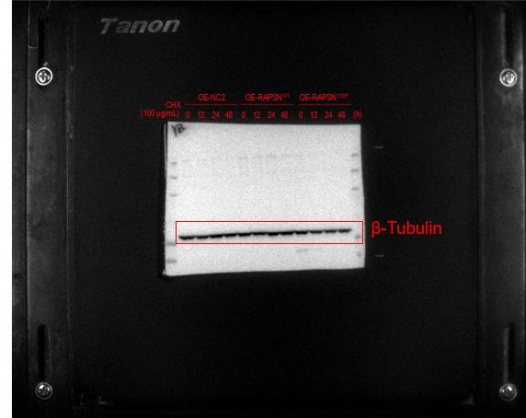

MEG-01

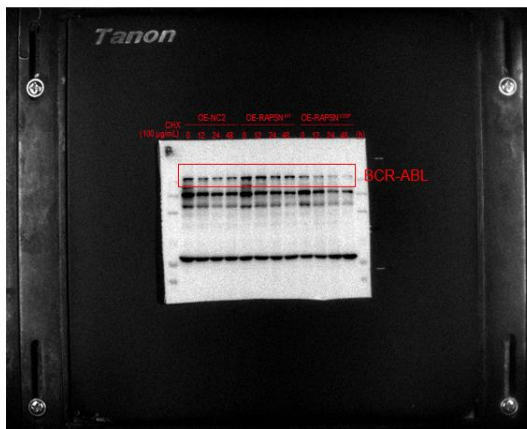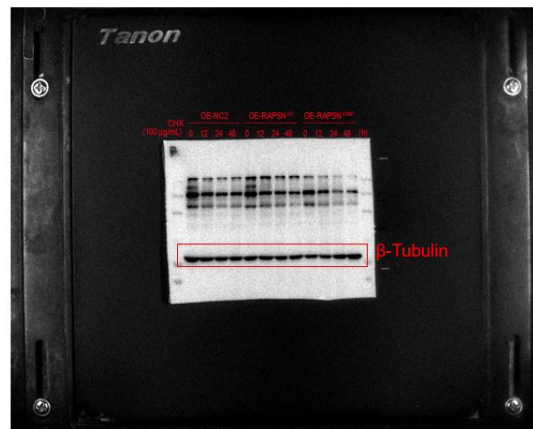

Supplement: Figure 5—source data 12. [file elife-88375-fig5-data12.zip › Figure 5-source data 12/Figure 5-source data 12.pdf]
