# Supplementary material for: RAPSYN-mediated neddylation of BCR-ABL alternatively determines the fate of Philadelphia chromosome-positive leukemia
Source: eLife. 2024 Jun 12;12:RP88375. doi: 10.7554/eLife.88375 (PMC11168747; doi:10.7554/eLife.88375)
Supplement: Supplementary file 1. [file elife-88375-supp1.docx]

**Supplementary File1 Sequences of shRNA and primers**

| Name | Gene symbol (ID) | NCBI Reference Sequence | Sequence |
| --- | --- | --- | --- |
| shRNA targeting sequence: RAPSYN #1 | RAPSYN (5913) | NM_005055.5 | GCATTGCAGGTGTGGACAAAG |
| shRNA targeting sequence: RAPSYN #2 |  |  | GGAGTGTTGTGAGGAGTCTAT |
| shRNA targeting sequence: RAPSYN #3 |  |  | TGCACGCCAGAGGCCCATTTA |
| shRNA targeting sequence: SRC #1 | SRC (6714) | NM_005417.5 | GAGGGACCCTTCGAGATCATCACTT |
| shRNA targeting sequence: SRC #2 |  |  | CATCCTCAGGAACCAACAATT |
| shRNA targeting sequence: SRC #3 |  |  | CAGGTGTGGAGAGAGAGGCTTCAAT |
| shRNA targeting sequence: SRC #4 |  |  | GCGTCCATATTTAACATGTAA |
| shRNA targeting sequence: SRC #5 |  |  | GGTTGTAAATACTTTGCATATTGTC |
| h. RAPSYN RT-PCR Primers | RAPSYN (5913) | NM_005055.5 | Forward: CGCTACAAGGAGATGCTGAAG |
|  |  |  | Reverse: CTTGCAGTAGGAGATGGTCTTG |
| h. ACTIN RT-PCR Primers | ACTB (60) | NM_001101.5 | Forward: CTACAATGAGCTGCGTGTGGC |
|  |  |  | Reverse: CAGGTCCAGACGCAGGATGGC |
| h. GAPDH RT-PCR Primers | GAPDH (2597) | NM_002046.7 | Forward: GCGTGACATTAAGGAGAAG |
|  |  |  | Reverse: GAAGGAAGGCTGGAAGAG |
| HA-NEDD8 △GG mutant primers | NEDD8 (4738) | NM_006156.3 | Forward: GTTGGCTCTGAGAGCAGCTGGTGGTCTTAGGC |
|  |  |  | Reverse: GCCTAAGACCACCAGCTGCTCTCAGAGCCAAC |
| GFP-RAPSYN C366A mutant primers | RAPSYN (5913) | NM_005055.5 | Forward: TCTACTGCGGCCTGGCCGGCGAGTCCATAG |
|  |  |  | Reverse: CTATGGACTCGCCGGCCAGGCCGCAGTAGA |
| GST-RAPSYN Y59F mutant primers |  |  | Forward: GGAGATGGGCCGCTTTAAGGAGATGCTGAAGTT |
|  |  |  | Reverse: AACTTCAGCATCTCCTTAAAGCGGCCCATCTCC |
| GST-RAPSYN Y152F mutant primers |  |  | Forward: GAGAAGGCCCTGCGCTTTGCCCACAACAATGAT |
|  |  |  | Reverse: ATCATTGTTGTGGGCAAAGCGCAGGGCCTTCTC |
| GST-RAPSYN Y336F mutant primers |  |  | Forward: TGAGCGAGAGCATTTTTCGCAGCAAAGGGCTC |
|  |  |  | Reverse: GCAGCCCTTTGCTGCGAAAAATGCTCTCGCTCA |
| His-BCR-ABL K257R mutant primers |  |  | Forward: CCCCCGCTTCCTGAGGGACAACCTGATCGAC |
|  |  |  | Reverse: GTCGATCAGGTTGTCCCTCAGGAAGCGGGGG |
| His-BCR-ABL K500R mutant primers |  |  | Forward: GGCTTGGAGATGAGAAGATGGGTCCTGTCGGG |
|  |  |  | Reverse: CCCGACAGGACCCATCTTCTCATCTCCAAGCC |
| His-BCR-ABL K739R mutant primers |  |  | Forward: CTGCTTCTCTGCACCAGGCTCAAGAAGCAGAGC |
|  |  |  | Reverse: GCTCTGCTTCTTGAGCCTGGTGCAGAGAAGCG |
| His-BCR-ABL K802R mutant primers |  |  | Forward: GACATCCAGAGAGAGAGGAGGGCGAACAAGGC |
|  |  |  | Reverse: GCCCTTGTTCGCCCTCCTCTCTCTCTGGATGTC |
| His-BCR-ABL K1025R mutant primers |  |  | Forward: GTCAACAGTCTGGAGAGACACTCCTGGTACCAT |
|  |  |  | Reverse: ATGGTACCAGGAGTGTCTCTCCAGACTGTTGAC |
| His-BCR-ABL K1135R mutant primers |  |  | Forward: TCCCCCAACTACGACAGGTGGGAGATGGAACC |
|  |  |  | Reverse: GCGTTCCATCTCCCACCTGTCGTAGTTGGGGGA |
| His-BCR-ABL K1590R mutant primers |  |  | Forward: CCCACCTGTGGAAGAGGTCCAGCACGCTGAC |
|  |  |  | Reverse: GTCAGCGTGCTGGACCTCTTCCACAGGTGGG |
| His-BCR-ABL K1990R mutant primers |  |  | Forward: CGAGAGGCCATCAACAGACTGGAGAATAATCTC |
|  |  |  | Reverse: GAGATTATTCTCCAGTCTGTTGATGGCCTCTCG |
